# Longitudinal physical-mental multimorbidity trajectories by sociodemographic factors: a cohort study using electronic health records in Scotland (2000–2021)

**DOI:** 10.1371/journal.pone.0359871

**Published:** 2026-10-15

**Authors:** Thu Nguyen, Katherine Keenan, Colin McCowan, Michail Papathomas, Areti Manataki

**Affiliations:** 1 School of Computer Science, University of St Andrews, St Andrews, United Kingdom; 2 School of Geography and Sustainable Development, University of St Andrews, St Andrews, United Kingdom; 3 Division of Population and Behavioural Sciences, School of Medicine, University of St Andrews, St Andrews, United Kingdom; 4 School of Mathematics and Statistics, University of St Andrews, St Andrews, United Kingdom; University of Illinois Urbana-Champaign, UNITED STATES OF AMERICA

## Abstract

Multimorbidity, the co-occurrence of two or more chronic conditions, is a pressing global health challenge. However, research capturing the dynamic, longitudinal nature of health trajectories, particularly the intersection of physical and mental domains, remains scarce compared to traditional cross-sectional “snapshots.” Leveraging up to 21 years of electronic health records data from 858,789 Scottish patients in the University of Dundee Health Informatics Centre, we used multistate modelling to explore how individuals transition from a disease-free baseline into multimorbidity, evaluating how age, sex, and socioeconomic status shape these pathways. Overall transition estimates were driven by the younger demographic of our baseline disease-free cohort. The findings highlight the critical role of severe, hospital-contacting mental health conditions in driving early-onset multimorbidity, particularly in young men and socioeconomically disadvantaged groups. These findings underscore the urgent need to prioritise integrated physical-mental care and address upstream structural determinants of health. This temporal approach identifies high-risk populations for targeted early interventions and informs policy initiatives aimed at reducing health disparities.

## Introduction

Multimorbidity – “the coexistence of two or more chronic conditions in the same individual” [1] – is one of the most pressing challenges facing healthcare systems worldwide now and in the coming years [2]. It is estimated that around one in four adults in England [3,4] and one-third of adults worldwide [5] have multimorbidity. Unlike comorbidity, which is the combined effects of additional diseases co-existing with a particular index condition of interest, multimorbidity care is patient-centred and does not prioritise any disease [6]. Patients with multimorbidity are at a greater risk of premature mortality, lower quality of life, polypharmacy, psychological distress, and frequent and complex interaction with healthcare services [6–10]. During the COVID-19 pandemic, multimorbid people were put in a further vulnerable state and at higher risk of being admitted to hospital and having adverse outcomes [11,12]. Multimorbidity is also associated with higher treatment burden and higher healthcare cost [6,13]. Care for multimorbid patients in the UK accounts for more than 55% of NHS costs and 75% of primary care prescription costs [3].

As populations age and the burden of chronic diseases rises, health systems face the complexity of managing multiple interacting conditions, often spanning physical and mental health domains. Previous studies show that mental health issues, including mood disorders (e.g., depression), severe psychiatric conditions (e.g., schizophrenia), and substance abuse, often co-occur with physical diseases such as diabetes, heart disease and HIV [14,15]. Patients with physical and mental health conditions tend to have poorer health outcomes, a reduced quality of life, and a higher risk of premature death than people who have only physical diseases [16–18]. Although the combined adverse impact of mental and physical multimorbidity is well established, less than half of the multimorbidity studies include any single mental health condition, highlighting a clear gap in the literature [19]. Depression, dementia, and anxiety are the most common mental health conditions that are included [19]. Other mental conditions, such as schizophrenia, alcohol and drug use disorders, and bipolar disorder, are only considered in fewer than 20% of multimorbidity studies [19]. The relationship between mental and physical health is often complex and is believed to be bidirectional [8,14]. However, most data come from cross-sectional studies [8], which do not give any evidence on whether physical illness leads to mental health problems or vice versa.

Studies on multimorbidity have largely focused on older population. A recent review by Ho et al. [19] revealed a significant gap in the literature on early-onset multimorbidity. While most studies focused on older adults or unrestricted age cohorts, only 1.8% restricted their research exclusively to young and middle-aged adults aged 18–64. Research exploring multimorbidity across age groups, especially in adults under 65 years of age, is generally small, even though there are more people in the population in this age group and in absolute numbers there are a higher number of them affected by multimorbidity than older adults [8]. We emphasise this gap because our study design, which tracks incident disease trajectories from a disease-free baseline, offers a unique perspective on how multimorbidity develops in this historically under-researched younger demographic.

Despite the growing body of evidence on its prevalence, the temporal dynamics of transitioning from disease-free to multimorbidity, and the roles of physical and mental health trajectories in this process, especially for young people, remain poorly understood. Therefore, we sought to explore the development of physical and mental health conditions in adults aged 18 years and older over the course of their life. We used large, population-scale, individual-level electronic health records from the Health Informatics Centre, University of Dundee, which included a random 80% sample of all individuals who were 18 years and older in the Tayside and Fife NHS secondary care in Scotland.

The growing burden of multimorbidity not only strains healthcare services, but also contributes to health inequalities. We also sought to examine the role of socioeconomic status, age, and sex to uncover the social aspects of multimorbidity and provide further insights into the potential contributors to specific physical and mental health pathways over time. A life course approach is particularly crucial to gain a deeper understanding of the development of multimorbidity [20] and its inequalities from social status, as it can reveal the early preventable factors that contribute to the onset of multimorbidity and later life factors that influence the accumulation of additional diseases. This can ultimately inform equitable prevention, integrated care delivery, and long-term health system planning.

In general, this study aimed to characterise the progression of physical-mental multimorbidity in adults aged 18 years and older and to identify demographic and social patterns in disease accumulation and outcomes.

## Methods

### Study design

We conducted a longitudinal cohort study using de-identified electronic health records held in the Health Informatics Centre (HIC). The dataset included a random 80% sample of individuals aged 18 years and older (n = 981,879) in NHS Tayside and Fife (Scotland) secondary care at any point between 1 January 2000–31 December 2021. This gave a maximum of 21 years follow-up for an individual. An 80% random sample of the available data was made available by the data provider. This was primarily done to support patient privacy and confidentiality as it was then unclear who exactly in the population has data within the study dataset. To capture the trajectories of disease accumulation and mortality over time, we applied a multistate modelling approach.

### Data variables and measurement

We identified disease diagnoses from electronic health records derived from NHS Tayside and Fife secondary care and mortality data from National Records Scotland. Hospitalisation data from Scottish Morbidity Records [21] included inpatient and day case admissions to acute and psychiatric hospitals, outpatient attendances, accident and emergency attendances, and cancer registry records.

Diagnoses were coded using a multimorbidity index [22], which categorises 55 chronic conditions across 14 body systems based on recorded ICD-10 diagnostic codes (S1 Table). These conditions encompassed a wide range of illnesses, including physical diseases (cardiovascular disease, respiratory disease, metabolic disorder, infectious disease, cancer, etc.) and mental and behavioural disorders (drug/alcohol misuse, dementia, depression, anxiety, schizophrenia, bipolar disorder, post-traumatic stress disorder (PTSD), autism and eating disorder). A condition was considered present if a relevant ICD-10 code was found in the patient’s record, and the diagnosis date was assigned to the date of first occurrence. Multimorbidity was defined as the co-occurrence of two or more chronic conditions from the listed conditions.

Our case identification system primarily captures severe, hospital-contacting events and misses conditions managed entirely in primary care. Thus, the overall burden of mental health is likely underrepresented. This particularly impacts women whose primary care-managed mental health conditions such as mild to moderate depression and anxiety likely contribute significantly to overall morbidity.

Demographic data, including age, sex assigned at birth, and area-level socioeconomic deprivation, was linked to health data. Deprivation was measured using the Scottish Index of Multiple Deprivation (SIMD), a composite index comprising income, employment, education, health, housing, access to services, and crime [23]. Individuals were assigned a SIMD quintile (ranging from 1 to 5, with 1 representing the most deprived and 5 the least deprived) based on their area of residence. Missing SIMD data were recorded as “missing” and retained as a separate category.

### Participants

Eligible participants were individuals 18 years and older residing in Tayside and Fife on or after 1 January 2005. To ensure participants enter in a comparatively disease-free state, a 5-year lookback period [24] (1 January 2000–31 December 2004) was used to exclude individuals with pre-existing diagnoses of any of the 55 conditions. The study followed the participants until their death or the end of the study period on 31 December 2021.

Participants who turned 18 during the study period were eligible for inclusion if they remained disease-free for at least five years before their 18th birthday. Follow-up for these participants began on the date they turned 18. Individuals aged 100 and older at cohort entry were excluded.

The final cohort comprised 858,789 individuals, all of whom had no secondary care diagnoses in the five years prior to the start of follow-up. The first diagnoses were captured from 1 January 2005, or from the date the participants turned 18 onwards.

Our cohort requires individuals to be free of all 55 conditions at baseline. Therefore, transition probabilities estimated should be interpreted as conditional probabilities, which depend on the individual having survived to that age and having no prior diseases for 5 years before follow-up.

### Statistical analysis

We used multistate Markov modelling to examine transitions between disease-free status, physical and mental morbidity, multimorbidity subtypes, and death. The model had seven mutually exclusive states and allowed 17 direct transitions (Fig 1):

State 1: Disease-free
State 2: Physical morbidity (1 physical condition)
State 3: Mental morbidity (1 mental condition)
State 4: Physical multimorbidity (2+ physical conditions)
State 5: Mental multimorbidity (2+ mental conditions)
State 6: Physical-mental multimorbidity (1+ physical and 1+ mental condition)
State 7: Death

**Fig 1:**
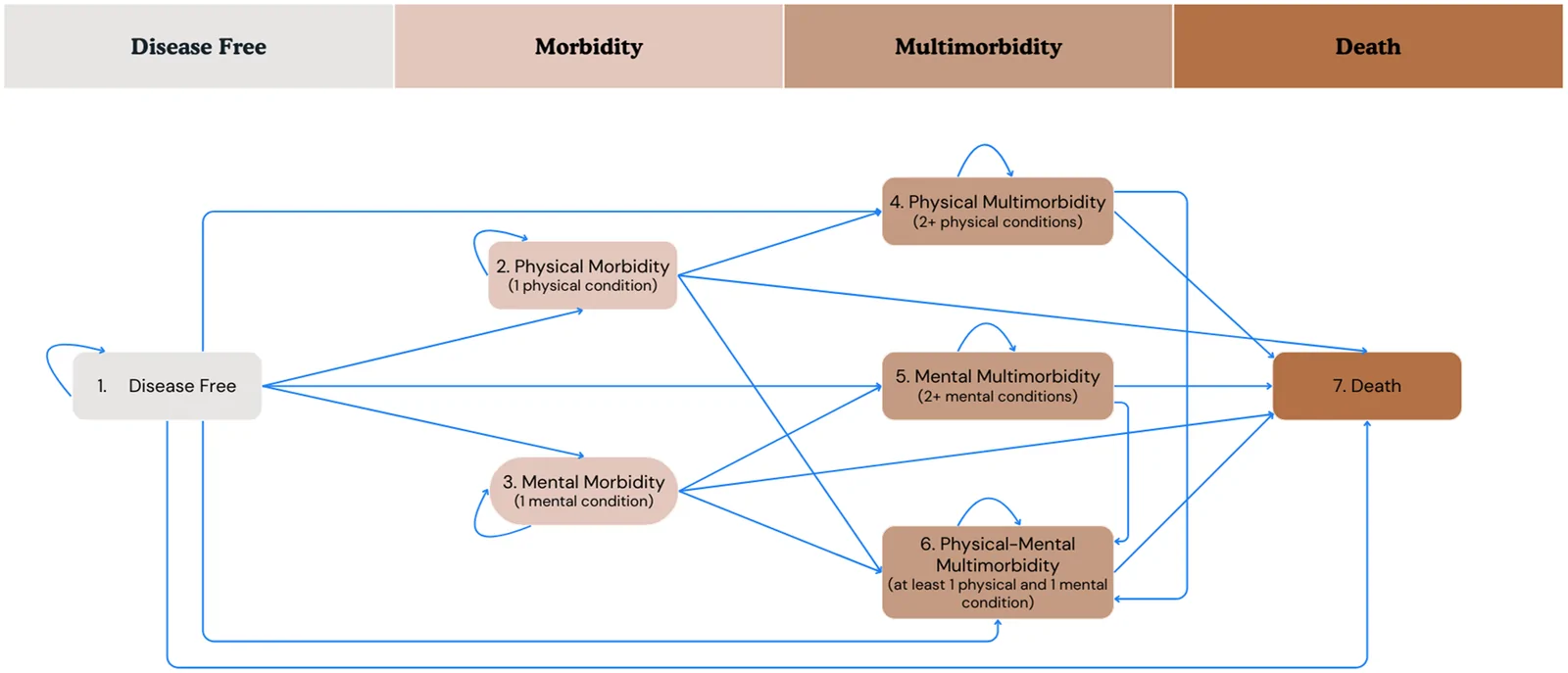
State space.

All individuals entered the model in a disease-free state. Transitions were allowed between disease-free and all other states, between morbidity and multimorbidity states, and from any health state to death. Permitted instantaneous transitions are shown in Fig 1, with direct solid lines and arrows indicating the direction of the transition. The model estimated instantaneous transition probabilities for all allowed forward transitions.

Two key assumptions were applied: (1) once a condition was diagnosed, individuals could not return to the disease-free state; and (2) no more than one transition could occur on the same day. Individuals with two or more diagnoses recorded on the same date could transition directly into a multimorbidity state.

We used time to state or time to death in years as the timescale for transition between different states. Analyses were stratified by age at cohort entry (categorised into 18–24, 25–44, 45–64, and 65+ years) and adjusted for sex, SIMD quintiles, and time-varying age (defined as age at entry into a new state as a continuous variable). All analyses were done in the Health Informatics Centre Trusted Research Environment. Analysis was performed using the msm package [25] in R version 4.1.3.

## Results

### Descriptive statistics

858,789 individuals were included in the analysis. The baseline characteristics of the cohort are presented in Table 1. At the end of the study, 80,948 (9.43%) had single-condition morbidity, and 125,774 (14.65%) had multimorbidity (S2 Table). Of the 80,948 with a single condition, 69,362 (85.69%) had a physical condition and 11,586 (14.31%) had a mental health condition (S3 Table). Of the 125,774 with multimorbidity, 88,891 (70.67%) had physical-only multimorbidity, 2,185 (1.74%) had mental-only multimorbidity, and 34,698 (27.59%) had a combination of physical and mental conditions (S4 Table). Young adults (18–24 years) accounted for 41.56% of individuals with mental-only multimorbidity, while physical-only and physical-mental multimorbidity were more prevalent among those over 45.

**Table 1:** Baseline characteristics of the study population.

Characteristics	All individuals (N, %)
All	858,789 (100)
Sex	
Male	423,206 (49.28)
Female	435,583 (50.72)
Age group (years)	
18-24	339,946 (39.58)
25-44	263,393 (30.67)
45-64	177,600 (20.68)
65+	77,850 (9.07)
Deprivation quintile	
Most deprived SIMD1	117,937 (13.73)
SIMD2	138,167 (16.09)
SIMD3	152,961 (17.81)
SIMD4	208,478 (24.28)
Least deprived SIMD5	180,499 (21.02)
Missing	60,747 (7.07)
Median age (IQR) at start of follow-up (years)	31 (18-48)
Median age (IQR) at end of follow-up (years)	49 (35-65)

Table 2 details demographic and socioeconomic characteristics across all transitions. During the study, 637,428 individuals (74%) remained disease-free and alive. They had a median age of 25 years (IQR: 18–39), a nearly even gender distribution (50.11% female), and lived mostly in less deprived areas. Transitions to disease states followed a temporal logic and were socially stratified. Earlier transitions, such as from disease-free to mental morbidity, occurred at a median age of 35 years (IQR: 20–50) and were slightly male-dominant (55% male), highlighting the burden of mental illness emerging earlier in the life course. In contrast, the median age for transition to physical morbidity was 52 (IQR: 38–64), suggesting a later onset trajectory. Transitions to multimorbidity and death occurred progressively later. The median age for transitions ending in death ranged from 49 (IQR: 33–70) following mental multimorbidity, to 72 years (IQR: 62–78) following physical-mental multimorbidity.

**Table 2:** Summary statistics for each transition.

Transition	Total	Median age at entry (IQR)	Female (N, %)	SIMD1 (N, %)	SIMD2 (N, %)	SIMD3 (N, %)	SIMD4 (N, %)	SIMD5 (N, %)	Missing SIMD (N, %)
Disease-free → Disease-free	637,428	25 (18-39)	319,441 (50.11)	81,133 (12.73)	98,493 (15.45)	110,768 (17.38)	152,975 (24.00)	143,694 (22.54)	50,365 (7.90)
Disease-free → Physical morbidity	133,302	52 (38-64)	71,764 (53.84)	20,551 (15.42)	22,518 (16.89)	25,191 (18.90)	34,332 (25.76)	23,985 (17.99)	6,725 (5.04)
Disease-free → Mental morbidity	18,931	35 (20-50)	8,519 (45.00)	4,596 (24.28)	4,142 (21.88)	3,427 (18.10)	3,582 (18.92)	2,030 (10.72)	1,154 (6.10)
Disease-free → Physical multimorbidity	44,550	62 (52-72)	23,807 (53.44)	7,392 (16.59)	8,460 (18.99)	8,775 (19.70)	11,196 (25.13)	7,210 (16.18)	1,517 (3.41)
Disease-free → Mental multimorbidity	1,304	36 (22-51)	675 (51.76)	281 (21.55)	302 (23.16)	223 (17.10)	285 (21.86)	136 (10.43)	77 (5.90)
Disease-free → Physical-mental multimorbidity	8,635	60 (44-74)	4,795 (55.53)	1,562 (18.09)	1,758 (20.36)	1,671 (19.35)	2,088 (24.18)	1,228 (14.22)	328 (3.80)
Disease-free → Death	14,639	63 (45-77)	6,582 (44.96)	2,422 (16.54)	2,494 (17.04)	2,906 (19.85)	4,020 (27.46)	2,216 (15.14)	581 (3.97)
Physical morbidity → Physical morbidity	61,816	43 (30-54)	33,975 (54.96)	9,053 (14.65)	10,132 (16.39)	11,492 (18.59)	15,596 (25.23)	11,442 (18.51)	4,101 (6.63)
Physical morbidity → Physical multimorbidity	57,204	59 (48-69)	30,074 (52.57)	8,838 (15.45)	9,838 (17.20)	11,064 (19.34)	14,922 (26.09)	10,402 (18.18)	2,140 (3.74)
Physical morbidity → Physical-mental multimorbidity	6,736	58 (39-73)	3,743 (55.57)	1,369 (20.32)	1,257 (18.66)	1,203 (17.86)	1,621 (24.07)	962 (14.28)	324 (4.81)
Physical morbidity → Death	7,546	69 (57-78)	3,972 (52.64)	1,291 (17.11)	1,291 (17.11)	1,432 (18.98)	2,193 (29.06)	1,179 (15.62)	160 (2.12)
Mental morbidity → Mental morbidity	9,951	26 (18-39)	4,225 (42.46)	2,421 (24.33)	2,196 (22.07)	1,757 (17.66)	1,760 (17.69)	1,091 (10.96)	726 (7.29)
Mental morbidity → Mental multimorbidity	1,926	32 (20-45)	966 (50.16)	508 (26.38)	449 (23.31)	361 (18.74)	324 (16.82)	155 (8.05)	129 (6.70)
Mental morbidity → Physical-mental multimorbidity	5,419	48 (34-64)	2,631 (48.55)	1,325 (24.45)	1,196 (22.07)	1,010 (18.64)	1,080 (19.93)	557 (10.28)	251 (4.63)
Mental morbidity → Death	1,635	61 (40-76)	697 (42.63)	342 (20.92)	301 (18.41)	299 (18.29)	418 (25.56)	227 (13.88)	48 (2.94)
Physical multimorbidity → Physical multimorbidity	51,721	54 (43-62)	27,129 (52.45)	7,878 (15.23)	9,055 (17.51)	9,915 (19.17)	12,905 (24.95)	9,518 (18.40)	2,450 (4.74)
Physical multimorbidity → Physical-mental multimorbidity	12,863	69 (59-76)	7,349 (57.13)	2,153 (16.74)	2,344 (18.22)	2,502 (19.45)	3,342 (25.98)	2,042 (15.88)	480 (3.73)
Physical multimorbidity → Death	37,170	68 (59-76)	19,400 (52.19)	6,196 (16.67)	6,897 (18.56)	7,421 (19.96)	9,871 (26.56)	6,051 (16.28)	734 (1.97)
Mental multimorbidity → Mental multimorbidity	1,811	26 (18-37)	879 (48.54)	474 (26.17)	428 (23.63)	336 (18.55)	283 (15.63)	159 (8.78)	131 (7.24)
Mental multimorbidity → Physical-mental multimorbidity	1,045	43 (31-60)	586 (56.08)	242 (23.16)	238 (22.77)	187 (17.89)	231 (22.11)	87 (8.33)	60 (5.74)
Mental multimorbidity → Death	374	49 (33-70)	176 (47.06)	73 (19.52)	85 (22.73)	61 (16.31)	94 (25.13)	45 (12.03)	16 (4.28)
Physical-mental multimorbidity → Physical-mental multimorbidity	15,782	47 (33-61)	8,645 (54.78)	3,450 (21.86)	3,438 (21.78)	2,864 (18.15)	3,152 (19.97)	1,972 (12.50)	906 (5.74)
Physical-mental multimorbidity → Death	18,916	72 (62-78)	10,458 (55.29)	3,201 (16.92)	3,354 (17.73)	3,708 (19.60)	5,210 (27.55)	2,904 (15.35)	539 (2.85)

Women were more represented in transitions involving multimorbidity, particularly those with physical and mental conditions. 57.13% of individuals moving from physical multimorbidity to physical-mental multimorbidity were female, as were 56.08% transitioning from mental multimorbidity to physical-mental multimorbidity. Male were over-represented in transitions into mental morbidity (55%) and from mental morbidity to death (57.37%).

People from the most deprived areas (SIMD1) were over-represented in mental morbidity and in mental-to-physical health transitions. In the transition from disease-free to mental morbidity, 24.28% were from the most deprived areas compared to 10.72% from the least deprived. Similarly, in the transition from mental multimorbidity to physical-mental multimorbidity, the most deprived areas accounted for 23.16% of cases compared to the least deprived with 8.33%. However, physical-to-mental pathways were more evenly distributed between the deprivation quintiles. Those in the least deprived areas were more likely to remain disease-free or stay in the same morbidity state. Conversely, higher proportions of individuals in more deprived areas were observed among those who underwent health transitions, indicating a strong socioeconomic gradient in disease progression.

### Transitioning between health states

#### Transitions from being disease-free.

Fig 2 illustrates estimated transition probabilities between health states over time by age group. Detailed 95% confidence intervals for all transitions are provided in S5 Table. Transitions from disease-free state to physical morbidity were more common than to mental morbidity across all age groups, but there were important age variations. Within five years, the cumulative probability of transitioning from disease-free to physical morbidity (DF → Physical M) was 34% in middle-aged adults (45–64 years) and 45% in older adults (65+ years), compared to just 4% and 12% in young adults (18–24 years) and adults (25–44 years), respectively (Fig 2). However, young adults and adults aged 18–44 years showed higher probabilities of transitioning to mental morbidity from being disease-free, with the cumulative probability exceeding 6% within 10 years (Fig 3). In contrast, middle-aged and older adults aged 45 years and over had less than 1% probability for this transition.

**Fig 2:**
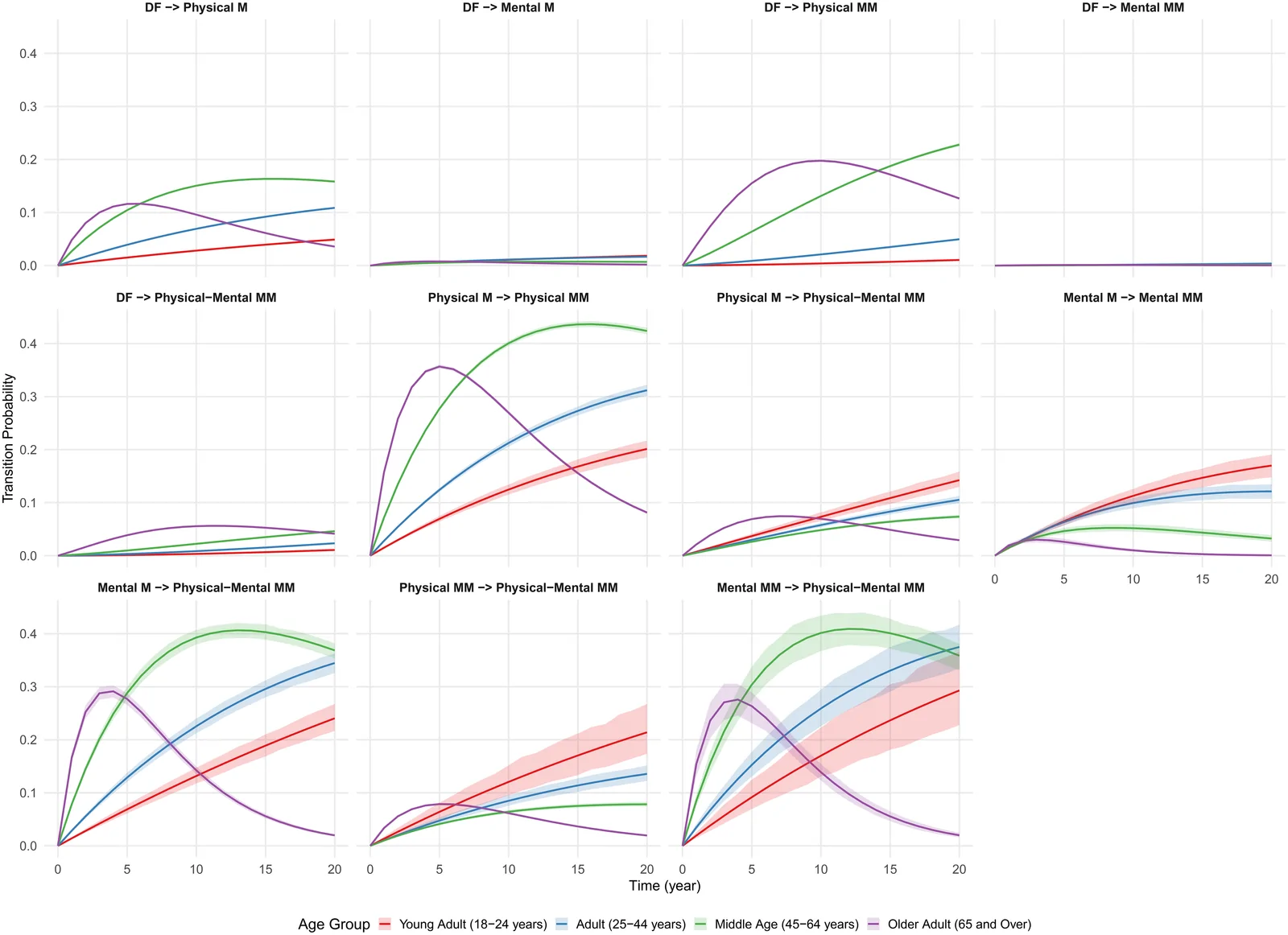
Transition probabilities between health states over time by age group.

**Fig 3:**
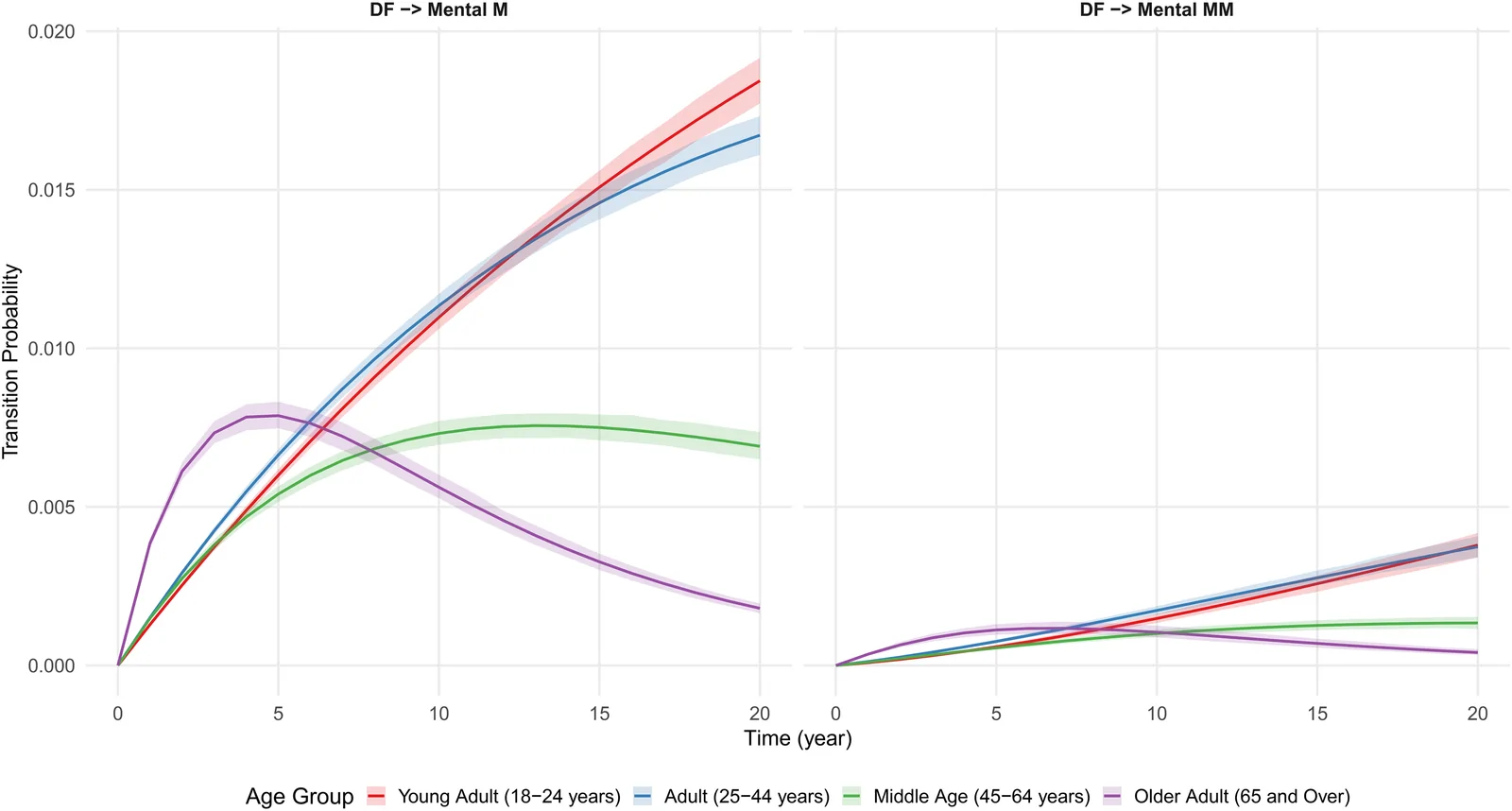
Transition probabilities for DF → Mental Morbidity and DF → Mental Multimorbidity over time by age group.

In people aged 45 years and older, the probability of transitioning directly to physical multimorbidity (DF → Physical MM) exceeded that of transitioning to one physical condition (DF → Physical M), suggesting clusters of physical conditions in later life (Fig 2). Transitions from having no condition straight to multiple mental conditions (DF → Mental MM) were most common in adults aged 18–44 years (Fig 3). Transitions to physical-mental multimorbidity (DF → Physical-Mental MM) occurred across the life course, with gradual increases in older groups (Fig 2). The stability of the disease-free state declined with age. After 5 years, approximately 50% of adults aged 65 years and over remained disease-free, while most people aged 18–24 years maintained this state for over 20 years (S1 Fig).

#### Transitions from morbidity states.

Individuals having one physical condition were more likely to acquire additional physical conditions and progress to physical multimorbidity (Physical M → Physical MM) than to physical-mental multimorbidity (Physical M → Physical-Mental MM) (Fig 2). This progression was more rapid and frequent in people aged 45 years and over compared to adults aged 18–44 years, who showed a slower progression. From physical morbidity, a smaller proportion transitioned to physical-mental multimorbidity, with older adults experiencing this earlier, while other groups showed a steady increase in risk over time.

Individuals with one mental condition followed a distinct pattern. From mental morbidity, young adults had a significantly higher probability of transitioning to mental multimorbidity (Mental M → Mental MM) within five years, exceeding 20%, compared to less than 5% in older adults. The transition probabilities from mental morbidity to physical-mental multimorbidity (Mental M → Physical-Mental MM) were higher than those of physical morbidity (Physical M → Physical-Mental MM) in all age groups, suggesting that mental health issues often precede physical decline and play an important role in pathways into complex physical-mental multimorbidity. This trend is particularly evident in younger adults, where the transition rose more gradually but persisted over time. This indicates extended vulnerability to physical comorbidity following the emergence of mental illness earlier in life.

#### Transitions from multimorbidity states.

Most individuals having multiple physical conditions either remained in that state (Physical MM → Physical MM) or progressed to death (Physical MM → Death) (S1 and S2 Fig). Mortality risk following physical multimorbidity was particularly high among older adults. In contrast, adults aged 18–44 years had a greater probability of having additional mental conditions and transitioning from physical multimorbidity to physical-mental multimorbidity (Physical MM → Physical-Mental MM), suggesting growing vulnerability to mental health issues in this age group (Fig 2).

People with multiple mental conditions were more likely to progress to physical-mental multimorbidity (Mental MM → Physical-Mental MM) than those with multiple physical conditions (Physical MM → Physical-Mental MM), particularly in middle-aged group. Transitions to death from any state increased significantly with age, particularly when both physical and mental conditions were present. Adults aged 18–24 years having mental multimorbidity faced the highest mortality risk, while individuals aged 25 and over had the greatest mortality risk from physical-mental multimorbidity (S3 Fig).

### Trajectory pathways to multimorbidity

We quantified the total pathway probabilities for four distinct routes leading to multimorbidity by multiplying these individual probabilities using a 10-year follow-up time point (Table 3). In 10 years, the trajectory toward physical-only multimorbidity was the most common pathway across all age groups, peaking in middle-aged adults (6.03%). Conversely, mental-only multimorbidity was most prominent among young people. It dropped from 0.12% in young adulthood and 0.11% in adulthood to 0.04% in middle age and 0.01% in older age.

**Table 3:** Conditional probability of multimorbidity pathways by age groups at t = 10 years (%).

Pathway	18-24 years	25-44 years	45-64 years	65 and over
Mental-only multimorbidity				
(DF → Mental M → Mental MM)	0.12	0.11	0.04	0.01
Physical-only multimorbidity				
(DF → Physical M → Physical MM)	0.35	1.47	6.03	2.59
Mental-first pathway				
(DF → Mental M → Physical-mental MM)	0.14	0.26	0.29	0.08
Physical-first pathway				
(DF → Physical M → Physical-mental MM)	0.21	0.40	0.73	0.67

All probabilities expressed as %. Detailed 95% confidence intervals for all transitions are provided in S5 Table.

Regarding complex physical-mental multimorbidity, the physical-first pathway was more probable than the mental-first pathway across all ages. However, this probability ratio narrows significantly in younger cohorts. For young adults, the risk of transitioning to a mental-first complex state is about 70% that of the physical-first route (0.14% vs 0.21%). In older adults, the mental-first route is only approximately 12% as likely (0.08% vs 0.67%). Moreover, the mental-first route showed notable persistence. It started at 0.14% in young adulthood and rose to 0.29% in middle age, suggesting that even as physical diseases increased significantly in mid-life, the mental-first route to complex multimorbidity persisted.

### Effects of sex and area-level deprivation

#### Sex differences.

Fig 4 shows hazard ratios (HRs) and 95% confidence intervals for each transition by sex and area-level deprivation. Females had lower estimated HRs for death across all age groups compared to males. For example, HRs for the transition from disease-free to death (DF → Death) were 0.42 (95% CI: 0.36–0.48) in young women aged 18–24 years, 0.38 (95% CI: 0.35–0.42) in women aged 25–44 years, 0.51 (95% CI: 0.47–0.54) in middle aged women aged 45–64 years and 0.97 (95% CI: 0.92–1.02) in older women aged 65+ years. The mortality risk of women having physical-mental multimorbidity from all age groups was also consistently lower than that of men.

**Fig 4:**
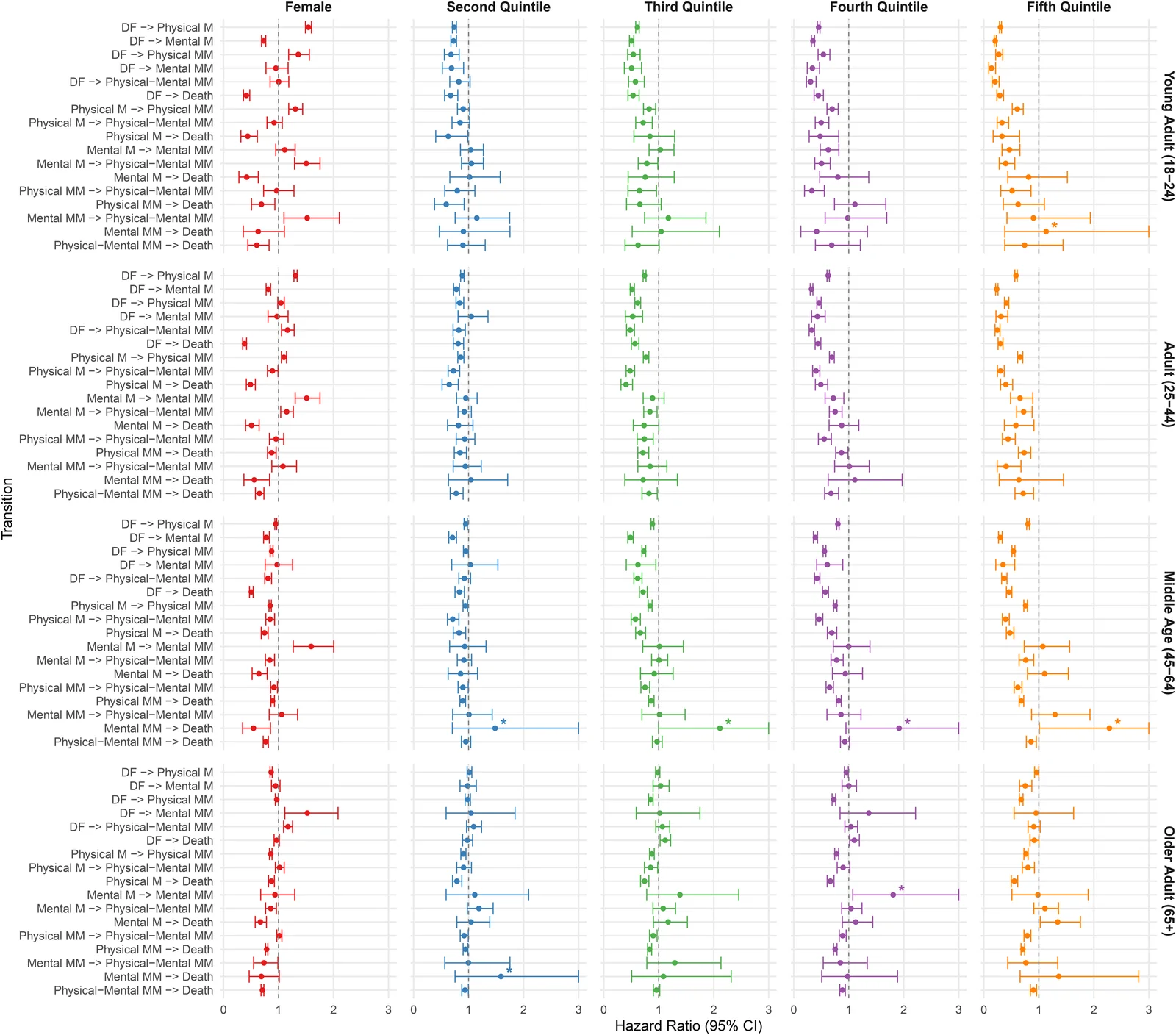
Hazard ratios of moving to each state, with 95% confidence intervals.

On the other hand, we found that women were more likely to accumulate conditions than men. For example, the HR of transitioning from being disease-free to having one physical condition (DF → Physical M) was 1.54 (95% CI: 1.49–1.60) in young adult females. HRs of progressing to further multiple mental conditions from a single mental illness (Mental M → Mental MM) were also higher for adult (HR: 1.52; 95% CI: 1.31–1.77) and middle-aged (HR: 1.61; 95% CI: 1.28–2.02) females compared to males in the same age groups. This suggests that compared to men, women may live longer, but are more likely to develop chronic conditions. Of note, the protective effect of female sex diminishes with age as the relative advantage in mortality transitions and in disease accumulation and progression narrowed among middle aged and older adults.

Nevertheless, an exception was observed in transition of mental morbidity, where young men aged 18−24 years were more likely to have a mental condition from disease-free state (DF → Mental M) compared to women in the same age group. While men were more likely to develop mental morbidity in early adulthood, women had higher risk of progressing to further mental health state (HR: 1.51 [95% CI: 1.30–1.76] in adults aged 25−44 years) and complex mix of physical and mental state (HR: 1.15 [95% CI: 1.04–1.27]). This suggests that men are more likely to experience mental morbidity at a young age and women are more likely to accumulate persistent and complex disease burdens over time, underscoring the differences in health trajectories by sex.

#### Socioeconomic deprivation.

First SIMD quintile (the most deprived areas) was treated as the reference group. Less deprived individuals (second to fifth quintile) had lower estimated HRs across most transitions, especially for transitions to mental illness states. From disease-free state, the HRs of having a mental condition (DF → Mental M) and having multiple mental conditions (DF → Mental MM) were respectively 0.21 (95% CI: 0.19–0.23) and 0.14 (95% CI: 0.09–0.21) in the least deprived young adults. Similar protective effects were observed for physical health and combined multimorbidity transitions. The HRs of progressing to physical-mental multimorbidity from having one physical condition (Physical M → Physical-Mental MM) were significantly lower in less deprived groups (e.g., HR: 0.30; 95% CI: 0.25–0.37 in adults). More socioeconomically advantaged individuals were less likely to progress to physical-mental multimorbidity from mental morbidity, especially among young adults (HR: 0.40; 95% CI: 0.28–0.56). These effects were not limited to the least deprived quintile, indicating that even moderate shifts in socioeconomic status are associated with health improvement. The consistency of protective effects across various transitions suggests a robust social gradient in multimorbidity progression.

The HRs of death-related transitions were lower in less deprived groups. The HRs of mortality from one physical condition (Physical M → Death) and from multiple physical conditions (Physical MM → Death) in the least deprived older adults were 0.55 (95% CI: 0.50–0.62) and 0.71 (95% CI: 0.68–0.74), respectively. The least deprived individuals were also less likely to die from physical-mental multimorbidity than people living in the most deprived areas, with HRs in young adults, adults, middle age, and older adults being 0.74, 0.71, 0.86, and 0.90, respectively. However, the HRs of death from mental multimorbidity were higher among middle-aged adults in the least deprived areas (HR: 2.33; 95% CI: 1.04–5.25), reversing the expected gradient. Confidence intervals were wide in some age groups, suggesting subgroup variability or limited case counts in rarer transitions. Considering the 95% confidence level, random variation may account for some of the confidence intervals that do not include 1. Overall, the findings highlight strong sex and socioeconomic gradients in disease onset, progression, and mortality.

## Discussion

This longitudinal study provides important insights into the dynamic progression of multimorbidity, specifically by depicting the distinct transitional pathways between mental and physical health states. While the cross-sectional association between advancing age and multimorbidity is well established [26–28], traditional static prevalence estimates fail to capture the sequence and directionality of disease accumulation. By applying multi-state modelling to a large, representative cohort, we map the distinct trajectories individuals take from a disease-free baseline, through single-domain morbidities, into complex multimorbid states, and ultimately to mortality.

This temporal approach yields highly specific insights into how chronic disease risk shifts across the life course. It demonstrates that the risk of transitioning from a disease-free state into mental morbidity, and subsequently progressing into mental-only multimorbidity, is disproportionately prominent in young adults (aged 18–44 years). Our longitudinal modelling extends previous literature by revealing a stark mid-life inflection point: after age 45, the probability of transitioning directly from a disease-free state to physical multimorbidity exceeds the probability of acquiring a single physical condition, indicating a rapid, aggressive clustering of chronic physical diseases in mid-to-late life.

We found that the most common trajectory was towards physical-only multimorbidity, peaking at 6.03% in middle-aged adults. Conversely, mental-only multimorbidity was most prominent among young people. Our longitudinal analysis also reveals that while the physical-first pathway is the most frequent route to complex physical-mental multimorbidity across all age groups, the mental-first pathway serves as a critical and persistent gateway. In young adults, the probability of the mental-first pathway (0.14%) is substantial, reaching nearly 70% of the frequency of the physical-first pathway (0.21%), which establishes mental morbidity as a primary driver of incident disease at this stage of life. By middle age, while the physical-first route becomes the dominant driver (0.73% vs. 0.29%), the mental-first pathway continues to operate as a consistent risk factor for complex multi-system decline. This indicates that mental health transitions are a strong, yet less frequent, gateway to complex multimorbidity throughout life and individuals with an incident mental health condition represent a high-risk group that requires proactive, longitudinal monitoring to prevent the subsequent accumulation of physical comorbidities

The diagnostic burden of these multimorbid states varies distinctly by age (S6 Table). Drug/alcohol misuse, anxiety and depression made the top three most common conditions adults aged 18–44 years with physical-mental multimorbidity had. In middle aged adults (45–64 years), the profile shifted toward drug/alcohol misuse, hypertension (treated and untreated) and solid organ cancers. For older adults (65+ years), dementia emerged as the most common condition, followed by hypertension and, persistently, and drug/alcohol misuse.

These findings support previous work [29–31] identifying the critical role of mental health conditions in driving physical health deterioration and vice versa. Our study quantifies this relationship, demonstrating that the impact of mental morbidity is not static, but dynamically accelerates multimorbidity progression throughout adulthood, particularly among socioeconomically disadvantaged groups. Crucially, the consistent presence of subtance and alcohol use disorders as a top-three conditions across all age cohorts having physical-mental multimorbidity, including the elderly, underscores that drug and alcohol misuse remains a pervasive driver of complex multimorbidity and a critical public health challenge in Scotland.

Our findings show that women were less likely to die but consistently had higher risks of developing multiple morbidities. This supports the male-female health-survival paradox [32], and we add further nuance by highlighting how men were more likely to transition to mental morbidity at younger ages, while women accumulated mental health comorbidity and transitioned to physical-mental multimorbidity over time. This reflects both early vulnerability among men and a chronic mental health burden among women. A possible explanation is that men may experience more acute, externally visible mental health crises or substance disorders, while women are more likely to suffer from chronic internalising disorders like anxiety and depression [33–35]. When men are often reluctant to seek help [36,37] which may contribute to sharp early mental morbidity, women are more likely to access services and experience prolonged disease trajectories.

Individuals in less deprived areas had lower transition risks across most pathways, including those involving both mental and physical morbidity. This socioeconomic gradient in multimorbidity aligns with previous findings [27,38], emphasising that deprivation accelerates both the onset and complexity of disease. However, the protective effect of affluence diminished with age, especially in mortality-related transitions. Our work also adds granularity by quantifying how even modest shifts in deprivation can reduce transition risk across multiple disease pathways.

Unexpectedly, our study found a high risk of death from mental multimorbidity in adults aged 45–64 years from the least deprived areas. This challenges the assumptions that affluence always protects against chronic conditions and mortality. While counterintuitive, there are several possible explanations for this finding. First, this could be related to dementia, as it started to emerge as one of the most common conditions among middle aged adults who died having multiple mental conditions (S7 Table). Middle aged people in the least deprived quintile may experience more complex comorbidity due to longer survival, including dementia, potentially contributing to their high mortality risk. People in deprived areas with severe conditions may die earlier or go undiagnosed, while more affluent people may survive long enough to be captured in multimorbidity registries. In addition, better access to healthcare in affluent areas may lead to more frequent formal diagnosis of mental disorders, artificially increasing the apparent mortality risk. Additionally, the severity and duration of mental illness, potential treatment resistance and physiological vulnerability likely outweigh the advantages of socioeconomic status. While this finding requires further investigation, it highlights the need for equitable mental health surveillance across socioeconomic groups.

The study effectively links hospital, psychiatric, and mortality data; and employs multistate models that capture the full longitudinal temporal dynamics of morbidity and mortality over time. The inclusion of a wide range of mental and physical conditions and the use of a validated multimorbidity index add further clinical and methodological robustness. A key strength of this study lies in its exceptionally long observation window, with a maximum follow-up of 21 years. Because the majority of our baseline disease-free cohort was young (resulting in a median age of 49 at the end of follow-up), this extensive timeframe allowed us to capture the critical, early incident trajectories of multimorbidity development. By capturing 21 years of disease trajectories in an initially disease-free, younger cohort, our data specifically highlights the “blind spot” of early-to-mid-life multimorbidity – a phase of the life course that is often overlooked in traditional multimorbidity literature, which predominantly focuses on older populations – and shows that multimorbidity is not just an old person’s issue. However, despite the 21-year follow-up, most of our cohort did not reach the age band over 65 where multimorbidity prevalence traditionally peaks. Therefore, our findings primarily reflect the development of early-onset and mid-life multimorbidity and cannot be generalised to the late-life disease accumulation that this younger cohort will eventually experience.

Our study has some important limitations. Our ascertainment relied primarily on ICD-10 codes from secondary care records. Relying on routinely collected healthcare data may underestimate the prevalence of undiagnosed or unrecorded conditions, particularly for mental health. We were limited to hospital recorded diagnosis for our conditions which is highly likely to have underestimated some of the conditions of interest [39], and may also influence our findings on clustering as conditions accumulated over a period of time will only be recorded at the next hospital visit. Within our baseline cohort, we identified approximately 12.5% of individuals as having one or more conditions, compared to historical Scottish estimates suggesting a population prevalence of roughly 20%. This discrepancy reflects the under-ascertainment of community-managed and primary-care conditions. Consequently, our findings regarding mental health primarily capture severe, hospital-contacting psychiatric conditions (e.g., serious mental illness or acute crises), which frequently present in younger adulthood. This also means our data likely misses reactive depression or anxiety in older adults that develops secondary to a physical diagnosis, as these are typically managed by General Practitioners (GPs). Moreover, this ascertainment bias is particularly more likely to impact women, who are usually present in primary care settings with mild to moderate psychiatric illness and psychological distress and consult their GPs more frequently than men [40,41].

Our inclusion criteria – requiring individuals to be entirely disease-free at baseline and with no diagnoses in the 5 years prior, introduces a further healthy bias to estimates for all age groups – which is likely to be more of an issue for older age groups. Since the likelihood of multimorbidity increases with age, those who survive to age 65 without developing any of the 55 tracked conditions in the previous 5 years represent a resilient demographic. Therefore, our overall transition estimates are likely more representative of population processes for young adults aged 18–44; our older-age transition estimates should be interpreted with care. Our findings emphasise the role of socioeconomic deprivation in accelerating the transition to multimorbidity. However, the true extent of this disparity among older adults is likely obscured by our study design. Since there is a healthier bias in our older cohort, the apparent narrowing of socioeconomic inequalities in our older age groups is probably due to survival bias. Consequently, the impact of SIMD is most accurately reflected in our younger age groups.

Despite the established high diagnostic validity of Scottish routine healthcare data, our reliance on longitudinal diagnosis coding data makes our transition estimates susceptible to diagnostic misclassification. In routine clinical practice, initial diagnoses are often refined as more clinical information becomes available. For example, an initial asthma diagnosis might later be corrected to chronic obstructive pulmonary disease (COPD) after definitive pulmonary function testing. Since our modelling approach tracks the temporal sequence of recorded codes, any diagnostic refinement or correction is interpreted as a disease transition. Therefore, the transition probabilities between clinically adjacent or phenotypically similar conditions, such as asthma to COPD or unipolar depression to bipolar disorder, may be inflated, representing clinical reclassification rather than accumulation of diseases.

Our findings provide significant policy implications, challenging the traditional, siloed approach to chronic disease management. By demonstrating that mental health conditions are important drivers of early-onset multimorbidity, rather than peripheral complications, this study underscores the urgent need for integrated care models that treat physical and mental health concurrently. The findings show clear and age-specific preventative windows: early adulthood represents a critical period to disrupt the psychiatric gateways to complex illness, suggesting that proactive mental health interventions during this phase can yield long-term protective effects. Conversely, midlife emerges as the critical juncture for intercepting the rapid accumulation of physical multimorbidity and its associated mortality risks.

Furthermore, our trajectory analysis highlights clear socioeconomic and demographic disparities. A stark socioeconomic gradient dictates that individuals from deprived areas face earlier and more complex disease trajectories. However, high mortality observed among affluent middle-aged adults with mental multimorbidity challenges the assumption that higher socioeconomic status is universally protective. This nuance highlights the need for equitable, proactive screening strategies across all socioeconomic strata. In addition, demographic differences, specifically the acute emergence of mental morbidity in young men and the morbidity-mortality paradox in women suggest tailored interventions. These could range from rapid early-detection and crisis response services for men, to sustained, integrated chronic care models for women. Ultimately, clinical interventions must be matched by structural public health policies that address upstream determinants like poverty, education, and healthcare access, which act as fundamental levers in multimorbidity progression.

In conclusion, this study provides a comprehensive, dynamic picture of how physical and mental multimorbidity emerge and intersect across the lifespan. By quantifying the persistent influence of mental health on disease trajectories and mapping the compounding effects of age, sex, and socioeconomic deprivation, our results provide a robust evidence base for public health initiatives. To meaningfully mitigate the escalating global burden of multimorbidity and reduce population-level health disparities, public health strategies should move beyond single-disease frameworks to become actively age-sensitive, integrated, trajectory-based, and fundamentally equity-focused.

## Supporting information

S1 Table Chronic conditions included in the study follow-up. (DOCX)

S2 Table Prevalence of chronic morbidity and multimorbidity across age groups. (DOCX)

S3 Table Population distribution of different types of morbidity at the end of the study. (DOCX)

S4 Table Population distribution of different types of multimorbidity at the end of the study. (DOCX)

S5 Table Transition probabilities with 95% confidence interval. (DOCX)

S6 Table Top 5 most common conditions among people across all age groups with physical-mental multimorbidity. (DOCX)

S7 Table Top 5 most common conditions among people across all age groups who died having multiple mental conditions. (DOCX)

S1 Fig Transition probabilities for stable transitions over time by age groups. (PDF)

S2 Fig Probabilities for transitions to death over time by age groups. (PDF)

S3 Fig Survival probabilities over time by age groups. (PDF)
