# Supplementary material for: Longitudinal physical-mental multimorbidity trajectories by sociodemographic factors: a cohort study using electronic health records in Scotland (2000–2021)
Source: PLoS One. 2026 Oct 15;21(10):e0359871. doi: 10.1371/journal.pone.0359871 (PMC13671504; doi:10.1371/journal.pone.0359871)
Supplement: Supplementary file 1 [file pone.0359871.s008.pdf]

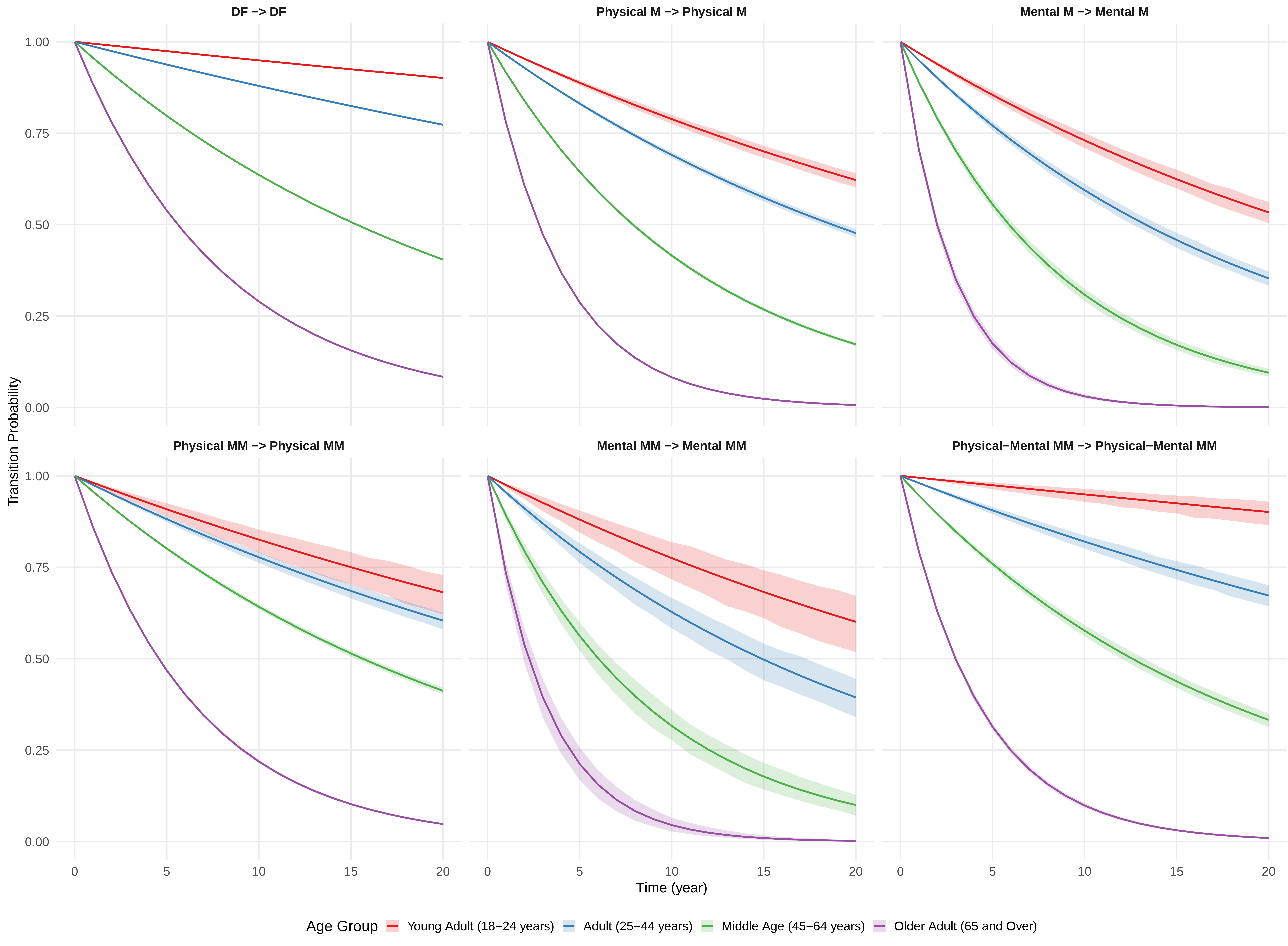
DF −> DF
Physical M −> Physical M
Mental M −> Mental M
Physical MM −> Physical MM
Mental MM −> Mental MM
Physical−Mental MM −> Physical−Mental MM
Transition Probability
1.00
0.75
0.50
0.25
0.00
0
5
10
15
20
Time (year)
Age Group
Young Adult (18−24 years)
Adult (25−44 years)
Middle Age (45−64 years)
Older Adult (65 and Over)
