# Supplementary material for: Longitudinal physical-mental multimorbidity trajectories by sociodemographic factors: a cohort study using electronic health records in Scotland (2000–2021)
Source: PLoS One. 2026 Oct 15;21(10):e0359871. doi: 10.1371/journal.pone.0359871 (PMC13671504; doi:10.1371/journal.pone.0359871)
Supplement: Supplementary file 3 [file pone.0359871.s010.pdf]

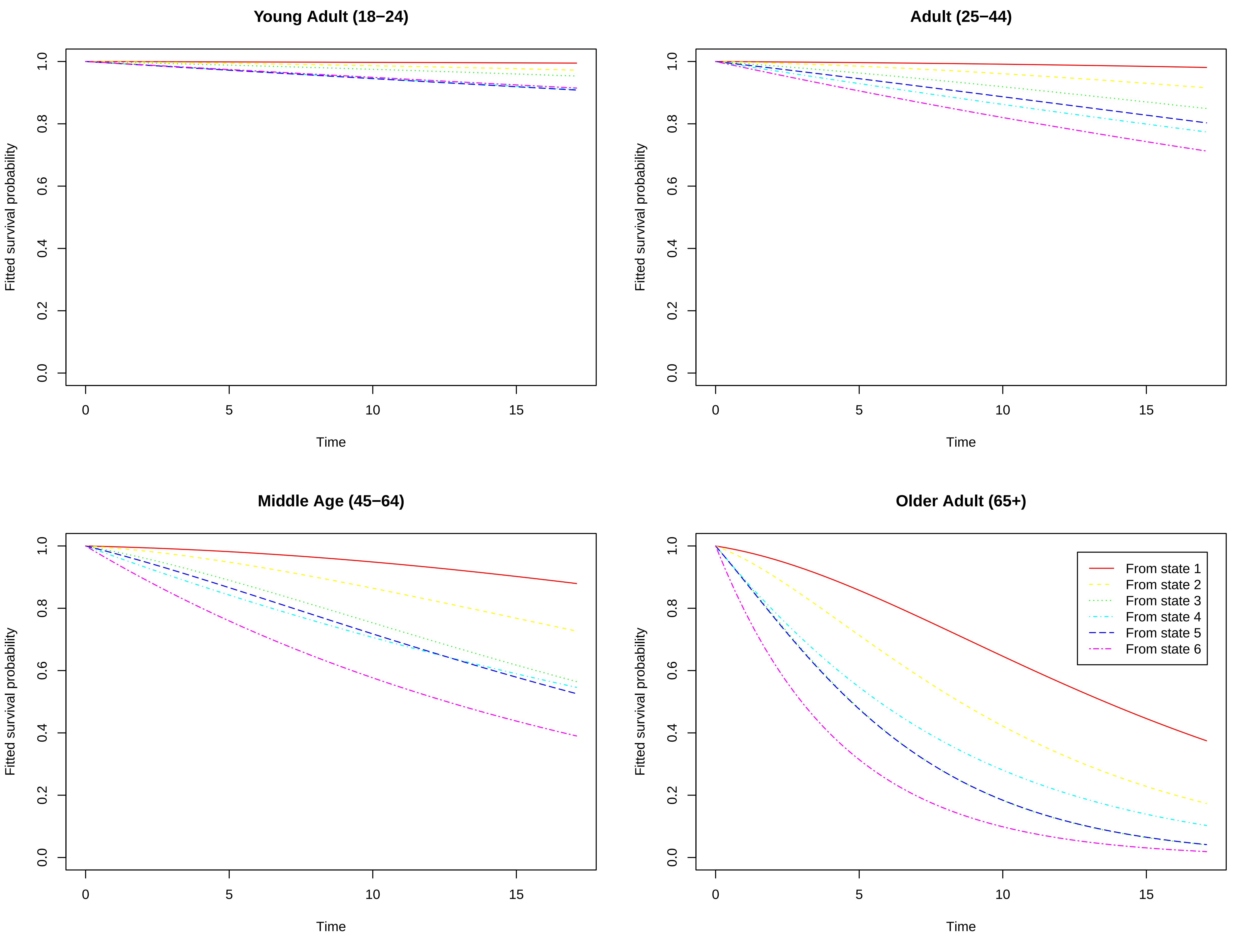

Young Adult (18−24)
Adult (25−44)
Middle Age (45−64)
Older Adult (65+)
Fitted survival probability
Time
0.0
0.2
0.4
0.6
0.8
1.0
0
5
10
15
From state 1
From state 2
From state 3
From state 4
From state 5
From state 6
