# Supplementary material for: Longitudinal physical-mental multimorbidity trajectories by sociodemographic factors: a cohort study using electronic health records in Scotland (2000–2021)
Source: PLoS One. 2026 Oct 15;21(10):e0359871. doi: 10.1371/journal.pone.0359871 (PMC13671504; doi:10.1371/journal.pone.0359871)
Supplement: Supplementary file 4 [file pone.0359871.s001.docx]

**S1 Table. Chronic conditions included in the study follow-up.**

| **Body system** | **Condition** |
| --- | --- |
| Cardiovascular disease | Hypertension (treated and untreated), Coronary Artery Disease, Arrhythmia, Stroke, Heart Failure, Heart Valve Disorder, Peripheral Artery Disease, Venous Thromboembolic disease, Aneurysm |
| Metabolic and endocrine disease | Diabetes, Thyroid disorders, Addison's Disease, Cystic Fibrosis |
| Respiratory disease | Chronic obstructive pulmonary disease (COPD), Asthma, Bronchiectasis |
| Neurological disease | Chronic Primary Pain, Epilepsy, Parkinson's Disease, Paralysis, Multiple Sclerosis |
| Cancer | Solid Organ Cancers, Melanoma, Metastatic Cancer, Haematological Cancer, Benign cerebral tumours that can cause disability |
| Mental and behavioural disorder | Drug/ Alcohol misuse, Dementia, Depression, Anxiety, Schizophrenia, Bipolar disorder, Post-Traumatic Stress Disorder (PTSD), Autism, Eating Disorder |
| Musculoskeletal disease | Osteoarthritis, Long term musculoskeletal problems due to injury, Connective Tissue Disease, Gout |
| Digestive disease | Chronic Liver Disease, Peptic Ulcer, Inflammatory bowel disease (IBD), Chronic Pancreatic Disease |
| Urogenital disorder | Chronic Kidney Disease, Chronic urinary tract infection, Endometriosis, End Stage Kidney Disease |
| Haematological disorder | Anaemia (including pernicious anaemia, sickle cell anaemia) |
| Eye disease | Vision impairment that cannot be corrected |
| Ear disease | Hearing impairment that cannot be corrected, Meniere’s Disease |
| Infectious disease | HIV / AIDS, Tuberculosis, Chronic Lyme disease |
| Congenital disease | Congenital Disease and Chromosomal abnormalities |
