# Supplementary material for: Longitudinal physical-mental multimorbidity trajectories by sociodemographic factors: a cohort study using electronic health records in Scotland (2000–2021)
Source: PLoS One. 2026 Oct 15;21(10):e0359871. doi: 10.1371/journal.pone.0359871 (PMC13671504; doi:10.1371/journal.pone.0359871)
Supplement: Supplementary file 5 [file pone.0359871.s002.docx]

**S2 Table. Prevalence of chronic morbidity and multimorbidity across age groups.**

|  | **Total** | **No condition (N)** | **Morbidity (N, %)** | **Multimorbidity (N, %)** |
| --- | --- | --- | --- | --- |
| 18-24 | 339,946 | 317,279 (93.33) | 16,464 (4.84) | 6,203 (1.82) |
| 25-44 | 263,393 | 215,960 (81.99) | 27,079 (10.28) | 20,354 (7.73) |
| 45-64 | 177,600 | 99,202 (55.86) | 27,838 (15.67) | 50,560 (28.47) |
| 65+ | 77,850 | 19,626 (25.21) | 9,567 (12.29) | 48,657 (62.50) |
| All | 858,789 | 652,067 (75.92) | 80,948 (9.43) | 125,774 (14.65) |
