# Supplementary material for: Longitudinal physical-mental multimorbidity trajectories by sociodemographic factors: a cohort study using electronic health records in Scotland (2000–2021)
Source: PLoS One. 2026 Oct 15;21(10):e0359871. doi: 10.1371/journal.pone.0359871 (PMC13671504; doi:10.1371/journal.pone.0359871)
Supplement: Supplementary file 6 [file pone.0359871.s003.docx]

**S3 Table. Population distribution of different types of morbidity at the end of the study.**

|  | **18-24 (N, %)** | **25-44 (N, %)** | **45-64 (N, %)** | **65+ (N, %)** |
| --- | --- | --- | --- | --- |
| Physical morbidity (n = 69,362) | 11,712 (16.89) | 22,864 (32.96) | 26,161 (37.72) | 8,625 (12.43) |
| Mental morbidity (n = 11,586) | 4,752 (41.02) | 4,215 (36.38) | 1,677 (14.47) | 942 (8.13) |
| All morbidity (n = 80,948) | 16,464 (20.34) | 27,079 (33.45) | 27,838 (34.39) | 9,567 (11.82) |
