# Supplementary material for: Longitudinal physical-mental multimorbidity trajectories by sociodemographic factors: a cohort study using electronic health records in Scotland (2000–2021)
Source: PLoS One. 2026 Oct 15;21(10):e0359871. doi: 10.1371/journal.pone.0359871 (PMC13671504; doi:10.1371/journal.pone.0359871)
Supplement: Supplementary file 7 [file pone.0359871.s004.docx]

**S4 Table. Population distribution of different types of multimorbidity at the end of the study.**

|  | **18-24 (N, %)** | **25-44 (N, %)** | **45-64 (N, %)** | **65+ (N, %)** |
| --- | --- | --- | --- | --- |
| Physical multimorbidity (n = 88,891) | 2,801 (3.15) | 13,463 (15.15) | 40,235 (45.26) | 32,392 (36.44) |
| Mental multimorbidity (n = 2,185) | 908 (41.56) | 842 (38.53) | 281 (12.86) | 154 (7.05) |
| Physical-mental multimorbidity (n = 34,698) | 2,494 (7.19) | 6,049 (17.43) | 10,044 (28.95) | 16,111 (46.43) |
| All multimorbidity (n = 125,774) | 6,203 (4.93) | 20,354 (16.18) | 50,560 (40.20) | 48,657 (38.69) |
