# Supplementary material for: Longitudinal physical-mental multimorbidity trajectories by sociodemographic factors: a cohort study using electronic health records in Scotland (2000–2021)
Source: PLoS One. 2026 Oct 15;21(10):e0359871. doi: 10.1371/journal.pone.0359871 (PMC13671504; doi:10.1371/journal.pone.0359871)
Supplement: Supplementary file 8 [file pone.0359871.s005.docx]

**S5 Table. Transition probabilities with 95% confidence interval.**

| **from** | **to** | **prob** | **lower** | **upper** | **time** | **age group** | **transition** |
| --- | --- | --- | --- | --- | --- | --- | --- |
| State 1 | State 1 | 1 | 1 | 1 | 0 | Young Adult (18-24 years) | DF -> DF |
| State 1 | State 2 | 0 | 0 | 0 | 0 | Young Adult (18-24 years) | DF -> Physical M |
| State 2 | State 2 | 1 | 1 | 1 | 0 | Young Adult (18-24 years) | Physical M -> Physical M |
| State 1 | State 3 | 0 | 0 | 0 | 0 | Young Adult (18-24 years) | DF -> Mental M |
| State 3 | State 3 | 1 | 1 | 1 | 0 | Young Adult (18-24 years) | Mental M -> Mental M |
| State 1 | State 4 | 0 | 0 | 0 | 0 | Young Adult (18-24 years) | DF -> Physical MM |
| State 2 | State 4 | 0 | 0 | 0 | 0 | Young Adult (18-24 years) | Physical M -> Physical MM |
| State 4 | State 4 | 1 | 1 | 1 | 0 | Young Adult (18-24 years) | Physical MM -> Physical MM |
| State 1 | State 5 | 0 | 0 | 0 | 0 | Young Adult (18-24 years) | DF -> Mental MM |
| State 3 | State 5 | 0 | 0 | 0 | 0 | Young Adult (18-24 years) | Mental M -> Mental MM |
| State 5 | State 5 | 1 | 1 | 1 | 0 | Young Adult (18-24 years) | Mental MM -> Mental MM |
| State 1 | State 6 | 0 | 0 | 0 | 0 | Young Adult (18-24 years) | DF -> Physical-Mental MM |
| State 2 | State 6 | 0 | 0 | 0 | 0 | Young Adult (18-24 years) | Physical M -> Physical-Mental MM |
| State 3 | State 6 | 0 | 0 | 0 | 0 | Young Adult (18-24 years) | Mental M -> Physical-Mental MM |
| State 4 | State 6 | 0 | 0 | 0 | 0 | Young Adult (18-24 years) | Physical MM -> Physical-Mental MM |
| State 5 | State 6 | 0 | 0 | 0 | 0 | Young Adult (18-24 years) | Mental MM -> Physical-Mental MM |
| State 6 | State 6 | 1 | 1 | 1 | 0 | Young Adult (18-24 years) | Physical-Mental MM -> Physical-Mental MM |
| State 1 | State 7 | 0 | 0 | 0 | 0 | Young Adult (18-24 years) | DF -> Death |
| State 2 | State 7 | 0 | 0 | 0 | 0 | Young Adult (18-24 years) | Physical M -> Death |
| State 3 | State 7 | 0 | 0 | 0 | 0 | Young Adult (18-24 years) | Mental M -> Death |
| State 4 | State 7 | 0 | 0 | 0 | 0 | Young Adult (18-24 years) | Physical MM -> Death |
| State 5 | State 7 | 0 | 0 | 0 | 0 | Young Adult (18-24 years) | Mental MM -> Death |
| State 6 | State 7 | 0 | 0 | 0 | 0 | Young Adult (18-24 years) | Physical-Mental MM -> Death |
| State 1 | State 1 | 0.99479971 | 0.99472201 | 0.99488085 | 1 | Young Adult (18-24 years) | DF -> DF |
| State 1 | State 2 | 0.00320797 | 0.00314921 | 0.0032643 | 1 | Young Adult (18-24 years) | DF -> Physical M |
| State 2 | State 2 | 0.97653125 | 0.97489109 | 0.9780642 | 1 | Young Adult (18-24 years) | Physical M -> Physical M |
| State 1 | State 3 | 0.00129084 | 0.0012496 | 0.00133368 | 1 | Young Adult (18-24 years) | DF -> Mental M |
| State 3 | State 3 | 0.9690755 | 0.9665596 | 0.97148057 | 1 | Young Adult (18-24 years) | Mental M -> Mental M |
| State 1 | State 4 | 0.00023212 | 0.00021687 | 0.00024791 | 1 | Young Adult (18-24 years) | DF -> Physical MM |
| State 2 | State 4 | 0.01512665 | 0.01396458 | 0.01637197 | 1 | Young Adult (18-24 years) | Physical M -> Physical MM |
| State 4 | State 4 | 0.98102348 | 0.97676225 | 0.98422282 | 1 | Young Adult (18-24 years) | Physical MM -> Physical MM |
| State 1 | State 5 | 8.76E-05 | 7.73E-05 | 9.81E-05 | 1 | Young Adult (18-24 years) | DF -> Mental MM |
| State 3 | State 5 | 0.01457917 | 0.01304897 | 0.01653529 | 1 | Young Adult (18-24 years) | Mental M -> Mental MM |
| State 5 | State 5 | 0.97485288 | 0.96793496 | 0.97982619 | 1 | Young Adult (18-24 years) | Mental MM -> Mental MM |
| State 1 | State 6 | 0.00015152 | 0.00013977 | 0.00016442 | 1 | Young Adult (18-24 years) | DF -> Physical-Mental MM |
| State 2 | State 6 | 0.00742644 | 0.00657115 | 0.00835825 | 1 | Young Adult (18-24 years) | Physical M -> Physical-Mental MM |
| State 3 | State 6 | 0.01421465 | 0.0125052 | 0.01604899 | 1 | Young Adult (18-24 years) | Mental M -> Physical-Mental MM |
| State 4 | State 6 | 0.01344402 | 0.01064679 | 0.01734242 | 1 | Young Adult (18-24 years) | Physical MM -> Physical-Mental MM |
| State 5 | State 6 | 0.01946955 | 0.01489709 | 0.02565832 | 1 | Young Adult (18-24 years) | Mental MM -> Physical-Mental MM |
| State 6 | State 6 | 0.99480865 | 0.99271961 | 0.99622871 | 1 | Young Adult (18-24 years) | Physical-Mental MM -> Physical-Mental MM |
| State 1 | State 7 | 0.00023022 | 0.00021448 | 0.00024759 | 1 | Young Adult (18-24 years) | DF -> Death |
| State 2 | State 7 | 0.00091567 | 0.0006787 | 0.00122805 | 1 | Young Adult (18-24 years) | Physical M -> Death |
| State 3 | State 7 | 0.00213069 | 0.00161477 | 0.00282641 | 1 | Young Adult (18-24 years) | Mental M -> Death |
| State 4 | State 7 | 0.00553249 | 0.00398869 | 0.00784851 | 1 | Young Adult (18-24 years) | Physical MM -> Death |
| State 5 | State 7 | 0.00567756 | 0.00343736 | 0.00921791 | 1 | Young Adult (18-24 years) | Mental MM -> Death |
| State 6 | State 7 | 0.00519135 | 0.00377129 | 0.00728039 | 1 | Young Adult (18-24 years) | Physical-Mental MM -> Death |
| State 1 | State 1 | 0.98962647 | 0.98947424 | 0.98978694 | 2 | Young Adult (18-24 years) | DF -> DF |
| State 1 | State 2 | 0.00632397 | 0.00620761 | 0.00643939 | 2 | Young Adult (18-24 years) | DF -> Physical M |
| State 2 | State 2 | 0.95361328 | 0.95040229 | 0.95662811 | 2 | Young Adult (18-24 years) | Physical M -> Physical M |
| State 1 | State 3 | 0.00253505 | 0.00245516 | 0.00261059 | 2 | Young Adult (18-24 years) | DF -> Mental M |
| State 3 | State 3 | 0.93910732 | 0.93355204 | 0.94430169 | 2 | Young Adult (18-24 years) | Mental M -> Mental M |
| State 1 | State 4 | 0.00050716 | 0.00047486 | 0.00053853 | 2 | Young Adult (18-24 years) | DF -> Physical MM |
| State 2 | State 4 | 0.02961124 | 0.02730139 | 0.03212967 | 2 | Young Adult (18-24 years) | Physical M -> Physical MM |
| State 4 | State 4 | 0.96240708 | 0.95377965 | 0.9687447 | 2 | Young Adult (18-24 years) | Physical MM -> Physical MM |
| State 1 | State 5 | 0.00019138 | 0.00017149 | 0.00021431 | 2 | Young Adult (18-24 years) | DF -> Mental MM |
| State 3 | State 5 | 0.02834085 | 0.02493185 | 0.0318109 | 2 | Young Adult (18-24 years) | Mental M -> Mental MM |
| State 5 | State 5 | 0.95033814 | 0.93630112 | 0.96097779 | 2 | Young Adult (18-24 years) | Mental MM -> Mental MM |
| State 1 | State 6 | 0.00034847 | 0.00032283 | 0.00037725 | 2 | Young Adult (18-24 years) | DF -> Physical-Mental MM |
| State 2 | State 6 | 0.01484339 | 0.01312927 | 0.01666488 | 2 | Young Adult (18-24 years) | Physical M -> Physical-Mental MM |
| State 3 | State 6 | 0.02819977 | 0.02491647 | 0.03217165 | 2 | Young Adult (18-24 years) | Mental M -> Physical-Mental MM |
| State 4 | State 6 | 0.02656313 | 0.02080904 | 0.03409396 | 2 | Young Adult (18-24 years) | Physical MM -> Physical-Mental MM |
| State 5 | State 6 | 0.03834843 | 0.02854274 | 0.0511142 | 2 | Young Adult (18-24 years) | Mental MM -> Physical-Mental MM |
| State 6 | State 6 | 0.98964425 | 0.98526994 | 0.99245037 | 2 | Young Adult (18-24 years) | Physical-Mental MM -> Physical-Mental MM |
| State 1 | State 7 | 0.00046751 | 0.00043661 | 0.000502 | 2 | Young Adult (18-24 years) | DF -> Death |
| State 2 | State 7 | 0.00193209 | 0.00150833 | 0.00253549 | 2 | Young Adult (18-24 years) | Physical M -> Death |
| State 3 | State 7 | 0.00435205 | 0.00328543 | 0.0057751 | 2 | Young Adult (18-24 years) | Mental M -> Death |
| State 4 | State 7 | 0.01102979 | 0.00811917 | 0.01538001 | 2 | Young Adult (18-24 years) | Physical MM -> Death |
| State 5 | State 7 | 0.01131342 | 0.0068508 | 0.01852073 | 2 | Young Adult (18-24 years) | Mental MM -> Death |
| State 6 | State 7 | 0.01035575 | 0.00754963 | 0.01473006 | 2 | Young Adult (18-24 years) | Physical-Mental MM -> Death |
| State 1 | State 1 | 0.98448012 | 0.98424917 | 0.98471541 | 3 | Young Adult (18-24 years) | DF -> DF |
| State 1 | State 2 | 0.00935024 | 0.00917543 | 0.00952632 | 3 | Young Adult (18-24 years) | DF -> Physical M |
| State 2 | State 2 | 0.93123317 | 0.92630649 | 0.93542051 | 3 | Young Adult (18-24 years) | Physical M -> Physical M |
| State 1 | State 3 | 0.0037341 | 0.00361717 | 0.00384984 | 3 | Young Adult (18-24 years) | DF -> Mental M |
| State 3 | State 3 | 0.91006589 | 0.90250721 | 0.91691608 | 3 | Young Adult (18-24 years) | Mental M -> Mental M |
| State 1 | State 4 | 0.00082291 | 0.00077503 | 0.00087159 | 3 | Young Adult (18-24 years) | DF -> Physical MM |
| State 2 | State 4 | 0.04347429 | 0.04030357 | 0.04713235 | 3 | Young Adult (18-24 years) | Physical M -> Physical MM |
| State 4 | State 4 | 0.94414394 | 0.93183936 | 0.95355302 | 3 | Young Adult (18-24 years) | Physical MM -> Physical MM |
| State 1 | State 5 | 0.00031022 | 0.00027928 | 0.00034308 | 3 | Young Adult (18-24 years) | DF -> Mental MM |
| State 3 | State 5 | 0.04131956 | 0.03639891 | 0.04644382 | 3 | Young Adult (18-24 years) | Mental M -> Mental MM |
| State 5 | State 5 | 0.92643988 | 0.90275497 | 0.94252477 | 3 | Young Adult (18-24 years) | Mental MM -> Mental MM |
| State 1 | State 6 | 0.00059016 | 0.00054946 | 0.0006351 | 3 | Young Adult (18-24 years) | DF -> Physical-Mental MM |
| State 2 | State 6 | 0.02224638 | 0.01967426 | 0.02496165 | 3 | Young Adult (18-24 years) | Physical M -> Physical-Mental MM |
| State 3 | State 6 | 0.04195424 | 0.03719525 | 0.04745101 | 3 | Young Adult (18-24 years) | Mental M -> Physical-Mental MM |
| State 4 | State 6 | 0.03936385 | 0.03097787 | 0.04952577 | 3 | Young Adult (18-24 years) | Physical MM -> Physical-Mental MM |
| State 5 | State 6 | 0.05665201 | 0.0422266 | 0.07794041 | 3 | Young Adult (18-24 years) | Mental MM -> Physical-Mental MM |
| State 6 | State 6 | 0.98450666 | 0.97753006 | 0.98909217 | 3 | Young Adult (18-24 years) | Physical-Mental MM -> Physical-Mental MM |
| State 1 | State 7 | 0.00071224 | 0.00066305 | 0.00076893 | 3 | Young Adult (18-24 years) | DF -> Death |
| State 2 | State 7 | 0.00304616 | 0.00242512 | 0.00402338 | 3 | Young Adult (18-24 years) | Physical M -> Death |
| State 3 | State 7 | 0.0066603 | 0.00512702 | 0.00867754 | 3 | Young Adult (18-24 years) | Mental M -> Death |
| State 4 | State 7 | 0.0164922 | 0.01183271 | 0.02301145 | 3 | Young Adult (18-24 years) | Physical MM -> Death |
| State 5 | State 7 | 0.01690811 | 0.0102642 | 0.02872986 | 3 | Young Adult (18-24 years) | Mental MM -> Death |
| State 6 | State 7 | 0.01549334 | 0.01090783 | 0.02246994 | 3 | Young Adult (18-24 years) | Physical-Mental MM -> Death |
| State 1 | State 1 | 0.97936054 | 0.9790714 | 0.9796485 | 4 | Young Adult (18-24 years) | DF -> DF |
| State 1 | State 2 | 0.01228899 | 0.01205843 | 0.01251655 | 4 | Young Adult (18-24 years) | DF -> Physical M |
| State 2 | State 2 | 0.90937829 | 0.90350084 | 0.91497145 | 4 | Young Adult (18-24 years) | Physical M -> Physical M |
| State 1 | State 3 | 0.00488944 | 0.00473745 | 0.00503255 | 4 | Young Adult (18-24 years) | DF -> Mental M |
| State 3 | State 3 | 0.88192256 | 0.87088258 | 0.89152674 | 4 | Young Adult (18-24 years) | Mental M -> Mental M |
| State 1 | State 4 | 0.00117726 | 0.00111342 | 0.00125179 | 4 | Young Adult (18-24 years) | DF -> Physical MM |
| State 2 | State 4 | 0.05673573 | 0.05267452 | 0.06142464 | 4 | Young Adult (18-24 years) | Physical M -> Physical MM |
| State 4 | State 4 | 0.92622738 | 0.91102123 | 0.93837912 | 4 | Young Adult (18-24 years) | Physical MM -> Physical MM |
| State 1 | State 5 | 0.00044311 | 0.0004005 | 0.00048484 | 4 | Young Adult (18-24 years) | DF -> Mental MM |
| State 3 | State 5 | 0.0535485 | 0.04731568 | 0.06031215 | 4 | Young Adult (18-24 years) | Mental M -> Mental MM |
| State 5 | State 5 | 0.90314259 | 0.87595583 | 0.92292981 | 4 | Young Adult (18-24 years) | Mental MM -> Mental MM |
| State 1 | State 6 | 0.00087589 | 0.00081828 | 0.00094328 | 4 | Young Adult (18-24 years) | DF -> Physical-Mental MM |
| State 2 | State 6 | 0.0296311 | 0.02620526 | 0.03332149 | 4 | Young Adult (18-24 years) | Physical M -> Physical-Mental MM |
| State 3 | State 6 | 0.05547719 | 0.0494248 | 0.06288397 | 4 | Young Adult (18-24 years) | Mental M -> Physical-Mental MM |
| State 4 | State 6 | 0.05185259 | 0.04041079 | 0.06600668 | 4 | Young Adult (18-24 years) | Physical MM -> Physical-Mental MM |
| State 5 | State 6 | 0.07439529 | 0.05624508 | 0.09858738 | 4 | Young Adult (18-24 years) | Mental MM -> Physical-Mental MM |
| State 6 | State 6 | 0.97939575 | 0.97074266 | 0.98544398 | 4 | Young Adult (18-24 years) | Physical-Mental MM -> Physical-Mental MM |
| State 1 | State 7 | 0.00096478 | 0.000902 | 0.0010439 | 4 | Young Adult (18-24 years) | DF -> Death |
| State 2 | State 7 | 0.00425488 | 0.00333013 | 0.00553635 | 4 | Young Adult (18-24 years) | Physical M -> Death |
| State 3 | State 7 | 0.00905176 | 0.00687736 | 0.01183883 | 4 | Young Adult (18-24 years) | Mental M -> Death |
| State 4 | State 7 | 0.02192003 | 0.0162991 | 0.03014992 | 4 | Young Adult (18-24 years) | Physical MM -> Death |
| State 5 | State 7 | 0.02246213 | 0.01382986 | 0.03854196 | 4 | Young Adult (18-24 years) | Mental MM -> Death |
| State 6 | State 7 | 0.02060425 | 0.01455602 | 0.02925734 | 4 | Young Adult (18-24 years) | Physical-Mental MM -> Death |
| State 1 | State 1 | 0.97426758 | 0.97385977 | 0.97465812 | 5 | Young Adult (18-24 years) | DF -> DF |
| State 1 | State 2 | 0.01514234 | 0.0148545 | 0.01544967 | 5 | Young Adult (18-24 years) | DF -> Physical M |
| State 2 | State 2 | 0.88803632 | 0.88096015 | 0.89419493 | 5 | Young Adult (18-24 years) | Physical M -> Physical M |
| State 1 | State 3 | 0.00600243 | 0.00581941 | 0.00619052 | 5 | Young Adult (18-24 years) | DF -> Mental M |
| State 3 | State 3 | 0.85464954 | 0.84268485 | 0.86630917 | 5 | Young Adult (18-24 years) | Mental M -> Mental M |
| State 1 | State 4 | 0.00156814 | 0.00148767 | 0.00166289 | 5 | Young Adult (18-24 years) | DF -> Physical MM |
| State 2 | State 4 | 0.06941493 | 0.06462424 | 0.07480195 | 5 | Young Adult (18-24 years) | Physical M -> Physical MM |
| State 4 | State 4 | 0.90865081 | 0.88920901 | 0.92525991 | 5 | Young Adult (18-24 years) | Physical MM -> Physical MM |
| State 1 | State 5 | 0.00058905 | 0.00053342 | 0.00064766 | 5 | Young Adult (18-24 years) | DF -> Mental MM |
| State 3 | State 5 | 0.0650596 | 0.05702261 | 0.07371898 | 5 | Young Adult (18-24 years) | Mental M -> Mental MM |
| State 5 | State 5 | 0.88043116 | 0.8451969 | 0.90486817 | 5 | Young Adult (18-24 years) | Mental MM -> Mental MM |
| State 1 | State 6 | 0.00120496 | 0.00113536 | 0.00128287 | 5 | Young Adult (18-24 years) | DF -> Physical-Mental MM |
| State 2 | State 6 | 0.03699347 | 0.03294292 | 0.04128376 | 5 | Young Adult (18-24 years) | Physical M -> Physical-Mental MM |
| State 3 | State 6 | 0.06876797 | 0.06133319 | 0.07749811 | 5 | Young Adult (18-24 years) | Mental M -> Physical-Mental MM |
| State 4 | State 6 | 0.06403563 | 0.04919361 | 0.08122271 | 5 | Young Adult (18-24 years) | Physical MM -> Physical-Mental MM |
| State 5 | State 6 | 0.09159286 | 0.069427 | 0.12477791 | 5 | Young Adult (18-24 years) | Mental MM -> Physical-Mental MM |
| State 6 | State 6 | 0.97431136 | 0.96350587 | 0.98232402 | 5 | Young Adult (18-24 years) | Physical-Mental MM -> Physical-Mental MM |
| State 1 | State 7 | 0.0012255 | 0.0011468 | 0.00131424 | 5 | Young Adult (18-24 years) | DF -> Death |
| State 2 | State 7 | 0.00555528 | 0.00436042 | 0.00721196 | 5 | Young Adult (18-24 years) | Physical M -> Death |
| State 3 | State 7 | 0.01152289 | 0.00885454 | 0.01525737 | 5 | Young Adult (18-24 years) | Mental M -> Death |
| State 4 | State 7 | 0.02731356 | 0.02043599 | 0.03804698 | 5 | Young Adult (18-24 years) | Physical MM -> Death |
| State 5 | State 7 | 0.02797599 | 0.01714609 | 0.04673521 | 5 | Young Adult (18-24 years) | Mental MM -> Death |
| State 6 | State 7 | 0.02568864 | 0.01767598 | 0.03649413 | 5 | Young Adult (18-24 years) | Physical-Mental MM -> Death |
| State 1 | State 1 | 0.96920111 | 0.96872432 | 0.96967608 | 6 | Young Adult (18-24 years) | DF -> DF |
| State 1 | State 2 | 0.01791238 | 0.01754964 | 0.01826077 | 6 | Young Adult (18-24 years) | DF -> Physical M |
| State 2 | State 2 | 0.86719522 | 0.8587404 | 0.87493778 | 6 | Young Adult (18-24 years) | Physical M -> Physical M |
| State 1 | State 3 | 0.00707443 | 0.00685768 | 0.00729905 | 6 | Young Adult (18-24 years) | DF -> Mental M |
| State 3 | State 3 | 0.82821993 | 0.81435061 | 0.84035503 | 6 | Young Adult (18-24 years) | Mental M -> Mental M |
| State 1 | State 4 | 0.00199359 | 0.00187942 | 0.00209759 | 6 | Young Adult (18-24 years) | DF -> Physical MM |
| State 2 | State 4 | 0.08153069 | 0.07558888 | 0.08764503 | 6 | Young Adult (18-24 years) | Physical M -> Physical MM |
| State 4 | State 4 | 0.89140779 | 0.86908317 | 0.91084967 | 6 | Young Adult (18-24 years) | Physical MM -> Physical MM |
| State 1 | State 5 | 0.0007471 | 0.00067707 | 0.00082006 | 6 | Young Adult (18-24 years) | DF -> Mental MM |
| State 3 | State 5 | 0.07588362 | 0.06750488 | 0.08546832 | 6 | Young Adult (18-24 years) | Mental M -> Mental MM |
| State 5 | State 5 | 0.85829085 | 0.81807049 | 0.88762996 | 6 | Young Adult (18-24 years) | Mental MM -> Mental MM |
| State 1 | State 6 | 0.00157665 | 0.0014882 | 0.00168577 | 6 | Young Adult (18-24 years) | DF -> Physical-Mental MM |
| State 2 | State 6 | 0.04432958 | 0.03961703 | 0.05015489 | 6 | Young Adult (18-24 years) | Physical M -> Physical-Mental MM |
| State 3 | State 6 | 0.0818262 | 0.0728739 | 0.09230445 | 6 | Young Adult (18-24 years) | Mental M -> Physical-Mental MM |
| State 4 | State 6 | 0.07591912 | 0.0602239 | 0.0954615 | 6 | Young Adult (18-24 years) | Physical MM -> Physical-Mental MM |
| State 5 | State 6 | 0.10825897 | 0.08220112 | 0.1424237 | 6 | Young Adult (18-24 years) | Mental MM -> Physical-Mental MM |
| State 6 | State 6 | 0.96925337 | 0.95603757 | 0.97789396 | 6 | Young Adult (18-24 years) | Physical-Mental MM -> Physical-Mental MM |
| State 1 | State 7 | 0.00149473 | 0.00139962 | 0.00159902 | 6 | Young Adult (18-24 years) | DF -> Death |
| State 2 | State 7 | 0.00694451 | 0.00552775 | 0.00874562 | 6 | Young Adult (18-24 years) | Physical M -> Death |
| State 3 | State 7 | 0.01407026 | 0.01117371 | 0.01830441 | 6 | Young Adult (18-24 years) | Mental M -> Death |
| State 4 | State 7 | 0.0326731 | 0.02376999 | 0.04422296 | 6 | Young Adult (18-24 years) | Physical MM -> Death |
| State 5 | State 7 | 0.03345018 | 0.02060435 | 0.05305633 | 6 | Young Adult (18-24 years) | Mental MM -> Death |
| State 6 | State 7 | 0.03074663 | 0.02210604 | 0.04396243 | 6 | Young Adult (18-24 years) | Physical-Mental MM -> Death |
| State 1 | State 1 | 0.96416098 | 0.96358337 | 0.9646958 | 7 | Young Adult (18-24 years) | DF -> DF |
| State 1 | State 2 | 0.02060117 | 0.02019963 | 0.02101023 | 7 | Young Adult (18-24 years) | DF -> Physical M |
| State 2 | State 2 | 0.84684323 | 0.83699791 | 0.85514873 | 7 | Young Adult (18-24 years) | Physical M -> Physical M |
| State 1 | State 3 | 0.00810674 | 0.00784064 | 0.00838674 | 7 | Young Adult (18-24 years) | DF -> Mental M |
| State 3 | State 3 | 0.80260764 | 0.78621323 | 0.81670824 | 7 | Young Adult (18-24 years) | Mental M -> Mental M |
| State 1 | State 4 | 0.00245168 | 0.00231699 | 0.00258674 | 7 | Young Adult (18-24 years) | DF -> Physical MM |
| State 2 | State 4 | 0.09310128 | 0.08647582 | 0.10021591 | 7 | Young Adult (18-24 years) | Physical M -> Physical MM |
| State 4 | State 4 | 0.87449197 | 0.84712308 | 0.89684899 | 7 | Young Adult (18-24 years) | Physical MM -> Physical MM |
| State 1 | State 5 | 0.00091636 | 0.00083952 | 0.00100617 | 7 | Young Adult (18-24 years) | DF -> Mental MM |
| State 3 | State 5 | 0.08605012 | 0.07592787 | 0.09787454 | 7 | Young Adult (18-24 years) | Mental M -> Mental MM |
| State 5 | State 5 | 0.83670731 | 0.79438027 | 0.86978846 | 7 | Young Adult (18-24 years) | Mental MM -> Mental MM |
| State 1 | State 6 | 0.00199026 | 0.00186794 | 0.0021236 | 7 | Young Adult (18-24 years) | DF -> Physical-Mental MM |
| State 2 | State 6 | 0.05163572 | 0.04601202 | 0.05776548 | 7 | Young Adult (18-24 years) | Physical M -> Physical-Mental MM |
| State 3 | State 6 | 0.09465168 | 0.083963 | 0.10566721 | 7 | Young Adult (18-24 years) | Mental M -> Physical-Mental MM |
| State 4 | State 6 | 0.0875091 | 0.0687094 | 0.11063974 | 7 | Young Adult (18-24 years) | Physical MM -> Physical-Mental MM |
| State 5 | State 6 | 0.1244075 | 0.09336934 | 0.1628703 | 7 | Young Adult (18-24 years) | Mental MM -> Physical-Mental MM |
| State 6 | State 6 | 0.96422164 | 0.94974613 | 0.97398172 | 7 | Young Adult (18-24 years) | Physical-Mental MM -> Physical-Mental MM |
| State 1 | State 7 | 0.0017728 | 0.00165322 | 0.00189223 | 7 | Young Adult (18-24 years) | DF -> Death |
| State 2 | State 7 | 0.00841977 | 0.00681072 | 0.01053667 | 7 | Young Adult (18-24 years) | Physical M -> Death |
| State 3 | State 7 | 0.01669056 | 0.01284186 | 0.0216918 | 7 | Young Adult (18-24 years) | Mental M -> Death |
| State 4 | State 7 | 0.03799893 | 0.02804243 | 0.05159998 | 7 | Young Adult (18-24 years) | Physical MM -> Death |
| State 5 | State 7 | 0.03888519 | 0.02397186 | 0.06290527 | 7 | Young Adult (18-24 years) | Mental MM -> Death |
| State 6 | State 7 | 0.03577836 | 0.02601828 | 0.05025387 | 7 | Young Adult (18-24 years) | Physical-Mental MM -> Death |
| State 1 | State 1 | 0.95914707 | 0.95854529 | 0.95973279 | 8 | Young Adult (18-24 years) | DF -> DF |
| State 1 | State 2 | 0.02321068 | 0.02279745 | 0.02363672 | 8 | Young Adult (18-24 years) | DF -> Physical M |
| State 2 | State 2 | 0.82696887 | 0.8155396 | 0.83771512 | 8 | Young Adult (18-24 years) | Physical M -> Physical M |
| State 1 | State 3 | 0.00910062 | 0.00880801 | 0.00939288 | 8 | Young Adult (18-24 years) | DF -> Mental M |
| State 3 | State 3 | 0.7777874 | 0.76010732 | 0.79325064 | 8 | Young Adult (18-24 years) | Mental M -> Mental M |
| State 1 | State 4 | 0.00294059 | 0.00276875 | 0.00310979 | 8 | Young Adult (18-24 years) | DF -> Physical MM |
| State 2 | State 4 | 0.10414444 | 0.09582014 | 0.11244669 | 8 | Young Adult (18-24 years) | Physical M -> Physical MM |
| State 4 | State 4 | 0.85789716 | 0.82653535 | 0.88098984 | 8 | Young Adult (18-24 years) | Physical MM -> Physical MM |
| State 1 | State 5 | 0.00109598 | 0.0009914 | 0.00120612 | 8 | Young Adult (18-24 years) | DF -> Mental MM |
| State 3 | State 5 | 0.09558756 | 0.08424443 | 0.10767875 | 8 | Young Adult (18-24 years) | Mental M -> Mental MM |
| State 5 | State 5 | 0.81566653 | 0.7648077 | 0.85314058 | 8 | Young Adult (18-24 years) | Mental MM -> Mental MM |
| State 1 | State 6 | 0.00244505 | 0.00231002 | 0.0026052 | 8 | Young Adult (18-24 years) | DF -> Physical-Mental MM |
| State 2 | State 6 | 0.05890835 | 0.05292495 | 0.06575185 | 8 | Young Adult (18-24 years) | Physical M -> Physical-Mental MM |
| State 3 | State 6 | 0.10724446 | 0.0956143 | 0.1201334 | 8 | Young Adult (18-24 years) | Mental M -> Physical-Mental MM |
| State 4 | State 6 | 0.0988115 | 0.07742859 | 0.12388518 | 8 | Young Adult (18-24 years) | Physical MM -> Physical-Mental MM |
| State 5 | State 6 | 0.14005198 | 0.10517832 | 0.18584756 | 8 | Young Adult (18-24 years) | Mental MM -> Physical-Mental MM |
| State 6 | State 6 | 0.95921603 | 0.9419053 | 0.97138525 | 8 | Young Adult (18-24 years) | Physical-Mental MM -> Physical-Mental MM |
| State 1 | State 7 | 0.00206 | 0.00193022 | 0.00221329 | 8 | Young Adult (18-24 years) | DF -> Death |
| State 2 | State 7 | 0.00997834 | 0.00796265 | 0.01261934 | 8 | Young Adult (18-24 years) | Physical M -> Death |
| State 3 | State 7 | 0.01938059 | 0.01541347 | 0.02505317 | 8 | Young Adult (18-24 years) | Mental M -> Death |
| State 4 | State 7 | 0.04329134 | 0.03147497 | 0.05976116 | 8 | Young Adult (18-24 years) | Physical MM -> Death |
| State 5 | State 7 | 0.04428149 | 0.02815283 | 0.07390545 | 8 | Young Adult (18-24 years) | Mental MM -> Death |
| State 6 | State 7 | 0.04078397 | 0.02861475 | 0.0580947 | 8 | Young Adult (18-24 years) | Physical-Mental MM -> Death |
| State 1 | State 1 | 0.95415923 | 0.95350194 | 0.95485331 | 9 | Young Adult (18-24 years) | DF -> DF |
| State 1 | State 2 | 0.02574287 | 0.0252392 | 0.02629526 | 9 | Young Adult (18-24 years) | DF -> Physical M |
| State 2 | State 2 | 0.80756095 | 0.79575806 | 0.81827404 | 9 | Young Adult (18-24 years) | Physical M -> Physical M |
| State 1 | State 3 | 0.0100573 | 0.00972884 | 0.01038173 | 9 | Young Adult (18-24 years) | DF -> Mental M |
| State 3 | State 3 | 0.75373471 | 0.73467031 | 0.77214774 | 9 | Young Adult (18-24 years) | Mental M -> Mental M |
| State 1 | State 4 | 0.00345853 | 0.0032622 | 0.00363875 | 9 | Young Adult (18-24 years) | DF -> Physical MM |
| State 2 | State 4 | 0.1146774 | 0.10590825 | 0.12387113 | 9 | Young Adult (18-24 years) | Physical M -> Physical MM |
| State 4 | State 4 | 0.84161727 | 0.8118407 | 0.86812744 | 9 | Young Adult (18-24 years) | Physical MM -> Physical MM |
| State 1 | State 5 | 0.00128513 | 0.0011744 | 0.00141231 | 9 | Young Adult (18-24 years) | DF -> Mental MM |
| State 3 | State 5 | 0.1045233 | 0.09279436 | 0.11721533 | 9 | Young Adult (18-24 years) | Mental M -> Mental MM |
| State 5 | State 5 | 0.79515487 | 0.74162149 | 0.83571602 | 9 | Young Adult (18-24 years) | Mental MM -> Mental MM |
| State 1 | State 6 | 0.00294029 | 0.00275253 | 0.00314784 | 9 | Young Adult (18-24 years) | DF -> Physical-Mental MM |
| State 2 | State 6 | 0.06614408 | 0.05964247 | 0.07380128 | 9 | Young Adult (18-24 years) | Physical M -> Physical-Mental MM |
| State 3 | State 6 | 0.11960474 | 0.10657908 | 0.13370611 | 9 | Young Adult (18-24 years) | Mental M -> Physical-Mental MM |
| State 4 | State 6 | 0.10983212 | 0.08641538 | 0.13565001 | 9 | Young Adult (18-24 years) | Physical MM -> Physical-Mental MM |
| State 5 | State 6 | 0.15520558 | 0.11524401 | 0.20387379 | 9 | Young Adult (18-24 years) | Mental MM -> Physical-Mental MM |
| State 6 | State 6 | 0.95423641 | 0.93581328 | 0.96680776 | 9 | Young Adult (18-24 years) | Physical-Mental MM -> Physical-Mental MM |
| State 1 | State 7 | 0.00235665 | 0.00220651 | 0.00253308 | 9 | Young Adult (18-24 years) | DF -> Death |
| State 2 | State 7 | 0.01161757 | 0.00962927 | 0.01453187 | 9 | Young Adult (18-24 years) | Physical M -> Death |
| State 3 | State 7 | 0.02213726 | 0.01782676 | 0.02832697 | 9 | Young Adult (18-24 years) | Mental M -> Death |
| State 4 | State 7 | 0.04855062 | 0.03599679 | 0.06588349 | 9 | Young Adult (18-24 years) | Physical MM -> Death |
| State 5 | State 7 | 0.04963954 | 0.03184419 | 0.08027878 | 9 | Young Adult (18-24 years) | Mental MM -> Death |
| State 6 | State 7 | 0.04576359 | 0.03319224 | 0.06418672 | 9 | Young Adult (18-24 years) | Physical-Mental MM -> Death |
| State 1 | State 1 | 0.94919732 | 0.94845243 | 0.94988122 | 10 | Young Adult (18-24 years) | DF -> DF |
| State 1 | State 2 | 0.02819963 | 0.02764802 | 0.02875268 | 10 | Young Adult (18-24 years) | DF -> Physical M |
| State 2 | State 2 | 0.7886085 | 0.77578983 | 0.80015982 | 10 | Young Adult (18-24 years) | Physical M -> Physical M |
| State 1 | State 3 | 0.01097795 | 0.01061698 | 0.01132236 | 10 | Young Adult (18-24 years) | DF -> Mental M |
| State 3 | State 3 | 0.73042584 | 0.7093478 | 0.74968833 | 10 | Young Adult (18-24 years) | Mental M -> Mental M |
| State 1 | State 4 | 0.00400379 | 0.00378173 | 0.00423226 | 10 | Young Adult (18-24 years) | DF -> Physical MM |
| State 2 | State 4 | 0.12471692 | 0.1154956 | 0.13412935 | 10 | Young Adult (18-24 years) | Physical M -> Physical MM |
| State 4 | State 4 | 0.8256463 | 0.78987477 | 0.85285939 | 10 | Young Adult (18-24 years) | Physical MM -> Physical MM |
| State 1 | State 5 | 0.00148303 | 0.00134063 | 0.00162392 | 10 | Young Adult (18-24 years) | DF -> Mental MM |
| State 3 | State 5 | 0.11288366 | 0.09903832 | 0.1257583 | 10 | Young Adult (18-24 years) | Mental M -> Mental MM |
| State 5 | State 5 | 0.77515902 | 0.71666015 | 0.81856156 | 10 | Young Adult (18-24 years) | Mental MM -> Mental MM |
| State 1 | State 6 | 0.00347526 | 0.00328296 | 0.0037059 | 10 | Young Adult (18-24 years) | DF -> Physical-Mental MM |
| State 2 | State 6 | 0.07333973 | 0.06552462 | 0.08180511 | 10 | Young Adult (18-24 years) | Physical M -> Physical-Mental MM |
| State 3 | State 6 | 0.13173292 | 0.11769972 | 0.14727712 | 10 | Young Adult (18-24 years) | Mental M -> Physical-Mental MM |
| State 4 | State 6 | 0.12057666 | 0.09523959 | 0.15195385 | 10 | Young Adult (18-24 years) | Physical MM -> Physical-Mental MM |
| State 5 | State 6 | 0.16988117 | 0.1310513 | 0.22305981 | 10 | Young Adult (18-24 years) | Mental MM -> Physical-Mental MM |
| State 6 | State 6 | 0.94928263 | 0.92917122 | 0.96491822 | 10 | Young Adult (18-24 years) | Physical-Mental MM -> Physical-Mental MM |
| State 1 | State 7 | 0.00266302 | 0.00250597 | 0.00284921 | 10 | Young Adult (18-24 years) | DF -> Death |
| State 2 | State 7 | 0.01333485 | 0.01091288 | 0.01672201 | 10 | Young Adult (18-24 years) | Physical M -> Death |
| State 3 | State 7 | 0.02495758 | 0.01992097 | 0.03165018 | 10 | Young Adult (18-24 years) | Mental M -> Death |
| State 4 | State 7 | 0.05377704 | 0.04028533 | 0.07209616 | 10 | Young Adult (18-24 years) | Physical MM -> Death |
| State 5 | State 7 | 0.05495981 | 0.03657573 | 0.08637083 | 10 | Young Adult (18-24 years) | Mental MM -> Death |
| State 6 | State 7 | 0.05071737 | 0.03508178 | 0.07082878 | 10 | Young Adult (18-24 years) | Physical-Mental MM -> Death |
| State 1 | State 1 | 0.94426122 | 0.94342869 | 0.94500254 | 11 | Young Adult (18-24 years) | DF -> DF |
| State 1 | State 2 | 0.03058282 | 0.03000126 | 0.03120585 | 11 | Young Adult (18-24 years) | DF -> Physical M |
| State 2 | State 2 | 0.77010085 | 0.75590192 | 0.78205696 | 11 | Young Adult (18-24 years) | Physical M -> Physical M |
| State 1 | State 3 | 0.01186372 | 0.01146213 | 0.01227389 | 11 | Young Adult (18-24 years) | DF -> Mental M |
| State 3 | State 3 | 0.70783778 | 0.6866831 | 0.72855242 | 11 | Young Adult (18-24 years) | Mental M -> Mental M |
| State 1 | State 4 | 0.00457471 | 0.00431123 | 0.00482568 | 11 | Young Adult (18-24 years) | DF -> Physical MM |
| State 2 | State 4 | 0.13427922 | 0.12405762 | 0.14478201 | 11 | Young Adult (18-24 years) | Physical M -> Physical MM |
| State 4 | State 4 | 0.80997841 | 0.76952676 | 0.84072521 | 11 | Young Adult (18-24 years) | Physical MM -> Physical MM |
| State 1 | State 5 | 0.00168894 | 0.00154281 | 0.00186317 | 11 | Young Adult (18-24 years) | DF -> Mental MM |
| State 3 | State 5 | 0.12069396 | 0.10728577 | 0.135912 | 11 | Young Adult (18-24 years) | Mental M -> Mental MM |
| State 5 | State 5 | 0.75566601 | 0.6941195 | 0.80762246 | 11 | Young Adult (18-24 years) | Mental MM -> Mental MM |
| State 1 | State 6 | 0.00404922 | 0.00379652 | 0.00431937 | 11 | Young Adult (18-24 years) | DF -> Physical-Mental MM |
| State 2 | State 6 | 0.08049225 | 0.07245585 | 0.08928192 | 11 | Young Adult (18-24 years) | Physical M -> Physical-Mental MM |
| State 3 | State 6 | 0.14362959 | 0.12771906 | 0.16045341 | 11 | Young Adult (18-24 years) | Mental M -> Physical-Mental MM |
| State 4 | State 6 | 0.13105071 | 0.10437416 | 0.16793303 | 11 | Young Adult (18-24 years) | Physical MM -> Physical-Mental MM |
| State 5 | State 6 | 0.18409126 | 0.1385828 | 0.24187985 | 11 | Young Adult (18-24 years) | Mental MM -> Physical-Mental MM |
| State 6 | State 6 | 0.94435458 | 0.92351269 | 0.96032898 | 11 | Young Adult (18-24 years) | Physical-Mental MM -> Physical-Mental MM |
| State 1 | State 7 | 0.00297937 | 0.00279888 | 0.00318553 | 11 | Young Adult (18-24 years) | DF -> Death |
| State 2 | State 7 | 0.01512768 | 0.0123372 | 0.01834479 | 11 | Young Adult (18-24 years) | Physical M -> Death |
| State 3 | State 7 | 0.02783866 | 0.02234669 | 0.03530412 | 11 | Young Adult (18-24 years) | Mental M -> Death |
| State 4 | State 7 | 0.05897087 | 0.04434107 | 0.08040474 | 11 | Young Adult (18-24 years) | Physical MM -> Death |
| State 5 | State 7 | 0.06024274 | 0.03814194 | 0.09401083 | 11 | Young Adult (18-24 years) | Mental MM -> Death |
| State 6 | State 7 | 0.05564542 | 0.03967102 | 0.07648731 | 11 | Young Adult (18-24 years) | Physical-Mental MM -> Death |
| State 1 | State 1 | 0.93935079 | 0.93842359 | 0.9402033 | 12 | Young Adult (18-24 years) | DF -> DF |
| State 1 | State 2 | 0.03289424 | 0.0322269 | 0.0335388 | 12 | Young Adult (18-24 years) | DF -> Physical M |
| State 2 | State 2 | 0.75202754 | 0.73753862 | 0.76594093 | 12 | Young Adult (18-24 years) | Physical M -> Physical M |
| State 1 | State 3 | 0.01271573 | 0.01231228 | 0.01317186 | 12 | Young Adult (18-24 years) | DF -> Mental M |
| State 3 | State 3 | 0.68594825 | 0.66253217 | 0.70662237 | 12 | Young Adult (18-24 years) | Mental M -> Mental M |
| State 1 | State 4 | 0.00516969 | 0.00485785 | 0.00548282 | 12 | Young Adult (18-24 years) | DF -> Physical MM |
| State 2 | State 4 | 0.14338012 | 0.13274865 | 0.15428362 | 12 | Young Adult (18-24 years) | Physical M -> Physical MM |
| State 4 | State 4 | 0.79460784 | 0.75473612 | 0.82926802 | 12 | Young Adult (18-24 years) | Physical MM -> Physical MM |
| State 1 | State 5 | 0.00190216 | 0.0017508 | 0.00208945 | 12 | Young Adult (18-24 years) | DF -> Mental MM |
| State 3 | State 5 | 0.12797854 | 0.11391414 | 0.14308173 | 12 | Young Adult (18-24 years) | Mental M -> Mental MM |
| State 5 | State 5 | 0.73666319 | 0.67129691 | 0.78927713 | 12 | Young Adult (18-24 years) | Mental MM -> Mental MM |
| State 1 | State 6 | 0.00466142 | 0.00438661 | 0.00499663 | 12 | Young Adult (18-24 years) | DF -> Physical-Mental MM |
| State 2 | State 6 | 0.08759874 | 0.07906415 | 0.0975055 | 12 | Young Adult (18-24 years) | Physical M -> Physical-Mental MM |
| State 3 | State 6 | 0.15529549 | 0.13822898 | 0.17301685 | 12 | Young Adult (18-24 years) | Mental M -> Physical-Mental MM |
| State 4 | State 6 | 0.14125975 | 0.11111946 | 0.17531701 | 12 | Young Adult (18-24 years) | Physical MM -> Physical-Mental MM |
| State 5 | State 6 | 0.19784806 | 0.15183372 | 0.25669931 | 12 | Young Adult (18-24 years) | Mental MM -> Physical-Mental MM |
| State 6 | State 6 | 0.9394521 | 0.91371168 | 0.95619918 | 12 | Young Adult (18-24 years) | Physical-Mental MM -> Physical-Mental MM |
| State 1 | State 7 | 0.00330596 | 0.00309604 | 0.00353077 | 12 | Young Adult (18-24 years) | DF -> Death |
| State 2 | State 7 | 0.0169936 | 0.01380674 | 0.02088845 | 12 | Young Adult (18-24 years) | Physical M -> Death |
| State 3 | State 7 | 0.03077772 | 0.0250515 | 0.03893212 | 12 | Young Adult (18-24 years) | Mental M -> Death |
| State 4 | State 7 | 0.06413241 | 0.0476152 | 0.08551284 | 12 | Young Adult (18-24 years) | Physical MM -> Death |
| State 5 | State 7 | 0.06548876 | 0.04095162 | 0.10413482 | 12 | Young Adult (18-24 years) | Mental MM -> Death |
| State 6 | State 7 | 0.0605479 | 0.04380082 | 0.08628832 | 12 | Young Adult (18-24 years) | Physical-Mental MM -> Death |
| State 1 | State 1 | 0.9344659 | 0.93348538 | 0.93543782 | 13 | Young Adult (18-24 years) | DF -> DF |
| State 1 | State 2 | 0.03513566 | 0.03439005 | 0.03588361 | 13 | Young Adult (18-24 years) | DF -> Physical M |
| State 2 | State 2 | 0.73437839 | 0.71889177 | 0.7498844 | 13 | Young Adult (18-24 years) | Physical M -> Physical M |
| State 1 | State 3 | 0.01353506 | 0.01305795 | 0.01399808 | 13 | Young Adult (18-24 years) | DF -> Mental M |
| State 3 | State 3 | 0.66473564 | 0.63975418 | 0.68741135 | 13 | Young Adult (18-24 years) | Mental M -> Mental M |
| State 1 | State 4 | 0.00578722 | 0.00545807 | 0.00613511 | 13 | Young Adult (18-24 years) | DF -> Physical MM |
| State 2 | State 4 | 0.15203492 | 0.14068634 | 0.16341245 | 13 | Young Adult (18-24 years) | Physical M -> Physical MM |
| State 4 | State 4 | 0.77952896 | 0.7353781 | 0.81584401 | 13 | Young Adult (18-24 years) | Physical MM -> Physical MM |
| State 1 | State 5 | 0.002122 | 0.00192555 | 0.00233903 | 13 | Young Adult (18-24 years) | DF -> Mental MM |
| State 3 | State 5 | 0.1347608 | 0.11915843 | 0.15258507 | 13 | Young Adult (18-24 years) | Mental M -> Mental MM |
| State 5 | State 5 | 0.71813823 | 0.6437846 | 0.77055944 | 13 | Young Adult (18-24 years) | Mental MM -> Mental MM |
| State 1 | State 6 | 0.00531113 | 0.00498897 | 0.00566309 | 13 | Young Adult (18-24 years) | DF -> Physical-Mental MM |
| State 2 | State 6 | 0.09465647 | 0.08440761 | 0.10517338 | 13 | Young Adult (18-24 years) | Physical M -> Physical-Mental MM |
| State 3 | State 6 | 0.16673149 | 0.14972403 | 0.18703965 | 13 | Young Adult (18-24 years) | Mental M -> Physical-Mental MM |
| State 4 | State 6 | 0.15120915 | 0.12079468 | 0.18854508 | 13 | Young Adult (18-24 years) | Physical MM -> Physical-Mental MM |
| State 5 | State 6 | 0.21116346 | 0.16336562 | 0.27046953 | 13 | Young Adult (18-24 years) | Mental MM -> Physical-Mental MM |
| State 6 | State 6 | 0.93457508 | 0.9102173 | 0.95329174 | 13 | Young Adult (18-24 years) | Physical-Mental MM -> Physical-Mental MM |
| State 1 | State 7 | 0.00364304 | 0.00343029 | 0.00391248 | 13 | Young Adult (18-24 years) | DF -> Death |
| State 2 | State 7 | 0.01893022 | 0.01586929 | 0.02326885 | 13 | Young Adult (18-24 years) | Physical M -> Death |
| State 3 | State 7 | 0.03377206 | 0.02720711 | 0.04322213 | 13 | Young Adult (18-24 years) | Mental M -> Death |
| State 4 | State 7 | 0.0692619 | 0.05154351 | 0.09407081 | 13 | Young Adult (18-24 years) | Physical MM -> Death |
| State 5 | State 7 | 0.07069831 | 0.04365631 | 0.11265925 | 13 | Young Adult (18-24 years) | Mental MM -> Death |
| State 6 | State 7 | 0.06542492 | 0.04670826 | 0.0897827 | 13 | Young Adult (18-24 years) | Physical-Mental MM -> Death |
| State 1 | State 1 | 0.9296064 | 0.92865199 | 0.93056098 | 14 | Young Adult (18-24 years) | DF -> DF |
| State 1 | State 2 | 0.03730881 | 0.03646789 | 0.03809524 | 14 | Young Adult (18-24 years) | DF -> Physical M |
| State 2 | State 2 | 0.71714345 | 0.69973892 | 0.73264045 | 14 | Young Adult (18-24 years) | Physical M -> Physical M |
| State 1 | State 3 | 0.01432274 | 0.01382127 | 0.01480982 | 14 | Young Adult (18-24 years) | DF -> Mental M |
| State 3 | State 3 | 0.64417902 | 0.61833164 | 0.66762223 | 14 | Young Adult (18-24 years) | Mental M -> Mental M |
| State 1 | State 4 | 0.00642579 | 0.00605804 | 0.00680376 | 14 | Young Adult (18-24 years) | DF -> Physical MM |
| State 2 | State 4 | 0.1602585 | 0.14845976 | 0.17235517 | 14 | Young Adult (18-24 years) | Physical M -> Physical MM |
| State 4 | State 4 | 0.76473621 | 0.71607111 | 0.80429193 | 14 | Young Adult (18-24 years) | Physical MM -> Physical MM |
| State 1 | State 5 | 0.00234784 | 0.00213714 | 0.00258402 | 14 | Young Adult (18-24 years) | DF -> Mental MM |
| State 3 | State 5 | 0.14106325 | 0.12454865 | 0.15878972 | 14 | Young Adult (18-24 years) | Mental M -> Mental MM |
| State 5 | State 5 | 0.70007912 | 0.62948925 | 0.75839044 | 14 | Young Adult (18-24 years) | Mental MM -> Mental MM |
| State 1 | State 6 | 0.0059976 | 0.00562363 | 0.00640682 | 14 | Young Adult (18-24 years) | DF -> Physical-Mental MM |
| State 2 | State 6 | 0.10166285 | 0.09227027 | 0.11378469 | 14 | Young Adult (18-24 years) | Physical M -> Physical-Mental MM |
| State 3 | State 6 | 0.17793865 | 0.15935557 | 0.19690339 | 14 | Young Adult (18-24 years) | Mental M -> Physical-Mental MM |
| State 4 | State 6 | 0.16090417 | 0.12668199 | 0.20120001 | 14 | Young Adult (18-24 years) | Physical MM -> Physical-Mental MM |
| State 5 | State 6 | 0.22404907 | 0.17007107 | 0.28512869 | 14 | Young Adult (18-24 years) | Mental MM -> Physical-Mental MM |
| State 6 | State 6 | 0.92972337 | 0.90211782 | 0.94915118 | 14 | Young Adult (18-24 years) | Physical-Mental MM -> Physical-Mental MM |
| State 1 | State 7 | 0.00399082 | 0.00375892 | 0.00427702 | 14 | Young Adult (18-24 years) | DF -> Death |
| State 2 | State 7 | 0.02093519 | 0.01756447 | 0.02574559 | 14 | Young Adult (18-24 years) | Physical M -> Death |
| State 3 | State 7 | 0.03681908 | 0.02936231 | 0.04623003 | 14 | Young Adult (18-24 years) | Mental M -> Death |
| State 4 | State 7 | 0.07435962 | 0.05565199 | 0.10088787 | 14 | Young Adult (18-24 years) | Physical MM -> Death |
| State 5 | State 7 | 0.0758718 | 0.04788726 | 0.11784714 | 14 | Young Adult (18-24 years) | Mental MM -> Death |
| State 6 | State 7 | 0.07027663 | 0.05084882 | 0.09788218 | 14 | Young Adult (18-24 years) | Physical-Mental MM -> Death |
| State 1 | State 1 | 0.92477218 | 0.92376355 | 0.92589054 | 15 | Young Adult (18-24 years) | DF -> DF |
| State 1 | State 2 | 0.03941536 | 0.03857032 | 0.04020594 | 15 | Young Adult (18-24 years) | DF -> Physical M |
| State 2 | State 2 | 0.70031299 | 0.68243457 | 0.71593279 | 15 | Young Adult (18-24 years) | Physical M -> Physical M |
| State 1 | State 3 | 0.01507979 | 0.014531 | 0.01560486 | 15 | Young Adult (18-24 years) | DF -> Mental M |
| State 3 | State 3 | 0.62425811 | 0.5989835 | 0.65055188 | 15 | Young Adult (18-24 years) | Mental M -> Mental M |
| State 1 | State 4 | 0.00708399 | 0.00670998 | 0.00751823 | 15 | Young Adult (18-24 years) | DF -> Physical MM |
| State 2 | State 4 | 0.16806533 | 0.15561782 | 0.18131113 | 15 | Young Adult (18-24 years) | Physical M -> Physical MM |
| State 4 | State 4 | 0.75022419 | 0.70356523 | 0.79121847 | 15 | Young Adult (18-24 years) | Physical MM -> Physical MM |
| State 1 | State 5 | 0.00257905 | 0.00232331 | 0.00283916 | 15 | Young Adult (18-24 years) | DF -> Mental MM |
| State 3 | State 5 | 0.14690751 | 0.12880817 | 0.1641957 | 15 | Young Adult (18-24 years) | Mental M -> Mental MM |
| State 5 | State 5 | 0.68247415 | 0.61088958 | 0.74197671 | 15 | Young Adult (18-24 years) | Mental MM -> Mental MM |
| State 1 | State 6 | 0.00672008 | 0.00626784 | 0.00718038 | 15 | Young Adult (18-24 years) | DF -> Physical-Mental MM |
| State 2 | State 6 | 0.10861542 | 0.09749761 | 0.1210099 | 15 | Young Adult (18-24 years) | Physical M -> Physical-Mental MM |
| State 3 | State 6 | 0.18891812 | 0.16842721 | 0.21028029 | 15 | Young Adult (18-24 years) | Mental M -> Physical-Mental MM |
| State 4 | State 6 | 0.17034999 | 0.13336212 | 0.21157801 | 15 | Young Adult (18-24 years) | Physical MM -> Physical-Mental MM |
| State 5 | State 6 | 0.23651618 | 0.18107183 | 0.30473618 | 15 | Young Adult (18-24 years) | Mental MM -> Physical-Mental MM |
| State 6 | State 6 | 0.92489686 | 0.89712218 | 0.94609296 | 15 | Young Adult (18-24 years) | Physical-Mental MM -> Physical-Mental MM |
| State 1 | State 7 | 0.00434954 | 0.0040683 | 0.0046626 | 15 | Young Adult (18-24 years) | DF -> Death |
| State 2 | State 7 | 0.02300625 | 0.01913078 | 0.02822119 | 15 | Young Adult (18-24 years) | Physical M -> Death |
| State 3 | State 7 | 0.03991626 | 0.03258063 | 0.04925968 | 15 | Young Adult (18-24 years) | Mental M -> Death |
| State 4 | State 7 | 0.07942582 | 0.05979728 | 0.10601021 | 15 | Young Adult (18-24 years) | Physical MM -> Death |
| State 5 | State 7 | 0.08100966 | 0.05116154 | 0.1240375 | 15 | Young Adult (18-24 years) | Mental MM -> Death |
| State 6 | State 7 | 0.07510314 | 0.05390704 | 0.10287782 | 15 | Young Adult (18-24 years) | Physical-Mental MM -> Death |
| State 1 | State 1 | 0.9199631 | 0.91885484 | 0.92107085 | 16 | Young Adult (18-24 years) | DF -> DF |
| State 1 | State 2 | 0.04145697 | 0.04056511 | 0.04231193 | 16 | Young Adult (18-24 years) | DF -> Physical M |
| State 2 | State 2 | 0.68387752 | 0.66704182 | 0.69964026 | 16 | Young Adult (18-24 years) | Physical M -> Physical M |
| State 1 | State 3 | 0.01580719 | 0.01525365 | 0.01640417 | 16 | Young Adult (18-24 years) | DF -> Mental M |
| State 3 | State 3 | 0.60495323 | 0.57818248 | 0.63009508 | 16 | Young Adult (18-24 years) | Mental M -> Mental M |
| State 1 | State 4 | 0.00776045 | 0.00726546 | 0.00824552 | 16 | Young Adult (18-24 years) | DF -> Physical MM |
| State 2 | State 4 | 0.17546942 | 0.16219345 | 0.18948252 | 16 | Young Adult (18-24 years) | Physical M -> Physical MM |
| State 4 | State 4 | 0.73598754 | 0.68739247 | 0.77554805 | 16 | Young Adult (18-24 years) | Physical MM -> Physical MM |
| State 1 | State 5 | 0.00281506 | 0.00255416 | 0.00310125 | 16 | Young Adult (18-24 years) | DF -> Mental MM |
| State 3 | State 5 | 0.15231437 | 0.13458612 | 0.17151523 | 16 | Young Adult (18-24 years) | Mental M -> Mental MM |
| State 5 | State 5 | 0.6653119 | 0.58561152 | 0.7283046 | 16 | Young Adult (18-24 years) | Mental MM -> Mental MM |
| State 1 | State 6 | 0.00747784 | 0.00703139 | 0.00796833 | 16 | Young Adult (18-24 years) | DF -> Physical-Mental MM |
| State 2 | State 6 | 0.11551187 | 0.1039538 | 0.12823752 | 16 | Young Adult (18-24 years) | Physical M -> Physical-Mental MM |
| State 3 | State 6 | 0.19967122 | 0.18085242 | 0.2205098 | 16 | Young Adult (18-24 years) | Mental M -> Physical-Mental MM |
| State 4 | State 6 | 0.17955167 | 0.14421768 | 0.22398459 | 16 | Young Adult (18-24 years) | Physical MM -> Physical-Mental MM |
| State 5 | State 6 | 0.24857581 | 0.18784256 | 0.31100878 | 16 | Young Adult (18-24 years) | Mental MM -> Physical-Mental MM |
| State 6 | State 6 | 0.92009539 | 0.88495886 | 0.94385745 | 16 | Young Adult (18-24 years) | Physical-Mental MM -> Physical-Mental MM |
| State 1 | State 7 | 0.00471938 | 0.00443522 | 0.00507346 | 16 | Young Adult (18-24 years) | DF -> Death |
| State 2 | State 7 | 0.02514119 | 0.02070884 | 0.03056294 | 16 | Young Adult (18-24 years) | Physical M -> Death |
| State 3 | State 7 | 0.04306118 | 0.03527639 | 0.05334585 | 16 | Young Adult (18-24 years) | Mental M -> Death |
| State 4 | State 7 | 0.08446078 | 0.06249104 | 0.11055501 | 16 | Young Adult (18-24 years) | Physical MM -> Death |
| State 5 | State 7 | 0.08611229 | 0.05714602 | 0.13511539 | 16 | Young Adult (18-24 years) | Mental MM -> Death |
| State 6 | State 7 | 0.07990461 | 0.05614255 | 0.11504114 | 16 | Young Adult (18-24 years) | Physical-Mental MM -> Death |
| State 1 | State 1 | 0.91517903 | 0.91402516 | 0.91635867 | 17 | Young Adult (18-24 years) | DF -> DF |
| State 1 | State 2 | 0.04343524 | 0.04242192 | 0.04440902 | 17 | Young Adult (18-24 years) | DF -> Physical M |
| State 2 | State 2 | 0.66782777 | 0.64923755 | 0.68552054 | 17 | Young Adult (18-24 years) | Physical M -> Physical M |
| State 1 | State 3 | 0.01650589 | 0.01585076 | 0.01709286 | 17 | Young Adult (18-24 years) | DF -> Mental M |
| State 3 | State 3 | 0.58624536 | 0.55629402 | 0.61010318 | 17 | Young Adult (18-24 years) | Mental M -> Mental M |
| State 1 | State 4 | 0.00845383 | 0.0079433 | 0.00897536 | 17 | Young Adult (18-24 years) | DF -> Physical MM |
| State 2 | State 4 | 0.1824844 | 0.16905265 | 0.19649313 | 17 | Young Adult (18-24 years) | Physical M -> Physical MM |
| State 4 | State 4 | 0.72202107 | 0.67340964 | 0.76775529 | 17 | Young Adult (18-24 years) | Physical MM -> Physical MM |
| State 1 | State 5 | 0.00305533 | 0.00275366 | 0.00336037 | 17 | Young Adult (18-24 years) | DF -> Mental MM |
| State 3 | State 5 | 0.15730382 | 0.13795253 | 0.1782968 | 17 | Young Adult (18-24 years) | Mental M -> Mental MM |
| State 5 | State 5 | 0.64858122 | 0.5682989 | 0.7127492 | 17 | Young Adult (18-24 years) | Mental MM -> Mental MM |
| State 1 | State 6 | 0.00827013 | 0.00775001 | 0.00887211 | 17 | Young Adult (18-24 years) | DF -> Physical-Mental MM |
| State 2 | State 6 | 0.12234999 | 0.11054765 | 0.13712044 | 17 | Young Adult (18-24 years) | Physical M -> Physical-Mental MM |
| State 3 | State 6 | 0.21019935 | 0.18956648 | 0.23320178 | 17 | Young Adult (18-24 years) | Mental M -> Physical-Mental MM |
| State 4 | State 6 | 0.18851419 | 0.14860262 | 0.22976366 | 17 | Young Adult (18-24 years) | Physical MM -> Physical-Mental MM |
| State 5 | State 6 | 0.2602387 | 0.19917311 | 0.33511113 | 17 | Young Adult (18-24 years) | Mental MM -> Physical-Mental MM |
| State 6 | State 6 | 0.91531886 | 0.88299209 | 0.93857917 | 17 | Young Adult (18-24 years) | Physical-Mental MM -> Physical-Mental MM |
| State 1 | State 7 | 0.00510056 | 0.00477063 | 0.0054836 | 17 | Young Adult (18-24 years) | DF -> Death |
| State 2 | State 7 | 0.02733784 | 0.02252905 | 0.03346115 | 17 | Young Adult (18-24 years) | Physical M -> Death |
| State 3 | State 7 | 0.04625148 | 0.03679828 | 0.05748807 | 17 | Young Adult (18-24 years) | Mental M -> Death |
| State 4 | State 7 | 0.08946474 | 0.06704978 | 0.11760102 | 17 | Young Adult (18-24 years) | Physical MM -> Death |
| State 5 | State 7 | 0.09118008 | 0.06081748 | 0.1427777 | 17 | Young Adult (18-24 years) | Mental MM -> Death |
| State 6 | State 7 | 0.08468114 | 0.06142083 | 0.11700791 | 17 | Young Adult (18-24 years) | Physical-Mental MM -> Death |
| State 1 | State 1 | 0.91041983 | 0.90905601 | 0.91172333 | 18 | Young Adult (18-24 years) | DF -> DF |
| State 1 | State 2 | 0.04535174 | 0.0443378 | 0.0464283 | 18 | Young Adult (18-24 years) | DF -> Physical M |
| State 2 | State 2 | 0.65215469 | 0.63322753 | 0.6699906 | 18 | Young Adult (18-24 years) | Physical M -> Physical M |
| State 1 | State 3 | 0.0171768 | 0.01652721 | 0.01784758 | 18 | Young Adult (18-24 years) | DF -> Mental M |
| State 3 | State 3 | 0.56811601 | 0.53758931 | 0.59731595 | 18 | Young Adult (18-24 years) | Mental M -> Mental M |
| State 1 | State 4 | 0.00916287 | 0.00860371 | 0.00973913 | 18 | Young Adult (18-24 years) | DF -> Physical MM |
| State 2 | State 4 | 0.18912348 | 0.174985 | 0.20414471 | 18 | Young Adult (18-24 years) | Physical M -> Physical MM |
| State 4 | State 4 | 0.70831962 | 0.64904133 | 0.75501636 | 18 | Young Adult (18-24 years) | Physical MM -> Physical MM |
| State 1 | State 5 | 0.00329931 | 0.00296047 | 0.00363221 | 18 | Young Adult (18-24 years) | DF -> Mental MM |
| State 3 | State 5 | 0.16189505 | 0.14140024 | 0.18216881 | 18 | Young Adult (18-24 years) | Mental M -> Mental MM |
| State 5 | State 5 | 0.63227127 | 0.54815754 | 0.698098 | 18 | Young Adult (18-24 years) | Mental MM -> Mental MM |
| State 1 | State 6 | 0.0090962 | 0.00853513 | 0.00973095 | 18 | Young Adult (18-24 years) | DF -> Physical-Mental MM |
| State 2 | State 6 | 0.12912773 | 0.11654378 | 0.14352189 | 18 | Young Adult (18-24 years) | Physical M -> Physical-Mental MM |
| State 3 | State 6 | 0.22050404 | 0.19827447 | 0.2461002 | 18 | Young Adult (18-24 years) | Mental M -> Physical-Mental MM |
| State 4 | State 6 | 0.19724241 | 0.15658513 | 0.24756474 | 18 | Young Adult (18-24 years) | Physical MM -> Physical-Mental MM |
| State 5 | State 6 | 0.27151529 | 0.20929292 | 0.34609648 | 18 | Young Adult (18-24 years) | Mental MM -> Physical-Mental MM |
| State 6 | State 6 | 0.91056712 | 0.877024 | 0.93629968 | 18 | Young Adult (18-24 years) | Physical-Mental MM -> Physical-Mental MM |
| State 1 | State 7 | 0.00549325 | 0.00514211 | 0.00588303 | 18 | Young Adult (18-24 years) | DF -> Death |
| State 2 | State 7 | 0.02959411 | 0.02488844 | 0.03615601 | 18 | Young Adult (18-24 years) | Physical M -> Death |
| State 3 | State 7 | 0.04948491 | 0.03976452 | 0.06215357 | 18 | Young Adult (18-24 years) | Mental M -> Death |
| State 4 | State 7 | 0.09443796 | 0.07096629 | 0.123572 | 18 | Young Adult (18-24 years) | Physical MM -> Death |
| State 5 | State 7 | 0.09621343 | 0.06352034 | 0.14797412 | 18 | Young Adult (18-24 years) | Mental MM -> Death |
| State 6 | State 7 | 0.08943288 | 0.06370032 | 0.122976 | 18 | Young Adult (18-24 years) | Physical-Mental MM -> Death |
| State 1 | State 1 | 0.90568538 | 0.90421335 | 0.9071022 | 19 | Young Adult (18-24 years) | DF -> DF |
| State 1 | State 2 | 0.04720799 | 0.04609923 | 0.04835621 | 19 | Young Adult (18-24 years) | DF -> Physical M |
| State 2 | State 2 | 0.63684943 | 0.61650116 | 0.65438366 | 19 | Young Adult (18-24 years) | Physical M -> Physical M |
| State 1 | State 3 | 0.01782082 | 0.01710942 | 0.01852788 | 19 | Young Adult (18-24 years) | DF -> Mental M |
| State 3 | State 3 | 0.55054731 | 0.52095761 | 0.57737341 | 19 | Young Adult (18-24 years) | Mental M -> Mental M |
| State 1 | State 4 | 0.00988634 | 0.00929041 | 0.01050569 | 19 | Young Adult (18-24 years) | DF -> Physical MM |
| State 2 | State 4 | 0.19539948 | 0.18048651 | 0.21091061 | 19 | Young Adult (18-24 years) | Physical M -> Physical MM |
| State 4 | State 4 | 0.69487818 | 0.6367696 | 0.73881619 | 19 | Young Adult (18-24 years) | Physical MM -> Physical MM |
| State 1 | State 5 | 0.00354653 | 0.0031835 | 0.00389763 | 19 | Young Adult (18-24 years) | DF -> Mental MM |
| State 3 | State 5 | 0.16610651 | 0.1452801 | 0.18658282 | 19 | Young Adult (18-24 years) | Mental M -> Mental MM |
| State 5 | State 5 | 0.61637147 | 0.53333577 | 0.68775628 | 19 | Young Adult (18-24 years) | Mental MM -> Mental MM |
| State 1 | State 6 | 0.00995531 | 0.00937172 | 0.01061778 | 19 | Young Adult (18-24 years) | DF -> Physical-Mental MM |
| State 2 | State 6 | 0.13584315 | 0.12309789 | 0.15022936 | 19 | Young Adult (18-24 years) | Physical M -> Physical-Mental MM |
| State 3 | State 6 | 0.23058692 | 0.20828689 | 0.25647481 | 19 | Young Adult (18-24 years) | Mental M -> Physical-Mental MM |
| State 4 | State 6 | 0.20574112 | 0.165339 | 0.2558973 | 19 | Young Adult (18-24 years) | Physical MM -> Physical-Mental MM |
| State 5 | State 6 | 0.2824158 | 0.21767466 | 0.35566137 | 19 | Young Adult (18-24 years) | Mental MM -> Physical-Mental MM |
| State 6 | State 6 | 0.90584005 | 0.87035305 | 0.93427522 | 19 | Young Adult (18-24 years) | Physical-Mental MM -> Physical-Mental MM |
| State 1 | State 7 | 0.00589762 | 0.00551131 | 0.00633131 | 19 | Young Adult (18-24 years) | DF -> Death |
| State 2 | State 7 | 0.03190794 | 0.02642656 | 0.03874391 | 19 | Young Adult (18-24 years) | Physical M -> Death |
| State 3 | State 7 | 0.05275927 | 0.04354678 | 0.0663269 | 19 | Young Adult (18-24 years) | Mental M -> Death |
| State 4 | State 7 | 0.09938069 | 0.07641468 | 0.1316102 | 19 | Young Adult (18-24 years) | Physical MM -> Death |
| State 5 | State 7 | 0.10121272 | 0.06690744 | 0.15380324 | 19 | Young Adult (18-24 years) | Mental MM -> Death |
| State 6 | State 7 | 0.09415995 | 0.06572478 | 0.12964695 | 19 | Young Adult (18-24 years) | Physical-Mental MM -> Death |
| State 1 | State 1 | 0.90097556 | 0.89949619 | 0.9023049 | 20 | Young Adult (18-24 years) | DF -> DF |
| State 1 | State 2 | 0.04900549 | 0.04789919 | 0.05017914 | 20 | Young Adult (18-24 years) | DF -> Physical M |
| State 2 | State 2 | 0.62190337 | 0.6023068 | 0.6406163 | 20 | Young Adult (18-24 years) | Physical M -> Physical M |
| State 1 | State 3 | 0.01843882 | 0.0177299 | 0.01916335 | 20 | Young Adult (18-24 years) | DF -> Mental M |
| State 3 | State 3 | 0.5335219 | 0.50429245 | 0.56224145 | 20 | Young Adult (18-24 years) | Mental M -> Mental M |
| State 1 | State 4 | 0.01062306 | 0.00990916 | 0.01129789 | 20 | Young Adult (18-24 years) | DF -> Physical MM |
| State 2 | State 4 | 0.20132488 | 0.18541781 | 0.21672237 | 20 | Young Adult (18-24 years) | Physical M -> Physical MM |
| State 4 | State 4 | 0.68169182 | 0.62071565 | 0.72902105 | 20 | Young Adult (18-24 years) | Physical MM -> Physical MM |
| State 1 | State 5 | 0.0037965 | 0.00340494 | 0.00417722 | 20 | Young Adult (18-24 years) | DF -> Mental MM |
| State 3 | State 5 | 0.16995593 | 0.14803325 | 0.19035409 | 20 | Young Adult (18-24 years) | Mental M -> Mental MM |
| State 5 | State 5 | 0.60087151 | 0.51853491 | 0.6719393 | 20 | Young Adult (18-24 years) | Mental MM -> Mental MM |
| State 1 | State 6 | 0.01084673 | 0.01016783 | 0.01161075 | 20 | Young Adult (18-24 years) | DF -> Physical-Mental MM |
| State 2 | State 6 | 0.14249442 | 0.12987068 | 0.15864121 | 20 | Young Adult (18-24 years) | Physical M -> Physical-Mental MM |
| State 3 | State 6 | 0.24044972 | 0.21653415 | 0.2677058 | 20 | Young Adult (18-24 years) | Mental M -> Physical-Mental MM |
| State 4 | State 6 | 0.21401501 | 0.17348618 | 0.26739177 | 20 | Young Adult (18-24 years) | Physical MM -> Physical-Mental MM |
| State 5 | State 6 | 0.29295016 | 0.2277778 | 0.36603179 | 20 | Young Adult (18-24 years) | Mental MM -> Physical-Mental MM |
| State 6 | State 6 | 0.90113752 | 0.86540602 | 0.92934366 | 20 | Young Adult (18-24 years) | Physical-Mental MM -> Physical-Mental MM |
| State 1 | State 7 | 0.00631384 | 0.00592114 | 0.00681292 | 20 | Young Adult (18-24 years) | DF -> Death |
| State 2 | State 7 | 0.03427733 | 0.02888789 | 0.04164069 | 20 | Young Adult (18-24 years) | Physical M -> Death |
| State 3 | State 7 | 0.05607245 | 0.04593787 | 0.07082454 | 20 | Young Adult (18-24 years) | Mental M -> Death |
| State 4 | State 7 | 0.10429318 | 0.07828146 | 0.13768981 | 20 | Young Adult (18-24 years) | Physical MM -> Death |
| State 5 | State 7 | 0.10617833 | 0.06977256 | 0.15572431 | 20 | Young Adult (18-24 years) | Mental MM -> Death |
| State 6 | State 7 | 0.09886248 | 0.07065634 | 0.13459398 | 20 | Young Adult (18-24 years) | Physical-Mental MM -> Death |
| State 1 | State 1 | 1 | 1 | 1 | 0 | Adult (25-44 years) | DF -> DF |
| State 1 | State 2 | 0 | 0 | 0 | 0 | Adult (25-44 years) | DF -> Physical M |
| State 2 | State 2 | 1 | 1 | 1 | 0 | Adult (25-44 years) | Physical M -> Physical M |
| State 1 | State 3 | 0 | 0 | 0 | 0 | Adult (25-44 years) | DF -> Mental M |
| State 3 | State 3 | 1 | 1 | 1 | 0 | Adult (25-44 years) | Mental M -> Mental M |
| State 1 | State 4 | 0 | 0 | 0 | 0 | Adult (25-44 years) | DF -> Physical MM |
| State 2 | State 4 | 0 | 0 | 0 | 0 | Adult (25-44 years) | Physical M -> Physical MM |
| State 4 | State 4 | 1 | 1 | 1 | 0 | Adult (25-44 years) | Physical MM -> Physical MM |
| State 1 | State 5 | 0 | 0 | 0 | 0 | Adult (25-44 years) | DF -> Mental MM |
| State 3 | State 5 | 0 | 0 | 0 | 0 | Adult (25-44 years) | Mental M -> Mental MM |
| State 5 | State 5 | 1 | 1 | 1 | 0 | Adult (25-44 years) | Mental MM -> Mental MM |
| State 1 | State 6 | 0 | 0 | 0 | 0 | Adult (25-44 years) | DF -> Physical-Mental MM |
| State 2 | State 6 | 0 | 0 | 0 | 0 | Adult (25-44 years) | Physical M -> Physical-Mental MM |
| State 3 | State 6 | 0 | 0 | 0 | 0 | Adult (25-44 years) | Mental M -> Physical-Mental MM |
| State 4 | State 6 | 0 | 0 | 0 | 0 | Adult (25-44 years) | Physical MM -> Physical-Mental MM |
| State 5 | State 6 | 0 | 0 | 0 | 0 | Adult (25-44 years) | Mental MM -> Physical-Mental MM |
| State 6 | State 6 | 1 | 1 | 1 | 0 | Adult (25-44 years) | Physical-Mental MM -> Physical-Mental MM |
| State 1 | State 7 | 0 | 0 | 0 | 0 | Adult (25-44 years) | DF -> Death |
| State 2 | State 7 | 0 | 0 | 0 | 0 | Adult (25-44 years) | Physical M -> Death |
| State 3 | State 7 | 0 | 0 | 0 | 0 | Adult (25-44 years) | Mental M -> Death |
| State 4 | State 7 | 0 | 0 | 0 | 0 | Adult (25-44 years) | Physical MM -> Death |
| State 5 | State 7 | 0 | 0 | 0 | 0 | Adult (25-44 years) | Mental MM -> Death |
| State 6 | State 7 | 0 | 0 | 0 | 0 | Adult (25-44 years) | Physical-Mental MM -> Death |
| State 1 | State 1 | 0.98721679 | 0.9870977 | 0.98733397 | 1 | Adult (25-44 years) | DF -> DF |
| State 1 | State 2 | 0.00865645 | 0.00855934 | 0.00874926 | 1 | Adult (25-44 years) | DF -> Physical M |
| State 2 | State 2 | 0.9636624 | 0.96252289 | 0.96474558 | 1 | Adult (25-44 years) | Physical M -> Physical M |
| State 1 | State 3 | 0.00151016 | 0.00147242 | 0.00155461 | 1 | Adult (25-44 years) | DF -> Mental M |
| State 3 | State 3 | 0.94928558 | 0.94654751 | 0.95190084 | 1 | Adult (25-44 years) | Mental M -> Mental M |
| State 1 | State 4 | 0.00143519 | 0.0013977 | 0.00147178 | 1 | Adult (25-44 years) | DF -> Physical MM |
| State 2 | State 4 | 0.02809789 | 0.02714134 | 0.02904125 | 1 | Adult (25-44 years) | Physical M -> Physical MM |
| State 4 | State 4 | 0.97513198 | 0.97329457 | 0.97679543 | 1 | Adult (25-44 years) | Physical MM -> Physical MM |
| State 1 | State 5 | 0.00012383 | 0.00011267 | 0.00013629 | 1 | Adult (25-44 years) | DF -> Mental MM |
| State 3 | State 5 | 0.01547011 | 0.01403316 | 0.01703744 | 1 | Adult (25-44 years) | Mental M -> Mental MM |
| State 5 | State 5 | 0.95452252 | 0.94752394 | 0.96036547 | 1 | Adult (25-44 years) | Mental MM -> Mental MM |
| State 1 | State 6 | 0.00046757 | 0.00044626 | 0.00048978 | 1 | Adult (25-44 years) | DF -> Physical-Mental MM |
| State 2 | State 6 | 0.00609867 | 0.00570932 | 0.00658828 | 1 | Adult (25-44 years) | Physical M -> Physical-Mental MM |
| State 3 | State 6 | 0.02858953 | 0.02664087 | 0.03065787 | 1 | Adult (25-44 years) | Mental M -> Physical-Mental MM |
| State 4 | State 6 | 0.01039966 | 0.00933685 | 0.01161885 | 1 | Adult (25-44 years) | Physical MM -> Physical-Mental MM |
| State 5 | State 6 | 0.03480428 | 0.02969135 | 0.04115342 | 1 | Adult (25-44 years) | Mental MM -> Physical-Mental MM |
| State 6 | State 6 | 0.98037611 | 0.97803403 | 0.98237874 | 1 | Adult (25-44 years) | Physical-Mental MM -> Physical-Mental MM |
| State 1 | State 7 | 0.00059002 | 0.00056438 | 0.00061601 | 1 | Adult (25-44 years) | DF -> Death |
| State 2 | State 7 | 0.00214104 | 0.00190717 | 0.00240804 | 1 | Adult (25-44 years) | Physical M -> Death |
| State 3 | State 7 | 0.00665478 | 0.00568073 | 0.00767691 | 1 | Adult (25-44 years) | Mental M -> Death |
| State 4 | State 7 | 0.01446835 | 0.0132187 | 0.01584618 | 1 | Adult (25-44 years) | Physical MM -> Death |
| State 5 | State 7 | 0.01067319 | 0.00799517 | 0.01427298 | 1 | Adult (25-44 years) | Mental MM -> Death |
| State 6 | State 7 | 0.01962389 | 0.01762126 | 0.02196597 | 1 | Adult (25-44 years) | Physical-Mental MM -> Death |
| State 1 | State 1 | 0.97459699 | 0.97435026 | 0.97484282 | 2 | Adult (25-44 years) | DF -> DF |
| State 1 | State 2 | 0.01688768 | 0.01669038 | 0.01706534 | 2 | Adult (25-44 years) | DF -> Physical M |
| State 2 | State 2 | 0.92864522 | 0.92646032 | 0.9307889 | 2 | Adult (25-44 years) | Physical M -> Physical M |
| State 1 | State 3 | 0.00292442 | 0.00283905 | 0.00301181 | 2 | Adult (25-44 years) | DF -> Mental M |
| State 3 | State 3 | 0.90114311 | 0.89565004 | 0.90646442 | 2 | Adult (25-44 years) | Mental M -> Mental M |
| State 1 | State 4 | 0.00305958 | 0.00297879 | 0.00313624 | 2 | Adult (25-44 years) | DF -> Physical MM |
| State 2 | State 4 | 0.05447603 | 0.05272811 | 0.05637929 | 2 | Adult (25-44 years) | Physical M -> Physical MM |
| State 4 | State 4 | 0.95088239 | 0.94715493 | 0.95423734 | 2 | Adult (25-44 years) | Physical MM -> Physical MM |
| State 1 | State 5 | 0.0002638 | 0.00024219 | 0.0002872 | 2 | Adult (25-44 years) | DF -> Mental MM |
| State 3 | State 5 | 0.02945213 | 0.0264872 | 0.03254963 | 2 | Adult (25-44 years) | Mental M -> Mental MM |
| State 5 | State 5 | 0.91111325 | 0.89827834 | 0.9227097 | 2 | Adult (25-44 years) | Mental MM -> Mental MM |
| State 1 | State 6 | 0.00103519 | 0.00099177 | 0.00108101 | 2 | Adult (25-44 years) | DF -> Physical-Mental MM |
| State 2 | State 6 | 0.01214825 | 0.011299 | 0.01307782 | 2 | Adult (25-44 years) | Physical M -> Physical-Mental MM |
| State 3 | State 6 | 0.05570655 | 0.0517784 | 0.05975138 | 2 | Adult (25-44 years) | Mental M -> Physical-Mental MM |
| State 4 | State 6 | 0.02033663 | 0.01818953 | 0.02291271 | 2 | Adult (25-44 years) | Physical MM -> Physical-Mental MM |
| State 5 | State 6 | 0.06734276 | 0.05765807 | 0.07921053 | 2 | Adult (25-44 years) | Mental MM -> Physical-Mental MM |
| State 6 | State 6 | 0.96113731 | 0.95674985 | 0.9652558 | 2 | Adult (25-44 years) | Physical-Mental MM -> Physical-Mental MM |
| State 1 | State 7 | 0.00123234 | 0.00117965 | 0.00128898 | 2 | Adult (25-44 years) | DF -> Death |
| State 2 | State 7 | 0.0047305 | 0.00422781 | 0.00527404 | 2 | Adult (25-44 years) | Physical M -> Death |
| State 3 | State 7 | 0.01369821 | 0.01198832 | 0.01589411 | 2 | Adult (25-44 years) | Mental M -> Death |
| State 4 | State 7 | 0.02878099 | 0.02622532 | 0.03144188 | 2 | Adult (25-44 years) | Physical MM -> Death |
| State 5 | State 7 | 0.02154399 | 0.0164362 | 0.02857145 | 2 | Adult (25-44 years) | Mental MM -> Death |
| State 6 | State 7 | 0.03886269 | 0.0347442 | 0.04325015 | 2 | Adult (25-44 years) | Physical-Mental MM -> Death |
| State 1 | State 1 | 0.96213851 | 0.96178605 | 0.96249135 | 3 | Adult (25-44 years) | DF -> DF |
| State 1 | State 2 | 0.02471057 | 0.02442944 | 0.02500304 | 3 | Adult (25-44 years) | DF -> Physical M |
| State 2 | State 2 | 0.89490049 | 0.89174158 | 0.89790313 | 3 | Adult (25-44 years) | Physical M -> Physical M |
| State 1 | State 3 | 0.00424791 | 0.0041296 | 0.00436547 | 3 | Adult (25-44 years) | DF -> Mental M |
| State 3 | State 3 | 0.85544215 | 0.84836632 | 0.86237721 | 3 | Adult (25-44 years) | Mental M -> Mental M |
| State 1 | State 4 | 0.00485673 | 0.00474762 | 0.00497488 | 3 | Adult (25-44 years) | DF -> Physical MM |
| State 2 | State 4 | 0.07921428 | 0.0765574 | 0.08188397 | 3 | Adult (25-44 years) | Physical M -> Physical MM |
| State 4 | State 4 | 0.92723583 | 0.92141941 | 0.93189345 | 3 | Adult (25-44 years) | Physical MM -> Physical MM |
| State 1 | State 5 | 0.00041772 | 0.0003853 | 0.00045353 | 3 | Adult (25-44 years) | DF -> Mental MM |
| State 3 | State 5 | 0.0420535 | 0.03824377 | 0.04619803 | 3 | Adult (25-44 years) | Mental M -> Mental MM |
| State 5 | State 5 | 0.86967812 | 0.85177472 | 0.88566328 | 3 | Adult (25-44 years) | Mental MM -> Mental MM |
| State 1 | State 6 | 0.00169817 | 0.00163376 | 0.0017709 | 3 | Adult (25-44 years) | DF -> Physical-Mental MM |
| State 2 | State 6 | 0.01813989 | 0.01688827 | 0.01934419 | 3 | Adult (25-44 years) | Physical M -> Physical-Mental MM |
| State 3 | State 6 | 0.0814017 | 0.07590705 | 0.08688683 | 3 | Adult (25-44 years) | Mental M -> Physical-Mental MM |
| State 4 | State 6 | 0.0298264 | 0.02653248 | 0.03381263 | 3 | Adult (25-44 years) | Physical MM -> Physical-Mental MM |
| State 5 | State 6 | 0.09773188 | 0.08451677 | 0.11294661 | 3 | Adult (25-44 years) | Mental MM -> Physical-Mental MM |
| State 6 | State 6 | 0.94227605 | 0.9359061 | 0.94813768 | 3 | Adult (25-44 years) | Physical-Mental MM -> Physical-Mental MM |
| State 1 | State 7 | 0.00193038 | 0.00185045 | 0.00201402 | 3 | Adult (25-44 years) | DF -> Death |
| State 2 | State 7 | 0.00774534 | 0.00705252 | 0.00850053 | 3 | Adult (25-44 years) | Physical M -> Death |
| State 3 | State 7 | 0.02110264 | 0.01862003 | 0.02423487 | 3 | Adult (25-44 years) | Mental M -> Death |
| State 4 | State 7 | 0.04293777 | 0.03914882 | 0.04699507 | 3 | Adult (25-44 years) | Physical MM -> Death |
| State 5 | State 7 | 0.03259 | 0.02529342 | 0.04215416 | 3 | Adult (25-44 years) | Mental MM -> Death |
| State 6 | State 7 | 0.05772395 | 0.05186232 | 0.0640939 | 3 | Adult (25-44 years) | Physical-Mental MM -> Death |
| State 1 | State 1 | 0.94983929 | 0.94939136 | 0.95028935 | 4 | Adult (25-44 years) | DF -> DF |
| State 1 | State 2 | 0.03214135 | 0.03179203 | 0.03249569 | 4 | Adult (25-44 years) | DF -> Physical M |
| State 2 | State 2 | 0.86238195 | 0.85821031 | 0.86591619 | 4 | Adult (25-44 years) | Physical M -> Physical M |
| State 1 | State 3 | 0.00548546 | 0.00533669 | 0.00564298 | 4 | Adult (25-44 years) | DF -> Mental M |
| State 3 | State 3 | 0.8120589 | 0.80304143 | 0.8211326 | 4 | Adult (25-44 years) | Mental M -> Mental M |
| State 1 | State 4 | 0.00681113 | 0.00665058 | 0.00697774 | 4 | Adult (25-44 years) | DF -> Physical MM |
| State 2 | State 4 | 0.10238919 | 0.09930409 | 0.10599746 | 4 | Adult (25-44 years) | Physical M -> Physical MM |
| State 4 | State 4 | 0.90417731 | 0.89730516 | 0.91046932 | 4 | Adult (25-44 years) | Physical MM -> Physical MM |
| State 1 | State 5 | 0.00058358 | 0.00054048 | 0.00063052 | 4 | Adult (25-44 years) | DF -> Mental MM |
| State 3 | State 5 | 0.0533748 | 0.0484287 | 0.05868389 | 4 | Adult (25-44 years) | Mental M -> Mental MM |
| State 5 | State 5 | 0.83012735 | 0.80698197 | 0.85089463 | 4 | Adult (25-44 years) | Mental MM -> Mental MM |
| State 1 | State 6 | 0.0024519 | 0.00235744 | 0.00254362 | 4 | Adult (25-44 years) | DF -> Physical-Mental MM |
| State 2 | State 6 | 0.02406542 | 0.02256067 | 0.02579984 | 4 | Adult (25-44 years) | Physical M -> Physical-Mental MM |
| State 3 | State 6 | 0.10572461 | 0.09889681 | 0.11283638 | 4 | Adult (25-44 years) | Mental M -> Physical-Mental MM |
| State 4 | State 6 | 0.03888403 | 0.03440088 | 0.04379539 | 4 | Adult (25-44 years) | Physical MM -> Physical-Mental MM |
| State 5 | State 6 | 0.12608252 | 0.10788485 | 0.1462492 | 4 | Adult (25-44 years) | Mental MM -> Physical-Mental MM |
| State 6 | State 6 | 0.92378493 | 0.91553436 | 0.93169456 | 4 | Adult (25-44 years) | Physical-Mental MM -> Physical-Mental MM |
| State 1 | State 7 | 0.00268729 | 0.00258463 | 0.00279769 | 4 | Adult (25-44 years) | DF -> Death |
| State 2 | State 7 | 0.01116344 | 0.01029912 | 0.01221376 | 4 | Adult (25-44 years) | Physical M -> Death |
| State 3 | State 7 | 0.02884168 | 0.02523953 | 0.03302157 | 4 | Adult (25-44 years) | Mental M -> Death |
| State 4 | State 7 | 0.05693866 | 0.05199068 | 0.06261944 | 4 | Adult (25-44 years) | Physical MM -> Death |
| State 5 | State 7 | 0.04379013 | 0.03425857 | 0.05598613 | 4 | Adult (25-44 years) | Mental MM -> Death |
| State 6 | State 7 | 0.07621507 | 0.06830544 | 0.08446564 | 4 | Adult (25-44 years) | Physical-Mental MM -> Death |
| State 1 | State 1 | 0.93769729 | 0.93712013 | 0.93825562 | 5 | Adult (25-44 years) | DF -> DF |
| State 1 | State 2 | 0.03919565 | 0.03876218 | 0.03963073 | 5 | Adult (25-44 years) | DF -> Physical M |
| State 2 | State 2 | 0.83104506 | 0.82656982 | 0.83533378 | 5 | Adult (25-44 years) | Physical M -> Physical M |
| State 1 | State 3 | 0.00664168 | 0.00646181 | 0.00682231 | 5 | Adult (25-44 years) | DF -> Mental M |
| State 3 | State 3 | 0.7708758 | 0.75938756 | 0.78175353 | 5 | Adult (25-44 years) | Mental M -> Mental M |
| State 1 | State 4 | 0.00890805 | 0.00871081 | 0.00910465 | 5 | Adult (25-44 years) | DF -> Physical MM |
| State 2 | State 4 | 0.12407409 | 0.12017546 | 0.12805147 | 5 | Adult (25-44 years) | Physical M -> Physical MM |
| State 4 | State 4 | 0.88169222 | 0.87391152 | 0.889657 | 5 | Adult (25-44 years) | Physical MM -> Physical MM |
| State 1 | State 5 | 0.00075951 | 0.00070515 | 0.00081812 | 5 | Adult (25-44 years) | DF -> Mental MM |
| State 3 | State 5 | 0.0635101 | 0.05753349 | 0.07034478 | 5 | Adult (25-44 years) | Mental M -> Mental MM |
| State 5 | State 5 | 0.79237526 | 0.76214495 | 0.81580238 | 5 | Adult (25-44 years) | Mental MM -> Mental MM |
| State 1 | State 6 | 0.00329189 | 0.00317686 | 0.00342488 | 5 | Adult (25-44 years) | DF -> Physical-Mental MM |
| State 2 | State 6 | 0.02991736 | 0.02790638 | 0.03202381 | 5 | Adult (25-44 years) | Physical M -> Physical-Mental MM |
| State 3 | State 6 | 0.12872394 | 0.12076322 | 0.13767499 | 5 | Adult (25-44 years) | Mental M -> Physical-Mental MM |
| State 4 | State 6 | 0.04752411 | 0.04270029 | 0.05338919 | 5 | Adult (25-44 years) | Physical MM -> Physical-Mental MM |
| State 5 | State 6 | 0.15250028 | 0.1308114 | 0.17692492 | 5 | Adult (25-44 years) | Mental MM -> Physical-Mental MM |
| State 6 | State 6 | 0.90565667 | 0.89480953 | 0.91507266 | 5 | Adult (25-44 years) | Physical-Mental MM -> Physical-Mental MM |
| State 1 | State 7 | 0.00350592 | 0.00337805 | 0.00364416 | 5 | Adult (25-44 years) | DF -> Death |
| State 2 | State 7 | 0.01496349 | 0.0137949 | 0.01628326 | 5 | Adult (25-44 years) | Physical M -> Death |
| State 3 | State 7 | 0.03689016 | 0.03272961 | 0.0414705 | 5 | Adult (25-44 years) | Mental M -> Death |
| State 4 | State 7 | 0.07078367 | 0.06496597 | 0.07689915 | 5 | Adult (25-44 years) | Physical MM -> Death |
| State 5 | State 7 | 0.05512446 | 0.04423916 | 0.07059893 | 5 | Adult (25-44 years) | Mental MM -> Death |
| State 6 | State 7 | 0.09434333 | 0.08492734 | 0.10519047 | 5 | Adult (25-44 years) | Physical-Mental MM -> Death |
| State 1 | State 1 | 0.92571051 | 0.92501943 | 0.92641625 | 6 | Adult (25-44 years) | DF -> DF |
| State 1 | State 2 | 0.0458885 | 0.04535306 | 0.04641604 | 6 | Adult (25-44 years) | DF -> Physical M |
| State 2 | State 2 | 0.80084688 | 0.7955886 | 0.80615324 | 6 | Adult (25-44 years) | Physical M -> Physical M |
| State 1 | State 3 | 0.00772092 | 0.0074786 | 0.00794786 | 6 | Adult (25-44 years) | DF -> Mental M |
| State 3 | State 3 | 0.73178128 | 0.71899274 | 0.74401327 | 6 | Adult (25-44 years) | Mental M -> Mental M |
| State 1 | State 4 | 0.01113362 | 0.01088336 | 0.01139186 | 6 | Adult (25-44 years) | DF -> Physical MM |
| State 2 | State 4 | 0.14433922 | 0.13958587 | 0.14903522 | 6 | Adult (25-44 years) | Physical M -> Physical MM |
| State 4 | State 4 | 0.85976628 | 0.84941763 | 0.86882162 | 6 | Adult (25-44 years) | Physical MM -> Physical MM |
| State 1 | State 5 | 0.00094383 | 0.00087667 | 0.00101743 | 6 | Adult (25-44 years) | DF -> Mental MM |
| State 3 | State 5 | 0.07254735 | 0.06595382 | 0.080165 | 6 | Adult (25-44 years) | Mental M -> Mental MM |
| State 5 | State 5 | 0.75634003 | 0.72409968 | 0.78416115 | 6 | Adult (25-44 years) | Mental MM -> Mental MM |
| State 1 | State 6 | 0.00421373 | 0.00404618 | 0.00436981 | 6 | Adult (25-44 years) | DF -> Physical-Mental MM |
| State 2 | State 6 | 0.03568886 | 0.03331438 | 0.03828455 | 6 | Adult (25-44 years) | Physical M -> Physical-Mental MM |
| State 3 | State 6 | 0.15044728 | 0.14139712 | 0.16031485 | 6 | Adult (25-44 years) | Mental M -> Physical-Mental MM |
| State 4 | State 6 | 0.05576081 | 0.04993449 | 0.06261233 | 6 | Adult (25-44 years) | Physical MM -> Physical-Mental MM |
| State 5 | State 6 | 0.17708568 | 0.15228223 | 0.20428746 | 6 | Adult (25-44 years) | Mental MM -> Physical-Mental MM |
| State 6 | State 6 | 0.88788416 | 0.87484979 | 0.89881619 | 6 | Adult (25-44 years) | Physical-Mental MM -> Physical-Mental MM |
| State 1 | State 7 | 0.00438889 | 0.0042241 | 0.00456947 | 6 | Adult (25-44 years) | DF -> Death |
| State 2 | State 7 | 0.01912504 | 0.01772448 | 0.02082316 | 6 | Adult (25-44 years) | Physical M -> Death |
| State 3 | State 7 | 0.04522409 | 0.0405191 | 0.05052173 | 6 | Adult (25-44 years) | Mental M -> Death |
| State 4 | State 7 | 0.08447291 | 0.07725364 | 0.09241054 | 6 | Adult (25-44 years) | Physical MM -> Death |
| State 5 | State 7 | 0.06657429 | 0.05253736 | 0.08642841 | 6 | Adult (25-44 years) | Mental MM -> Death |
| State 6 | State 7 | 0.11211584 | 0.10118381 | 0.12515021 | 6 | Adult (25-44 years) | Physical-Mental MM -> Death |
| State 1 | State 1 | 0.91387696 | 0.9131554 | 0.91466818 | 7 | Adult (25-44 years) | DF -> DF |
| State 1 | State 2 | 0.05223438 | 0.05159494 | 0.05280758 | 7 | Adult (25-44 years) | DF -> Physical M |
| State 2 | State 2 | 0.77174603 | 0.76520362 | 0.77815454 | 7 | Adult (25-44 years) | Physical M -> Physical M |
| State 1 | State 3 | 0.00872733 | 0.00845718 | 0.00899453 | 7 | Adult (25-44 years) | DF -> Mental M |
| State 3 | State 3 | 0.69466941 | 0.68099147 | 0.70747628 | 7 | Adult (25-44 years) | Mental M -> Mental M |
| State 1 | State 4 | 0.01347469 | 0.01318526 | 0.01379098 | 7 | Adult (25-44 years) | DF -> Physical MM |
| State 2 | State 4 | 0.1632519 | 0.15803559 | 0.16862079 | 7 | Adult (25-44 years) | Physical M -> Physical MM |
| State 4 | State 4 | 0.8383856 | 0.82724386 | 0.84923244 | 7 | Adult (25-44 years) | Physical MM -> Physical MM |
| State 1 | State 5 | 0.00113498 | 0.00105681 | 0.00121852 | 7 | Adult (25-44 years) | DF -> Mental MM |
| State 3 | State 5 | 0.08056882 | 0.07331697 | 0.08840785 | 7 | Adult (25-44 years) | Mental M -> Mental MM |
| State 5 | State 5 | 0.7219436 | 0.68654208 | 0.75209961 | 7 | Adult (25-44 years) | Mental MM -> Mental MM |
| State 1 | State 6 | 0.00521311 | 0.00502398 | 0.00539325 | 7 | Adult (25-44 years) | DF -> Physical-Mental MM |
| State 2 | State 6 | 0.04137368 | 0.0387234 | 0.04406516 | 7 | Adult (25-44 years) | Physical M -> Physical-Mental MM |
| State 3 | State 6 | 0.17094116 | 0.16056331 | 0.18110168 | 7 | Adult (25-44 years) | Mental M -> Physical-Mental MM |
| State 4 | State 6 | 0.06360784 | 0.05653886 | 0.07126182 | 7 | Adult (25-44 years) | Physical MM -> Physical-Mental MM |
| State 5 | State 6 | 0.19993444 | 0.17351336 | 0.23045758 | 7 | Adult (25-44 years) | Mental MM -> Physical-Mental MM |
| State 6 | State 6 | 0.87046041 | 0.85612617 | 0.88309993 | 7 | Adult (25-44 years) | Physical-Mental MM -> Physical-Mental MM |
| State 1 | State 7 | 0.00533856 | 0.00514118 | 0.0055507 | 7 | Adult (25-44 years) | DF -> Death |
| State 2 | State 7 | 0.02362839 | 0.02189107 | 0.02545746 | 7 | Adult (25-44 years) | Physical M -> Death |
| State 3 | State 7 | 0.0538206 | 0.04820462 | 0.0609589 | 7 | Adult (25-44 years) | Mental M -> Death |
| State 4 | State 7 | 0.09800656 | 0.08950457 | 0.10664353 | 7 | Adult (25-44 years) | Physical MM -> Death |
| State 5 | State 7 | 0.07812196 | 0.06351627 | 0.10007621 | 7 | Adult (25-44 years) | Mental MM -> Death |
| State 6 | State 7 | 0.12953959 | 0.11690007 | 0.14387383 | 7 | Adult (25-44 years) | Physical-Mental MM -> Death |
| State 1 | State 1 | 0.90219468 | 0.90127188 | 0.90307048 | 8 | Adult (25-44 years) | DF -> DF |
| State 1 | State 2 | 0.05824724 | 0.05756824 | 0.05897171 | 8 | Adult (25-44 years) | DF -> Physical M |
| State 2 | State 2 | 0.74370263 | 0.736775 | 0.74956454 | 8 | Adult (25-44 years) | Physical M -> Physical M |
| State 1 | State 3 | 0.00966482 | 0.00937944 | 0.00996766 | 8 | Adult (25-44 years) | DF -> Mental M |
| State 3 | State 3 | 0.65943966 | 0.64405342 | 0.67390014 | 8 | Adult (25-44 years) | Mental M -> Mental M |
| State 1 | State 4 | 0.01591887 | 0.01558289 | 0.01629364 | 8 | Adult (25-44 years) | DF -> Physical MM |
| State 2 | State 4 | 0.18087658 | 0.17573857 | 0.18634984 | 8 | Adult (25-44 years) | Physical M -> Physical MM |
| State 4 | State 4 | 0.81753661 | 0.80520618 | 0.82856656 | 8 | Adult (25-44 years) | Physical MM -> Physical MM |
| State 1 | State 5 | 0.00133154 | 0.00123775 | 0.00143849 | 8 | Adult (25-44 years) | DF -> Mental MM |
| State 3 | State 5 | 0.08765137 | 0.07984765 | 0.09562008 | 8 | Adult (25-44 years) | Mental M -> Mental MM |
| State 5 | State 5 | 0.68911142 | 0.64806609 | 0.72249365 | 8 | Adult (25-44 years) | Mental MM -> Mental MM |
| State 1 | State 6 | 0.00628581 | 0.00605964 | 0.00651972 | 8 | Adult (25-44 years) | DF -> Physical-Mental MM |
| State 2 | State 6 | 0.04696616 | 0.0441272 | 0.05000924 | 8 | Adult (25-44 years) | Physical M -> Physical-Mental MM |
| State 3 | State 6 | 0.19025105 | 0.17906519 | 0.20241033 | 8 | Adult (25-44 years) | Mental M -> Physical-Mental MM |
| State 4 | State 6 | 0.07107854 | 0.06350867 | 0.07920765 | 8 | Adult (25-44 years) | Physical MM -> Physical-Mental MM |
| State 5 | State 6 | 0.22113768 | 0.19095751 | 0.25445315 | 8 | Adult (25-44 years) | Mental MM -> Physical-Mental MM |
| State 6 | State 6 | 0.85337859 | 0.83700826 | 0.86809641 | 8 | Adult (25-44 years) | Physical-Mental MM -> Physical-Mental MM |
| State 1 | State 7 | 0.00635705 | 0.00613028 | 0.00660368 | 8 | Adult (25-44 years) | DF -> Death |
| State 2 | State 7 | 0.02845463 | 0.02644696 | 0.03093607 | 8 | Adult (25-44 years) | Physical M -> Death |
| State 3 | State 7 | 0.06265793 | 0.05657746 | 0.06991159 | 8 | Adult (25-44 years) | Mental M -> Death |
| State 4 | State 7 | 0.11138485 | 0.10218931 | 0.12104356 | 8 | Adult (25-44 years) | Physical MM -> Death |
| State 5 | State 7 | 0.08975089 | 0.07272071 | 0.11140484 | 8 | Adult (25-44 years) | Mental MM -> Death |
| State 6 | State 7 | 0.14662141 | 0.13190359 | 0.16299174 | 8 | Adult (25-44 years) | Physical-Mental MM -> Death |
| State 1 | State 1 | 0.89066173 | 0.88967081 | 0.89162104 | 9 | Adult (25-44 years) | DF -> DF |
| State 1 | State 2 | 0.06394048 | 0.06314643 | 0.06462412 | 9 | Adult (25-44 years) | DF -> Physical M |
| State 2 | State 2 | 0.71667826 | 0.7094294 | 0.72357178 | 9 | Adult (25-44 years) | Physical M -> Physical M |
| State 1 | State 3 | 0.01053713 | 0.01022973 | 0.01086449 | 9 | Adult (25-44 years) | DF -> Mental M |
| State 3 | State 3 | 0.62599655 | 0.60983475 | 0.64106679 | 9 | Adult (25-44 years) | Mental M -> Mental M |
| State 1 | State 4 | 0.01845444 | 0.01802506 | 0.01887725 | 9 | Adult (25-44 years) | DF -> Physical MM |
| State 2 | State 4 | 0.19727501 | 0.19092712 | 0.20332921 | 9 | Adult (25-44 years) | Physical M -> Physical MM |
| State 4 | State 4 | 0.7972061 | 0.7827525 | 0.8101504 | 9 | Adult (25-44 years) | Physical MM -> Physical MM |
| State 1 | State 5 | 0.00153221 | 0.00142177 | 0.00165263 | 9 | Adult (25-44 years) | DF -> Mental MM |
| State 3 | State 5 | 0.09386681 | 0.08533258 | 0.10329859 | 9 | Adult (25-44 years) | Mental M -> Mental MM |
| State 5 | State 5 | 0.65777238 | 0.6174292 | 0.69424894 | 9 | Adult (25-44 years) | Mental MM -> Mental MM |
| State 1 | State 6 | 0.00742773 | 0.00715912 | 0.00769962 | 9 | Adult (25-44 years) | DF -> Physical-Mental MM |
| State 2 | State 6 | 0.05246115 | 0.04899771 | 0.05611289 | 9 | Adult (25-44 years) | Physical M -> Physical-Mental MM |
| State 3 | State 6 | 0.2084213 | 0.19591669 | 0.22074412 | 9 | Adult (25-44 years) | Mental M -> Physical-Mental MM |
| State 4 | State 6 | 0.07818581 | 0.06872911 | 0.08811739 | 9 | Adult (25-44 years) | Physical MM -> Physical-Mental MM |
| State 5 | State 6 | 0.24078213 | 0.20844569 | 0.2732796 | 9 | Adult (25-44 years) | Mental MM -> Physical-Mental MM |
| State 6 | State 6 | 0.83663198 | 0.81870074 | 0.8523094 | 9 | Adult (25-44 years) | Physical-Mental MM -> Physical-Mental MM |
| State 1 | State 7 | 0.00744627 | 0.00720349 | 0.00773053 | 9 | Adult (25-44 years) | DF -> Death |
| State 2 | State 7 | 0.03358558 | 0.03122444 | 0.03608735 | 9 | Adult (25-44 years) | Physical M -> Death |
| State 3 | State 7 | 0.07171534 | 0.06520276 | 0.08024779 | 9 | Adult (25-44 years) | Mental M -> Death |
| State 4 | State 7 | 0.12460809 | 0.11475989 | 0.1351596 | 9 | Adult (25-44 years) | Physical MM -> Death |
| State 5 | State 7 | 0.1014455 | 0.08175229 | 0.12773134 | 9 | Adult (25-44 years) | Mental MM -> Death |
| State 6 | State 7 | 0.16336802 | 0.1476906 | 0.18129926 | 9 | Adult (25-44 years) | Physical-Mental MM -> Death |
| State 1 | State 1 | 0.87927622 | 0.8782038 | 0.88033934 | 10 | Adult (25-44 years) | DF -> DF |
| State 1 | State 2 | 0.069327 | 0.06848299 | 0.07010158 | 10 | Adult (25-44 years) | DF -> Physical M |
| State 2 | State 2 | 0.6906359 | 0.68196545 | 0.69814795 | 10 | Adult (25-44 years) | Physical M -> Physical M |
| State 1 | State 3 | 0.01134779 | 0.01098046 | 0.01172251 | 10 | Adult (25-44 years) | DF -> Mental M |
| State 3 | State 3 | 0.5942495 | 0.57706534 | 0.61229071 | 10 | Adult (25-44 years) | Mental M -> Mental M |
| State 1 | State 4 | 0.02107038 | 0.02056648 | 0.02157303 | 10 | Adult (25-44 years) | DF -> Physical MM |
| State 2 | State 4 | 0.21250632 | 0.20561271 | 0.21988546 | 10 | Adult (25-44 years) | Physical M -> Physical MM |
| State 4 | State 4 | 0.77738117 | 0.76205355 | 0.79112691 | 10 | Adult (25-44 years) | Physical MM -> Physical MM |
| State 1 | State 5 | 0.00173583 | 0.00160884 | 0.00186887 | 10 | Adult (25-44 years) | DF -> Mental MM |
| State 3 | State 5 | 0.09928222 | 0.08950808 | 0.10851532 | 10 | Adult (25-44 years) | Mental M -> Mental MM |
| State 5 | State 5 | 0.62785855 | 0.5828814 | 0.6663331 | 10 | Adult (25-44 years) | Mental MM -> Mental MM |
| State 1 | State 6 | 0.00863487 | 0.00830001 | 0.0089684 | 10 | Adult (25-44 years) | DF -> Physical-Mental MM |
| State 2 | State 6 | 0.05785403 | 0.05388813 | 0.06185301 | 10 | Adult (25-44 years) | Physical M -> Physical-Mental MM |
| State 3 | State 6 | 0.22549518 | 0.21264139 | 0.24011181 | 10 | Adult (25-44 years) | Mental M -> Physical-Mental MM |
| State 4 | State 6 | 0.08494217 | 0.07577705 | 0.09603585 | 10 | Adult (25-44 years) | Physical MM -> Physical-Mental MM |
| State 5 | State 6 | 0.25895034 | 0.22536734 | 0.2942704 | 10 | Adult (25-44 years) | Mental MM -> Physical-Mental MM |
| State 6 | State 6 | 0.820214 | 0.80107299 | 0.8370368 | 10 | Adult (25-44 years) | Physical-Mental MM -> Physical-Mental MM |
| State 1 | State 7 | 0.00860792 | 0.00827721 | 0.00892496 | 10 | Adult (25-44 years) | DF -> Death |
| State 2 | State 7 | 0.03900375 | 0.03647726 | 0.04189109 | 10 | Adult (25-44 years) | Physical M -> Death |
| State 3 | State 7 | 0.0809731 | 0.07297062 | 0.09072074 | 10 | Adult (25-44 years) | Mental M -> Death |
| State 4 | State 7 | 0.13767666 | 0.12702425 | 0.14974095 | 10 | Adult (25-44 years) | Physical MM -> Death |
| State 5 | State 7 | 0.11319111 | 0.09212373 | 0.13988452 | 10 | Adult (25-44 years) | Mental MM -> Death |
| State 6 | State 7 | 0.179786 | 0.1629632 | 0.19892701 | 10 | Adult (25-44 years) | Physical-Mental MM -> Death |
| State 1 | State 1 | 0.86803624 | 0.86693492 | 0.86914772 | 11 | Adult (25-44 years) | DF -> DF |
| State 1 | State 2 | 0.07441923 | 0.07351879 | 0.07529562 | 11 | Adult (25-44 years) | DF -> Physical M |
| State 2 | State 2 | 0.66553985 | 0.65691454 | 0.67346988 | 11 | Adult (25-44 years) | Physical M -> Physical M |
| State 1 | State 3 | 0.01210014 | 0.01171594 | 0.01249629 | 11 | Adult (25-44 years) | DF -> Mental M |
| State 3 | State 3 | 0.56411248 | 0.5474171 | 0.58170292 | 11 | Adult (25-44 years) | Mental M -> Mental M |
| State 1 | State 4 | 0.02375628 | 0.02324525 | 0.02431371 | 11 | Adult (25-44 years) | DF -> Physical MM |
| State 2 | State 4 | 0.22662711 | 0.21978676 | 0.23368333 | 11 | Adult (25-44 years) | Physical M -> Physical MM |
| State 4 | State 4 | 0.75804924 | 0.74184159 | 0.77178348 | 11 | Adult (25-44 years) | Physical MM -> Physical MM |
| State 1 | State 5 | 0.00194131 | 0.001787 | 0.0021005 | 11 | Adult (25-44 years) | DF -> Mental MM |
| State 3 | State 5 | 0.10396023 | 0.09379935 | 0.11424965 | 11 | Adult (25-44 years) | Mental M -> Mental MM |
| State 5 | State 5 | 0.59930513 | 0.55440051 | 0.64175644 | 11 | Adult (25-44 years) | Mental MM -> Mental MM |
| State 1 | State 6 | 0.00990331 | 0.00954954 | 0.01029301 | 11 | Adult (25-44 years) | DF -> Physical-Mental MM |
| State 2 | State 6 | 0.06314067 | 0.05906525 | 0.06734246 | 11 | Adult (25-44 years) | Physical M -> Physical-Mental MM |
| State 3 | State 6 | 0.24151484 | 0.22658036 | 0.25566869 | 11 | Adult (25-44 years) | Mental M -> Physical-Mental MM |
| State 4 | State 6 | 0.09135978 | 0.08220469 | 0.10130194 | 11 | Adult (25-44 years) | Physical MM -> Physical-Mental MM |
| State 5 | State 6 | 0.2757209 | 0.24201245 | 0.31519879 | 11 | Adult (25-44 years) | Mental MM -> Physical-Mental MM |
| State 6 | State 6 | 0.80411821 | 0.78347005 | 0.8227679 | 11 | Adult (25-44 years) | Physical-Mental MM -> Physical-Mental MM |
| State 1 | State 7 | 0.00984348 | 0.00947888 | 0.01022158 | 11 | Adult (25-44 years) | DF -> Death |
| State 2 | State 7 | 0.04469237 | 0.0416548 | 0.04768692 | 11 | Adult (25-44 years) | Physical M -> Death |
| State 3 | State 7 | 0.09041245 | 0.08146795 | 0.09994914 | 11 | Adult (25-44 years) | Mental M -> Death |
| State 4 | State 7 | 0.15059098 | 0.13920655 | 0.16251568 | 11 | Adult (25-44 years) | Physical MM -> Death |
| State 5 | State 7 | 0.12497398 | 0.10200105 | 0.15145309 | 11 | Adult (25-44 years) | Mental MM -> Death |
| State 6 | State 7 | 0.19588179 | 0.1772321 | 0.21652995 | 11 | Adult (25-44 years) | Physical-Mental MM -> Death |
| State 1 | State 1 | 0.85693995 | 0.85563094 | 0.85812285 | 12 | Adult (25-44 years) | DF -> DF |
| State 1 | State 2 | 0.07922912 | 0.07827667 | 0.08018097 | 12 | Adult (25-44 years) | DF -> Physical M |
| State 2 | State 2 | 0.64135573 | 0.63306302 | 0.64991805 | 12 | Adult (25-44 years) | Physical M -> Physical M |
| State 1 | State 3 | 0.01279736 | 0.01237026 | 0.01321885 | 12 | Adult (25-44 years) | DF -> Mental M |
| State 3 | State 3 | 0.53550384 | 0.51662284 | 0.5549408 | 12 | Adult (25-44 years) | Mental M -> Mental M |
| State 1 | State 4 | 0.02650233 | 0.02590665 | 0.02713686 | 12 | Adult (25-44 years) | DF -> Physical MM |
| State 2 | State 4 | 0.23969161 | 0.23200801 | 0.2470942 | 12 | Adult (25-44 years) | Physical M -> Physical MM |
| State 4 | State 4 | 0.73919806 | 0.72225592 | 0.75391589 | 12 | Adult (25-44 years) | Physical MM -> Physical MM |
| State 1 | State 5 | 0.0021477 | 0.00198247 | 0.0023087 | 12 | Adult (25-44 years) | DF -> Mental MM |
| State 3 | State 5 | 0.10795926 | 0.09691612 | 0.11871516 | 12 | Adult (25-44 years) | Mental M -> Mental MM |
| State 5 | State 5 | 0.57205024 | 0.52229125 | 0.61436084 | 12 | Adult (25-44 years) | Mental MM -> Mental MM |
| State 1 | State 6 | 0.01122926 | 0.01083015 | 0.01164117 | 12 | Adult (25-44 years) | DF -> Physical-Mental MM |
| State 2 | State 6 | 0.06831735 | 0.0639716 | 0.07273324 | 12 | Adult (25-44 years) | Physical M -> Physical-Mental MM |
| State 3 | State 6 | 0.25652136 | 0.24228158 | 0.27224732 | 12 | Adult (25-44 years) | Mental M -> Physical-Mental MM |
| State 4 | State 6 | 0.0974504 | 0.0874573 | 0.1092206 | 12 | Adult (25-44 years) | Physical MM -> Physical-Mental MM |
| State 5 | State 6 | 0.29116856 | 0.25603692 | 0.32958086 | 12 | Adult (25-44 years) | Mental MM -> Physical-Mental MM |
| State 6 | State 6 | 0.78833828 | 0.76720156 | 0.81043473 | 12 | Adult (25-44 years) | Physical-Mental MM -> Physical-Mental MM |
| State 1 | State 7 | 0.01115428 | 0.01075755 | 0.01158704 | 12 | Adult (25-44 years) | DF -> Death |
| State 2 | State 7 | 0.05063531 | 0.04746351 | 0.05448791 | 12 | Adult (25-44 years) | Physical M -> Death |
| State 3 | State 7 | 0.10001554 | 0.09116923 | 0.10941246 | 12 | Adult (25-44 years) | Mental M -> Death |
| State 4 | State 7 | 0.16335154 | 0.15039617 | 0.17685534 | 12 | Adult (25-44 years) | Physical MM -> Death |
| State 5 | State 7 | 0.1367812 | 0.11232018 | 0.17006774 | 12 | Adult (25-44 years) | Mental MM -> Death |
| State 6 | State 7 | 0.21166172 | 0.18956527 | 0.23279844 | 12 | Adult (25-44 years) | Physical-Mental MM -> Death |
| State 1 | State 1 | 0.84598551 | 0.84463673 | 0.84735951 | 13 | Adult (25-44 years) | DF -> DF |
| State 1 | State 2 | 0.08376818 | 0.08264178 | 0.08486767 | 13 | Adult (25-44 years) | DF -> Physical M |
| State 2 | State 2 | 0.6180504 | 0.60894754 | 0.62624262 | 13 | Adult (25-44 years) | Physical M -> Physical M |
| State 1 | State 3 | 0.01344246 | 0.0130117 | 0.0138663 | 13 | Adult (25-44 years) | DF -> Mental M |
| State 3 | State 3 | 0.50834607 | 0.48973517 | 0.52615315 | 13 | Adult (25-44 years) | Mental M -> Mental M |
| State 1 | State 4 | 0.02929931 | 0.0286175 | 0.02991562 | 13 | Adult (25-44 years) | DF -> Physical MM |
| State 2 | State 4 | 0.2517517 | 0.24402798 | 0.25942581 | 13 | Adult (25-44 years) | Physical M -> Physical MM |
| State 4 | State 4 | 0.72081567 | 0.70281375 | 0.73731878 | 13 | Adult (25-44 years) | Physical MM -> Physical MM |
| State 1 | State 5 | 0.00235412 | 0.00217635 | 0.002548 | 13 | Adult (25-44 years) | DF -> Mental MM |
| State 3 | State 5 | 0.11133385 | 0.10083793 | 0.12318673 | 13 | Adult (25-44 years) | Mental M -> Mental MM |
| State 5 | State 5 | 0.54603484 | 0.49895794 | 0.59097471 | 13 | Adult (25-44 years) | Mental MM -> Mental MM |
| State 1 | State 6 | 0.012609 | 0.01214892 | 0.0131035 | 13 | Adult (25-44 years) | DF -> Physical-Mental MM |
| State 2 | State 6 | 0.07338083 | 0.06915357 | 0.07824851 | 13 | Adult (25-44 years) | Physical M -> Physical-Mental MM |
| State 3 | State 6 | 0.27055466 | 0.25579618 | 0.28525463 | 13 | Adult (25-44 years) | Mental M -> Physical-Mental MM |
| State 4 | State 6 | 0.10322546 | 0.092947 | 0.11608441 | 13 | Adult (25-44 years) | Physical MM -> Physical-Mental MM |
| State 5 | State 6 | 0.3053645 | 0.26865355 | 0.34383008 | 13 | Adult (25-44 years) | Mental MM -> Physical-Mental MM |
| State 6 | State 6 | 0.77286801 | 0.74887721 | 0.7956488 | 13 | Adult (25-44 years) | Physical-Mental MM -> Physical-Mental MM |
| State 1 | State 7 | 0.01254141 | 0.01208685 | 0.01303257 | 13 | Adult (25-44 years) | DF -> Death |
| State 2 | State 7 | 0.05681707 | 0.05304512 | 0.06090183 | 13 | Adult (25-44 years) | Physical M -> Death |
| State 3 | State 7 | 0.10976542 | 0.10013257 | 0.12086515 | 13 | Adult (25-44 years) | Mental M -> Death |
| State 4 | State 7 | 0.17595887 | 0.1623376 | 0.18990665 | 13 | Adult (25-44 years) | Physical MM -> Death |
| State 5 | State 7 | 0.14860066 | 0.12473753 | 0.17973352 | 13 | Adult (25-44 years) | Mental MM -> Death |
| State 6 | State 7 | 0.22713199 | 0.2043512 | 0.25112279 | 13 | Adult (25-44 years) | Physical-Mental MM -> Death |
| State 1 | State 1 | 0.8351711 | 0.83380803 | 0.83666777 | 14 | Adult (25-44 years) | DF -> DF |
| State 1 | State 2 | 0.08804748 | 0.08696946 | 0.08917309 | 14 | Adult (25-44 years) | DF -> Physical M |
| State 2 | State 2 | 0.59559193 | 0.58534946 | 0.60527761 | 14 | Adult (25-44 years) | Physical M -> Physical M |
| State 1 | State 3 | 0.01403831 | 0.0135976 | 0.01453643 | 14 | Adult (25-44 years) | DF -> Mental M |
| State 3 | State 3 | 0.4825656 | 0.46427716 | 0.5013956 | 14 | Adult (25-44 years) | Mental M -> Mental M |
| State 1 | State 4 | 0.03213856 | 0.03138192 | 0.03284812 | 14 | Adult (25-44 years) | DF -> Physical MM |
| State 2 | State 4 | 0.26285704 | 0.25427381 | 0.27056344 | 14 | Adult (25-44 years) | Physical M -> Physical MM |
| State 4 | State 4 | 0.70289041 | 0.68411614 | 0.72106867 | 14 | Adult (25-44 years) | Physical MM -> Physical MM |
| State 1 | State 5 | 0.00255977 | 0.0023742 | 0.00275619 | 14 | Adult (25-44 years) | DF -> Mental MM |
| State 3 | State 5 | 0.11413484 | 0.10285549 | 0.12515866 | 14 | Adult (25-44 years) | Mental M -> Mental MM |
| State 5 | State 5 | 0.52120255 | 0.46797672 | 0.5657683 | 14 | Adult (25-44 years) | Mental MM -> Mental MM |
| State 1 | State 6 | 0.01403895 | 0.01353781 | 0.01456888 | 14 | Adult (25-44 years) | DF -> Physical-Mental MM |
| State 2 | State 6 | 0.07832823 | 0.07332739 | 0.08348899 | 14 | Adult (25-44 years) | Physical M -> Physical-Mental MM |
| State 3 | State 6 | 0.2836536 | 0.26756184 | 0.2987708 | 14 | Adult (25-44 years) | Mental M -> Physical-Mental MM |
| State 4 | State 6 | 0.10869601 | 0.09760493 | 0.12095025 | 14 | Adult (25-44 years) | Physical MM -> Physical-Mental MM |
| State 5 | State 6 | 0.31837641 | 0.27948319 | 0.35964361 | 14 | Adult (25-44 years) | Mental MM -> Physical-Mental MM |
| State 6 | State 6 | 0.75770133 | 0.73215026 | 0.77790735 | 14 | Adult (25-44 years) | Physical-Mental MM -> Physical-Mental MM |
| State 1 | State 7 | 0.01400584 | 0.01349418 | 0.01455955 | 14 | Adult (25-44 years) | DF -> Death |
| State 2 | State 7 | 0.0632228 | 0.05908289 | 0.06763595 | 14 | Adult (25-44 years) | Physical M -> Death |
| State 3 | State 7 | 0.11964597 | 0.10924832 | 0.13199046 | 14 | Adult (25-44 years) | Mental M -> Death |
| State 4 | State 7 | 0.18841357 | 0.17472628 | 0.20389165 | 14 | Adult (25-44 years) | Physical MM -> Death |
| State 5 | State 7 | 0.16042103 | 0.13519203 | 0.19322278 | 14 | Adult (25-44 years) | Mental MM -> Death |
| State 6 | State 7 | 0.24229867 | 0.22209265 | 0.26784974 | 14 | Adult (25-44 years) | Physical-Mental MM -> Death |
| State 1 | State 1 | 0.82449493 | 0.82296315 | 0.82588763 | 15 | Adult (25-44 years) | DF -> DF |
| State 1 | State 2 | 0.09207766 | 0.09090939 | 0.09327854 | 15 | Adult (25-44 years) | DF -> Physical M |
| State 2 | State 2 | 0.57394955 | 0.56323171 | 0.5838451 | 15 | Adult (25-44 years) | Physical M -> Physical M |
| State 1 | State 3 | 0.0145876 | 0.01406811 | 0.01506839 | 15 | Adult (25-44 years) | DF -> Mental M |
| State 3 | State 3 | 0.45809256 | 0.43639551 | 0.47755394 | 15 | Adult (25-44 years) | Mental M -> Mental M |
| State 1 | State 4 | 0.03501192 | 0.03406647 | 0.03585658 | 15 | Adult (25-44 years) | DF -> Physical MM |
| State 2 | State 4 | 0.27305518 | 0.26440117 | 0.28097924 | 15 | Adult (25-44 years) | Physical M -> Physical MM |
| State 4 | State 4 | 0.68541093 | 0.66485711 | 0.70348752 | 15 | Adult (25-44 years) | Physical MM -> Physical MM |
| State 1 | State 5 | 0.00276395 | 0.00253764 | 0.00299862 | 15 | Adult (25-44 years) | DF -> Mental MM |
| State 3 | State 5 | 0.11640962 | 0.10457752 | 0.12945591 | 15 | Adult (25-44 years) | Mental M -> Mental MM |
| State 5 | State 5 | 0.49749958 | 0.44160072 | 0.54147149 | 15 | Adult (25-44 years) | Mental MM -> Mental MM |
| State 1 | State 6 | 0.01551559 | 0.01497671 | 0.01612354 | 15 | Adult (25-44 years) | DF -> Physical-Mental MM |
| State 2 | State 6 | 0.08315707 | 0.07801009 | 0.08884956 | 15 | Adult (25-44 years) | Physical M -> Physical-Mental MM |
| State 3 | State 6 | 0.29585591 | 0.28018812 | 0.31202969 | 15 | Adult (25-44 years) | Mental M -> Physical-Mental MM |
| State 4 | State 6 | 0.11387279 | 0.10187654 | 0.12725982 | 15 | Adult (25-44 years) | Physical MM -> Physical-Mental MM |
| State 5 | State 6 | 0.33026871 | 0.29022213 | 0.37521372 | 15 | Adult (25-44 years) | Mental MM -> Physical-Mental MM |
| State 6 | State 6 | 0.74283228 | 0.71696228 | 0.76691806 | 15 | Adult (25-44 years) | Physical-Mental MM -> Physical-Mental MM |
| State 1 | State 7 | 0.01554836 | 0.01502102 | 0.01618596 | 15 | Adult (25-44 years) | DF -> Death |
| State 2 | State 7 | 0.0698382 | 0.06564679 | 0.07438958 | 15 | Adult (25-44 years) | Physical M -> Death |
| State 3 | State 7 | 0.12964191 | 0.11795345 | 0.1422628 | 15 | Adult (25-44 years) | Mental M -> Death |
| State 4 | State 7 | 0.20071628 | 0.18715819 | 0.21715299 | 15 | Adult (25-44 years) | Physical MM -> Death |
| State 5 | State 7 | 0.17223171 | 0.14607058 | 0.20952234 | 15 | Adult (25-44 years) | Mental MM -> Death |
| State 6 | State 7 | 0.25716772 | 0.23308194 | 0.28303772 | 15 | Adult (25-44 years) | Physical-Mental MM -> Death |
| State 1 | State 1 | 0.81395524 | 0.81243841 | 0.81545105 | 16 | Adult (25-44 years) | DF -> DF |
| State 1 | State 2 | 0.09586898 | 0.09460948 | 0.09718333 | 16 | Adult (25-44 years) | DF -> Physical M |
| State 2 | State 2 | 0.5530936 | 0.5424314 | 0.56315414 | 16 | Adult (25-44 years) | Physical M -> Physical M |
| State 1 | State 3 | 0.01509292 | 0.01454385 | 0.01560027 | 16 | Adult (25-44 years) | DF -> Mental M |
| State 3 | State 3 | 0.43486066 | 0.41392524 | 0.45640327 | 16 | Adult (25-44 years) | Mental M -> Mental M |
| State 1 | State 4 | 0.03791174 | 0.03696896 | 0.03890623 | 16 | Adult (25-44 years) | DF -> Physical MM |
| State 2 | State 4 | 0.28239161 | 0.27357606 | 0.29248912 | 16 | Adult (25-44 years) | Physical M -> Physical MM |
| State 4 | State 4 | 0.66836612 | 0.64719467 | 0.68779599 | 16 | Adult (25-44 years) | Physical MM -> Physical MM |
| State 1 | State 5 | 0.00296602 | 0.00271047 | 0.00318494 | 16 | Adult (25-44 years) | DF -> Mental MM |
| State 3 | State 5 | 0.11820235 | 0.10620051 | 0.13008981 | 16 | Adult (25-44 years) | Mental M -> Mental MM |
| State 5 | State 5 | 0.47487455 | 0.42232878 | 0.52098587 | 16 | Adult (25-44 years) | Mental MM -> Mental MM |
| State 1 | State 6 | 0.01703554 | 0.01640048 | 0.01772749 | 16 | Adult (25-44 years) | DF -> Physical-Mental MM |
| State 2 | State 6 | 0.08786521 | 0.08247939 | 0.09365011 | 16 | Adult (25-44 years) | Physical M -> Physical-Mental MM |
| State 3 | State 6 | 0.30719828 | 0.28948561 | 0.32431461 | 16 | Adult (25-44 years) | Mental M -> Physical-Mental MM |
| State 4 | State 6 | 0.11876621 | 0.10691476 | 0.13333046 | 16 | Adult (25-44 years) | Physical MM -> Physical-Mental MM |
| State 5 | State 6 | 0.34110267 | 0.30464606 | 0.38381632 | 16 | Adult (25-44 years) | Mental MM -> Physical-Mental MM |
| State 6 | State 6 | 0.72825501 | 0.70133703 | 0.75479753 | 16 | Adult (25-44 years) | Physical-Mental MM -> Physical-Mental MM |
| State 1 | State 7 | 0.01716958 | 0.01656468 | 0.01782586 | 16 | Adult (25-44 years) | DF -> Death |
| State 2 | State 7 | 0.07664957 | 0.07216018 | 0.08176732 | 16 | Adult (25-44 years) | Physical M -> Death |
| State 3 | State 7 | 0.13973872 | 0.12809033 | 0.15299844 | 16 | Adult (25-44 years) | Mental M -> Death |
| State 4 | State 7 | 0.21286767 | 0.19791069 | 0.23128523 | 16 | Adult (25-44 years) | Physical MM -> Death |
| State 5 | State 7 | 0.18402278 | 0.15561989 | 0.22051017 | 16 | Adult (25-44 years) | Mental MM -> Death |
| State 6 | State 7 | 0.27174499 | 0.24520247 | 0.29866297 | 16 | Adult (25-44 years) | Physical-Mental MM -> Death |
| State 1 | State 1 | 0.80355027 | 0.80197532 | 0.80516877 | 17 | Adult (25-44 years) | DF -> DF |
| State 1 | State 2 | 0.09943129 | 0.09815184 | 0.10083885 | 17 | Adult (25-44 years) | DF -> Physical M |
| State 2 | State 2 | 0.53299551 | 0.52245632 | 0.54299678 | 17 | Adult (25-44 years) | Physical M -> Physical M |
| State 1 | State 3 | 0.01555669 | 0.01498709 | 0.01605289 | 17 | Adult (25-44 years) | DF -> Mental M |
| State 3 | State 3 | 0.41280695 | 0.39187121 | 0.43192174 | 17 | Adult (25-44 years) | Mental M -> Mental M |
| State 1 | State 4 | 0.04083084 | 0.03982155 | 0.0418305 | 17 | Adult (25-44 years) | DF -> Physical MM |
| State 2 | State 4 | 0.29090986 | 0.28216993 | 0.30014053 | 17 | Adult (25-44 years) | Physical M -> Physical MM |
| State 4 | State 4 | 0.65174518 | 0.63049467 | 0.67077178 | 17 | Adult (25-44 years) | Physical MM -> Physical MM |
| State 1 | State 5 | 0.00316541 | 0.00292809 | 0.00345142 | 17 | Adult (25-44 years) | DF -> Mental MM |
| State 3 | State 5 | 0.11955415 | 0.10814892 | 0.13374719 | 17 | Adult (25-44 years) | Mental M -> Mental MM |
| State 5 | State 5 | 0.45327846 | 0.40133888 | 0.50665481 | 17 | Adult (25-44 years) | Mental MM -> Mental MM |
| State 1 | State 6 | 0.01859548 | 0.0178854 | 0.01933714 | 17 | Adult (25-44 years) | DF -> Physical-Mental MM |
| State 2 | State 6 | 0.09245086 | 0.08683806 | 0.0982266 | 17 | Adult (25-44 years) | Physical M -> Physical-Mental MM |
| State 3 | State 6 | 0.31771626 | 0.30163152 | 0.33542006 | 17 | Adult (25-44 years) | Mental M -> Physical-Mental MM |
| State 4 | State 6 | 0.12338634 | 0.1104325 | 0.13800113 | 17 | Adult (25-44 years) | Physical MM -> Physical-Mental MM |
| State 5 | State 6 | 0.35093657 | 0.30971335 | 0.39088947 | 17 | Adult (25-44 years) | Mental MM -> Physical-Mental MM |
| State 6 | State 6 | 0.71396382 | 0.68796808 | 0.74072669 | 17 | Adult (25-44 years) | Physical-Mental MM -> Physical-Mental MM |
| State 1 | State 7 | 0.01887001 | 0.01818705 | 0.01961536 | 17 | Adult (25-44 years) | DF -> Death |
| State 2 | State 7 | 0.08364377 | 0.0782867 | 0.08927343 | 17 | Adult (25-44 years) | Physical M -> Death |
| State 3 | State 7 | 0.14992264 | 0.13758046 | 0.16443282 | 17 | Adult (25-44 years) | Mental M -> Death |
| State 4 | State 7 | 0.22486849 | 0.20935396 | 0.24153988 | 17 | Adult (25-44 years) | Physical MM -> Death |
| State 5 | State 7 | 0.19578497 | 0.1635985 | 0.23172177 | 17 | Adult (25-44 years) | Mental MM -> Death |
| State 6 | State 7 | 0.28603618 | 0.25927331 | 0.31203192 | 17 | Adult (25-44 years) | Physical-Mental MM -> Death |
| State 1 | State 1 | 0.79327832 | 0.79144061 | 0.79497048 | 18 | Adult (25-44 years) | DF -> DF |
| State 1 | State 2 | 0.10277408 | 0.10130131 | 0.10431124 | 18 | Adult (25-44 years) | DF -> Physical M |
| State 2 | State 2 | 0.51362773 | 0.50331729 | 0.52332915 | 18 | Adult (25-44 years) | Physical M -> Physical M |
| State 1 | State 3 | 0.01598123 | 0.01544374 | 0.01655224 | 18 | Adult (25-44 years) | DF -> Mental M |
| State 3 | State 3 | 0.39187169 | 0.37280541 | 0.41060769 | 18 | Adult (25-44 years) | Mental M -> Mental M |
| State 1 | State 4 | 0.04376252 | 0.04268042 | 0.04488795 | 18 | Adult (25-44 years) | DF -> Physical MM |
| State 2 | State 4 | 0.29865155 | 0.28915582 | 0.30813495 | 18 | Adult (25-44 years) | Physical M -> Physical MM |
| State 4 | State 4 | 0.63553757 | 0.61307363 | 0.65672317 | 18 | Adult (25-44 years) | Physical MM -> Physical MM |
| State 1 | State 5 | 0.00336161 | 0.00308484 | 0.00364644 | 18 | Adult (25-44 years) | DF -> Mental MM |
| State 3 | State 5 | 0.1205033 | 0.10739294 | 0.13356088 | 18 | Adult (25-44 years) | Mental M -> Mental MM |
| State 5 | State 5 | 0.4326645 | 0.38291 | 0.48494354 | 18 | Adult (25-44 years) | Mental MM -> Mental MM |
| State 1 | State 6 | 0.02019224 | 0.01945057 | 0.02095146 | 18 | Adult (25-44 years) | DF -> Physical-Mental MM |
| State 2 | State 6 | 0.09691255 | 0.09112767 | 0.10297008 | 18 | Adult (25-44 years) | Physical M -> Physical-Mental MM |
| State 3 | State 6 | 0.32744438 | 0.30907066 | 0.34414278 | 18 | Adult (25-44 years) | Mental M -> Physical-Mental MM |
| State 4 | State 6 | 0.12774295 | 0.1142286 | 0.14215864 | 18 | Adult (25-44 years) | Physical MM -> Physical-Mental MM |
| State 5 | State 6 | 0.35982586 | 0.31713259 | 0.40296833 | 18 | Adult (25-44 years) | Mental MM -> Physical-Mental MM |
| State 6 | State 6 | 0.69995306 | 0.66934179 | 0.72702881 | 18 | Adult (25-44 years) | Physical-Mental MM -> Physical-Mental MM |
| State 1 | State 7 | 0.02064999 | 0.01986569 | 0.02148396 | 18 | Adult (25-44 years) | DF -> Death |
| State 2 | State 7 | 0.09080817 | 0.0853333 | 0.096695 | 18 | Adult (25-44 years) | Physical M -> Death |
| State 3 | State 7 | 0.16018063 | 0.14826292 | 0.17483033 | 18 | Adult (25-44 years) | Mental M -> Death |
| State 4 | State 7 | 0.23671949 | 0.21949042 | 0.25506255 | 18 | Adult (25-44 years) | Physical MM -> Death |
| State 5 | State 7 | 0.20750964 | 0.17544501 | 0.24949126 | 18 | Adult (25-44 years) | Mental MM -> Death |
| State 6 | State 7 | 0.30004694 | 0.27297119 | 0.33065821 | 18 | Adult (25-44 years) | Physical-Mental MM -> Death |
| State 1 | State 1 | 0.78313768 | 0.7813207 | 0.78496268 | 19 | Adult (25-44 years) | DF -> DF |
| State 1 | State 2 | 0.10590649 | 0.10432227 | 0.10741477 | 19 | Adult (25-44 years) | DF -> Physical M |
| State 2 | State 2 | 0.49496374 | 0.48370755 | 0.50614761 | 19 | Adult (25-44 years) | Physical M -> Physical M |
| State 1 | State 3 | 0.01636873 | 0.01578919 | 0.01698142 | 19 | Adult (25-44 years) | DF -> Mental M |
| State 3 | State 3 | 0.37199814 | 0.35165526 | 0.39098589 | 19 | Adult (25-44 years) | Mental M -> Mental M |
| State 1 | State 4 | 0.04670048 | 0.04550281 | 0.0478723 | 19 | Adult (25-44 years) | DF -> Physical MM |
| State 2 | State 4 | 0.30565653 | 0.29593576 | 0.31527186 | 19 | Adult (25-44 years) | Physical M -> Physical MM |
| State 4 | State 4 | 0.61973301 | 0.59782145 | 0.6412938 | 19 | Adult (25-44 years) | Physical MM -> Physical MM |
| State 1 | State 5 | 0.0035542 | 0.00324225 | 0.00386551 | 19 | Adult (25-44 years) | DF -> Mental MM |
| State 3 | State 5 | 0.12108541 | 0.10773274 | 0.13397023 | 19 | Adult (25-44 years) | Mental M -> Mental MM |
| State 5 | State 5 | 0.41298801 | 0.36116678 | 0.46550232 | 19 | Adult (25-44 years) | Mental MM -> Mental MM |
| State 1 | State 6 | 0.0218227 | 0.02097982 | 0.02266661 | 19 | Adult (25-44 years) | DF -> Physical-Mental MM |
| State 2 | State 6 | 0.10124907 | 0.09545841 | 0.10776596 | 19 | Adult (25-44 years) | Physical M -> Physical-Mental MM |
| State 3 | State 6 | 0.33641611 | 0.31723714 | 0.35509482 | 19 | Adult (25-44 years) | Mental M -> Physical-Mental MM |
| State 4 | State 6 | 0.13184551 | 0.11835418 | 0.1462883 | 19 | Adult (25-44 years) | Physical MM -> Physical-Mental MM |
| State 5 | State 6 | 0.36782325 | 0.32477138 | 0.40683167 | 19 | Adult (25-44 years) | Mental MM -> Physical-Mental MM |
| State 6 | State 6 | 0.68621726 | 0.65664175 | 0.7146973 | 19 | Adult (25-44 years) | Physical-Mental MM -> Physical-Mental MM |
| State 1 | State 7 | 0.02250973 | 0.02169861 | 0.02339492 | 19 | Adult (25-44 years) | DF -> Death |
| State 2 | State 7 | 0.09813066 | 0.09216469 | 0.10441281 | 19 | Adult (25-44 years) | Physical M -> Death |
| State 3 | State 7 | 0.17050034 | 0.15646437 | 0.18634409 | 19 | Adult (25-44 years) | Mental M -> Death |
| State 4 | State 7 | 0.24842148 | 0.22967006 | 0.26554313 | 19 | Adult (25-44 years) | Physical MM -> Death |
| State 5 | State 7 | 0.21918874 | 0.18928748 | 0.26036032 | 19 | Adult (25-44 years) | Mental MM -> Death |
| State 6 | State 7 | 0.31378274 | 0.2853027 | 0.34335825 | 19 | Adult (25-44 years) | Physical-Mental MM -> Death |
| State 1 | State 1 | 0.77312666 | 0.77109865 | 0.77495274 | 20 | Adult (25-44 years) | DF -> DF |
| State 1 | State 2 | 0.1088373 | 0.10720541 | 0.11031937 | 20 | Adult (25-44 years) | DF -> Physical M |
| State 2 | State 2 | 0.47697794 | 0.4655134 | 0.4880013 | 20 | Adult (25-44 years) | Physical M -> Physical M |
| State 1 | State 3 | 0.01672126 | 0.01610647 | 0.01732074 | 20 | Adult (25-44 years) | DF -> Mental M |
| State 3 | State 3 | 0.35313247 | 0.33349976 | 0.37200907 | 20 | Adult (25-44 years) | Mental M -> Mental M |
| State 1 | State 4 | 0.04963883 | 0.04828552 | 0.05094019 | 20 | Adult (25-44 years) | DF -> Physical MM |
| State 2 | State 4 | 0.3119629 | 0.30173664 | 0.32226713 | 20 | Adult (25-44 years) | Physical M -> Physical MM |
| State 4 | State 4 | 0.60432148 | 0.5797295 | 0.62525735 | 20 | Adult (25-44 years) | Physical MM -> Physical MM |
| State 1 | State 5 | 0.00374276 | 0.00341637 | 0.00407259 | 20 | Adult (25-44 years) | DF -> Mental MM |
| State 3 | State 5 | 0.1213336 | 0.10764582 | 0.1347396 | 20 | Adult (25-44 years) | Mental M -> Mental MM |
| State 5 | State 5 | 0.39420636 | 0.34023706 | 0.44500182 | 20 | Adult (25-44 years) | Mental MM -> Mental MM |
| State 1 | State 6 | 0.02348385 | 0.02258197 | 0.02452464 | 20 | Adult (25-44 years) | DF -> Physical-Mental MM |
| State 2 | State 6 | 0.10545951 | 0.09894556 | 0.11285874 | 20 | Adult (25-44 years) | Physical M -> Physical-Mental MM |
| State 3 | State 6 | 0.34466386 | 0.32576231 | 0.36258351 | 20 | Adult (25-44 years) | Mental M -> Physical-Mental MM |
| State 4 | State 6 | 0.1357032 | 0.12238684 | 0.15180408 | 20 | Adult (25-44 years) | Physical MM -> Physical-Mental MM |
| State 5 | State 6 | 0.37497888 | 0.3342634 | 0.41692235 | 20 | Adult (25-44 years) | Mental MM -> Physical-Mental MM |
| State 6 | State 6 | 0.672751 | 0.6430463 | 0.70087864 | 20 | Adult (25-44 years) | Physical-Mental MM -> Physical-Mental MM |
| State 1 | State 7 | 0.02444934 | 0.02356024 | 0.02532009 | 20 | Adult (25-44 years) | DF -> Death |
| State 2 | State 7 | 0.10559965 | 0.09912551 | 0.11221487 | 20 | Adult (25-44 years) | Physical M -> Death |
| State 3 | State 7 | 0.18087007 | 0.1664574 | 0.19637521 | 20 | Adult (25-44 years) | Mental M -> Death |
| State 4 | State 7 | 0.25997532 | 0.24163766 | 0.27869707 | 20 | Adult (25-44 years) | Physical MM -> Death |
| State 5 | State 7 | 0.23081476 | 0.19738278 | 0.27178918 | 20 | Adult (25-44 years) | Mental MM -> Death |
| State 6 | State 7 | 0.327249 | 0.29912136 | 0.3569537 | 20 | Adult (25-44 years) | Physical-Mental MM -> Death |
| State 1 | State 1 | 1 | 1 | 1 | 0 | Middle Age (45-64 years) | DF -> DF |
| State 1 | State 2 | 0 | 0 | 0 | 0 | Middle Age (45-64 years) | DF -> Physical M |
| State 2 | State 2 | 1 | 1 | 1 | 0 | Middle Age (45-64 years) | Physical M -> Physical M |
| State 1 | State 3 | 0 | 0 | 0 | 0 | Middle Age (45-64 years) | DF -> Mental M |
| State 3 | State 3 | 1 | 1 | 1 | 0 | Middle Age (45-64 years) | Mental M -> Mental M |
| State 1 | State 4 | 0 | 0 | 0 | 0 | Middle Age (45-64 years) | DF -> Physical MM |
| State 2 | State 4 | 0 | 0 | 0 | 0 | Middle Age (45-64 years) | Physical M -> Physical MM |
| State 4 | State 4 | 1 | 1 | 1 | 0 | Middle Age (45-64 years) | Physical MM -> Physical MM |
| State 1 | State 5 | 0 | 0 | 0 | 0 | Middle Age (45-64 years) | DF -> Mental MM |
| State 3 | State 5 | 0 | 0 | 0 | 0 | Middle Age (45-64 years) | Mental M -> Mental MM |
| State 5 | State 5 | 1 | 1 | 1 | 0 | Middle Age (45-64 years) | Mental MM -> Mental MM |
| State 1 | State 6 | 0 | 0 | 0 | 0 | Middle Age (45-64 years) | DF -> Physical-Mental MM |
| State 2 | State 6 | 0 | 0 | 0 | 0 | Middle Age (45-64 years) | Physical M -> Physical-Mental MM |
| State 3 | State 6 | 0 | 0 | 0 | 0 | Middle Age (45-64 years) | Mental M -> Physical-Mental MM |
| State 4 | State 6 | 0 | 0 | 0 | 0 | Middle Age (45-64 years) | Physical MM -> Physical-Mental MM |
| State 5 | State 6 | 0 | 0 | 0 | 0 | Middle Age (45-64 years) | Mental MM -> Physical-Mental MM |
| State 6 | State 6 | 1 | 1 | 1 | 0 | Middle Age (45-64 years) | Physical-Mental MM -> Physical-Mental MM |
| State 1 | State 7 | 0 | 0 | 0 | 0 | Middle Age (45-64 years) | DF -> Death |
| State 2 | State 7 | 0 | 0 | 0 | 0 | Middle Age (45-64 years) | Physical M -> Death |
| State 3 | State 7 | 0 | 0 | 0 | 0 | Middle Age (45-64 years) | Mental M -> Death |
| State 4 | State 7 | 0 | 0 | 0 | 0 | Middle Age (45-64 years) | Physical MM -> Death |
| State 5 | State 7 | 0 | 0 | 0 | 0 | Middle Age (45-64 years) | Mental MM -> Death |
| State 6 | State 7 | 0 | 0 | 0 | 0 | Middle Age (45-64 years) | Physical-Mental MM -> Death |
| State 1 | State 1 | 0.955766 | 0.95542782 | 0.95609464 | 1 | Middle Age (45-64 years) | DF -> DF |
| State 1 | State 2 | 0.02719639 | 0.0269464 | 0.02745656 | 1 | Middle Age (45-64 years) | DF -> Physical M |
| State 2 | State 2 | 0.91593925 | 0.9147983 | 0.91715396 | 1 | Middle Age (45-64 years) | Physical M -> Physical M |
| State 1 | State 3 | 0.00149035 | 0.00142157 | 0.00155341 | 1 | Middle Age (45-64 years) | DF -> Mental M |
| State 3 | State 3 | 0.88905043 | 0.88397155 | 0.89372275 | 1 | Middle Age (45-64 years) | Mental M -> Mental M |
| State 1 | State 4 | 0.01137376 | 0.01121599 | 0.01153608 | 1 | Middle Age (45-64 years) | DF -> Physical MM |
| State 2 | State 4 | 0.07202477 | 0.07097883 | 0.07307206 | 1 | Middle Age (45-64 years) | Physical M -> Physical MM |
| State 4 | State 4 | 0.9566656 | 0.95559406 | 0.95770574 | 1 | Middle Age (45-64 years) | Physical MM -> Physical MM |
| State 1 | State 5 | 0.00010703 | 9.13E-05 | 0.00012598 | 1 | Middle Age (45-64 years) | DF -> Mental MM |
| State 3 | State 5 | 0.01478549 | 0.01321843 | 0.01681105 | 1 | Middle Age (45-64 years) | Mental M -> Mental MM |
| State 5 | State 5 | 0.89127872 | 0.87768164 | 0.90302652 | 1 | Middle Age (45-64 years) | Mental MM -> Mental MM |
| State 1 | State 6 | 0.00157124 | 0.00151378 | 0.00163329 | 1 | Middle Age (45-64 years) | DF -> Physical-Mental MM |
| State 2 | State 6 | 0.00539235 | 0.00509023 | 0.00571003 | 1 | Middle Age (45-64 years) | Physical M -> Physical-Mental MM |
| State 3 | State 6 | 0.07838147 | 0.07439036 | 0.08273968 | 1 | Middle Age (45-64 years) | Mental M -> Physical-Mental MM |
| State 4 | State 6 | 0.01004831 | 0.00956084 | 0.01055083 | 1 | Middle Age (45-64 years) | Physical MM -> Physical-Mental MM |
| State 5 | State 6 | 0.08504704 | 0.07466791 | 0.09657847 | 1 | Middle Age (45-64 years) | Mental MM -> Physical-Mental MM |
| State 6 | State 6 | 0.94643951 | 0.94392201 | 0.94896113 | 1 | Middle Age (45-64 years) | Physical-Mental MM -> Physical-Mental MM |
| State 1 | State 7 | 0.00249524 | 0.00241469 | 0.00257995 | 1 | Middle Age (45-64 years) | DF -> Death |
| State 2 | State 7 | 0.00664363 | 0.00633256 | 0.00697074 | 1 | Middle Age (45-64 years) | Physical M -> Death |
| State 3 | State 7 | 0.01778261 | 0.01589363 | 0.02005828 | 1 | Middle Age (45-64 years) | Mental M -> Death |
| State 4 | State 7 | 0.03328609 | 0.03236958 | 0.03432164 | 1 | Middle Age (45-64 years) | Physical MM -> Death |
| State 5 | State 7 | 0.02367424 | 0.01848441 | 0.03062778 | 1 | Middle Age (45-64 years) | Mental MM -> Death |
| State 6 | State 7 | 0.05356049 | 0.05103887 | 0.05607799 | 1 | Middle Age (45-64 years) | Physical-Mental MM -> Death |
| State 1 | State 1 | 0.91348864 | 0.91282817 | 0.91411474 | 2 | Middle Age (45-64 years) | DF -> DF |
| State 1 | State 2 | 0.05090363 | 0.05043506 | 0.05141929 | 2 | Middle Age (45-64 years) | DF -> Physical M |
| State 2 | State 2 | 0.83894471 | 0.83680685 | 0.84118505 | 2 | Middle Age (45-64 years) | Physical M -> Physical M |
| State 1 | State 3 | 0.00274942 | 0.00264054 | 0.00287565 | 2 | Middle Age (45-64 years) | DF -> Mental M |
| State 3 | State 3 | 0.79041067 | 0.78180583 | 0.79870322 | 2 | Middle Age (45-64 years) | Mental M -> Mental M |
| State 1 | State 4 | 0.02371035 | 0.02339765 | 0.02402137 | 2 | Middle Age (45-64 years) | DF -> Physical MM |
| State 2 | State 4 | 0.13487394 | 0.13275236 | 0.13694125 | 2 | Middle Age (45-64 years) | Physical M -> Physical MM |
| State 4 | State 4 | 0.91520907 | 0.91308235 | 0.9172111 | 2 | Middle Age (45-64 years) | Physical MM -> Physical MM |
| State 1 | State 5 | 0.00021972 | 0.00019188 | 0.00025417 | 2 | Middle Age (45-64 years) | DF -> Mental MM |
| State 3 | State 5 | 0.02632304 | 0.02333586 | 0.02956907 | 2 | Middle Age (45-64 years) | Mental M -> Mental MM |
| State 5 | State 5 | 0.79437776 | 0.76920048 | 0.81471366 | 2 | Middle Age (45-64 years) | Mental MM -> Mental MM |
| State 1 | State 6 | 0.00337567 | 0.00325875 | 0.00349675 | 2 | Middle Age (45-64 years) | DF -> Physical-Mental MM |
| State 2 | State 6 | 0.01076633 | 0.01020694 | 0.01131866 | 2 | Middle Age (45-64 years) | Physical M -> Physical-Mental MM |
| State 3 | State 6 | 0.14512586 | 0.13840543 | 0.15234449 | 2 | Middle Age (45-64 years) | Mental M -> Physical-Mental MM |
| State 4 | State 6 | 0.01912298 | 0.0182716 | 0.02009481 | 2 | Middle Age (45-64 years) | Physical MM -> Physical-Mental MM |
| State 5 | State 6 | 0.15629249 | 0.13853207 | 0.17697975 | 2 | Middle Age (45-64 years) | Mental MM -> Physical-Mental MM |
| State 6 | State 6 | 0.89574774 | 0.89085463 | 0.90021946 | 2 | Middle Age (45-64 years) | Physical-Mental MM -> Physical-Mental MM |
| State 1 | State 7 | 0.00555257 | 0.00540202 | 0.00570876 | 2 | Middle Age (45-64 years) | DF -> Death |
| State 2 | State 7 | 0.01541502 | 0.0147713 | 0.01606644 | 2 | Middle Age (45-64 years) | Physical M -> Death |
| State 3 | State 7 | 0.03814043 | 0.03452673 | 0.04209433 | 2 | Middle Age (45-64 years) | Mental M -> Death |
| State 4 | State 7 | 0.06566795 | 0.06380721 | 0.06745886 | 2 | Middle Age (45-64 years) | Physical MM -> Death |
| State 5 | State 7 | 0.04932975 | 0.03984426 | 0.06387894 | 2 | Middle Age (45-64 years) | Mental MM -> Death |
| State 6 | State 7 | 0.10425226 | 0.09978054 | 0.10914537 | 2 | Middle Age (45-64 years) | Physical-Mental MM -> Death |
| State 1 | State 1 | 0.87308138 | 0.87218323 | 0.87390251 | 3 | Middle Age (45-64 years) | DF -> DF |
| State 1 | State 2 | 0.07146822 | 0.07083092 | 0.07215443 | 3 | Middle Age (45-64 years) | DF -> Physical M |
| State 2 | State 2 | 0.76842239 | 0.76555372 | 0.77136259 | 3 | Middle Age (45-64 years) | Physical M -> Physical M |
| State 1 | State 3 | 0.00380578 | 0.00364919 | 0.00397366 | 3 | Middle Age (45-64 years) | DF -> Mental M |
| State 3 | State 3 | 0.70271495 | 0.69138012 | 0.71399391 | 3 | Middle Age (45-64 years) | Mental M -> Mental M |
| State 1 | State 4 | 0.036739 | 0.0362827 | 0.03720521 | 3 | Middle Age (45-64 years) | DF -> Physical MM |
| State 2 | State 4 | 0.18945406 | 0.18669806 | 0.19207663 | 3 | Middle Age (45-64 years) | Physical M -> Physical MM |
| State 4 | State 4 | 0.87554903 | 0.87274038 | 0.87849582 | 3 | Middle Age (45-64 years) | Physical MM -> Physical MM |
| State 1 | State 5 | 0.00033426 | 0.00029389 | 0.00037747 | 3 | Middle Age (45-64 years) | DF -> Mental MM |
| State 3 | State 5 | 0.03514777 | 0.03096674 | 0.03948191 | 3 | Middle Age (45-64 years) | Mental M -> Mental MM |
| State 5 | State 5 | 0.70801199 | 0.67691255 | 0.73658732 | 3 | Middle Age (45-64 years) | Mental MM -> Mental MM |
| State 1 | State 6 | 0.0053771 | 0.00520465 | 0.00556242 | 3 | Middle Age (45-64 years) | DF -> Physical-Mental MM |
| State 2 | State 6 | 0.01606882 | 0.01532943 | 0.01689121 | 3 | Middle Age (45-64 years) | Physical M -> Physical-Mental MM |
| State 3 | State 6 | 0.2015451 | 0.1931799 | 0.21090406 | 3 | Middle Age (45-64 years) | Mental M -> Physical-Mental MM |
| State 4 | State 6 | 0.02729505 | 0.02600135 | 0.02871043 | 3 | Middle Age (45-64 years) | Physical MM -> Physical-Mental MM |
| State 5 | State 6 | 0.21548087 | 0.19106044 | 0.24202992 | 3 | Middle Age (45-64 years) | Mental MM -> Physical-Mental MM |
| State 6 | State 6 | 0.84777105 | 0.84077784 | 0.85463933 | 3 | Middle Age (45-64 years) | Physical-Mental MM -> Physical-Mental MM |
| State 1 | State 7 | 0.00919425 | 0.00897874 | 0.00943507 | 3 | Middle Age (45-64 years) | DF -> Death |
| State 2 | State 7 | 0.02605473 | 0.02519769 | 0.02695933 | 3 | Middle Age (45-64 years) | Physical M -> Death |
| State 3 | State 7 | 0.06059219 | 0.05552553 | 0.06667429 | 3 | Middle Age (45-64 years) | Mental M -> Death |
| State 4 | State 7 | 0.09715592 | 0.09455201 | 0.0997602 | 3 | Middle Age (45-64 years) | Physical MM -> Death |
| State 5 | State 7 | 0.07650714 | 0.06344691 | 0.09404394 | 3 | Middle Age (45-64 years) | Mental MM -> Death |
| State 6 | State 7 | 0.15222895 | 0.14536067 | 0.15922216 | 3 | Middle Age (45-64 years) | Physical-Mental MM -> Death |
| State 1 | State 1 | 0.8344615 | 0.83331547 | 0.83563534 | 4 | Middle Age (45-64 years) | DF -> DF |
| State 1 | State 2 | 0.08920521 | 0.08834468 | 0.08999902 | 4 | Middle Age (45-64 years) | DF -> Physical M |
| State 2 | State 2 | 0.70382823 | 0.70025534 | 0.70729303 | 4 | Middle Age (45-64 years) | Physical M -> Physical M |
| State 1 | State 3 | 0.00468473 | 0.00449811 | 0.00490424 | 4 | Middle Age (45-64 years) | DF -> Mental M |
| State 3 | State 3 | 0.62474903 | 0.60928038 | 0.63811502 | 4 | Middle Age (45-64 years) | Mental M -> Mental M |
| State 1 | State 4 | 0.05022463 | 0.04961042 | 0.0508398 | 4 | Middle Age (45-64 years) | DF -> Physical MM |
| State 2 | State 4 | 0.23658963 | 0.23355857 | 0.23991736 | 4 | Middle Age (45-64 years) | Physical M -> Physical MM |
| State 4 | State 4 | 0.83760764 | 0.83412018 | 0.84108728 | 4 | Middle Age (45-64 years) | Physical MM -> Physical MM |
| State 1 | State 5 | 0.00044763 | 0.00039714 | 0.00050813 | 4 | Middle Age (45-64 years) | DF -> Mental MM |
| State 3 | State 5 | 0.04171644 | 0.03722946 | 0.04678972 | 4 | Middle Age (45-64 years) | Mental M -> Mental MM |
| State 5 | State 5 | 0.63103602 | 0.59500361 | 0.66422788 | 4 | Middle Age (45-64 years) | Mental MM -> Mental MM |
| State 1 | State 6 | 0.0075422 | 0.00730559 | 0.00775459 | 4 | Middle Age (45-64 years) | DF -> Physical-Mental MM |
| State 2 | State 6 | 0.02125546 | 0.02027706 | 0.02228448 | 4 | Middle Age (45-64 years) | Physical M -> Physical-Mental MM |
| State 3 | State 6 | 0.24881929 | 0.23875829 | 0.26018225 | 4 | Middle Age (45-64 years) | Mental M -> Physical-Mental MM |
| State 4 | State 6 | 0.0346309 | 0.03302398 | 0.03645289 | 4 | Middle Age (45-64 years) | Physical MM -> Physical-Mental MM |
| State 5 | State 6 | 0.26415393 | 0.23617991 | 0.29367263 | 4 | Middle Age (45-64 years) | Mental MM -> Physical-Mental MM |
| State 6 | State 6 | 0.80236402 | 0.79374644 | 0.81104816 | 4 | Middle Age (45-64 years) | Physical-Mental MM -> Physical-Mental MM |
| State 1 | State 7 | 0.0134341 | 0.0131241 | 0.01373664 | 4 | Middle Age (45-64 years) | DF -> Death |
| State 2 | State 7 | 0.03832668 | 0.03709037 | 0.03958862 | 4 | Middle Age (45-64 years) | Physical M -> Death |
| State 3 | State 7 | 0.08471524 | 0.07847563 | 0.09219075 | 4 | Middle Age (45-64 years) | Mental M -> Death |
| State 4 | State 7 | 0.12776146 | 0.12452167 | 0.13091582 | 4 | Middle Age (45-64 years) | Physical MM -> Death |
| State 5 | State 7 | 0.10481004 | 0.08743864 | 0.12733552 | 4 | Middle Age (45-64 years) | Mental MM -> Death |
| State 6 | State 7 | 0.19763598 | 0.18895184 | 0.20625356 | 4 | Middle Age (45-64 years) | Physical-Mental MM -> Death |
| State 1 | State 1 | 0.79754993 | 0.79616871 | 0.79901893 | 5 | Middle Age (45-64 years) | DF -> DF |
| State 1 | State 2 | 0.1044009 | 0.10337451 | 0.10541386 | 5 | Middle Age (45-64 years) | DF -> Physical M |
| State 2 | State 2 | 0.6446639 | 0.64040411 | 0.64880867 | 5 | Middle Age (45-64 years) | Physical M -> Physical M |
| State 1 | State 3 | 0.0054086 | 0.00515642 | 0.00565388 | 5 | Middle Age (45-64 years) | DF -> Mental M |
| State 3 | State 3 | 0.55543339 | 0.54107607 | 0.56981818 | 5 | Middle Age (45-64 years) | Mental M -> Mental M |
| State 1 | State 4 | 0.06396413 | 0.06326441 | 0.0646967 | 5 | Middle Age (45-64 years) | DF -> Physical MM |
| State 2 | State 4 | 0.27703022 | 0.27336801 | 0.28062746 | 5 | Middle Age (45-64 years) | Physical M -> Physical MM |
| State 4 | State 4 | 0.80131041 | 0.79663161 | 0.80544784 | 5 | Middle Age (45-64 years) | Physical MM -> Physical MM |
| State 1 | State 5 | 0.00055754 | 0.00049547 | 0.00062896 | 5 | Middle Age (45-64 years) | DF -> Mental MM |
| State 3 | State 5 | 0.0464182 | 0.040791 | 0.05233525 | 5 | Middle Age (45-64 years) | Mental M -> Mental MM |
| State 5 | State 5 | 0.56242898 | 0.52150121 | 0.59960855 | 5 | Middle Age (45-64 years) | Mental MM -> Mental MM |
| State 1 | State 6 | 0.00984033 | 0.0095638 | 0.01011359 | 5 | Middle Age (45-64 years) | DF -> Physical-Mental MM |
| State 2 | State 6 | 0.02628962 | 0.02518402 | 0.02748013 | 5 | Middle Age (45-64 years) | Physical M -> Physical-Mental MM |
| State 3 | State 6 | 0.28800901 | 0.27565574 | 0.30050827 | 5 | Middle Age (45-64 years) | Mental M -> Physical-Mental MM |
| State 4 | State 6 | 0.04119259 | 0.03927424 | 0.04335733 | 5 | Middle Age (45-64 years) | Physical MM -> Physical-Mental MM |
| State 5 | State 6 | 0.30367346 | 0.27387961 | 0.33697428 | 5 | Middle Age (45-64 years) | Mental MM -> Physical-Mental MM |
| State 6 | State 6 | 0.75938901 | 0.75006644 | 0.76969835 | 5 | Middle Age (45-64 years) | Physical-Mental MM -> Physical-Mental MM |
| State 1 | State 7 | 0.01827857 | 0.0178603 | 0.01868891 | 5 | Middle Age (45-64 years) | DF -> Death |
| State 2 | State 7 | 0.05201625 | 0.05051454 | 0.0536536 | 5 | Middle Age (45-64 years) | Physical M -> Death |
| State 3 | State 7 | 0.1101394 | 0.10201708 | 0.11882264 | 5 | Middle Age (45-64 years) | Mental M -> Death |
| State 4 | State 7 | 0.157497 | 0.15359414 | 0.16177577 | 5 | Middle Age (45-64 years) | Physical MM -> Death |
| State 5 | State 7 | 0.13389756 | 0.1140982 | 0.15929975 | 5 | Middle Age (45-64 years) | Mental MM -> Death |
| State 6 | State 7 | 0.24061099 | 0.23030165 | 0.24993356 | 5 | Middle Age (45-64 years) | Physical-Mental MM -> Death |
| State 1 | State 1 | 0.7622711 | 0.76079234 | 0.76381275 | 6 | Middle Age (45-64 years) | DF -> DF |
| State 1 | State 2 | 0.11731536 | 0.11621961 | 0.11846813 | 6 | Middle Age (45-64 years) | DF -> Physical M |
| State 2 | State 2 | 0.59047297 | 0.58608537 | 0.59508907 | 6 | Middle Age (45-64 years) | Physical M -> Physical M |
| State 1 | State 3 | 0.00599714 | 0.00571111 | 0.00626936 | 6 | Middle Age (45-64 years) | DF -> Mental M |
| State 3 | State 3 | 0.4938083 | 0.4773416 | 0.51034644 | 6 | Middle Age (45-64 years) | Mental M -> Mental M |
| State 1 | State 4 | 0.07778287 | 0.07688772 | 0.07863139 | 6 | Middle Age (45-64 years) | DF -> Physical MM |
| State 2 | State 4 | 0.31145706 | 0.30754027 | 0.31533861 | 6 | Middle Age (45-64 years) | Physical M -> Physical MM |
| State 4 | State 4 | 0.7665861 | 0.7614523 | 0.77164269 | 6 | Middle Age (45-64 years) | Physical MM -> Physical MM |
| State 1 | State 5 | 0.00066226 | 0.00058957 | 0.00074488 | 6 | Middle Age (45-64 years) | DF -> Mental MM |
| State 3 | State 5 | 0.04958391 | 0.0434926 | 0.05582542 | 6 | Middle Age (45-64 years) | Mental M -> Mental MM |
| State 5 | State 5 | 0.50128098 | 0.45655183 | 0.5378665 | 6 | Middle Age (45-64 years) | Mental MM -> Mental MM |
| State 1 | State 6 | 0.01224347 | 0.01190802 | 0.01260046 | 6 | Middle Age (45-64 years) | DF -> Physical-Mental MM |
| State 2 | State 6 | 0.03114147 | 0.02985418 | 0.03245889 | 6 | Middle Age (45-64 years) | Physical M -> Physical-Mental MM |
| State 3 | State 6 | 0.32006652 | 0.30721404 | 0.33350921 | 6 | Middle Age (45-64 years) | Mental M -> Physical-Mental MM |
| State 4 | State 6 | 0.04703811 | 0.04479532 | 0.04918651 | 6 | Middle Age (45-64 years) | Physical MM -> Physical-Mental MM |
| State 5 | State 6 | 0.33524148 | 0.30383722 | 0.37058101 | 6 | Middle Age (45-64 years) | Mental MM -> Physical-Mental MM |
| State 6 | State 6 | 0.71871576 | 0.70661855 | 0.73054111 | 6 | Middle Age (45-64 years) | Physical-Mental MM -> Physical-Mental MM |
| State 1 | State 7 | 0.0237278 | 0.023267 | 0.02427075 | 6 | Middle Age (45-64 years) | DF -> Death |
| State 2 | State 7 | 0.0669285 | 0.06513109 | 0.06887272 | 6 | Middle Age (45-64 years) | Physical M -> Death |
| State 3 | State 7 | 0.13654127 | 0.12764542 | 0.1465946 | 6 | Middle Age (45-64 years) | Mental M -> Death |
| State 4 | State 7 | 0.18637579 | 0.18206881 | 0.19070349 | 6 | Middle Age (45-64 years) | Physical MM -> Death |
| State 5 | State 7 | 0.16347754 | 0.14150403 | 0.19171316 | 6 | Middle Age (45-64 years) | Mental MM -> Death |
| State 6 | State 7 | 0.28128424 | 0.26945889 | 0.29338145 | 6 | Middle Age (45-64 years) | Physical-Mental MM -> Death |
| State 1 | State 1 | 0.7285528 | 0.72674698 | 0.73040603 | 7 | Middle Age (45-64 years) | DF -> DF |
| State 1 | State 2 | 0.12818476 | 0.12702544 | 0.12938738 | 7 | Middle Age (45-64 years) | DF -> Physical M |
| State 2 | State 2 | 0.54083737 | 0.53655156 | 0.5458102 | 7 | Middle Age (45-64 years) | Physical M -> Physical M |
| State 1 | State 3 | 0.00646781 | 0.00615753 | 0.00675663 | 7 | Middle Age (45-64 years) | DF -> Mental M |
| State 3 | State 3 | 0.43902048 | 0.42342176 | 0.45649126 | 7 | Middle Age (45-64 years) | Mental M -> Mental M |
| State 1 | State 4 | 0.0915317 | 0.09054933 | 0.09254017 | 7 | Middle Age (45-64 years) | DF -> Physical MM |
| State 2 | State 4 | 0.34048893 | 0.33626244 | 0.34428143 | 7 | Middle Age (45-64 years) | Physical M -> Physical MM |
| State 4 | State 4 | 0.73336656 | 0.72764644 | 0.73909796 | 7 | Middle Age (45-64 years) | Physical MM -> Physical MM |
| State 1 | State 5 | 0.00076051 | 0.00067873 | 0.000849 | 7 | Middle Age (45-64 years) | DF -> Mental MM |
| State 3 | State 5 | 0.05149428 | 0.04512894 | 0.05773232 | 7 | Middle Age (45-64 years) | Mental M -> Mental MM |
| State 5 | State 5 | 0.44678107 | 0.40029784 | 0.48716196 | 7 | Middle Age (45-64 years) | Mental MM -> Mental MM |
| State 1 | State 6 | 0.01472599 | 0.01429773 | 0.01509693 | 7 | Middle Age (45-64 years) | DF -> Physical-Mental MM |
| State 2 | State 6 | 0.03578717 | 0.03430996 | 0.03727932 | 7 | Middle Age (45-64 years) | Physical M -> Physical-Mental MM |
| State 3 | State 6 | 0.34584599 | 0.33203049 | 0.35988884 | 7 | Middle Age (45-64 years) | Mental M -> Physical-Mental MM |
| State 4 | State 6 | 0.05222161 | 0.04970279 | 0.05471074 | 7 | Middle Age (45-64 years) | Physical MM -> Physical-Mental MM |
| State 5 | State 6 | 0.35991825 | 0.32739915 | 0.39287869 | 7 | Middle Age (45-64 years) | Mental MM -> Physical-Mental MM |
| State 6 | State 6 | 0.68022099 | 0.66732382 | 0.69232196 | 7 | Middle Age (45-64 years) | Physical-Mental MM -> Physical-Mental MM |
| State 1 | State 7 | 0.02977643 | 0.02917404 | 0.03041165 | 7 | Middle Age (45-64 years) | DF -> Death |
| State 2 | State 7 | 0.08288652 | 0.08063849 | 0.08507062 | 7 | Middle Age (45-64 years) | Physical M -> Death |
| State 3 | State 7 | 0.16363926 | 0.15404784 | 0.17403319 | 7 | Middle Age (45-64 years) | Mental M -> Death |
| State 4 | State 7 | 0.21441183 | 0.20925675 | 0.21942566 | 7 | Middle Age (45-64 years) | Physical MM -> Death |
| State 5 | State 7 | 0.19330068 | 0.16761727 | 0.22737098 | 7 | Middle Age (45-64 years) | Mental MM -> Death |
| State 6 | State 7 | 0.31977901 | 0.30767804 | 0.33267618 | 7 | Middle Age (45-64 years) | Physical-Mental MM -> Death |
| State 1 | State 1 | 0.69632599 | 0.69436472 | 0.69826476 | 8 | Middle Age (45-64 years) | DF -> DF |
| State 1 | State 2 | 0.13722346 | 0.13591796 | 0.13849562 | 8 | Middle Age (45-64 years) | DF -> Physical M |
| State 2 | State 2 | 0.49537418 | 0.49038928 | 0.5003198 | 8 | Middle Age (45-64 years) | Physical M -> Physical M |
| State 1 | State 3 | 0.00683601 | 0.00651361 | 0.007156 | 8 | Middle Age (45-64 years) | DF -> Mental M |
| State 3 | State 3 | 0.39031134 | 0.37293932 | 0.40877669 | 8 | Middle Age (45-64 years) | Mental M -> Mental M |
| State 1 | State 4 | 0.10508409 | 0.1040014 | 0.10633869 | 8 | Middle Age (45-64 years) | DF -> Physical MM |
| State 2 | State 4 | 0.36468774 | 0.36023505 | 0.36911145 | 8 | Middle Age (45-64 years) | Physical M -> Physical MM |
| State 4 | State 4 | 0.70158655 | 0.69525079 | 0.70775339 | 8 | Middle Age (45-64 years) | Physical MM -> Physical MM |
| State 1 | State 5 | 0.00085144 | 0.00075816 | 0.00095458 | 8 | Middle Age (45-64 years) | DF -> Mental MM |
| State 3 | State 5 | 0.05238689 | 0.04564682 | 0.05930716 | 8 | Middle Age (45-64 years) | Mental M -> Mental MM |
| State 5 | State 5 | 0.39820646 | 0.34995377 | 0.44282426 | 8 | Middle Age (45-64 years) | Mental MM -> Mental MM |
| State 1 | State 6 | 0.01726458 | 0.01681863 | 0.01773551 | 8 | Middle Age (45-64 years) | DF -> Physical-Mental MM |
| State 2 | State 6 | 0.04020812 | 0.03865467 | 0.04188412 | 8 | Middle Age (45-64 years) | Physical M -> Physical-Mental MM |
| State 3 | State 6 | 0.36611281 | 0.3508312 | 0.38118966 | 8 | Middle Age (45-64 years) | Mental M -> Physical-Mental MM |
| State 4 | State 6 | 0.05679369 | 0.05405447 | 0.05947002 | 8 | Middle Age (45-64 years) | Physical MM -> Physical-Mental MM |
| State 5 | State 6 | 0.37863826 | 0.34510252 | 0.41635108 | 8 | Middle Age (45-64 years) | Mental MM -> Physical-Mental MM |
| State 6 | State 6 | 0.64378802 | 0.62968747 | 0.65753455 | 8 | Middle Age (45-64 years) | Physical-Mental MM -> Physical-Mental MM |
| State 1 | State 7 | 0.03641444 | 0.03562502 | 0.03712091 | 8 | Middle Age (45-64 years) | DF -> Death |
| State 2 | State 7 | 0.09972996 | 0.09709321 | 0.10229017 | 8 | Middle Age (45-64 years) | Physical M -> Death |
| State 3 | State 7 | 0.19118895 | 0.17959923 | 0.2026017 | 8 | Middle Age (45-64 years) | Mental M -> Death |
| State 4 | State 7 | 0.24161975 | 0.2356309 | 0.24731114 | 8 | Middle Age (45-64 years) | Physical MM -> Death |
| State 5 | State 7 | 0.22315528 | 0.19570165 | 0.25463782 | 8 | Middle Age (45-64 years) | Mental MM -> Death |
| State 6 | State 7 | 0.35621198 | 0.34246545 | 0.37031253 | 8 | Middle Age (45-64 years) | Physical-Mental MM -> Death |
| State 1 | State 1 | 0.66552471 | 0.66331144 | 0.66764848 | 9 | Middle Age (45-64 years) | DF -> DF |
| State 1 | State 2 | 0.14462591 | 0.14313259 | 0.14607386 | 9 | Middle Age (45-64 years) | DF -> Physical M |
| State 2 | State 2 | 0.45373266 | 0.44877368 | 0.45916254 | 9 | Middle Age (45-64 years) | Physical M -> Physical M |
| State 1 | State 3 | 0.00711532 | 0.00677722 | 0.00745262 | 9 | Middle Age (45-64 years) | DF -> Mental M |
| State 3 | State 3 | 0.34700647 | 0.32944535 | 0.36413334 | 9 | Middle Age (45-64 years) | Mental M -> Mental M |
| State 1 | State 4 | 0.11833367 | 0.11700282 | 0.11963012 | 9 | Middle Age (45-64 years) | DF -> Physical MM |
| State 2 | State 4 | 0.38456342 | 0.37975925 | 0.38909007 | 9 | Middle Age (45-64 years) | Physical M -> Physical MM |
| State 4 | State 4 | 0.67118372 | 0.66385875 | 0.67794537 | 9 | Middle Age (45-64 years) | Physical MM -> Physical MM |
| State 1 | State 5 | 0.00093447 | 0.00083552 | 0.00104645 | 9 | Middle Age (45-64 years) | DF -> Mental MM |
| State 3 | State 5 | 0.05246226 | 0.04629555 | 0.05946922 | 9 | Middle Age (45-64 years) | Mental M -> Mental MM |
| State 5 | State 5 | 0.35491295 | 0.30897893 | 0.40086879 | 9 | Middle Age (45-64 years) | Mental MM -> Mental MM |
| State 1 | State 6 | 0.01983807 | 0.0193011 | 0.02041787 | 9 | Middle Age (45-64 years) | DF -> Physical-Mental MM |
| State 2 | State 6 | 0.04439028 | 0.04266445 | 0.04609229 | 9 | Middle Age (45-64 years) | Physical M -> Physical-Mental MM |
| State 3 | State 6 | 0.38155216 | 0.3672431 | 0.39661509 | 9 | Middle Age (45-64 years) | Mental M -> Physical-Mental MM |
| State 4 | State 6 | 0.06080155 | 0.0576962 | 0.06400936 | 9 | Middle Age (45-64 years) | Physical MM -> Physical-Mental MM |
| State 5 | State 6 | 0.39222449 | 0.35582323 | 0.42666772 | 9 | Middle Age (45-64 years) | Mental MM -> Physical-Mental MM |
| State 6 | State 6 | 0.60930642 | 0.59449907 | 0.62386082 | 9 | Middle Age (45-64 years) | Physical-Mental MM -> Physical-Mental MM |
| State 1 | State 7 | 0.04362786 | 0.04279102 | 0.04444863 | 9 | Middle Age (45-64 years) | DF -> Death |
| State 2 | State 7 | 0.11731364 | 0.11438943 | 0.12019891 | 9 | Middle Age (45-64 years) | Physical M -> Death |
| State 3 | State 7 | 0.21897911 | 0.20746991 | 0.23048229 | 9 | Middle Age (45-64 years) | Mental M -> Death |
| State 4 | State 7 | 0.26801473 | 0.2617132 | 0.27468547 | 9 | Middle Age (45-64 years) | Physical MM -> Death |
| State 5 | State 7 | 0.25286257 | 0.22336573 | 0.28531853 | 9 | Middle Age (45-64 years) | Mental MM -> Death |
| State 6 | State 7 | 0.39069358 | 0.37613918 | 0.40550093 | 9 | Middle Age (45-64 years) | Physical-Mental MM -> Death |
| State 1 | State 1 | 0.63608589 | 0.63381793 | 0.63828507 | 10 | Middle Age (45-64 years) | DF -> DF |
| State 1 | State 2 | 0.15056842 | 0.14908546 | 0.15214979 | 10 | Middle Age (45-64 years) | DF -> Physical M |
| State 2 | State 2 | 0.41559155 | 0.41060659 | 0.4208986 | 10 | Middle Age (45-64 years) | Physical M -> Physical M |
| State 1 | State 3 | 0.00731774 | 0.00696053 | 0.00770968 | 10 | Middle Age (45-64 years) | DF -> Mental M |
| State 3 | State 3 | 0.30850625 | 0.29156748 | 0.32495309 | 10 | Middle Age (45-64 years) | Mental M -> Mental M |
| State 1 | State 4 | 0.13119191 | 0.12979425 | 0.13269846 | 10 | Middle Age (45-64 years) | DF -> Physical MM |
| State 2 | State 4 | 0.40057859 | 0.39564675 | 0.40501365 | 10 | Middle Age (45-64 years) | Physical M -> Physical MM |
| State 4 | State 4 | 0.64209838 | 0.63487847 | 0.64894618 | 10 | Middle Age (45-64 years) | Physical MM -> Physical MM |
| State 1 | State 5 | 0.00100931 | 0.00089875 | 0.00113239 | 10 | Middle Age (45-64 years) | DF -> Mental MM |
| State 3 | State 5 | 0.05188916 | 0.04535263 | 0.05934153 | 10 | Middle Age (45-64 years) | Mental M -> Mental MM |
| State 5 | State 5 | 0.31632636 | 0.27783791 | 0.36011736 | 10 | Middle Age (45-64 years) | Mental MM -> Mental MM |
| State 1 | State 6 | 0.02242734 | 0.02184434 | 0.02312361 | 10 | Middle Age (45-64 years) | DF -> Physical-Mental MM |
| State 2 | State 6 | 0.04832361 | 0.04650062 | 0.05029817 | 10 | Middle Age (45-64 years) | Physical M -> Physical-Mental MM |
| State 3 | State 6 | 0.39277667 | 0.37979576 | 0.40698353 | 10 | Middle Age (45-64 years) | Mental M -> Physical-Mental MM |
| State 4 | State 6 | 0.06428925 | 0.06102603 | 0.06778244 | 10 | Middle Age (45-64 years) | Physical MM -> Physical-Mental MM |
| State 5 | State 6 | 0.40140105 | 0.36748766 | 0.43412657 | 10 | Middle Age (45-64 years) | Mental MM -> Physical-Mental MM |
| State 6 | State 6 | 0.57667167 | 0.56123036 | 0.59196317 | 10 | Middle Age (45-64 years) | Physical-Mental MM -> Physical-Mental MM |
| State 1 | State 7 | 0.0513994 | 0.05046348 | 0.05231583 | 10 | Middle Age (45-64 years) | DF -> Death |
| State 2 | State 7 | 0.13550625 | 0.13245646 | 0.13857457 | 10 | Middle Age (45-64 years) | Physical M -> Death |
| State 3 | State 7 | 0.24682792 | 0.23463436 | 0.25937364 | 10 | Middle Age (45-64 years) | Mental M -> Death |
| State 4 | State 7 | 0.29361237 | 0.2874476 | 0.30025309 | 10 | Middle Age (45-64 years) | Physical MM -> Death |
| State 5 | State 7 | 0.2822726 | 0.2532318 | 0.31542272 | 10 | Middle Age (45-64 years) | Mental MM -> Death |
| State 6 | State 7 | 0.42332833 | 0.40803683 | 0.43876964 | 10 | Middle Age (45-64 years) | Physical-Mental MM -> Death |
| State 1 | State 1 | 0.60794926 | 0.60557826 | 0.61014189 | 11 | Middle Age (45-64 years) | DF -> DF |
| State 1 | State 2 | 0.15521076 | 0.15354017 | 0.15682154 | 11 | Middle Age (45-64 years) | DF -> Physical M |
| State 2 | State 2 | 0.38065661 | 0.37491021 | 0.38639662 | 11 | Middle Age (45-64 years) | Physical M -> Physical M |
| State 1 | State 3 | 0.00745383 | 0.00709069 | 0.00783148 | 11 | Middle Age (45-64 years) | DF -> Mental M |
| State 3 | State 3 | 0.27427762 | 0.2575635 | 0.28996577 | 11 | Middle Age (45-64 years) | Mental M -> Mental M |
| State 1 | State 4 | 0.14358613 | 0.14203079 | 0.14518638 | 11 | Middle Age (45-64 years) | DF -> Physical MM |
| State 2 | State 4 | 0.41315264 | 0.40819828 | 0.41810991 | 11 | Middle Age (45-64 years) | Physical M -> Physical MM |
| State 4 | State 4 | 0.61427343 | 0.60681062 | 0.62141629 | 11 | Middle Age (45-64 years) | Physical MM -> Physical MM |
| State 1 | State 5 | 0.00107585 | 0.0009564 | 0.00121592 | 11 | Middle Age (45-64 years) | DF -> Mental MM |
| State 3 | State 5 | 0.05080912 | 0.04413234 | 0.05806957 | 11 | Middle Age (45-64 years) | Mental M -> Mental MM |
| State 5 | State 5 | 0.28193495 | 0.23860649 | 0.32078661 | 11 | Middle Age (45-64 years) | Mental MM -> Mental MM |
| State 1 | State 6 | 0.02501515 | 0.02437552 | 0.02568251 | 11 | Middle Age (45-64 years) | DF -> Physical-Mental MM |
| State 2 | State 6 | 0.05200153 | 0.05003297 | 0.05388159 | 11 | Middle Age (45-64 years) | Physical M -> Physical-Mental MM |
| State 3 | State 6 | 0.40033356 | 0.38696875 | 0.41390331 | 11 | Middle Age (45-64 years) | Mental M -> Physical-Mental MM |
| State 4 | State 6 | 0.06729789 | 0.06417978 | 0.07070585 | 11 | Middle Age (45-64 years) | Physical MM -> Physical-Mental MM |
| State 5 | State 6 | 0.40680443 | 0.37438173 | 0.43909872 | 11 | Middle Age (45-64 years) | Mental MM -> Physical-Mental MM |
| State 6 | State 6 | 0.54578485 | 0.52904647 | 0.5622268 | 11 | Middle Age (45-64 years) | Physical-Mental MM -> Physical-Mental MM |
| State 1 | State 7 | 0.05970901 | 0.05871481 | 0.06083061 | 11 | Middle Age (45-64 years) | DF -> Death |
| State 2 | State 7 | 0.15418922 | 0.15083537 | 0.15746991 | 11 | Middle Age (45-64 years) | Physical M -> Death |
| State 3 | State 7 | 0.27457971 | 0.26137278 | 0.28902097 | 11 | Middle Age (45-64 years) | Mental M -> Death |
| State 4 | State 7 | 0.31842869 | 0.31156765 | 0.32555701 | 11 | Middle Age (45-64 years) | Physical MM -> Death |
| State 5 | State 7 | 0.31126062 | 0.27987747 | 0.35020543 | 11 | Middle Age (45-64 years) | Mental MM -> Death |
| State 6 | State 7 | 0.45421515 | 0.4377732 | 0.47095353 | 11 | Middle Age (45-64 years) | Physical-Mental MM -> Death |
| State 1 | State 1 | 0.58105723 | 0.57894282 | 0.58340195 | 12 | Middle Age (45-64 years) | DF -> DF |
| State 1 | State 2 | 0.15869766 | 0.15695586 | 0.16024346 | 12 | Middle Age (45-64 years) | DF -> Physical M |
| State 2 | State 2 | 0.34865833 | 0.3428149 | 0.35374519 | 12 | Middle Age (45-64 years) | Physical M -> Physical M |
| State 1 | State 3 | 0.00753289 | 0.00717029 | 0.00792824 | 12 | Middle Age (45-64 years) | DF -> Mental M |
| State 3 | State 3 | 0.24384663 | 0.22788247 | 0.25979459 | 12 | Middle Age (45-64 years) | Mental M -> Mental M |
| State 1 | State 4 | 0.1554576 | 0.15372997 | 0.1570465 | 12 | Middle Age (45-64 years) | DF -> Physical MM |
| State 2 | State 4 | 0.42266563 | 0.41759302 | 0.42773508 | 12 | Middle Age (45-64 years) | Physical M -> Physical MM |
| State 4 | State 4 | 0.58765426 | 0.57951392 | 0.59497106 | 12 | Middle Age (45-64 years) | Physical MM -> Physical MM |
| State 1 | State 5 | 0.00113416 | 0.00100377 | 0.00127599 | 12 | Middle Age (45-64 years) | DF -> Mental MM |
| State 3 | State 5 | 0.04934042 | 0.04250216 | 0.05714123 | 12 | Middle Age (45-64 years) | Mental M -> Mental MM |
| State 5 | State 5 | 0.25128262 | 0.21251076 | 0.2905498 | 12 | Middle Age (45-64 years) | Mental MM -> Mental MM |
| State 1 | State 6 | 0.02758605 | 0.02686193 | 0.02831858 | 12 | Middle Age (45-64 years) | DF -> Physical-Mental MM |
| State 2 | State 6 | 0.05542042 | 0.0532662 | 0.05751605 | 12 | Middle Age (45-64 years) | Physical M -> Physical-Mental MM |
| State 3 | State 6 | 0.40471094 | 0.39112645 | 0.41767376 | 12 | Middle Age (45-64 years) | Mental M -> Physical-Mental MM |
| State 4 | State 6 | 0.06986579 | 0.06628784 | 0.07343422 | 12 | Middle Age (45-64 years) | Physical MM -> Physical-Mental MM |
| State 5 | State 6 | 0.40899352 | 0.37872073 | 0.4386689 | 12 | Middle Age (45-64 years) | Mental MM -> Physical-Mental MM |
| State 6 | State 6 | 0.51655234 | 0.50008628 | 0.53389459 | 12 | Middle Age (45-64 years) | Physical-Mental MM -> Physical-Mental MM |
| State 1 | State 7 | 0.06853442 | 0.06723581 | 0.06977402 | 12 | Middle Age (45-64 years) | DF -> Death |
| State 2 | State 7 | 0.17325562 | 0.169482 | 0.17719676 | 12 | Middle Age (45-64 years) | Physical M -> Death |
| State 3 | State 7 | 0.30210201 | 0.28861597 | 0.31646904 | 12 | Middle Age (45-64 years) | Mental M -> Death |
| State 4 | State 7 | 0.34247996 | 0.33546163 | 0.34980801 | 12 | Middle Age (45-64 years) | Physical MM -> Death |
| State 5 | State 7 | 0.33972386 | 0.31066729 | 0.37634868 | 12 | Middle Age (45-64 years) | Mental MM -> Death |
| State 6 | State 7 | 0.48344766 | 0.46610541 | 0.49991372 | 12 | Middle Age (45-64 years) | Physical-Mental MM -> Death |
| State 1 | State 1 | 0.55535474 | 0.5528445 | 0.55788984 | 13 | Middle Age (45-64 years) | DF -> DF |
| State 1 | State 2 | 0.16116007 | 0.15933507 | 0.1629475 | 13 | Middle Age (45-64 years) | DF -> Physical M |
| State 2 | State 2 | 0.31934985 | 0.3135996 | 0.3247956 | 13 | Middle Age (45-64 years) | Physical M -> Physical M |
| State 1 | State 3 | 0.00756309 | 0.00717028 | 0.00795211 | 13 | Middle Age (45-64 years) | DF -> Mental M |
| State 3 | State 3 | 0.21679195 | 0.20123432 | 0.23302583 | 13 | Middle Age (45-64 years) | Mental M -> Mental M |
| State 1 | State 4 | 0.16675991 | 0.1649834 | 0.16864926 | 13 | Middle Age (45-64 years) | DF -> Physical MM |
| State 2 | State 4 | 0.4294617 | 0.42458905 | 0.43460497 | 13 | Middle Age (45-64 years) | Physical M -> Physical MM |
| State 4 | State 4 | 0.56218861 | 0.55445625 | 0.56969165 | 13 | Middle Age (45-64 years) | Physical MM -> Physical MM |
| State 1 | State 5 | 0.00118442 | 0.00105312 | 0.00133032 | 13 | Middle Age (45-64 years) | DF -> Mental MM |
| State 3 | State 5 | 0.04758145 | 0.04132137 | 0.0549944 | 13 | Middle Age (45-64 years) | Mental M -> Mental MM |
| State 5 | State 5 | 0.22396286 | 0.1855015 | 0.26458241 | 13 | Middle Age (45-64 years) | Mental MM -> Mental MM |
| State 1 | State 6 | 0.03012624 | 0.02924488 | 0.03097824 | 13 | Middle Age (45-64 years) | DF -> Physical-Mental MM |
| State 2 | State 6 | 0.05857923 | 0.05622895 | 0.06086611 | 13 | Middle Age (45-64 years) | Physical M -> Physical-Mental MM |
| State 3 | State 6 | 0.40634374 | 0.39174744 | 0.42029291 | 13 | Middle Age (45-64 years) | Mental M -> Physical-Mental MM |
| State 4 | State 6 | 0.07202867 | 0.06832544 | 0.07587931 | 13 | Middle Age (45-64 years) | Physical MM -> Physical-Mental MM |
| State 5 | State 6 | 0.40845847 | 0.37695277 | 0.44003315 | 13 | Middle Age (45-64 years) | Mental MM -> Physical-Mental MM |
| State 6 | State 6 | 0.48888555 | 0.47183336 | 0.50710986 | 13 | Middle Age (45-64 years) | Physical-Mental MM -> Physical-Mental MM |
| State 1 | State 7 | 0.07785153 | 0.07647481 | 0.07934079 | 13 | Middle Age (45-64 years) | DF -> Death |
| State 2 | State 7 | 0.19260921 | 0.18887213 | 0.19691459 | 13 | Middle Age (45-64 years) | Physical M -> Death |
| State 3 | State 7 | 0.32928285 | 0.31456006 | 0.34479044 | 13 | Middle Age (45-64 years) | Mental M -> Death |
| State 4 | State 7 | 0.36578272 | 0.35848081 | 0.37362526 | 13 | Middle Age (45-64 years) | Physical MM -> Death |
| State 5 | State 7 | 0.36757868 | 0.33468999 | 0.40227443 | 13 | Middle Age (45-64 years) | Mental MM -> Death |
| State 6 | State 7 | 0.51111445 | 0.49289014 | 0.52816664 | 13 | Middle Age (45-64 years) | Physical-Mental MM -> Death |
| State 1 | State 1 | 0.53078918 | 0.52822083 | 0.53330266 | 14 | Middle Age (45-64 years) | DF -> DF |
| State 1 | State 2 | 0.16271648 | 0.16085351 | 0.16443011 | 14 | Middle Age (45-64 years) | DF -> Physical M |
| State 2 | State 2 | 0.29250507 | 0.28732496 | 0.29780083 | 14 | Middle Age (45-64 years) | Physical M -> Physical M |
| State 1 | State 3 | 0.00755164 | 0.00718777 | 0.00795173 | 14 | Middle Age (45-64 years) | DF -> Mental M |
| State 3 | State 3 | 0.19273898 | 0.17824512 | 0.20724737 | 14 | Middle Age (45-64 years) | Mental M -> Mental M |
| State 1 | State 4 | 0.17745745 | 0.17539221 | 0.17933387 | 14 | Middle Age (45-64 years) | DF -> Physical MM |
| State 2 | State 4 | 0.43385234 | 0.42891941 | 0.43877849 | 14 | Middle Age (45-64 years) | Physical M -> Physical MM |
| State 4 | State 4 | 0.5378265 | 0.52978053 | 0.54626534 | 14 | Middle Age (45-64 years) | Physical MM -> Physical MM |
| State 1 | State 5 | 0.00122691 | 0.00107921 | 0.00137329 | 14 | Middle Age (45-64 years) | DF -> Mental MM |
| State 3 | State 5 | 0.04561371 | 0.03872074 | 0.05293032 | 14 | Middle Age (45-64 years) | Mental M -> Mental MM |
| State 5 | State 5 | 0.19961333 | 0.16012008 | 0.23823419 | 14 | Middle Age (45-64 years) | Mental MM -> Mental MM |
| State 1 | State 6 | 0.03262348 | 0.03177434 | 0.03356215 | 14 | Middle Age (45-64 years) | DF -> Physical-Mental MM |
| State 2 | State 6 | 0.06147911 | 0.05922218 | 0.06393106 | 14 | Middle Age (45-64 years) | Physical M -> Physical-Mental MM |
| State 3 | State 6 | 0.4056189 | 0.39153399 | 0.41853536 | 14 | Middle Age (45-64 years) | Mental M -> Physical-Mental MM |
| State 4 | State 6 | 0.07381982 | 0.07035298 | 0.0774433 | 14 | Middle Age (45-64 years) | Physical MM -> Physical-Mental MM |
| State 5 | State 6 | 0.40562861 | 0.37638756 | 0.43470489 | 14 | Middle Age (45-64 years) | Mental MM -> Physical-Mental MM |
| State 6 | State 6 | 0.4627006 | 0.44621147 | 0.4792449 | 14 | Middle Age (45-64 years) | Physical-Mental MM -> Physical-Mental MM |
| State 1 | State 7 | 0.08763485 | 0.08604149 | 0.08913554 | 14 | Middle Age (45-64 years) | DF -> Death |
| State 2 | State 7 | 0.21216349 | 0.20759467 | 0.21657319 | 14 | Middle Age (45-64 years) | Physical M -> Death |
| State 3 | State 7 | 0.35602841 | 0.34239279 | 0.3701917 | 14 | Middle Age (45-64 years) | Mental M -> Death |
| State 4 | State 7 | 0.38835367 | 0.38042869 | 0.39569296 | 14 | Middle Age (45-64 years) | Physical MM -> Death |
| State 5 | State 7 | 0.39475806 | 0.36255964 | 0.43318452 | 14 | Middle Age (45-64 years) | Mental MM -> Death |
| State 6 | State 7 | 0.5372994 | 0.5207551 | 0.55378853 | 14 | Middle Age (45-64 years) | Physical-Mental MM -> Death |
| State 1 | State 1 | 0.50731025 | 0.50455075 | 0.50988698 | 15 | Middle Age (45-64 years) | DF -> DF |
| State 1 | State 2 | 0.16347396 | 0.16171526 | 0.16522984 | 15 | Middle Age (45-64 years) | DF -> Physical M |
| State 2 | State 2 | 0.26791687 | 0.26300215 | 0.27354565 | 15 | Middle Age (45-64 years) | Physical M -> Physical M |
| State 1 | State 3 | 0.00750485 | 0.00709977 | 0.00790745 | 15 | Middle Age (45-64 years) | DF -> Mental M |
| State 3 | State 3 | 0.17135467 | 0.15683922 | 0.18454324 | 15 | Middle Age (45-64 years) | Mental M -> Mental M |
| State 1 | State 4 | 0.18752413 | 0.18506694 | 0.18953218 | 15 | Middle Age (45-64 years) | DF -> Physical MM |
| State 2 | State 4 | 0.43611921 | 0.43046767 | 0.44131888 | 15 | Middle Age (45-64 years) | Physical M -> Physical MM |
| State 4 | State 4 | 0.51452011 | 0.50544646 | 0.52226817 | 15 | Middle Age (45-64 years) | Physical MM -> Physical MM |
| State 1 | State 5 | 0.00126198 | 0.00111769 | 0.00142661 | 15 | Middle Age (45-64 years) | DF -> Mental MM |
| State 3 | State 5 | 0.04350427 | 0.03696893 | 0.05053254 | 15 | Middle Age (45-64 years) | Mental M -> Mental MM |
| State 5 | State 5 | 0.17791111 | 0.14244884 | 0.21487915 | 15 | Middle Age (45-64 years) | Mental MM -> Mental MM |
| State 1 | State 6 | 0.03506697 | 0.03406785 | 0.03613419 | 15 | Middle Age (45-64 years) | DF -> Physical-Mental MM |
| State 2 | State 6 | 0.06412303 | 0.06169565 | 0.06685479 | 15 | Middle Age (45-64 years) | Physical M -> Physical-Mental MM |
| State 3 | State 6 | 0.40288023 | 0.38862167 | 0.41768616 | 15 | Middle Age (45-64 years) | Mental M -> Physical-Mental MM |
| State 4 | State 6 | 0.07527024 | 0.07164987 | 0.07898936 | 15 | Middle Age (45-64 years) | Physical MM -> Physical-Mental MM |
| State 5 | State 6 | 0.40087946 | 0.37198497 | 0.42825472 | 15 | Middle Age (45-64 years) | Mental MM -> Physical-Mental MM |
| State 6 | State 6 | 0.43791813 | 0.42091382 | 0.45600865 | 15 | Middle Age (45-64 years) | Physical-Mental MM -> Physical-Mental MM |
| State 1 | State 7 | 0.09785786 | 0.09629687 | 0.09956996 | 15 | Middle Age (45-64 years) | DF -> Death |
| State 2 | State 7 | 0.23184088 | 0.22711577 | 0.23663311 | 15 | Middle Age (45-64 years) | Physical M -> Death |
| State 3 | State 7 | 0.38226083 | 0.36669773 | 0.39922646 | 15 | Middle Age (45-64 years) | Mental M -> Death |
| State 4 | State 7 | 0.41020964 | 0.4025229 | 0.41858083 | 15 | Middle Age (45-64 years) | Physical MM -> Death |
| State 5 | State 7 | 0.42120943 | 0.39008486 | 0.4603918 | 15 | Middle Age (45-64 years) | Mental MM -> Death |
| State 6 | State 7 | 0.56208187 | 0.54399135 | 0.57908618 | 15 | Middle Age (45-64 years) | Physical-Mental MM -> Death |
| State 1 | State 1 | 0.48486989 | 0.48200082 | 0.48786863 | 16 | Middle Age (45-64 years) | DF -> DF |
| State 1 | State 2 | 0.16352923 | 0.16135016 | 0.165395 | 16 | Middle Age (45-64 years) | DF -> Physical M |
| State 2 | State 2 | 0.24539558 | 0.2402157 | 0.25023972 | 16 | Middle Age (45-64 years) | Physical M -> Physical M |
| State 1 | State 3 | 0.00742826 | 0.0070309 | 0.007897 | 16 | Middle Age (45-64 years) | DF -> Mental M |
| State 3 | State 3 | 0.15234295 | 0.13878738 | 0.16664752 | 16 | Middle Age (45-64 years) | Mental M -> Mental M |
| State 1 | State 4 | 0.19694208 | 0.19480007 | 0.19920421 | 16 | Middle Age (45-64 years) | DF -> Physical MM |
| State 2 | State 4 | 0.4365169 | 0.43144257 | 0.44227872 | 16 | Middle Age (45-64 years) | Physical M -> Physical MM |
| State 4 | State 4 | 0.49222369 | 0.48397591 | 0.5005988 | 16 | Middle Age (45-64 years) | Physical MM -> Physical MM |
| State 1 | State 5 | 0.00129004 | 0.00114464 | 0.00146119 | 16 | Middle Age (45-64 years) | DF -> Mental MM |
| State 3 | State 5 | 0.04130799 | 0.03534114 | 0.04848162 | 16 | Middle Age (45-64 years) | Mental M -> Mental MM |
| State 5 | State 5 | 0.15856839 | 0.12695055 | 0.19621665 | 16 | Middle Age (45-64 years) | Mental MM -> Mental MM |
| State 1 | State 6 | 0.03744725 | 0.03636165 | 0.03855245 | 16 | Middle Age (45-64 years) | DF -> Physical-Mental MM |
| State 2 | State 6 | 0.06651553 | 0.06392522 | 0.06941063 | 16 | Middle Age (45-64 years) | Physical M -> Physical-Mental MM |
| State 3 | State 6 | 0.39843271 | 0.38410648 | 0.4099899 | 16 | Middle Age (45-64 years) | Mental M -> Physical-Mental MM |
| State 4 | State 6 | 0.07640879 | 0.07250024 | 0.08022021 | 16 | Middle Age (45-64 years) | Physical MM -> Physical-Mental MM |
| State 5 | State 6 | 0.39453898 | 0.36543025 | 0.41766393 | 16 | Middle Age (45-64 years) | Mental MM -> Physical-Mental MM |
| State 6 | State 6 | 0.41446302 | 0.39662437 | 0.4315699 | 16 | Middle Age (45-64 years) | Physical-Mental MM -> Physical-Mental MM |
| State 1 | State 7 | 0.10849326 | 0.10668595 | 0.11045473 | 16 | Middle Age (45-64 years) | DF -> Death |
| State 2 | State 7 | 0.25157199 | 0.24642955 | 0.25641803 | 16 | Middle Age (45-64 years) | Physical M -> Death |
| State 3 | State 7 | 0.40791635 | 0.39163784 | 0.42453567 | 16 | Middle Age (45-64 years) | Mental M -> Death |
| State 4 | State 7 | 0.43136752 | 0.42278744 | 0.43920981 | 16 | Middle Age (45-64 years) | Physical MM -> Death |
| State 5 | State 7 | 0.44689264 | 0.41210354 | 0.48450474 | 16 | Middle Age (45-64 years) | Mental MM -> Death |
| State 6 | State 7 | 0.58553698 | 0.5684301 | 0.60337563 | 16 | Middle Age (45-64 years) | Physical-Mental MM -> Death |
| State 1 | State 1 | 0.46342215 | 0.4608355 | 0.46612892 | 17 | Middle Age (45-64 years) | DF -> DF |
| State 1 | State 2 | 0.16296955 | 0.16104335 | 0.16493115 | 17 | Middle Age (45-64 years) | DF -> Physical M |
| State 2 | State 2 | 0.22476744 | 0.22023112 | 0.22975281 | 17 | Middle Age (45-64 years) | Physical M -> Physical M |
| State 1 | State 3 | 0.00732672 | 0.00694034 | 0.0077374 | 17 | Middle Age (45-64 years) | DF -> Mental M |
| State 3 | State 3 | 0.13544056 | 0.12189184 | 0.14800052 | 17 | Middle Age (45-64 years) | Mental M -> Mental M |
| State 1 | State 4 | 0.20570066 | 0.20317961 | 0.20806663 | 17 | Middle Age (45-64 years) | DF -> Physical MM |
| State 2 | State 4 | 0.43527526 | 0.42932368 | 0.44071374 | 17 | Middle Age (45-64 years) | Physical M -> Physical MM |
| State 4 | State 4 | 0.47089347 | 0.46208514 | 0.47890825 | 17 | Middle Age (45-64 years) | Physical MM -> Physical MM |
| State 1 | State 5 | 0.00131151 | 0.00114206 | 0.00148094 | 17 | Middle Age (45-64 years) | DF -> Mental MM |
| State 3 | State 5 | 0.0390694 | 0.03285594 | 0.04598202 | 17 | Middle Age (45-64 years) | Mental M -> Mental MM |
| State 5 | State 5 | 0.14132863 | 0.11138227 | 0.17655799 | 17 | Middle Age (45-64 years) | Mental MM -> Mental MM |
| State 1 | State 6 | 0.03975609 | 0.03857317 | 0.04108879 | 17 | Middle Age (45-64 years) | DF -> Physical-Mental MM |
| State 2 | State 6 | 0.06866244 | 0.06584072 | 0.07161432 | 17 | Middle Age (45-64 years) | Physical M -> Physical-Mental MM |
| State 3 | State 6 | 0.39254644 | 0.37828989 | 0.4061477 | 17 | Middle Age (45-64 years) | Mental M -> Physical-Mental MM |
| State 4 | State 6 | 0.07726231 | 0.07361187 | 0.08104283 | 17 | Middle Age (45-64 years) | Physical MM -> Physical-Mental MM |
| State 5 | State 6 | 0.38689305 | 0.3604208 | 0.4129138 | 17 | Middle Age (45-64 years) | Mental MM -> Physical-Mental MM |
| State 6 | State 6 | 0.39226417 | 0.37382257 | 0.41066624 | 17 | Middle Age (45-64 years) | Physical-Mental MM -> Physical-Mental MM |
| State 1 | State 7 | 0.11951331 | 0.11757948 | 0.12152236 | 17 | Middle Age (45-64 years) | DF -> Death |
| State 2 | State 7 | 0.27129485 | 0.26612369 | 0.27680977 | 17 | Middle Age (45-64 years) | Physical M -> Death |
| State 3 | State 7 | 0.43294359 | 0.41755379 | 0.44980816 | 17 | Middle Age (45-64 years) | Mental M -> Death |
| State 4 | State 7 | 0.45184421 | 0.44385282 | 0.46024366 | 17 | Middle Age (45-64 years) | Physical MM -> Death |
| State 5 | State 7 | 0.47177832 | 0.43662865 | 0.50876637 | 17 | Middle Age (45-64 years) | Mental MM -> Death |
| State 6 | State 7 | 0.60773583 | 0.58933376 | 0.62617743 | 17 | Middle Age (45-64 years) | Physical-Mental MM -> Death |
| State 1 | State 1 | 0.44292314 | 0.43979306 | 0.44576698 | 18 | Middle Age (45-64 years) | DF -> DF |
| State 1 | State 2 | 0.16187362 | 0.15978116 | 0.16380585 | 18 | Middle Age (45-64 years) | DF -> Physical M |
| State 2 | State 2 | 0.20587332 | 0.20105313 | 0.21071599 | 18 | Middle Age (45-64 years) | Physical M -> Physical M |
| State 1 | State 3 | 0.00720448 | 0.0067863 | 0.00764897 | 18 | Middle Age (45-64 years) | DF -> Mental M |
| State 3 | State 3 | 0.12041349 | 0.10825624 | 0.13227843 | 18 | Middle Age (45-64 years) | Mental M -> Mental M |
| State 1 | State 4 | 0.21379544 | 0.21132872 | 0.21628838 | 18 | Middle Age (45-64 years) | DF -> Physical MM |
| State 2 | State 4 | 0.43260169 | 0.42677963 | 0.43852654 | 18 | Middle Age (45-64 years) | Physical M -> Physical MM |
| State 4 | State 4 | 0.45048759 | 0.44173516 | 0.45884186 | 18 | Middle Age (45-64 years) | Physical MM -> Physical MM |
| State 1 | State 5 | 0.00132685 | 0.00116472 | 0.00151151 | 18 | Middle Age (45-64 years) | DF -> Mental MM |
| State 3 | State 5 | 0.03682428 | 0.03127471 | 0.04326924 | 18 | Middle Age (45-64 years) | Mental M -> Mental MM |
| State 5 | State 5 | 0.1259632 | 0.09724054 | 0.15976726 | 18 | Middle Age (45-64 years) | Mental MM -> Mental MM |
| State 1 | State 6 | 0.04198643 | 0.04068735 | 0.04325469 | 18 | Middle Age (45-64 years) | DF -> Physical-Mental MM |
| State 2 | State 6 | 0.07057065 | 0.06767423 | 0.07360082 | 18 | Middle Age (45-64 years) | Physical M -> Physical-Mental MM |
| State 3 | State 6 | 0.38546023 | 0.37076977 | 0.39951834 | 18 | Middle Age (45-64 years) | Mental M -> Physical-Mental MM |
| State 4 | State 6 | 0.07785579 | 0.07401655 | 0.08179464 | 18 | Middle Age (45-64 years) | Physical MM -> Physical-Mental MM |
| State 5 | State 6 | 0.37819045 | 0.35088312 | 0.40440557 | 18 | Middle Age (45-64 years) | Mental MM -> Physical-Mental MM |
| State 6 | State 6 | 0.37125431 | 0.35298551 | 0.38922955 | 18 | Middle Age (45-64 years) | Physical-Mental MM -> Physical-Mental MM |
| State 1 | State 7 | 0.13089004 | 0.12862337 | 0.13314461 | 18 | Middle Age (45-64 years) | DF -> Death |
| State 2 | State 7 | 0.29095433 | 0.285275 | 0.29661023 | 18 | Middle Age (45-64 years) | Physical M -> Death |
| State 3 | State 7 | 0.457302 | 0.4401712 | 0.4758697 | 18 | Middle Age (45-64 years) | Mental M -> Death |
| State 4 | State 7 | 0.47165663 | 0.46304437 | 0.4800605 | 18 | Middle Age (45-64 years) | Physical MM -> Death |
| State 5 | State 7 | 0.49584635 | 0.46241919 | 0.53210357 | 18 | Middle Age (45-64 years) | Mental MM -> Death |
| State 6 | State 7 | 0.62874569 | 0.61077045 | 0.64701449 | 18 | Middle Age (45-64 years) | Physical-Mental MM -> Death |
| State 1 | State 1 | 0.42333087 | 0.42063589 | 0.42627525 | 19 | Middle Age (45-64 years) | DF -> DF |
| State 1 | State 2 | 0.16031231 | 0.15829875 | 0.16225558 | 19 | Middle Age (45-64 years) | DF -> Physical M |
| State 2 | State 2 | 0.18856746 | 0.18420314 | 0.19292484 | 19 | Middle Age (45-64 years) | Physical M -> Physical M |
| State 1 | State 3 | 0.00706526 | 0.00664105 | 0.00749692 | 19 | Middle Age (45-64 years) | DF -> Mental M |
| State 3 | State 3 | 0.10705366 | 0.09564825 | 0.11814691 | 19 | Middle Age (45-64 years) | Mental M -> Mental M |
| State 1 | State 4 | 0.22122736 | 0.21846625 | 0.22374917 | 19 | Middle Age (45-64 years) | DF -> Physical MM |
| State 2 | State 4 | 0.42868314 | 0.42274524 | 0.43448342 | 19 | Middle Age (45-64 years) | Physical M -> Physical MM |
| State 4 | State 4 | 0.43096598 | 0.42155929 | 0.44014652 | 19 | Middle Age (45-64 years) | Physical MM -> Physical MM |
| State 1 | State 5 | 0.00133652 | 0.0011809 | 0.00152645 | 19 | Middle Age (45-64 years) | DF -> Mental MM |
| State 3 | State 5 | 0.03460107 | 0.02902388 | 0.04122644 | 19 | Middle Age (45-64 years) | Mental M -> Mental MM |
| State 5 | State 5 | 0.11226832 | 0.08570163 | 0.14372236 | 19 | Middle Age (45-64 years) | Mental MM -> Mental MM |
| State 1 | State 6 | 0.04413226 | 0.04269726 | 0.04558121 | 19 | Middle Age (45-64 years) | DF -> Physical-Mental MM |
| State 2 | State 6 | 0.07224791 | 0.06943968 | 0.07554057 | 19 | Middle Age (45-64 years) | Physical M -> Physical-Mental MM |
| State 3 | State 6 | 0.37738477 | 0.36263039 | 0.39103512 | 19 | Middle Age (45-64 years) | Mental M -> Physical-Mental MM |
| State 4 | State 6 | 0.07821243 | 0.07451922 | 0.0826723 | 19 | Middle Age (45-64 years) | Physical MM -> Physical-Mental MM |
| State 5 | State 6 | 0.36864718 | 0.34086115 | 0.39207801 | 19 | Middle Age (45-64 years) | Mental MM -> Physical-Mental MM |
| State 6 | State 6 | 0.35136975 | 0.33337463 | 0.3698722 | 19 | Middle Age (45-64 years) | Physical-Mental MM -> Physical-Mental MM |
| State 1 | State 7 | 0.14259542 | 0.14027663 | 0.14495269 | 19 | Middle Age (45-64 years) | DF -> Death |
| State 2 | State 7 | 0.3105015 | 0.30498746 | 0.31636043 | 19 | Middle Age (45-64 years) | Physical M -> Death |
| State 3 | State 7 | 0.48096049 | 0.46480844 | 0.49946974 | 19 | Middle Age (45-64 years) | Mental M -> Death |
| State 4 | State 7 | 0.49082159 | 0.48247754 | 0.49972153 | 19 | Middle Age (45-64 years) | Physical MM -> Death |
| State 5 | State 7 | 0.5190845 | 0.48502527 | 0.55691174 | 19 | Middle Age (45-64 years) | Mental MM -> Death |
| State 6 | State 7 | 0.64863025 | 0.6301278 | 0.66662537 | 19 | Middle Age (45-64 years) | Physical-Mental MM -> Death |
| State 1 | State 1 | 0.40460525 | 0.40163959 | 0.40745951 | 20 | Middle Age (45-64 years) | DF -> DF |
| State 1 | State 2 | 0.15834941 | 0.15628836 | 0.16037406 | 20 | Middle Age (45-64 years) | DF -> Physical M |
| State 2 | State 2 | 0.17271634 | 0.16826878 | 0.17698753 | 20 | Middle Age (45-64 years) | Physical M -> Physical M |
| State 1 | State 3 | 0.00691228 | 0.00650194 | 0.00736178 | 20 | Middle Age (45-64 years) | DF -> Mental M |
| State 3 | State 3 | 0.09517611 | 0.08483681 | 0.10600407 | 20 | Middle Age (45-64 years) | Mental M -> Mental M |
| State 1 | State 4 | 0.22800192 | 0.22519564 | 0.23090882 | 20 | Middle Age (45-64 years) | DF -> Physical MM |
| State 2 | State 4 | 0.42368794 | 0.41765689 | 0.42969184 | 20 | Middle Age (45-64 years) | Physical M -> Physical MM |
| State 4 | State 4 | 0.41229032 | 0.40334858 | 0.42146899 | 20 | Middle Age (45-64 years) | Physical MM -> Physical MM |
| State 1 | State 5 | 0.00134099 | 0.00115163 | 0.00153111 | 20 | Middle Age (45-64 years) | DF -> Mental MM |
| State 3 | State 5 | 0.03242204 | 0.02630526 | 0.03868013 | 20 | Middle Age (45-64 years) | Mental M -> Mental MM |
| State 5 | State 5 | 0.10006236 | 0.07062337 | 0.12837305 | 20 | Middle Age (45-64 years) | Mental MM -> Mental MM |
| State 1 | State 6 | 0.04618854 | 0.04469689 | 0.04769919 | 20 | Middle Age (45-64 years) | DF -> Physical-Mental MM |
| State 2 | State 6 | 0.07370264 | 0.07055986 | 0.0767979 | 20 | Middle Age (45-64 years) | Physical M -> Physical-Mental MM |
| State 3 | State 6 | 0.3685056 | 0.35339913 | 0.38175565 | 20 | Middle Age (45-64 years) | Mental M -> Physical-Mental MM |
| State 4 | State 6 | 0.07835381 | 0.07450614 | 0.08265901 | 20 | Middle Age (45-64 years) | Physical MM -> Physical-Mental MM |
| State 5 | State 6 | 0.35845034 | 0.33058145 | 0.38071485 | 20 | Middle Age (45-64 years) | Mental MM -> Physical-Mental MM |
| State 6 | State 6 | 0.33255021 | 0.31339674 | 0.34935099 | 20 | Middle Age (45-64 years) | Physical-Mental MM -> Physical-Mental MM |
| State 1 | State 7 | 0.15460161 | 0.15197952 | 0.15722499 | 20 | Middle Age (45-64 years) | DF -> Death |
| State 2 | State 7 | 0.32989309 | 0.32400523 | 0.33612065 | 20 | Middle Age (45-64 years) | Physical M -> Death |
| State 3 | State 7 | 0.50389625 | 0.48734031 | 0.5220928 | 20 | Middle Age (45-64 years) | Mental M -> Death |
| State 4 | State 7 | 0.50935586 | 0.4999729 | 0.51810309 | 20 | Middle Age (45-64 years) | Physical MM -> Death |
| State 5 | State 7 | 0.54148729 | 0.50927737 | 0.57929253 | 20 | Middle Age (45-64 years) | Mental MM -> Death |
| State 6 | State 7 | 0.66744979 | 0.65064901 | 0.68660326 | 20 | Middle Age (45-64 years) | Physical-Mental MM -> Death |
| State 1 | State 1 | 1 | 1 | 1 | 0 | Older Adult (65 and Over) | DF -> DF |
| State 1 | State 2 | 0 | 0 | 0 | 0 | Older Adult (65 and Over) | DF -> Physical M |
| State 2 | State 2 | 1 | 1 | 1 | 0 | Older Adult (65 and Over) | Physical M -> Physical M |
| State 1 | State 3 | 0 | 0 | 0 | 0 | Older Adult (65 and Over) | DF -> Mental M |
| State 3 | State 3 | 1 | 1 | 1 | 0 | Older Adult (65 and Over) | Mental M -> Mental M |
| State 1 | State 4 | 0 | 0 | 0 | 0 | Older Adult (65 and Over) | DF -> Physical MM |
| State 2 | State 4 | 0 | 0 | 0 | 0 | Older Adult (65 and Over) | Physical M -> Physical MM |
| State 4 | State 4 | 1 | 1 | 1 | 0 | Older Adult (65 and Over) | Physical MM -> Physical MM |
| State 1 | State 5 | 0 | 0 | 0 | 0 | Older Adult (65 and Over) | DF -> Mental MM |
| State 3 | State 5 | 0 | 0 | 0 | 0 | Older Adult (65 and Over) | Mental M -> Mental MM |
| State 5 | State 5 | 1 | 1 | 1 | 0 | Older Adult (65 and Over) | Mental MM -> Mental MM |
| State 1 | State 6 | 0 | 0 | 0 | 0 | Older Adult (65 and Over) | DF -> Physical-Mental MM |
| State 2 | State 6 | 0 | 0 | 0 | 0 | Older Adult (65 and Over) | Physical M -> Physical-Mental MM |
| State 3 | State 6 | 0 | 0 | 0 | 0 | Older Adult (65 and Over) | Mental M -> Physical-Mental MM |
| State 4 | State 6 | 0 | 0 | 0 | 0 | Older Adult (65 and Over) | Physical MM -> Physical-Mental MM |
| State 5 | State 6 | 0 | 0 | 0 | 0 | Older Adult (65 and Over) | Mental MM -> Physical-Mental MM |
| State 6 | State 6 | 1 | 1 | 1 | 0 | Older Adult (65 and Over) | Physical-Mental MM -> Physical-Mental MM |
| State 1 | State 7 | 0 | 0 | 0 | 0 | Older Adult (65 and Over) | DF -> Death |
| State 2 | State 7 | 0 | 0 | 0 | 0 | Older Adult (65 and Over) | Physical M -> Death |
| State 3 | State 7 | 0 | 0 | 0 | 0 | Older Adult (65 and Over) | Mental M -> Death |
| State 4 | State 7 | 0 | 0 | 0 | 0 | Older Adult (65 and Over) | Physical MM -> Death |
| State 5 | State 7 | 0 | 0 | 0 | 0 | Older Adult (65 and Over) | Mental MM -> Death |
| State 6 | State 7 | 0 | 0 | 0 | 0 | Older Adult (65 and Over) | Physical-Mental MM -> Death |
| State 1 | State 1 | 0.88358007 | 0.88264983 | 0.88454244 | 1 | Older Adult (65 and Over) | DF -> DF |
| State 1 | State 2 | 0.04824107 | 0.04760726 | 0.0488709 | 1 | Older Adult (65 and Over) | DF -> Physical M |
| State 2 | State 2 | 0.77945198 | 0.77682537 | 0.78182207 | 1 | Older Adult (65 and Over) | Physical M -> Physical M |
| State 1 | State 3 | 0.00385506 | 0.0036965 | 0.00402337 | 1 | Older Adult (65 and Over) | DF -> Mental M |
| State 3 | State 3 | 0.7057036 | 0.69307691 | 0.71642612 | 1 | Older Adult (65 and Over) | Mental M -> Mental M |
| State 1 | State 4 | 0.03812479 | 0.03763189 | 0.03864482 | 1 | Older Adult (65 and Over) | DF -> Physical MM |
| State 2 | State 4 | 0.15757243 | 0.15559235 | 0.15961545 | 1 | Older Adult (65 and Over) | Physical M -> Physical MM |
| State 4 | State 4 | 0.85909892 | 0.85741408 | 0.86080467 | 1 | Older Adult (65 and Over) | Physical MM -> Physical MM |
| State 1 | State 5 | 0.00035445 | 0.00031083 | 0.00040449 | 1 | Older Adult (65 and Over) | DF -> Mental MM |
| State 3 | State 5 | 0.01946763 | 0.01637105 | 0.02285324 | 1 | Older Adult (65 and Over) | Mental M -> Mental MM |
| State 5 | State 5 | 0.73364127 | 0.70200695 | 0.76260172 | 1 | Older Adult (65 and Over) | Mental MM -> Mental MM |
| State 1 | State 6 | 0.0080123 | 0.0078 | 0.00823483 | 1 | Older Adult (65 and Over) | DF -> Physical-Mental MM |
| State 2 | State 6 | 0.02129311 | 0.02060111 | 0.02207003 | 1 | Older Adult (65 and Over) | Physical M -> Physical-Mental MM |
| State 3 | State 6 | 0.16651841 | 0.15828748 | 0.17594972 | 1 | Older Adult (65 and Over) | Mental M -> Physical-Mental MM |
| State 4 | State 6 | 0.03370483 | 0.03291627 | 0.03452009 | 1 | Older Adult (65 and Over) | Physical MM -> Physical-Mental MM |
| State 5 | State 6 | 0.15468852 | 0.13127091 | 0.1799404 | 1 | Older Adult (65 and Over) | Mental MM -> Physical-Mental MM |
| State 6 | State 6 | 0.79324108 | 0.78908548 | 0.79807778 | 1 | Older Adult (65 and Over) | Physical-Mental MM -> Physical-Mental MM |
| State 1 | State 7 | 0.01783225 | 0.01750333 | 0.0181404 | 1 | Older Adult (65 and Over) | DF -> Death |
| State 2 | State 7 | 0.04168247 | 0.04066297 | 0.04277807 | 1 | Older Adult (65 and Over) | Physical M -> Death |
| State 3 | State 7 | 0.10831036 | 0.10141164 | 0.11599635 | 1 | Older Adult (65 and Over) | Mental M -> Death |
| State 4 | State 7 | 0.10719625 | 0.10574455 | 0.10872499 | 1 | Older Adult (65 and Over) | Physical MM -> Death |
| State 5 | State 7 | 0.11167021 | 0.09509926 | 0.13654006 | 1 | Older Adult (65 and Over) | Mental MM -> Death |
| State 6 | State 7 | 0.20675892 | 0.20192222 | 0.21091452 | 1 | Older Adult (65 and Over) | Physical-Mental MM -> Death |
| State 1 | State 1 | 0.78071374 | 0.77899736 | 0.78242789 | 2 | Older Adult (65 and Over) | DF -> DF |
| State 1 | State 2 | 0.08022644 | 0.07923756 | 0.08122859 | 2 | Older Adult (65 and Over) | DF -> Physical M |
| State 2 | State 2 | 0.60754539 | 0.60406106 | 0.61134134 | 2 | Older Adult (65 and Over) | Physical M -> Physical M |
| State 1 | State 3 | 0.00612679 | 0.00587055 | 0.00640364 | 2 | Older Adult (65 and Over) | DF -> Mental M |
| State 3 | State 3 | 0.49801757 | 0.48093745 | 0.51318389 | 2 | Older Adult (65 and Over) | Mental M -> Mental M |
| State 1 | State 4 | 0.07404074 | 0.07315619 | 0.07492035 | 2 | Older Adult (65 and Over) | DF -> Physical MM |
| State 2 | State 4 | 0.25819044 | 0.25512642 | 0.26101345 | 2 | Older Adult (65 and Over) | Physical M -> Physical MM |
| State 4 | State 4 | 0.73805096 | 0.73506797 | 0.74075982 | 2 | Older Adult (65 and Over) | Physical MM -> Physical MM |
| State 1 | State 5 | 0.00064828 | 0.00056511 | 0.00074211 | 2 | Older Adult (65 and Over) | DF -> Mental MM |
| State 3 | State 5 | 0.02802063 | 0.02349302 | 0.03342918 | 2 | Older Adult (65 and Over) | Mental M -> Mental MM |
| State 5 | State 5 | 0.53822951 | 0.48848145 | 0.58295473 | 2 | Older Adult (65 and Over) | Mental MM -> Mental MM |
| State 1 | State 6 | 0.01644416 | 0.0160808 | 0.01685008 | 2 | Older Adult (65 and Over) | DF -> Physical-Mental MM |
| State 2 | State 6 | 0.03879848 | 0.03757361 | 0.04005016 | 2 | Older Adult (65 and Over) | Physical M -> Physical-Mental MM |
| State 3 | State 6 | 0.2526133 | 0.24056547 | 0.26522919 | 2 | Older Adult (65 and Over) | Mental M -> Physical-Mental MM |
| State 4 | State 6 | 0.05569183 | 0.05435988 | 0.05705692 | 2 | Older Adult (65 and Over) | Physical MM -> Physical-Mental MM |
| State 5 | State 6 | 0.23619117 | 0.2035117 | 0.27014769 | 2 | Older Adult (65 and Over) | Mental MM -> Physical-Mental MM |
| State 6 | State 6 | 0.62923141 | 0.62198345 | 0.63664152 | 2 | Older Adult (65 and Over) | Physical-Mental MM -> Physical-Mental MM |
| State 1 | State 7 | 0.04179986 | 0.04120417 | 0.04245387 | 2 | Older Adult (65 and Over) | DF -> Death |
| State 2 | State 7 | 0.09546568 | 0.09353987 | 0.09736014 | 2 | Older Adult (65 and Over) | Physical M -> Death |
| State 3 | State 7 | 0.22134849 | 0.21084498 | 0.23418517 | 2 | Older Adult (65 and Over) | Mental M -> Death |
| State 4 | State 7 | 0.20625721 | 0.20393264 | 0.20880217 | 2 | Older Adult (65 and Over) | Physical MM -> Death |
| State 5 | State 7 | 0.22557931 | 0.1963318 | 0.26080684 | 2 | Older Adult (65 and Over) | Mental MM -> Death |
| State 6 | State 7 | 0.37076859 | 0.36335848 | 0.37801655 | 2 | Older Adult (65 and Over) | Physical-Mental MM -> Death |
| State 1 | State 1 | 0.6898231 | 0.68767087 | 0.69198429 | 3 | Older Adult (65 and Over) | DF -> DF |
| State 1 | State 2 | 0.10019512 | 0.0989482 | 0.10142456 | 3 | Older Adult (65 and Over) | DF -> Physical M |
| State 2 | State 2 | 0.47355246 | 0.46920475 | 0.47749011 | 3 | Older Adult (65 and Over) | Physical M -> Physical M |
| State 1 | State 3 | 0.0073334 | 0.00700288 | 0.00770258 | 3 | Older Adult (65 and Over) | DF -> Mental M |
| State 3 | State 3 | 0.35145279 | 0.33228948 | 0.3687454 | 3 | Older Adult (65 and Over) | Mental M -> Mental M |
| State 1 | State 4 | 0.10601435 | 0.10488203 | 0.10717744 | 3 | Older Adult (65 and Over) | DF -> Physical MM |
| State 2 | State 4 | 0.31754354 | 0.31416625 | 0.32111747 | 3 | Older Adult (65 and Over) | Physical M -> Physical MM |
| State 4 | State 4 | 0.63405879 | 0.63065649 | 0.63775102 | 3 | Older Adult (65 and Over) | Physical MM -> Physical MM |
| State 1 | State 5 | 0.0008716 | 0.00076129 | 0.00099723 | 3 | Older Adult (65 and Over) | DF -> Mental MM |
| State 3 | State 5 | 0.03025231 | 0.02502536 | 0.03598376 | 3 | Older Adult (65 and Over) | Mental M -> Mental MM |
| State 5 | State 5 | 0.39486738 | 0.33941751 | 0.4429511 | 3 | Older Adult (65 and Over) | Mental MM -> Mental MM |
| State 1 | State 6 | 0.0246238 | 0.02412831 | 0.02515942 | 3 | Older Adult (65 and Over) | DF -> Physical-Mental MM |
| State 2 | State 6 | 0.05241535 | 0.05096307 | 0.05387559 | 3 | Older Adult (65 and Over) | Physical M -> Physical-Mental MM |
| State 3 | State 6 | 0.28764682 | 0.27555296 | 0.29913901 | 3 | Older Adult (65 and Over) | Mental M -> Physical-Mental MM |
| State 4 | State 6 | 0.06905293 | 0.06739187 | 0.07075098 | 3 | Older Adult (65 and Over) | Physical MM -> Physical-Mental MM |
| State 5 | State 6 | 0.27061447 | 0.23684608 | 0.3056868 | 3 | Older Adult (65 and Over) | Mental MM -> Physical-Mental MM |
| State 6 | State 6 | 0.49913221 | 0.49073488 | 0.50787883 | 3 | Older Adult (65 and Over) | Physical-Mental MM -> Physical-Mental MM |
| State 1 | State 7 | 0.07113864 | 0.07017412 | 0.07217374 | 3 | Older Adult (65 and Over) | DF -> Death |
| State 2 | State 7 | 0.15648865 | 0.15393566 | 0.15912241 | 3 | Older Adult (65 and Over) | Physical M -> Death |
| State 3 | State 7 | 0.33064808 | 0.31733134 | 0.34653179 | 3 | Older Adult (65 and Over) | Mental M -> Death |
| State 4 | State 7 | 0.29688829 | 0.29365669 | 0.29999564 | 3 | Older Adult (65 and Over) | Physical MM -> Death |
| State 5 | State 7 | 0.33451815 | 0.29638307 | 0.38122395 | 3 | Older Adult (65 and Over) | Mental MM -> Death |
| State 6 | State 7 | 0.50086779 | 0.49212117 | 0.50926512 | 3 | Older Adult (65 and Over) | Physical-Mental MM -> Death |
| State 1 | State 1 | 0.60951394 | 0.60688089 | 0.61206283 | 4 | Older Adult (65 and Over) | DF -> DF |
| State 1 | State 2 | 0.11137509 | 0.10996779 | 0.1126711 | 4 | Older Adult (65 and Over) | DF -> Physical M |
| State 2 | State 2 | 0.36911141 | 0.3646459 | 0.37355462 | 4 | Older Adult (65 and Over) | Physical M -> Physical M |
| State 1 | State 3 | 0.00783451 | 0.00741537 | 0.00823788 | 4 | Older Adult (65 and Over) | DF -> Mental M |
| State 3 | State 3 | 0.2480215 | 0.23133785 | 0.26417646 | 4 | Older Adult (65 and Over) | Mental M -> Mental M |
| State 1 | State 4 | 0.13316416 | 0.13163851 | 0.13463287 | 4 | Older Adult (65 and Over) | DF -> Physical MM |
| State 2 | State 4 | 0.34742012 | 0.34400409 | 0.35080835 | 4 | Older Adult (65 and Over) | Physical M -> Physical MM |
| State 4 | State 4 | 0.54471922 | 0.54064215 | 0.54930671 | 4 | Older Adult (65 and Over) | Physical MM -> Physical MM |
| State 1 | State 5 | 0.00102672 | 0.00089097 | 0.00117189 | 4 | Older Adult (65 and Over) | DF -> Mental MM |
| State 3 | State 5 | 0.0290363 | 0.02402426 | 0.0346403 | 4 | Older Adult (65 and Over) | Mental M -> Mental MM |
| State 5 | State 5 | 0.28969101 | 0.23987183 | 0.33804152 | 4 | Older Adult (65 and Over) | Mental MM -> Mental MM |
| State 1 | State 6 | 0.03212232 | 0.03143916 | 0.03276404 | 4 | Older Adult (65 and Over) | DF -> Physical-Mental MM |
| State 2 | State 6 | 0.06236416 | 0.06076548 | 0.06393791 | 4 | Older Adult (65 and Over) | Physical M -> Physical-Mental MM |
| State 3 | State 6 | 0.29137632 | 0.27979475 | 0.3028222 | 4 | Older Adult (65 and Over) | Mental M -> Physical-Mental MM |
| State 4 | State 6 | 0.07614646 | 0.07413652 | 0.07799272 | 4 | Older Adult (65 and Over) | Physical MM -> Physical-Mental MM |
| State 5 | State 6 | 0.27574397 | 0.24580615 | 0.30531499 | 4 | Older Adult (65 and Over) | Mental MM -> Physical-Mental MM |
| State 6 | State 6 | 0.39593217 | 0.38619379 | 0.4056683 | 4 | Older Adult (65 and Over) | Physical-Mental MM -> Physical-Mental MM |
| State 1 | State 7 | 0.10496327 | 0.10374999 | 0.10620117 | 4 | Older Adult (65 and Over) | DF -> Death |
| State 2 | State 7 | 0.22110431 | 0.21807075 | 0.22430079 | 4 | Older Adult (65 and Over) | Physical M -> Death |
| State 3 | State 7 | 0.43156588 | 0.41674271 | 0.44817948 | 4 | Older Adult (65 and Over) | Mental M -> Death |
| State 4 | State 7 | 0.37913432 | 0.37513057 | 0.38339773 | 4 | Older Adult (65 and Over) | Physical MM -> Death |
| State 5 | State 7 | 0.43456502 | 0.39244314 | 0.47993441 | 4 | Older Adult (65 and Over) | Mental MM -> Death |
| State 6 | State 7 | 0.60406783 | 0.5943317 | 0.61380621 | 4 | Older Adult (65 and Over) | Physical-Mental MM -> Death |
| State 1 | State 1 | 0.53855437 | 0.53559107 | 0.54145927 | 5 | Older Adult (65 and Over) | DF -> DF |
| State 1 | State 2 | 0.11621513 | 0.11473503 | 0.11778189 | 5 | Older Adult (65 and Over) | DF -> Physical M |
| State 2 | State 2 | 0.28770462 | 0.28323201 | 0.29217775 | 5 | Older Adult (65 and Over) | Physical M -> Physical M |
| State 1 | State 3 | 0.00787856 | 0.00748285 | 0.00831948 | 5 | Older Adult (65 and Over) | DF -> Mental M |
| State 3 | State 3 | 0.17502967 | 0.15994106 | 0.19018085 | 5 | Older Adult (65 and Over) | Mental M -> Mental M |
| State 1 | State 4 | 0.15518843 | 0.15354779 | 0.15686409 | 5 | Older Adult (65 and Over) | DF -> Physical MM |
| State 2 | State 4 | 0.35663003 | 0.35314725 | 0.36024675 | 5 | Older Adult (65 and Over) | Physical M -> Physical MM |
| State 4 | State 4 | 0.4679677 | 0.46329727 | 0.47221695 | 5 | Older Adult (65 and Over) | Physical MM -> Physical MM |
| State 1 | State 5 | 0.0011218 | 0.00097026 | 0.00130534 | 5 | Older Adult (65 and Over) | DF -> Mental MM |
| State 3 | State 5 | 0.02613062 | 0.02116717 | 0.03191708 | 5 | Older Adult (65 and Over) | Mental M -> Mental MM |
| State 5 | State 5 | 0.21252928 | 0.1688762 | 0.25734904 | 5 | Older Adult (65 and Over) | Mental MM -> Mental MM |
| State 1 | State 6 | 0.03868756 | 0.03792075 | 0.03951668 | 5 | Older Adult (65 and Over) | DF -> Physical-Mental MM |
| State 2 | State 6 | 0.06903908 | 0.06741391 | 0.07076736 | 5 | Older Adult (65 and Over) | Physical M -> Physical-Mental MM |
| State 3 | State 6 | 0.27692339 | 0.2661146 | 0.28765272 | 5 | Older Adult (65 and Over) | Mental M -> Physical-Mental MM |
| State 4 | State 6 | 0.07876217 | 0.07662009 | 0.08082534 | 5 | Older Adult (65 and Over) | Physical MM -> Physical-Mental MM |
| State 5 | State 6 | 0.26354332 | 0.23153699 | 0.28957314 | 5 | Older Adult (65 and Over) | Mental MM -> Physical-Mental MM |
| State 6 | State 6 | 0.31406967 | 0.30558197 | 0.32267596 | 5 | Older Adult (65 and Over) | Physical-Mental MM -> Physical-Mental MM |
| State 1 | State 7 | 0.14235415 | 0.14075335 | 0.1440583 | 5 | Older Adult (65 and Over) | DF -> Death |
| State 2 | State 7 | 0.28662627 | 0.28299083 | 0.29027315 | 5 | Older Adult (65 and Over) | Physical M -> Death |
| State 3 | State 7 | 0.52191632 | 0.50630238 | 0.53877307 | 5 | Older Adult (65 and Over) | Mental M -> Death |
| State 4 | State 7 | 0.45327014 | 0.4491269 | 0.45767091 | 5 | Older Adult (65 and Over) | Physical MM -> Death |
| State 5 | State 7 | 0.5239274 | 0.48181437 | 0.5702571 | 5 | Older Adult (65 and Over) | Mental MM -> Death |
| State 6 | State 7 | 0.68593033 | 0.67732404 | 0.69441803 | 5 | Older Adult (65 and Over) | Physical-Mental MM -> Death |
| State 1 | State 1 | 0.4758559 | 0.47277822 | 0.47909717 | 6 | Older Adult (65 and Over) | DF -> DF |
| State 1 | State 2 | 0.11656455 | 0.11502127 | 0.11811651 | 6 | Older Adult (65 and Over) | DF -> Physical M |
| State 2 | State 2 | 0.22425194 | 0.22042251 | 0.22826466 | 6 | Older Adult (65 and Over) | Physical M -> Physical M |
| State 1 | State 3 | 0.00763609 | 0.007203 | 0.00806032 | 6 | Older Adult (65 and Over) | DF -> Mental M |
| State 3 | State 3 | 0.12351907 | 0.1115042 | 0.13632069 | 6 | Older Adult (65 and Over) | Mental M -> Mental M |
| State 1 | State 4 | 0.17216679 | 0.17018726 | 0.17395908 | 6 | Older Adult (65 and Over) | DF -> Physical MM |
| State 2 | State 4 | 0.35171479 | 0.34804616 | 0.35493121 | 6 | Older Adult (65 and Over) | Physical M -> Physical MM |
| State 4 | State 4 | 0.40203054 | 0.39721581 | 0.40647392 | 6 | Older Adult (65 and Over) | Physical MM -> Physical MM |
| State 1 | State 5 | 0.00116727 | 0.00099769 | 0.00135076 | 6 | Older Adult (65 and Over) | DF -> Mental MM |
| State 3 | State 5 | 0.02257791 | 0.01801957 | 0.02751499 | 6 | Older Adult (65 and Over) | Mental M -> Mental MM |
| State 5 | State 5 | 0.15592025 | 0.11784555 | 0.19533801 | 6 | Older Adult (65 and Over) | Mental MM -> Mental MM |
| State 1 | State 6 | 0.04419426 | 0.04322901 | 0.04516901 | 6 | Older Adult (65 and Over) | DF -> Physical-Mental MM |
| State 2 | State 6 | 0.07291092 | 0.07109265 | 0.0747267 | 6 | Older Adult (65 and Over) | Physical M -> Physical-Mental MM |
| State 3 | State 6 | 0.25285478 | 0.24287081 | 0.26335791 | 6 | Older Adult (65 and Over) | Mental M -> Physical-Mental MM |
| State 4 | State 6 | 0.07825016 | 0.07615751 | 0.08038834 | 6 | Older Adult (65 and Over) | Physical MM -> Physical-Mental MM |
| State 5 | State 6 | 0.24192923 | 0.2168164 | 0.26455629 | 6 | Older Adult (65 and Over) | Mental MM -> Physical-Mental MM |
| State 6 | State 6 | 0.24913296 | 0.24113976 | 0.25812691 | 6 | Older Adult (65 and Over) | Physical-Mental MM -> Physical-Mental MM |
| State 1 | State 7 | 0.18241514 | 0.180587 | 0.1843403 | 6 | Older Adult (65 and Over) | DF -> Death |
| State 2 | State 7 | 0.35112235 | 0.34723475 | 0.35513606 | 6 | Older Adult (65 and Over) | Physical M -> Death |
| State 3 | State 7 | 0.60104824 | 0.58628793 | 0.61761307 | 6 | Older Adult (65 and Over) | Mental M -> Death |
| State 4 | State 7 | 0.5197193 | 0.51507019 | 0.52454297 | 6 | Older Adult (65 and Over) | Physical MM -> Death |
| State 5 | State 7 | 0.60215052 | 0.56125947 | 0.64522451 | 6 | Older Adult (65 and Over) | Mental MM -> Death |
| State 6 | State 7 | 0.75086704 | 0.74187309 | 0.75886024 | 6 | Older Adult (65 and Over) | Physical-Mental MM -> Death |
| State 1 | State 1 | 0.42045679 | 0.41729609 | 0.42357687 | 7 | Older Adult (65 and Over) | DF -> DF |
| State 1 | State 2 | 0.11381227 | 0.11224735 | 0.11545069 | 7 | Older Adult (65 and Over) | DF -> Physical M |
| State 2 | State 2 | 0.17479362 | 0.17141585 | 0.1786512 | 7 | Older Adult (65 and Over) | Physical M -> Physical M |
| State 1 | State 3 | 0.00722327 | 0.00678614 | 0.00766665 | 7 | Older Adult (65 and Over) | DF -> Mental M |
| State 3 | State 3 | 0.08716785 | 0.07706041 | 0.09725112 | 7 | Older Adult (65 and Over) | Mental M -> Mental M |
| State 1 | State 4 | 0.18441757 | 0.1827334 | 0.18626981 | 7 | Older Adult (65 and Over) | DF -> Physical MM |
| State 2 | State 4 | 0.33749372 | 0.33407352 | 0.34121345 | 7 | Older Adult (65 and Over) | Physical M -> Physical MM |
| State 4 | State 4 | 0.34538401 | 0.34101276 | 0.34991187 | 7 | Older Adult (65 and Over) | Physical MM -> Physical MM |
| State 1 | State 5 | 0.00117368 | 0.00100016 | 0.00138701 | 7 | Older Adult (65 and Over) | DF -> Mental MM |
| State 3 | State 5 | 0.01896871 | 0.01493285 | 0.02357192 | 7 | Older Adult (65 and Over) | Mental M -> Mental MM |
| State 5 | State 5 | 0.11438953 | 0.08266052 | 0.15001744 | 7 | Older Adult (65 and Over) | Mental MM -> Mental MM |
| State 1 | State 6 | 0.04860639 | 0.04755258 | 0.04966891 | 7 | Older Adult (65 and Over) | DF -> Physical-Mental MM |
| State 2 | State 6 | 0.07446544 | 0.07266521 | 0.07659535 | 7 | Older Adult (65 and Over) | Physical M -> Physical-Mental MM |
| State 3 | State 6 | 0.22463554 | 0.21467081 | 0.2336055 | 7 | Older Adult (65 and Over) | Mental M -> Physical-Mental MM |
| State 4 | State 6 | 0.07562161 | 0.07343204 | 0.0778351 | 7 | Older Adult (65 and Over) | Physical MM -> Physical-Mental MM |
| State 5 | State 6 | 0.21602727 | 0.19520763 | 0.23702001 | 7 | Older Adult (65 and Over) | Mental MM -> Physical-Mental MM |
| State 6 | State 6 | 0.1976225 | 0.18971208 | 0.20620813 | 7 | Older Adult (65 and Over) | Physical-Mental MM -> Physical-Mental MM |
| State 1 | State 7 | 0.22431003 | 0.22194125 | 0.22667074 | 7 | Older Adult (65 and Over) | DF -> Death |
| State 2 | State 7 | 0.41324722 | 0.40861434 | 0.41766213 | 7 | Older Adult (65 and Over) | Physical M -> Death |
| State 3 | State 7 | 0.6692279 | 0.65465355 | 0.68555542 | 7 | Older Adult (65 and Over) | Mental M -> Death |
| State 4 | State 7 | 0.57899438 | 0.57417728 | 0.58338629 | 7 | Older Adult (65 and Over) | Physical MM -> Death |
| State 5 | State 7 | 0.6695832 | 0.62851021 | 0.70777768 | 7 | Older Adult (65 and Over) | Mental MM -> Death |
| State 6 | State 7 | 0.8023775 | 0.79379187 | 0.81028792 | 7 | Older Adult (65 and Over) | Physical-Mental MM -> Death |
| State 1 | State 1 | 0.37150724 | 0.36851634 | 0.37450089 | 8 | Older Adult (65 and Over) | DF -> DF |
| State 1 | State 2 | 0.10899448 | 0.10729801 | 0.11056388 | 8 | Older Adult (65 and Over) | DF -> Physical M |
| State 2 | State 2 | 0.13624323 | 0.13267461 | 0.13974526 | 8 | Older Adult (65 and Over) | Physical M -> Physical M |
| State 1 | State 3 | 0.00671837 | 0.00632854 | 0.00715031 | 8 | Older Adult (65 and Over) | DF -> Mental M |
| State 3 | State 3 | 0.06151467 | 0.05412929 | 0.06964322 | 8 | Older Adult (65 and Over) | Mental M -> Mental M |
| State 1 | State 4 | 0.19239644 | 0.1903888 | 0.19422757 | 8 | Older Adult (65 and Over) | DF -> Physical MM |
| State 2 | State 4 | 0.31748315 | 0.31402301 | 0.32067133 | 8 | Older Adult (65 and Over) | Physical M -> Physical MM |
| State 4 | State 4 | 0.29671903 | 0.2924467 | 0.30091229 | 8 | Older Adult (65 and Over) | Physical MM -> Physical MM |
| State 1 | State 5 | 0.00115071 | 0.00098612 | 0.00135073 | 8 | Older Adult (65 and Over) | DF -> Mental MM |
| State 3 | State 5 | 0.01561318 | 0.01223946 | 0.01991279 | 8 | Older Adult (65 and Over) | Mental M -> Mental MM |
| State 5 | State 5 | 0.08392088 | 0.05724411 | 0.11404021 | 8 | Older Adult (65 and Over) | Mental MM -> Mental MM |
| State 1 | State 6 | 0.05194896 | 0.05077785 | 0.05310605 | 8 | Older Adult (65 and Over) | DF -> Physical-Mental MM |
| State 2 | State 6 | 0.07416612 | 0.07241147 | 0.07609729 | 8 | Older Adult (65 and Over) | Physical M -> Physical-Mental MM |
| State 3 | State 6 | 0.19563943 | 0.1867278 | 0.20365066 | 8 | Older Adult (65 and Over) | Mental M -> Physical-Mental MM |
| State 4 | State 6 | 0.07162727 | 0.06950308 | 0.07377947 | 8 | Older Adult (65 and Over) | Physical MM -> Physical-Mental MM |
| State 5 | State 6 | 0.18905646 | 0.16863679 | 0.20563504 | 8 | Older Adult (65 and Over) | Mental MM -> Physical-Mental MM |
| State 6 | State 6 | 0.15676229 | 0.14951089 | 0.16385993 | 8 | Older Adult (65 and Over) | Physical-Mental MM -> Physical-Mental MM |
| State 1 | State 7 | 0.2672838 | 0.26481756 | 0.26977447 | 8 | Older Adult (65 and Over) | DF -> Death |
| State 2 | State 7 | 0.47210751 | 0.46781845 | 0.47681735 | 8 | Older Adult (65 and Over) | Physical M -> Death |
| State 3 | State 7 | 0.72723272 | 0.71360823 | 0.74069085 | 8 | Older Adult (65 and Over) | Mental M -> Death |
| State 4 | State 7 | 0.6316537 | 0.62722799 | 0.63664559 | 8 | Older Adult (65 and Over) | Physical MM -> Death |
| State 5 | State 7 | 0.72702266 | 0.69096647 | 0.76415412 | 8 | Older Adult (65 and Over) | Mental MM -> Death |
| State 6 | State 7 | 0.84323771 | 0.83614007 | 0.85048911 | 8 | Older Adult (65 and Over) | Physical-Mental MM -> Death |
| State 1 | State 1 | 0.32825639 | 0.3250106 | 0.33143424 | 9 | Older Adult (65 and Over) | DF -> DF |
| State 1 | State 2 | 0.10287787 | 0.10130401 | 0.1043107 | 9 | Older Adult (65 and Over) | DF -> Physical M |
| State 2 | State 2 | 0.10619506 | 0.10314969 | 0.10891169 | 9 | Older Adult (65 and Over) | Physical M -> Physical M |
| State 1 | State 3 | 0.00617336 | 0.00578028 | 0.00656007 | 9 | Older Adult (65 and Over) | DF -> Mental M |
| State 3 | State 3 | 0.04341112 | 0.03708343 | 0.05040877 | 9 | Older Adult (65 and Over) | Mental M -> Mental M |
| State 1 | State 4 | 0.19662574 | 0.19441923 | 0.19866879 | 9 | Older Adult (65 and Over) | DF -> Physical MM |
| State 2 | State 4 | 0.29421761 | 0.29062725 | 0.29745502 | 9 | Older Adult (65 and Over) | Physical M -> Physical MM |
| State 4 | State 4 | 0.254911 | 0.25027082 | 0.2591033 | 9 | Older Adult (65 and Over) | Physical MM -> Physical MM |
| State 1 | State 5 | 0.00110668 | 0.00093859 | 0.00131345 | 9 | Older Adult (65 and Over) | DF -> Mental MM |
| State 3 | State 5 | 0.01265202 | 0.00966657 | 0.01600448 | 9 | Older Adult (65 and Over) | Mental M -> Mental MM |
| State 5 | State 5 | 0.06156782 | 0.04106266 | 0.08785326 | 9 | Older Adult (65 and Over) | Mental MM -> Mental MM |
| State 1 | State 6 | 0.05428693 | 0.05299638 | 0.05545256 | 9 | Older Adult (65 and Over) | DF -> Physical-Mental MM |
| State 2 | State 6 | 0.07243337 | 0.07042371 | 0.07437063 | 9 | Older Adult (65 and Over) | Physical M -> Physical-Mental MM |
| State 3 | State 6 | 0.16784774 | 0.15961531 | 0.17561975 | 9 | Older Adult (65 and Over) | Mental M -> Physical-Mental MM |
| State 4 | State 6 | 0.06681856 | 0.06466488 | 0.06889553 | 9 | Older Adult (65 and Over) | Physical MM -> Physical-Mental MM |
| State 5 | State 6 | 0.16294894 | 0.14496645 | 0.17913226 | 9 | Older Adult (65 and Over) | Mental MM -> Physical-Mental MM |
| State 6 | State 6 | 0.12435029 | 0.11793894 | 0.1306865 | 9 | Older Adult (65 and Over) | Physical-Mental MM -> Physical-Mental MM |
| State 1 | State 7 | 0.31067302 | 0.30797351 | 0.31352472 | 9 | Older Adult (65 and Over) | DF -> Death |
| State 2 | State 7 | 0.52715397 | 0.52296437 | 0.53170301 | 9 | Older Adult (65 and Over) | Physical M -> Death |
| State 3 | State 7 | 0.77608912 | 0.76381906 | 0.78903286 | 9 | Older Adult (65 and Over) | Mental M -> Death |
| State 4 | State 7 | 0.67827044 | 0.67379781 | 0.68331974 | 9 | Older Adult (65 and Over) | Physical MM -> Death |
| State 5 | State 7 | 0.77548323 | 0.74029446 | 0.80682034 | 9 | Older Adult (65 and Over) | Mental MM -> Death |
| State 6 | State 7 | 0.87564971 | 0.8693135 | 0.88206106 | 9 | Older Adult (65 and Over) | Physical-Mental MM -> Death |
| State 1 | State 1 | 0.2900408 | 0.28691433 | 0.29312154 | 10 | Older Adult (65 and Over) | DF -> DF |
| State 1 | State 2 | 0.0960238 | 0.09452665 | 0.097523 | 10 | Older Adult (65 and Over) | DF -> Physical M |
| State 2 | State 2 | 0.08277395 | 0.08012359 | 0.08552111 | 10 | Older Adult (65 and Over) | Physical M -> Physical M |
| State 1 | State 3 | 0.00562201 | 0.00527752 | 0.00601458 | 10 | Older Adult (65 and Over) | DF -> Mental M |
| State 3 | State 3 | 0.03063538 | 0.02607673 | 0.03594957 | 10 | Older Adult (65 and Over) | Mental M -> Mental M |
| State 1 | State 4 | 0.19764638 | 0.19559487 | 0.19978001 | 10 | Older Adult (65 and Over) | DF -> Physical MM |
| State 2 | State 4 | 0.26949544 | 0.26608708 | 0.27306532 | 10 | Older Adult (65 and Over) | Physical M -> Physical MM |
| State 4 | State 4 | 0.21899376 | 0.21498715 | 0.22318189 | 10 | Older Adult (65 and Over) | Physical MM -> Physical MM |
| State 1 | State 5 | 0.00104844 | 0.00088673 | 0.00125384 | 10 | Older Adult (65 and Over) | DF -> Mental MM |
| State 3 | State 5 | 0.01012715 | 0.00764675 | 0.01316032 | 10 | Older Adult (65 and Over) | Mental M -> Mental MM |
| State 5 | State 5 | 0.04516869 | 0.02784007 | 0.06521896 | 10 | Older Adult (65 and Over) | Mental MM -> Mental MM |
| State 1 | State 6 | 0.05570971 | 0.0544013 | 0.05696938 | 10 | Older Adult (65 and Over) | DF -> Physical-Mental MM |
| State 2 | State 6 | 0.0696349 | 0.06764037 | 0.07159624 | 10 | Older Adult (65 and Over) | Physical M -> Physical-Mental MM |
| State 3 | State 6 | 0.1423296 | 0.13537302 | 0.14959868 | 10 | Older Adult (65 and Over) | Mental M -> Physical-Mental MM |
| State 4 | State 6 | 0.06159496 | 0.0596802 | 0.06361754 | 10 | Older Adult (65 and Over) | Physical MM -> Physical-Mental MM |
| State 5 | State 6 | 0.13878163 | 0.12346038 | 0.15416234 | 10 | Older Adult (65 and Over) | Mental MM -> Physical-Mental MM |
| State 6 | State 6 | 0.09863975 | 0.0933956 | 0.10454753 | 10 | Older Adult (65 and Over) | Physical-Mental MM -> Physical-Mental MM |
| State 1 | State 7 | 0.35390885 | 0.35105734 | 0.35703759 | 10 | Older Adult (65 and Over) | DF -> Death |
| State 2 | State 7 | 0.57809571 | 0.5733625 | 0.58291998 | 10 | Older Adult (65 and Over) | Physical M -> Death |
| State 3 | State 7 | 0.81690786 | 0.8055143 | 0.82739293 | 10 | Older Adult (65 and Over) | Mental M -> Death |
| State 4 | State 7 | 0.71941128 | 0.71486252 | 0.72364607 | 10 | Older Adult (65 and Over) | Physical MM -> Death |
| State 5 | State 7 | 0.81604967 | 0.78494829 | 0.84377928 | 10 | Older Adult (65 and Over) | Mental MM -> Death |
| State 6 | State 7 | 0.90136025 | 0.89545247 | 0.9066044 | 10 | Older Adult (65 and Over) | Physical-Mental MM -> Death |
| State 1 | State 1 | 0.25627427 | 0.25335457 | 0.25932555 | 11 | Older Adult (65 and Over) | DF -> DF |
| State 1 | State 2 | 0.08883782 | 0.08734909 | 0.09025666 | 11 | Older Adult (65 and Over) | DF -> Physical M |
| State 2 | State 2 | 0.06451832 | 0.06228944 | 0.06678989 | 11 | Older Adult (65 and Over) | Physical M -> Physical M |
| State 1 | State 3 | 0.0050856 | 0.00473279 | 0.00548857 | 11 | Older Adult (65 and Over) | DF -> Mental M |
| State 3 | State 3 | 0.0216195 | 0.01791463 | 0.02586654 | 11 | Older Adult (65 and Over) | Mental M -> Mental M |
| State 1 | State 4 | 0.19598624 | 0.19394383 | 0.19798272 | 11 | Older Adult (65 and Over) | DF -> Physical MM |
| State 2 | State 4 | 0.24456614 | 0.24136567 | 0.24806611 | 11 | Older Adult (65 and Over) | Physical M -> Physical MM |
| State 4 | State 4 | 0.18813731 | 0.18449587 | 0.19197933 | 11 | Older Adult (65 and Over) | Physical MM -> Physical MM |
| State 1 | State 5 | 0.00098143 | 0.00081483 | 0.00117954 | 11 | Older Adult (65 and Over) | DF -> Mental MM |
| State 3 | State 5 | 0.0080261 | 0.00589652 | 0.01066469 | 11 | Older Adult (65 and Over) | Mental M -> Mental MM |
| State 5 | State 5 | 0.03313762 | 0.02036874 | 0.05145235 | 11 | Older Adult (65 and Over) | Mental MM -> Mental MM |
| State 1 | State 6 | 0.05631976 | 0.05487205 | 0.05773203 | 11 | Older Adult (65 and Over) | DF -> Physical-Mental MM |
| State 2 | State 6 | 0.06608307 | 0.06400253 | 0.06803828 | 11 | Older Adult (65 and Over) | Physical M -> Physical-Mental MM |
| State 3 | State 6 | 0.11956959 | 0.11303082 | 0.12652696 | 11 | Older Adult (65 and Over) | Mental M -> Physical-Mental MM |
| State 4 | State 6 | 0.0562408 | 0.05426444 | 0.05823582 | 11 | Older Adult (65 and Over) | Physical MM -> Physical-Mental MM |
| State 5 | State 6 | 0.11707437 | 0.10200217 | 0.13197874 | 11 | Older Adult (65 and Over) | Mental MM -> Physical-Mental MM |
| State 6 | State 6 | 0.07824511 | 0.0735374 | 0.08319743 | 11 | Older Adult (65 and Over) | Physical-Mental MM -> Physical-Mental MM |
| State 1 | State 7 | 0.39651487 | 0.39332214 | 0.39963223 | 11 | Older Adult (65 and Over) | DF -> Death |
| State 2 | State 7 | 0.62483247 | 0.6200695 | 0.62929213 | 11 | Older Adult (65 and Over) | Physical M -> Death |
| State 3 | State 7 | 0.85078481 | 0.84030761 | 0.86037541 | 11 | Older Adult (65 and Over) | Mental M -> Death |
| State 4 | State 7 | 0.7556219 | 0.75142431 | 0.7598133 | 11 | Older Adult (65 and Over) | Physical MM -> Death |
| State 5 | State 7 | 0.84978801 | 0.82189417 | 0.87403169 | 11 | Older Adult (65 and Over) | Mental MM -> Death |
| State 6 | State 7 | 0.92175489 | 0.91680257 | 0.9264626 | 11 | Older Adult (65 and Over) | Physical-Mental MM -> Death |
| State 1 | State 1 | 0.22643884 | 0.22350576 | 0.22921965 | 12 | Older Adult (65 and Over) | DF -> DF |
| State 1 | State 2 | 0.08160776 | 0.0803041 | 0.08294104 | 12 | Older Adult (65 and Over) | DF -> Physical M |
| State 2 | State 2 | 0.05028893 | 0.04850394 | 0.05211559 | 12 | Older Adult (65 and Over) | Physical M -> Physical M |
| State 1 | State 3 | 0.00457688 | 0.0042571 | 0.0048825 | 12 | Older Adult (65 and Over) | DF -> Mental M |
| State 3 | State 3 | 0.01525696 | 0.01222387 | 0.01833297 | 12 | Older Adult (65 and Over) | Mental M -> Mental M |
| State 1 | State 4 | 0.19214037 | 0.18981146 | 0.19430607 | 12 | Older Adult (65 and Over) | DF -> Physical MM |
| State 2 | State 4 | 0.22027281 | 0.21655145 | 0.22367388 | 12 | Older Adult (65 and Over) | Physical M -> Physical MM |
| State 4 | State 4 | 0.16162856 | 0.15767707 | 0.16538007 | 12 | Older Adult (65 and Over) | Physical MM -> Physical MM |
| State 1 | State 5 | 0.00090986 | 0.0007472 | 0.0011044 | 12 | Older Adult (65 and Over) | DF -> Mental MM |
| State 3 | State 5 | 0.00630916 | 0.00443571 | 0.00849999 | 12 | Older Adult (65 and Over) | Mental M -> Mental MM |
| State 5 | State 5 | 0.02431112 | 0.01437466 | 0.03939841 | 12 | Older Adult (65 and Over) | Mental MM -> Mental MM |
| State 1 | State 6 | 0.05622447 | 0.05493857 | 0.05768429 | 12 | Older Adult (65 and Over) | DF -> Physical-Mental MM |
| State 2 | State 6 | 0.06203666 | 0.06030802 | 0.06413442 | 12 | Older Adult (65 and Over) | Physical M -> Physical-Mental MM |
| State 3 | State 6 | 0.0996891 | 0.09371004 | 0.10541455 | 12 | Older Adult (65 and Over) | Mental M -> Physical-Mental MM |
| State 4 | State 6 | 0.05095365 | 0.04928074 | 0.05299151 | 12 | Older Adult (65 and Over) | Physical MM -> Physical-Mental MM |
| State 5 | State 6 | 0.09799421 | 0.08462663 | 0.11065917 | 12 | Older Adult (65 and Over) | Mental MM -> Physical-Mental MM |
| State 6 | State 6 | 0.06206723 | 0.05801926 | 0.06672523 | 12 | Older Adult (65 and Over) | Physical-Mental MM -> Physical-Mental MM |
| State 1 | State 7 | 0.43810183 | 0.43460038 | 0.44141185 | 12 | Older Adult (65 and Over) | DF -> Death |
| State 2 | State 7 | 0.66740159 | 0.6627042 | 0.67182376 | 12 | Older Adult (65 and Over) | Physical M -> Death |
| State 3 | State 7 | 0.87874478 | 0.87092501 | 0.88704882 | 12 | Older Adult (65 and Over) | Mental M -> Death |
| State 4 | State 7 | 0.7874178 | 0.78326449 | 0.79152051 | 12 | Older Adult (65 and Over) | Physical MM -> Death |
| State 5 | State 7 | 0.87769467 | 0.85252301 | 0.89948463 | 12 | Older Adult (65 and Over) | Mental MM -> Death |
| State 6 | State 7 | 0.93793277 | 0.93327477 | 0.94198074 | 12 | Older Adult (65 and Over) | Physical-Mental MM -> Death |
| State 1 | State 1 | 0.20007685 | 0.19715092 | 0.20283967 | 13 | Older Adult (65 and Over) | DF -> DF |
| State 1 | State 2 | 0.07453298 | 0.07335404 | 0.075771 | 13 | Older Adult (65 and Over) | DF -> Physical M |
| State 2 | State 2 | 0.03919781 | 0.03774657 | 0.04079842 | 13 | Older Adult (65 and Over) | Physical M -> Physical M |
| State 1 | State 3 | 0.00410286 | 0.00380681 | 0.00441998 | 13 | Older Adult (65 and Over) | DF -> Mental M |
| State 3 | State 3 | 0.01076689 | 0.00865022 | 0.01310403 | 13 | Older Adult (65 and Over) | Mental M -> Mental M |
| State 1 | State 4 | 0.18655965 | 0.18439196 | 0.18866356 | 13 | Older Adult (65 and Over) | DF -> Physical MM |
| State 2 | State 4 | 0.19716029 | 0.19384005 | 0.20033568 | 13 | Older Adult (65 and Over) | Physical M -> Physical MM |
| State 4 | State 4 | 0.13885492 | 0.13535223 | 0.14222337 | 13 | Older Adult (65 and Over) | Physical MM -> Physical MM |
| State 1 | State 5 | 0.00083687 | 0.00068734 | 0.00101899 | 13 | Older Adult (65 and Over) | DF -> Mental MM |
| State 3 | State 5 | 0.00492567 | 0.00352522 | 0.0069465 | 13 | Older Adult (65 and Over) | Mental M -> Mental MM |
| State 5 | State 5 | 0.01783564 | 0.01040151 | 0.03058454 | 13 | Older Adult (65 and Over) | Mental MM -> Mental MM |
| State 1 | State 6 | 0.05553048 | 0.05407557 | 0.05699894 | 13 | Older Adult (65 and Over) | DF -> Physical-Mental MM |
| State 2 | State 6 | 0.0577051 | 0.055879 | 0.0595664 | 13 | Older Adult (65 and Over) | Physical M -> Physical-Mental MM |
| State 3 | State 6 | 0.08259401 | 0.07707955 | 0.08799228 | 13 | Older Adult (65 and Over) | Mental M -> Physical-Mental MM |
| State 4 | State 6 | 0.04586619 | 0.04418621 | 0.04756805 | 13 | Older Adult (65 and Over) | Physical MM -> Physical-Mental MM |
| State 5 | State 6 | 0.08149369 | 0.07047603 | 0.09341612 | 13 | Older Adult (65 and Over) | Mental MM -> Physical-Mental MM |
| State 6 | State 6 | 0.04923428 | 0.04573283 | 0.05291393 | 13 | Older Adult (65 and Over) | Physical-Mental MM -> Physical-Mental MM |
| State 1 | State 7 | 0.47836032 | 0.47507117 | 0.48202609 | 13 | Older Adult (65 and Over) | DF -> Death |
| State 2 | State 7 | 0.70593681 | 0.70197828 | 0.71036074 | 13 | Older Adult (65 and Over) | Physical M -> Death |
| State 3 | State 7 | 0.90171342 | 0.89420317 | 0.90899437 | 13 | Older Adult (65 and Over) | Mental M -> Death |
| State 4 | State 7 | 0.81527889 | 0.8114836 | 0.8191805 | 13 | Older Adult (65 and Over) | Physical MM -> Death |
| State 5 | State 7 | 0.90067067 | 0.87896553 | 0.91812331 | 13 | Older Adult (65 and Over) | Mental MM -> Death |
| State 6 | State 7 | 0.95076572 | 0.94708607 | 0.95426717 | 13 | Older Adult (65 and Over) | Physical-Mental MM -> Death |
| State 1 | State 1 | 0.17678391 | 0.17388895 | 0.17922701 | 14 | Older Adult (65 and Over) | DF -> DF |
| State 1 | State 2 | 0.0677468 | 0.06649514 | 0.06893741 | 14 | Older Adult (65 and Over) | DF -> Physical M |
| State 2 | State 2 | 0.03055281 | 0.02921952 | 0.03183016 | 14 | Older Adult (65 and Over) | Physical M -> Physical M |
| State 1 | State 3 | 0.00366671 | 0.0034096 | 0.00396917 | 14 | Older Adult (65 and Over) | DF -> Mental M |
| State 3 | State 3 | 0.00759823 | 0.00605751 | 0.00968983 | 14 | Older Adult (65 and Over) | Mental M -> Mental M |
| State 1 | State 4 | 0.17964542 | 0.17728646 | 0.18189488 | 14 | Older Adult (65 and Over) | DF -> Physical MM |
| State 2 | State 4 | 0.17555668 | 0.1725255 | 0.1787863 | 14 | Older Adult (65 and Over) | Physical M -> Physical MM |
| State 4 | State 4 | 0.11929011 | 0.11616021 | 0.12248315 | 14 | Older Adult (65 and Over) | Physical MM -> Physical MM |
| State 1 | State 5 | 0.00076476 | 0.00062636 | 0.00093096 | 14 | Older Adult (65 and Over) | DF -> Mental MM |
| State 3 | State 5 | 0.00382328 | 0.00263499 | 0.00533978 | 14 | Older Adult (65 and Over) | Mental M -> Mental MM |
| State 5 | State 5 | 0.01308496 | 0.00703738 | 0.02234394 | 14 | Older Adult (65 and Over) | Mental MM -> Mental MM |
| State 1 | State 6 | 0.05433979 | 0.05275828 | 0.05578977 | 14 | Older Adult (65 and Over) | DF -> Physical-Mental MM |
| State 2 | State 6 | 0.05325395 | 0.05139981 | 0.05504966 | 14 | Older Adult (65 and Over) | Physical M -> Physical-Mental MM |
| State 3 | State 6 | 0.06807179 | 0.06319744 | 0.07259504 | 14 | Older Adult (65 and Over) | Mental M -> Physical-Mental MM |
| State 4 | State 6 | 0.04106302 | 0.03941323 | 0.04263598 | 14 | Older Adult (65 and Over) | Physical MM -> Physical-Mental MM |
| State 5 | State 6 | 0.06740311 | 0.05704566 | 0.07770054 | 14 | Older Adult (65 and Over) | Mental MM -> Physical-Mental MM |
| State 6 | State 6 | 0.03905465 | 0.03591628 | 0.04208051 | 14 | Older Adult (65 and Over) | Physical-Mental MM -> Physical-Mental MM |
| State 1 | State 7 | 0.51705261 | 0.5136371 | 0.52091206 | 14 | Older Adult (65 and Over) | DF -> Death |
| State 2 | State 7 | 0.74063656 | 0.73655829 | 0.74506865 | 14 | Older Adult (65 and Over) | Physical M -> Death |
| State 3 | State 7 | 0.92050669 | 0.91425031 | 0.92679115 | 14 | Older Adult (65 and Over) | Mental M -> Death |
| State 4 | State 7 | 0.83964686 | 0.83586779 | 0.84353186 | 14 | Older Adult (65 and Over) | Physical MM -> Death |
| State 5 | State 7 | 0.91951193 | 0.90064998 | 0.9351864 | 14 | Older Adult (65 and Over) | Mental MM -> Death |
| State 6 | State 7 | 0.96094535 | 0.95791949 | 0.96408372 | 14 | Older Adult (65 and Over) | Physical-Mental MM -> Death |
| State 1 | State 1 | 0.15620274 | 0.15369481 | 0.1587129 | 15 | Older Adult (65 and Over) | DF -> DF |
| State 1 | State 2 | 0.06133362 | 0.06027346 | 0.06253807 | 15 | Older Adult (65 and Over) | DF -> Physical M |
| State 2 | State 2 | 0.02381445 | 0.02281531 | 0.02498658 | 15 | Older Adult (65 and Over) | Physical M -> Physical M |
| State 1 | State 3 | 0.00326912 | 0.00301405 | 0.00352774 | 15 | Older Adult (65 and Over) | DF -> Mental M |
| State 3 | State 3 | 0.0053621 | 0.00417596 | 0.00678448 | 15 | Older Adult (65 and Over) | Mental M -> Mental M |
| State 1 | State 4 | 0.17174807 | 0.16955316 | 0.1739228 | 15 | Older Adult (65 and Over) | DF -> Physical MM |
| State 2 | State 4 | 0.15563484 | 0.15261658 | 0.15876303 | 15 | Older Adult (65 and Over) | Physical M -> Physical MM |
| State 4 | State 4 | 0.10248201 | 0.09967475 | 0.10523607 | 15 | Older Adult (65 and Over) | Physical MM -> Physical MM |
| State 1 | State 5 | 0.0006951 | 0.00056176 | 0.00084009 | 15 | Older Adult (65 and Over) | DF -> Mental MM |
| State 3 | State 5 | 0.00295284 | 0.00201168 | 0.00423287 | 15 | Older Adult (65 and Over) | Mental M -> Mental MM |
| State 5 | State 5 | 0.00959967 | 0.00485338 | 0.01697003 | 15 | Older Adult (65 and Over) | Mental MM -> Mental MM |
| State 1 | State 6 | 0.05274733 | 0.05138405 | 0.05423298 | 15 | Older Adult (65 and Over) | DF -> Physical-Mental MM |
| State 2 | State 6 | 0.04881089 | 0.04723261 | 0.05051461 | 15 | Older Adult (65 and Over) | Physical M -> Physical-Mental MM |
| State 3 | State 6 | 0.05585401 | 0.05169024 | 0.06002593 | 15 | Older Adult (65 and Over) | Mental M -> Physical-Mental MM |
| State 4 | State 6 | 0.03659353 | 0.03515576 | 0.03810548 | 15 | Older Adult (65 and Over) | Physical MM -> Physical-Mental MM |
| State 5 | State 6 | 0.05549101 | 0.04681312 | 0.06500143 | 15 | Older Adult (65 and Over) | Mental MM -> Physical-Mental MM |
| State 6 | State 6 | 0.03097975 | 0.02843649 | 0.03368981 | 15 | Older Adult (65 and Over) | Physical-Mental MM -> Physical-Mental MM |
| State 1 | State 7 | 0.55400402 | 0.55064235 | 0.55758794 | 15 | Older Adult (65 and Over) | DF -> Death |
| State 2 | State 7 | 0.77173982 | 0.76777052 | 0.77555019 | 15 | Older Adult (65 and Over) | Physical M -> Death |
| State 3 | State 7 | 0.93583105 | 0.93017879 | 0.94084681 | 15 | Older Adult (65 and Over) | Mental M -> Death |
| State 4 | State 7 | 0.86092446 | 0.85752722 | 0.86431399 | 15 | Older Adult (65 and Over) | Physical MM -> Death |
| State 5 | State 7 | 0.93490932 | 0.91889935 | 0.94725746 | 15 | Older Adult (65 and Over) | Mental MM -> Death |
| State 6 | State 7 | 0.96902025 | 0.96631019 | 0.97156351 | 15 | Older Adult (65 and Over) | Physical-Mental MM -> Death |
| State 1 | State 1 | 0.13801763 | 0.13550311 | 0.1403348 | 16 | Older Adult (65 and Over) | DF -> DF |
| State 1 | State 2 | 0.055342 | 0.05425514 | 0.05647427 | 16 | Older Adult (65 and Over) | DF -> Physical M |
| State 2 | State 2 | 0.01856222 | 0.01767245 | 0.01951618 | 16 | Older Adult (65 and Over) | Physical M -> Physical M |
| State 1 | State 3 | 0.0029092 | 0.0026827 | 0.00315037 | 16 | Older Adult (65 and Over) | DF -> Mental M |
| State 3 | State 3 | 0.00378405 | 0.00284351 | 0.00492722 | 16 | Older Adult (65 and Over) | Mental M -> Mental M |
| State 1 | State 4 | 0.16316827 | 0.16098433 | 0.16547423 | 16 | Older Adult (65 and Over) | DF -> Physical MM |
| State 2 | State 4 | 0.13745822 | 0.13470584 | 0.14043935 | 16 | Older Adult (65 and Over) | Physical M -> Physical MM |
| State 4 | State 4 | 0.08804218 | 0.08549748 | 0.09093409 | 16 | Older Adult (65 and Over) | Physical MM -> Physical MM |
| State 1 | State 5 | 0.00062896 | 0.00050207 | 0.0007755 | 16 | Older Adult (65 and Over) | DF -> Mental MM |
| State 3 | State 5 | 0.00227071 | 0.00147215 | 0.00340138 | 16 | Older Adult (65 and Over) | Mental M -> Mental MM |
| State 5 | State 5 | 0.00704271 | 0.00329047 | 0.01264251 | 16 | Older Adult (65 and Over) | Mental MM -> Mental MM |
| State 1 | State 6 | 0.05083951 | 0.04948776 | 0.0524123 | 16 | Older Adult (65 and Over) | DF -> Physical-Mental MM |
| State 2 | State 6 | 0.04447153 | 0.04294703 | 0.04619047 | 16 | Older Adult (65 and Over) | Physical M -> Physical-Mental MM |
| State 3 | State 6 | 0.04565535 | 0.04213907 | 0.04962639 | 16 | Older Adult (65 and Over) | Mental M -> Physical-Mental MM |
| State 4 | State 6 | 0.03248163 | 0.0311482 | 0.03390546 | 16 | Older Adult (65 and Over) | Physical MM -> Physical-Mental MM |
| State 5 | State 6 | 0.04550271 | 0.03759152 | 0.05362879 | 16 | Older Adult (65 and Over) | Mental MM -> Physical-Mental MM |
| State 6 | State 6 | 0.02457441 | 0.02246776 | 0.02709741 | 16 | Older Adult (65 and Over) | Physical-Mental MM -> Physical-Mental MM |
| State 1 | State 7 | 0.58909443 | 0.58541688 | 0.59258051 | 16 | Older Adult (65 and Over) | DF -> Death |
| State 2 | State 7 | 0.79950803 | 0.79548552 | 0.80309729 | 16 | Older Adult (65 and Over) | Physical M -> Death |
| State 3 | State 7 | 0.94828988 | 0.94314555 | 0.95261362 | 16 | Older Adult (65 and Over) | Mental M -> Death |
| State 4 | State 7 | 0.87947619 | 0.87613269 | 0.88259638 | 16 | Older Adult (65 and Over) | Physical MM -> Death |
| State 5 | State 7 | 0.94745458 | 0.93401978 | 0.95865047 | 16 | Older Adult (65 and Over) | Mental MM -> Death |
| State 6 | State 7 | 0.97542559 | 0.97290259 | 0.97753224 | 16 | Older Adult (65 and Over) | Physical-Mental MM -> Death |
| State 1 | State 1 | 0.12194963 | 0.119514 | 0.12404249 | 17 | Older Adult (65 and Over) | DF -> DF |
| State 1 | State 2 | 0.04979455 | 0.04889926 | 0.05082798 | 17 | Older Adult (65 and Over) | DF -> Physical M |
| State 2 | State 2 | 0.01446836 | 0.0137557 | 0.01527647 | 17 | Older Adult (65 and Over) | Physical M -> Physical M |
| State 1 | State 3 | 0.0025851 | 0.00238924 | 0.0027981 | 17 | Older Adult (65 and Over) | DF -> Mental M |
| State 3 | State 3 | 0.00267042 | 0.00199567 | 0.00349197 | 17 | Older Adult (65 and Over) | Mental M -> Mental M |
| State 1 | State 4 | 0.15415995 | 0.15199 | 0.15637578 | 17 | Older Adult (65 and Over) | DF -> Physical MM |
| State 2 | State 4 | 0.1210151 | 0.11828139 | 0.12384229 | 17 | Older Adult (65 and Over) | Physical M -> Physical MM |
| State 4 | State 4 | 0.07563694 | 0.07315893 | 0.07812004 | 17 | Older Adult (65 and Over) | Physical MM -> Physical MM |
| State 1 | State 5 | 0.00056699 | 0.00045915 | 0.00070615 | 17 | Older Adult (65 and Over) | DF -> Mental MM |
| State 3 | State 5 | 0.00173955 | 0.00113345 | 0.00265214 | 17 | Older Adult (65 and Over) | Mental M -> Mental MM |
| State 5 | State 5 | 0.00516683 | 0.00232172 | 0.0100978 | 17 | Older Adult (65 and Over) | Mental MM -> Mental MM |
| State 1 | State 6 | 0.04869352 | 0.0472421 | 0.05024317 | 17 | Older Adult (65 and Over) | DF -> Physical-Mental MM |
| State 2 | State 6 | 0.0403049 | 0.03882586 | 0.04193004 | 17 | Older Adult (65 and Over) | Physical M -> Physical-Mental MM |
| State 3 | State 6 | 0.03719707 | 0.03384761 | 0.0404946 | 17 | Older Adult (65 and Over) | Mental M -> Physical-Mental MM |
| State 4 | State 6 | 0.02873321 | 0.02747722 | 0.03006756 | 17 | Older Adult (65 and Over) | Physical MM -> Physical-Mental MM |
| State 5 | State 6 | 0.03718404 | 0.03017472 | 0.04449919 | 17 | Older Adult (65 and Over) | Mental MM -> Physical-Mental MM |
| State 6 | State 6 | 0.01949343 | 0.01761424 | 0.02151073 | 17 | Older Adult (65 and Over) | Physical-Mental MM -> Physical-Mental MM |
| State 1 | State 7 | 0.62225027 | 0.61848322 | 0.6256128 | 17 | Older Adult (65 and Over) | DF -> Death |
| State 2 | State 7 | 0.82421164 | 0.820537 | 0.82759989 | 17 | Older Adult (65 and Over) | Physical M -> Death |
| State 3 | State 7 | 0.95839296 | 0.95431586 | 0.9623056 | 17 | Older Adult (65 and Over) | Mental M -> Death |
| State 4 | State 7 | 0.89562985 | 0.89255555 | 0.8986049 | 17 | Older Adult (65 and Over) | Physical MM -> Death |
| State 5 | State 7 | 0.95764913 | 0.94650518 | 0.96721415 | 17 | Older Adult (65 and Over) | Mental MM -> Death |
| State 6 | State 7 | 0.98050657 | 0.97848927 | 0.98238576 | 17 | Older Adult (65 and Over) | Physical-Mental MM -> Death |
| State 1 | State 1 | 0.10775226 | 0.1057639 | 0.10999592 | 18 | Older Adult (65 and Over) | DF -> DF |
| State 1 | State 2 | 0.04469544 | 0.04376394 | 0.04563161 | 18 | Older Adult (65 and Over) | DF -> Physical M |
| State 2 | State 2 | 0.01127739 | 0.01065773 | 0.0119439 | 18 | Older Adult (65 and Over) | Physical M -> Physical M |
| State 1 | State 3 | 0.00229444 | 0.00210829 | 0.00248372 | 18 | Older Adult (65 and Over) | DF -> Mental M |
| State 3 | State 3 | 0.00188453 | 0.00136485 | 0.00247308 | 18 | Older Adult (65 and Over) | Mental M -> Mental M |
| State 1 | State 4 | 0.1449342 | 0.14281234 | 0.14700168 | 18 | Older Adult (65 and Over) | DF -> Physical MM |
| State 2 | State 4 | 0.10624376 | 0.10369045 | 0.10878582 | 18 | Older Adult (65 and Over) | Physical M -> Physical MM |
| State 4 | State 4 | 0.06497962 | 0.06280335 | 0.06711733 | 18 | Older Adult (65 and Over) | Physical MM -> Physical MM |
| State 1 | State 5 | 0.00050952 | 0.00041301 | 0.00062705 | 18 | Older Adult (65 and Over) | DF -> Mental MM |
| State 3 | State 5 | 0.0013282 | 0.00083756 | 0.00209023 | 18 | Older Adult (65 and Over) | Mental M -> Mental MM |
| State 5 | State 5 | 0.0037906 | 0.00169051 | 0.00749345 | 18 | Older Adult (65 and Over) | Mental MM -> Mental MM |
| State 1 | State 6 | 0.04637718 | 0.04490653 | 0.04774088 | 18 | Older Adult (65 and Over) | DF -> Physical-Mental MM |
| State 2 | State 6 | 0.03635837 | 0.0348719 | 0.03774637 | 18 | Older Adult (65 and Over) | Physical M -> Physical-Mental MM |
| State 3 | State 6 | 0.03022001 | 0.02739117 | 0.03312071 | 18 | Older Adult (65 and Over) | Mental M -> Physical-Mental MM |
| State 4 | State 6 | 0.02534169 | 0.02421274 | 0.02653763 | 18 | Older Adult (65 and Over) | Physical MM -> Physical-Mental MM |
| State 5 | State 6 | 0.03029516 | 0.02454067 | 0.03646061 | 18 | Older Adult (65 and Over) | Mental MM -> Physical-Mental MM |
| State 6 | State 6 | 0.01546299 | 0.01394302 | 0.01722916 | 18 | Older Adult (65 and Over) | Physical-Mental MM -> Physical-Mental MM |
| State 1 | State 7 | 0.65343696 | 0.64962832 | 0.6569648 | 18 | Older Adult (65 and Over) | DF -> Death |
| State 2 | State 7 | 0.84612048 | 0.84277146 | 0.84955503 | 18 | Older Adult (65 and Over) | Physical M -> Death |
| State 3 | State 7 | 0.96656727 | 0.96314243 | 0.96996777 | 18 | Older Adult (65 and Over) | Mental M -> Death |
| State 4 | State 7 | 0.90967869 | 0.90697391 | 0.91236741 | 18 | Older Adult (65 and Over) | Physical MM -> Death |
| State 5 | State 7 | 0.96591424 | 0.95667486 | 0.97348099 | 18 | Older Adult (65 and Over) | Mental MM -> Death |
| State 6 | State 7 | 0.98453701 | 0.98277084 | 0.98605698 | 18 | Older Adult (65 and Over) | Physical-Mental MM -> Death |
| State 1 | State 1 | 0.09520775 | 0.0934273 | 0.09706876 | 19 | Older Adult (65 and Over) | DF -> DF |
| State 1 | State 2 | 0.04003603 | 0.03916239 | 0.04086778 | 19 | Older Adult (65 and Over) | DF -> Physical M |
| State 2 | State 2 | 0.00879018 | 0.00828673 | 0.0092738 | 19 | Older Adult (65 and Over) | Physical M -> Physical M |
| State 1 | State 3 | 0.00203459 | 0.00187765 | 0.00219187 | 19 | Older Adult (65 and Over) | DF -> Mental M |
| State 3 | State 3 | 0.00132992 | 0.00096126 | 0.00179267 | 19 | Older Adult (65 and Over) | Mental M -> Mental M |
| State 1 | State 4 | 0.13566362 | 0.1334243 | 0.13806238 | 19 | Older Adult (65 and Over) | DF -> Physical MM |
| State 2 | State 4 | 0.09305091 | 0.09064172 | 0.09560111 | 19 | Older Adult (65 and Over) | Physical M -> Physical MM |
| State 4 | State 4 | 0.05582392 | 0.05380514 | 0.05799358 | 19 | Older Adult (65 and Over) | Physical MM -> Physical MM |
| State 1 | State 5 | 0.00045666 | 0.00035904 | 0.00056479 | 19 | Older Adult (65 and Over) | DF -> Mental MM |
| State 3 | State 5 | 0.00101111 | 0.00060627 | 0.00160638 | 19 | Older Adult (65 and Over) | Mental M -> Mental MM |
| State 5 | State 5 | 0.00278094 | 0.00112677 | 0.00559714 | 19 | Older Adult (65 and Over) | Mental MM -> Mental MM |
| State 1 | State 6 | 0.0439492 | 0.04261727 | 0.04532345 | 19 | Older Adult (65 and Over) | DF -> Physical-Mental MM |
| State 2 | State 6 | 0.03266201 | 0.03132075 | 0.03405185 | 19 | Older Adult (65 and Over) | Physical M -> Physical-Mental MM |
| State 3 | State 6 | 0.02449102 | 0.02219702 | 0.02680875 | 19 | Older Adult (65 and Over) | Mental M -> Physical-Mental MM |
| State 4 | State 6 | 0.0222922 | 0.02123971 | 0.02338232 | 19 | Older Adult (65 and Over) | Physical MM -> Physical-Mental MM |
| State 5 | State 6 | 0.02461773 | 0.0198368 | 0.02989306 | 19 | Older Adult (65 and Over) | Mental MM -> Physical-Mental MM |
| State 6 | State 6 | 0.01226588 | 0.01088603 | 0.01362921 | 19 | Older Adult (65 and Over) | Physical-Mental MM -> Physical-Mental MM |
| State 1 | State 7 | 0.68265215 | 0.67914083 | 0.68627407 | 19 | Older Adult (65 and Over) | DF -> Death |
| State 2 | State 7 | 0.8654969 | 0.86241757 | 0.86858926 | 19 | Older Adult (65 and Over) | Physical M -> Death |
| State 3 | State 7 | 0.97316796 | 0.9703264 | 0.97569827 | 19 | Older Adult (65 and Over) | Mental M -> Death |
| State 4 | State 7 | 0.92188388 | 0.91938639 | 0.92444601 | 19 | Older Adult (65 and Over) | Physical MM -> Death |
| State 5 | State 7 | 0.97260134 | 0.96467535 | 0.9787821 | 19 | Older Adult (65 and Over) | Mental MM -> Death |
| State 6 | State 7 | 0.98773412 | 0.98637079 | 0.98911397 | 19 | Older Adult (65 and Over) | Physical-Mental MM -> Death |
| State 1 | State 1 | 0.08412367 | 0.08239192 | 0.08586873 | 20 | Older Adult (65 and Over) | DF -> DF |
| State 1 | State 2 | 0.03579909 | 0.03498796 | 0.03657926 | 20 | Older Adult (65 and Over) | DF -> Physical M |
| State 2 | State 2 | 0.00685153 | 0.00645613 | 0.00728726 | 20 | Older Adult (65 and Over) | Physical M -> Physical M |
| State 1 | State 3 | 0.00180285 | 0.00165369 | 0.00195299 | 20 | Older Adult (65 and Over) | DF -> Mental M |
| State 3 | State 3 | 0.00093853 | 0.00065331 | 0.00127901 | 20 | Older Adult (65 and Over) | Mental M -> Mental M |
| State 1 | State 4 | 0.12648682 | 0.12438886 | 0.12853706 | 20 | Older Adult (65 and Over) | DF -> Physical MM |
| State 2 | State 4 | 0.08132502 | 0.0790932 | 0.0835808 | 20 | Older Adult (65 and Over) | Physical M -> Physical MM |
| State 4 | State 4 | 0.04795827 | 0.04611484 | 0.04977265 | 20 | Older Adult (65 and Over) | Physical MM -> Physical MM |
| State 1 | State 5 | 0.00040838 | 0.00033159 | 0.00051565 | 20 | Older Adult (65 and Over) | DF -> Mental MM |
| State 3 | State 5 | 0.00076768 | 0.00047306 | 0.00125207 | 20 | Older Adult (65 and Over) | Mental M -> Mental MM |
| State 5 | State 5 | 0.00204021 | 0.00087279 | 0.00426139 | 20 | Older Adult (65 and Over) | Mental MM -> Mental MM |
| State 1 | State 6 | 0.04145959 | 0.04008617 | 0.04275298 | 20 | Older Adult (65 and Over) | DF -> Physical-Mental MM |
| State 2 | State 6 | 0.02923228 | 0.02793476 | 0.03047795 | 20 | Older Adult (65 and Over) | Physical M -> Physical-Mental MM |
| State 3 | State 6 | 0.01980514 | 0.01790593 | 0.0217684 | 20 | Older Adult (65 and Over) | Mental M -> Physical-Mental MM |
| State 4 | State 6 | 0.01956462 | 0.01860294 | 0.02054614 | 20 | Older Adult (65 and Over) | Physical MM -> Physical-Mental MM |
| State 5 | State 6 | 0.01995797 | 0.01596406 | 0.02449363 | 20 | Older Adult (65 and Over) | Mental MM -> Physical-Mental MM |
| State 6 | State 6 | 0.0097298 | 0.00860256 | 0.01086374 | 20 | Older Adult (65 and Over) | Physical-Mental MM -> Physical-Mental MM |
| State 1 | State 7 | 0.70991961 | 0.70658023 | 0.71328599 | 20 | Older Adult (65 and Over) | DF -> Death |
| State 2 | State 7 | 0.88259117 | 0.87970769 | 0.88530752 | 20 | Older Adult (65 and Over) | Physical M -> Death |
| State 3 | State 7 | 0.97848865 | 0.97605897 | 0.98064591 | 20 | Older Adult (65 and Over) | Mental M -> Death |
| State 4 | State 7 | 0.93247711 | 0.93019992 | 0.93469457 | 20 | Older Adult (65 and Over) | Physical MM -> Death |
| State 5 | State 7 | 0.97800182 | 0.97164363 | 0.98308618 | 20 | Older Adult (65 and Over) | Mental MM -> Death |
| State 6 | State 7 | 0.9902702 | 0.98913626 | 0.99139744 | 20 | Older Adult (65 and Over) | Physical-Mental MM -> Death |
