# Supplementary material for: Longitudinal physical-mental multimorbidity trajectories by sociodemographic factors: a cohort study using electronic health records in Scotland (2000–2021)
Source: PLoS One. 2026 Oct 15;21(10):e0359871. doi: 10.1371/journal.pone.0359871 (PMC13671504; doi:10.1371/journal.pone.0359871)
Supplement: Supplementary file 9 [file pone.0359871.s006.docx]

**S6 Table. Top 5 most common conditions among people across all age groups with physical-mental multimorbidity.**

| **Young adults (18-24)** | **Adults (25-44)** | **Middle age (45-64)** | **Older adults (65+)** |
| --- | --- | --- | --- |
| Drug/ Alcohol misuse | Drug/ Alcohol misuse | Drug/ Alcohol misuse | Dementia |
| Anxiety | Depression | Hypertension (treated and untreated) | Hypertension (treated and untreated) |
| Depression | Anxiety | Solid Organ Cancers | Drug/ Alcohol misuse |
| Asthma | Hypertension (treated and untreated) | Chronic Kidney Disease | Chronic urinary tract infection |
| Chronic urinary tract infection | Asthma | Chronic obstructive pulmonary disease (COPD) | Chronic Kidney Disease |
