# Supplementary material for: Longitudinal physical-mental multimorbidity trajectories by sociodemographic factors: a cohort study using electronic health records in Scotland (2000–2021)
Source: PLoS One. 2026 Oct 15;21(10):e0359871. doi: 10.1371/journal.pone.0359871 (PMC13671504; doi:10.1371/journal.pone.0359871)
Supplement: Supplementary file 10 [file pone.0359871.s007.docx]

**S7 Table. Top 5 most common conditions among people across all age groups who died having multiple mental conditions.**

| **Young adults (18-24)** | **Adults (25-44)** | **Middle age (45-64)** | **Older adults (65+)** |
| --- | --- | --- | --- |
| Depression | Depression | Depression | Dementia |
| Drug/ Alcohol misuse | Drug/ Alcohol misuse | Drug/ Alcohol misuse | Drug/ Alcohol misuse |
| Anxiety | Anxiety | Anxiety | Depression |
| Schizophrenia | Bipolar disorder | Dementia | Anxiety |
| Bipolar disorder | Schizophrenia | Bipolar disorder | Bipolar disorder |
