# Supplementary material for: Longitudinal physical-mental multimorbidity trajectories by sociodemographic factors: a cohort study using electronic health records in Scotland (2000–2021)
Source: PLoS One. 2026 Oct 15;21(10):e0359871. doi: 10.1371/journal.pone.0359871 (PMC13671504; doi:10.1371/journal.pone.0359871)
Supplement: Supplementary file 11 [file pone.0359871.s011.docx]

Dear Dr Nunes, and Reviewers,

**Re:**

**Manuscript ID:** PONE-D-26-04365

**Title:** *Longitudinal physical-mental multimorbidity trajectories by sociodemographic factors: a cohort study using electronic health records in Scotland (2000-2021)*

Thank you for the opportunity to submit a revised version of our manuscript for consideration in PLOS One.

We are grateful to the reviewers for their thoughtful and constructive feedback, which we have very carefully considered. We provide below a point-by-point response to the points made. Reviewer comments are shown in **bold**, followed by our responses and revisions made in the manuscript.

We have also included a response to the editorial points.

We have uploaded a version of the revised manuscript with tracked changes and a clean unmarked copy.

**Responses to peer-review feedback:**

**Editorial comments**

**1. Please ensure that your manuscript meets PLOS ONE's style requirements, including those for file naming. The PLOS ONE style templates can be found at**

[**https://journals.plos.org/plosone/s/file?id=wjVg/PLOSOne_formatting_sample_main_body.pdf**](https://journals.plos.org/plosone/s/file?id=wjVg/PLOSOne_formatting_sample_main_body.pdf) **and**

[**https://journals.plos.org/plosone/s/file?id=ba62/PLOSOne_formatting_sample_title_authors_affiliations.pdf**](https://journals.plos.org/plosone/s/file?id=ba62/PLOSOne_formatting_sample_title_authors_affiliations.pdf)

We have formatted the paper as per the style requirement.

**2. Thank you for stating the following financial disclosure: "This work is independent research funded by the National Institute for Health and Care Research (NIHR, Artificial Intelligence for Multiple Long-Term Conditions (AIM), OPTIMising therapies, disease trajectories, and AI assisted clinical management for patients Living with complex multimorbidity (OPTIMAL study), NIHR202632). The views expressed in this publication are those of the author(s) and not necessarily those of the NHS, the National Institute for Health and Care Research or The Department of Health and Social Care."**

**Please state what role the funders took in the study.  If the funders had no role, please state: "The funders had no role in study design, data collection and analysis, decision to publish, or preparation of the manuscript."**

**If this statement is not correct you must amend it as needed.**

**Please include this amended Role of Funder statement in your cover letter; we will change the online submission form on your behalf.**

We are happy to add the proposed statement to our paper.

“The funders had no role in study design, data collection and analysis, decision to publish, or preparation of the manuscript.”

**3. We note that you have indicated that there are restrictions to data sharing for this study. PLOS only allows data to be available upon request if there are legal or ethical restrictions on sharing data publicly. For more information on unacceptable data access restrictions, please see** [**http://journals.plos.org/plosone/s/data-availability#loc-unacceptable-data-access-restrictions**](http://journals.plos.org/plosone/s/data-availability#loc-unacceptable-data-access-restrictions)**.**

**Before we proceed with your manuscript, please address the following prompts:**

a) **If there are ethical or legal restrictions on sharing a de-identified data set, please explain them in detail (e.g., data contain potentially identifying or sensitive patient information, data are owned by a third-party organization, etc.) and who has imposed them (e.g., a Research Ethics Committee or Institutional Review Board, etc.). Please also provide contact information for a data access committee, ethics committee, or other institutional body to which data requests may be sent.**
b) **If there are no restrictions, please upload the minimal anonymized data set necessary to replicate your study findings to a stable, public repository and provide us with the relevant URLs, DOIs, or accession numbers. For a list of recommended repositories, please see**

[**https://journals.plos.org/plosone/s/recommended-repositories**](https://journals.plos.org/plosone/s/recommended-repositories)**. You also have the option of uploading the data as Supporting Information files, but we would recommend depositing data directly to a data repository if possible.**

**We will update your Data Availability statement on your behalf to reflect the information you provide.**

Access to de-identified data and analysis files for this study can be obtained through an application made to the Health Informatics Centre at the University of Dundee. As data may contain potentially identifying or sensitive patient information, all research proposals are reviewed by independent panels that weigh the risk of giving access to personal pseudonymised data against the potential public benefit of the research results. Access to each dataset is only given for specific members of that projects research team so any subsequent access needs a new independent application. Information regarding the application process can be found on the Health Informatics Centre website (https://www.dundee.ac.uk/hic/data-service).

**4. If the reviewer comments include a recommendation to cite specific previously published works, please review and evaluate these publications to determine whether they are relevant and should be cited. There is no requirement to cite these works unless the editor has indicated otherwise.**

**Reviewer #1**

**In the current manuscript, the authors present a study that aims to quantify the process of progressing from no disease to a single disease to multimorbidity and eventually to death over a twenty year follow-up period. The states are further characterised by diseases being physical OR mental therefore creating two single disease states (physical and mental) and three multimorbidity states (two or more physical, two or more mental, one or more of both physical and mental). They quantify the transition probabilities using a multi-state model based on data from over 800k adults (>18) in Tayside and Fife (Scotland). The data are mainly from hospital records (ICD-10 diagnostic codes), although they also have information on age, sex assigned at birth, deprivation (SIMD), and mortality.

This research is highly relevant, as it begins to capture some of the life trajectories of how multimorbidity accumulates and in this particular case, the impact of having either a physical or a mental condition as the first type of diagnosis. This type of research is gradually gaining attention and I would suggest to the authors this paper (doi: 10.1016/j.lanhl.2025.100790 ), which makes a call for precisely this type of research and includes some relevant references that the authors might want to use to strengthen their background and discussion.

I believe that most of the general findings presented are generally correct. However, I have some concerns regarding the data, the impact of the inclusion/exclusion criteria has on the results, and some of the interpretation. I will expand on these points below.**

We thank the reviewer for the insightful and detailed comments and for recognising the importance of longitudinal research on multimorbidity trajectories.

We thank the reviewer for the suggestion of this paper (doi: 10.1016/j.lanhl.2025.100790). We reviewed the suggested publication and found it relevant to our study. We have incorporated it into the Introduction.

**1. Representativeness of data. The dataset available (80% random sample of all data) consists of nearly 1M individuals. This includes all adults (>18). The authors use the first 5 years of follow-up to identify all individuals with one or more of the 55 diagnoses that are included. Estimates in Scotland in 2005 suggest that the prevalence of people living with one or more condition was around 20% (1M people out of 5.2). The authors have excluded around 12.5% of people (identified as having one or more conditions). This would suggest that they are some of the diagnosis identified via ICD-10 are incomplete (or not available at all). It should be possible to use their information to determine how well ascertained their diagnoses are by looking at historical prevalence estimates of the 55 conditions (in 2005). The reason why this is important is because if the lack of diagnostic ascertainment varies by the type of condition (which they suggest in their discussion), this will have implications for their interpretation of the data as the conditions identified have a strong age association (which is part of what they are aiming to study). An extra point in this issue is that there might be diagnostic differences by age so age specific prevalence estimates might be ideal (but might be too difficult to obtain). As a related point, please clarify why they have a random 80% sample of the data.**

We thank the reviewer for this comprehensive observation. We agree that our ascertainment using ICD-10 codes reported by secondary care and hospital admissions, underestimates the true population prevalence compared to national estimates that include primary care and community-managed conditions; and this under-ascertainment is age and severity dependent.

We have rewritten our Discussion/Limitation section to explain how this ascertainment bias and age-related healthcare utilisation affect our findings.

“Our study has some important limitations. Our ascertainment relied primarily on ICD-10 codes from secondary care records. Within our baseline cohort, we identified approximately 12.5% of individuals as having one or more conditions, compared to historical Scottish estimates suggesting a population prevalence of roughly 20%. This discrepancy reflects the under-ascertainment of community-managed and primary-care conditions. Consequently, our findings regarding mental health primarily capture severe, hospital-contacting psychiatric conditions (e.g., serious mental illness or acute crises), which frequently present in younger adulthood. This also means our data likely misses reactive depression or anxiety in older adults that develops secondary to a physical diagnosis, as these are typically managed by General Practitioners (GPs). Moreover, this ascertainment bias is particularly more likely to impact women, who are usually present in primary care settings with mild to moderate psychiatric illness and psychological distress and consult their GPs more frequently than men [40, 41].”

As part of the information governance approval the data provider mandated that we would only have access to a random 80% of the eligible population based on our project definitions. This is primarily done to support patient privacy and confidentiality as it is then unclear who exactly in the population has data within the study dataset.

We have clarified this rationale in the Method section:

“An 80% random sample of the available data was made available by the data provider. This was primarily done to support patient privacy and confidentiality as it was then unclear who exactly in the population has data within the study dataset.”

**2. Inclusion/Exclusion criteria. The choice of including only those that are disease free makes sense from the perspective of understanding the disease state transitions from everyone starting in the disease free state. However, as the authors highlight, the probability of having one condition (or more) increases with age. This obviously means that the data selected for their analysis is strongly biased towards the younger age groups. This means that the transition probabilities that have been estimated are more likely to reflect accurately that group. This means both, the overall estimates and the age (young) specific ones. This is because the transitions estimated for the older age groups (certainly for the >65’s) are conditional transitions. Conditional on getting to that age disease free. This is a major issue as the types of multimorbidity are very different in young adults compared to the elderly (see: doi: 10.1186/s12916-020-01543-8). This is partly substantiated by their findings but it is important to realise that this approach limits what they can say about the transitions in any group beyond the 18-44 age band.**

We thank the reviewer for highlighting this epidemiological nuance. We agree that our study design, while necessary for establishing a baseline to model incident transition states, inherently skews the analytical cohort toward younger demographics.

We thank the reviewer for directing us to Zhu et al.’s (2020) paper which demonstrates how multimorbidity clusters differ across age bands. We have explicitly redefined our older-age estimates as conditional transition probabilities and refined our conclusions to avoid over-extrapolating our findings to the general elderly population.

Abstract: “Overall transition estimates were driven by the younger demographic of our baseline disease-free cohort. The findings highlight the critical role of severe, hospital-contacting mental health conditions in driving early-onset multimorbidity.”

Method: “Our cohort requires individuals to be free of all 55 conditions at baseline. Therefore, transition probabilities estimated should be interpreted as conditional probabilities, which depend on the individual having survived to that age and having no prior diseases for 5 years before follow-up.”

Discussion: “Our inclusion criteria – requiring individuals to be entirely disease-free at baseline and with no diagnoses in the 5 years prior – introduces a further healthy bias to estimates for all age groups, which is likely to be more of an issue for older age groups. Since the likelihood of multimorbidity increases with age, those who survive to age 65 without developing any of the 55 tracked conditions in the previous 5 years represent a resilient demographic. Therefore, our overall transition estimates are likely more representative of population processes for young adults aged 18-44; our older-age transition estimates should be interpreted with care.”

**3. Besides my statement above of the bias created by the inclusion/exclusion criteria used, there are some added issues regarding the interpretation of the results. The authors say that this highlights the increased importance of mental conditions on multimorbidity. This can be contested on multiple parts. First, the way the groups of conditions are created combined with the ascertainment of the different conditions suggests that there will likely be many cases of depression and anxiety that are likely not recorded. Cases with serious mental illness are much more likely to be identified and these are also more likely to be diagnosed in the younger adults. This would a) bias the identification of mental health states to the young adults, increase the chances of these being serious, and reduce the chances that the older adults with a physical condition are then identified with depression or anxiety arising from the first physical condition. Some of these issues can be explored by determining the level of ascertainment of the different conditions.**

We thank the reviewer for this comment. As explained in the methods we do not hold disease diagnosis from primary care settings but have however listed this as a limitation in our findings. We have addressed the reviewer’s comment on this across new additions to the paper as per their recommendations.

**4. Based on their findings, the most common transitions (most people) are to physical conditions. There is a very small proportion that transition to a Mental M. However, in the young it appears that the transitions to Mental M and Physical M are roughly the same (please provide the actual transition probabilities with 95% CI in a table as part of supplementary material). Given these transitions, they can work out the probability of ending with Physical and Mental MM conditional on following each one of the different routes. This way they can more directly do a comparison of what is most likely. Please provide 95%CI across all reported estimates, particularly in the Abstract.**

We have provided the transition probabilities with 95% CI in S5 Table as part of the supplementary material.

We have used our existing transition estimates to calculate and descriptively compare the total pathway probabilities for the distinct routes leading to multimorbidity by multiplying these individual probabilities using 10-year follow-up time point. We have added Table 3 and a paragraph to the Results and Discussion sections detailing these approximated pathway probabilities.

Results:

“Table 3. Conditional probability of multimorbidity pathways by age groups at t = 10 years (%)

| Pathway | Young adult (18-24 years) | Adult (25-44 years) | Middle Age (45-64 years) | Older Adult (65 and over) |
| --- | --- | --- | --- | --- |
| Mental-only multimorbidity (DF -> Mental M -> Mental MM) | 0.12 | 0.11 | 0.04 | 0.01 |
| Physical-only multimorbidity (DF -> Physical M -> Physical MM) | 0.35 | 1.47 | 6.03 | 2.59 |
| Mental-first pathway (DF -> Mental M -> Physical-mental MM) | 0.14 | 0.26 | 0.29 | 0.08 |
| Physical-first pathway (DF -> Physical M -> Physical-mental MM) | 0.21 | 0.40 | 0.73 | 0.67 |

All probabilities expressed as %. Detailed 95% confidence intervals for all transitions are provided in S5 Table.

Trajectory Pathways to Multimorbidity

We quantified the total pathway probabilities for four distinct routes leading to multimorbidity by multiplying these individual probabilities using a 10-year follow-up time point (Table 5). In 10 years, the trajectory toward physical-only multimorbidity was the most common pathway across all age groups, peaking in middle-aged adults (6.03%). Conversely, mental-only multimorbidity was most prominent among young people. It dropped from 0.12% in young adulthood and 0.11% in adulthood to 0.04% in middle age and 0.01% in older age.

Regarding complex physical-mental multimorbidity, the physical-first pathway was more probable than the mental-first pathway across all ages. However, this probability ratio narrows significantly in younger cohorts. For young adults, the risk of transitioning to a mental-first complex state is about 70% that of the physical-first route (0.14% vs 0.21%). In older adults, the mental-first route is only approximately 12% as likely (0.08% vs 0.67%). Moreover, the mental-first route showed notable persistence. It started at 0.14% in young adulthood and rose to 0.29% in middle age, suggesting that even as physical diseases increased significantly in mid-life, the mental-first route to complex multimorbidity persisted.”

Discussion:

“We found that the most common trajectory was towards physical-only multimorbidity, peaking at 6.03% in middle-aged adults. Conversely, mental-only multimorbidity was most prominent among young people. Our longitudinal analysis also reveals that while the physical-first pathway is the most frequent route to complex physical-mental multimorbidity across all age groups, the mental-first pathway serves as a critical and persistent gateway. In young adults, the probability of the mental-first pathway (0.14%) is substantial, reaching nearly 70% of the frequency of the physical-first pathway (0.21%), which establishes mental morbidity as a primary driver of incident disease at this stage of life. By middle age, while the physical-first route becomes the dominant driver (0.73% vs. 0.29%), the mental-first pathway continues to operate as a consistent risk factor for complex multi-system decline. This indicates that mental health transitions are a strong, yet less frequent, gateway to complex multimorbidity throughout life and individuals with an incident mental health condition represent a high-risk group that requires proactive, longitudinal monitoring to prevent the subsequent accumulation of physical comorbidities.”

**5. Related to the issue with their selection criteria. The analysis that looks at SIMD quantiles is likely biased in the elderly groups. This is because the chances of being disease free in the older age groups will be smaller in the more deprived quantiles creating a sample that will ‘look more similar’ as there is survival bias. This issue impacts on all their interaction estimates that incorporates SIMD.**

We thank the reviewer for this observation. We have added the following to the Discussion section:

“Our findings emphasise the role of socioeconomic deprivation in accelerating the transition to multimorbidity. However, the true extent of this disparity among older adults is likely obscured by our study design. Since there is a healthier bias in our older cohort, the apparent narrowing of socioeconomic inequalities in our older age groups is probably due to survival bias. Consequently, the impact of SIMD is most accurately reflected in our younger age groups.”

**6. The diagnostic ascertainment will likely affect women more, specifically in the mental health diagnoses that are NOT severe. I believe this is also picked up by the authors. Suggest this is highlighted earlier.**

We thank the reviewer for this suggestion. We have added the following to the Method section to introduce this earlier:

“Our case identification system primarily captures severe, hospital-contacting events and misses conditions managed entirely in primary care. Thus, the overall burden of mental health is likely underrepresented. This particularly impacts women whose primary care-managed mental health conditions such as mild to moderate depression and anxiety likely contribute significantly to overall morbidity.”

**Minor point:
The current sentence: “Ho et al.[19] demonstrated that of all the studies the most common population examined were adults of any age (42.4%), older adults (38.2%), middle-aged and older adults (14.1%), as young and middle-aged adults aged 18-64 years among the lowest with 1.8%.”, does not make sense. I would say that ‘adults of any age’ include those 18-64. Do they mean studies that ONLY included 18-64 account for 1.8%? Unclear why this would be relevant to highlight as in this study they include ‘all adults’.**

We appreciate the reviewer’s attention to this confusing phrasing. Ho et al. discovered that only 1.8% of studies restricted their inclusion criteria solely to young and middle-aged adults. While our dataset initially spans all adults, our "disease-free" baseline criteria heavily weight our incident trajectory findings toward younger adults. Highlighting this literature gap emphasises the unique value of our study's findings regarding early-life multimorbidity. We have rewritten this section to clarify the statistic and directly connect it to the strengths of our study design.

“A recent review by Ho et al. [19] revealed a significant gap in the literature on early-onset multimorbidity. While most studies focused on older adults or unrestricted age cohorts, only 1.8% restricted their research exclusively to young and middle-aged adults aged 18–64. We emphasise this gap because our study design, which tracks incident disease trajectories from a disease-free baseline, offers a unique perspective on how multimorbidity develops in this historically under-researched younger demographic.”

**Reviewer #2**
**General comment
Using electronic health records data of more than 858, 789 Scottish adults (>=18 years) in the University of Dundee Health Informatics Centre from Jan 2005 to Dec 2021, the study investigates the physical and mental health trajectories of multimorbidity. This study addressed an important research question. However, the following concerns need to be addressed.**

We thank the reviewer for their positive assessment of the importance of the research question and for their constructive suggestions.

**Comments
Definition of multimorbidity based on ICD-10 code: “A condition was considered present if a relevant ICD-10 code was found in the patient’s record, and the diagnosis date was assigned to the date of first occurrence.”.
1. Transition from old ICD-9 to ICD-10. What are the potential impacts on the diagnosis of multimorbidity?**

All diagnoses are recorded using the ICD-10 code, as ICD-9 has not been used in Scotland since 1997.

**2. Potential impact of misclassification? For example, a COPD case could be diagnosed as asthma initially; however, subsequent diagnostic testing confirmed a final diagnosis of copd. The potential impacts of misclassification need to be addressed in the discussion.**

We thank the reviewer for this insightful point. While the overall diagnostic accuracy of Scottish routine health data is demonstrably high (doi: [10.1093/ije/dyp155](https://doi.org/10.1093/ije/dyp155)), we agree the natural refinement of clinical diagnoses over time, particularly in administrative and secondary care datasets, presents a recognised systemic challenge in longitudinal trajectory modelling. Our model tracks the temporal sequence of ICD-10 codes, so a diagnostic refinement like the reviewer’s asthma-to-COPD example is statistically recorded as a “transition” between two diseases rather than a replacement. We agree this likely inflates transition probabilities between clinically adjacent or phenotypically similar conditions and have addressed this explicitly.

We have expanded the limitations to discuss the impact of diagnostic misclassification on our transition estimates, incorporating the reviewer's asthma/COPD example to show how clinical corrections may be captured as disease transitions:

“Despite the established high diagnostic validity of Scottish routine healthcare data, our reliance on longitudinal diagnosis coding data makes our transition estimates susceptible to diagnostic misclassification. In routine clinical practice, initial diagnoses are often refined as more clinical information becomes available. For example, an initial asthma diagnosis might later be corrected to chronic obstructive pulmonary disease (COPD) after definitive pulmonary function testing. Since our modelling approach tracks the temporal sequence of recorded codes, any diagnostic refinement or correction is interpreted as a disease transition. Therefore, the transition probabilities between clinically adjacent or phenotypically similar conditions, such as asthma to COPD or unipolar depression to bipolar disorder, may be inflated, representing clinical reclassification rather than accumulation of diseases.”

**Five-year lookback period: “To ensure participants enter in a comparatively disease-free state, a 5-year lookback period [23] (1 January 2000 - 31 December 2004)”
1. What ICD coding system was used during this period, ICD-9 or ICD-10?**

ICD-10 coding system was used during the whole study period, including the lookback period.

**2. How complete was the information during this lookback period in your dataset?**

Because we used Scottish national health data, the systemic completeness during this window was high. The collection of these records (SMR data) is nationally mandated, and routine audits by Public Health Scotland/Information Services Division (ISD). The completeness, accuracy, and validity of these routine national records have been formally evaluated and established in the literature (doi: [10.1093/ije/dyp155](https://doi.org/10.1093/ije/dyp155)). While the hospital data itself was highly complete during the lookback period, it does not show the presence of mild, community-managed conditions that did not generate a secondary care record.

**Follow-up period: Most of the individuals (around 70%) were younger than 45 at the baseline. After a maximum of 21 years of follow-up, those individuals were still younger than 65 years (median age at the end of follow-up was 49 years. As we know, multi-morbidity is higher among older individuals, do you have enough follow-up time for the outcomes?**

We thank the reviewer for this insightful question about the temporal scope of our study. We agree that the traditional peak of multimorbidity prevalence occurs in later life over 65 years. Since our cohort is predominantly young, most participants do not reach this high-prevalence demographic within the study window. However, rather than being a limitation of follow-up time, this is the specific strength of our study. A maximum follow-up of 21 years is an exceptionally long window, which is more than sufficient to capture the incident development and early accumulation of multimorbidity.

We have added the following to the Discussion:

“A key strength of this study lies in its exceptionally long observation window, with a maximum follow-up of 21 years. Because the majority of our baseline disease-free cohort was young (resulting in a median age of 49 at the end of follow-up), this extensive timeframe allowed us to capture the critical, early incident trajectories of multimorbidity development. By capturing 21 years of disease trajectories in an initially disease-free, younger cohort, our data specifically highlights the “blind spot” of early-to-mid-life multimorbidity – a phase of the life course that is often overlooked in traditional multimorbidity literature, which predominantly focuses on older populations – and shows that multimorbidity is not just an old person’s issue. However, despite the 21-year follow-up, most of our cohort did not reach the age band over 65 where multimorbidity prevalence traditionally peaks. Therefore, our findings primarily reflect the development of early-onset and mid-life multimorbidity and cannot be generalised to the late-life disease accumulation that this younger cohort will eventually experience.”

The opportunity to respond to this feedback has helped us further improve our paper, for which we are grateful. We believe we have responded to all the points made and as such we hope that the manuscript is now considered suitable for publication. Please do not however hesitate to contact us if you require any further clarification or revisions.

With kind regards,

Thu Nguyen & Colin McCowan on behalf of the authors
