# Supplementary material for: Longitudinal physical-mental multimorbidity trajectories by sociodemographic factors: a cohort study using electronic health records in Scotland (2000–2021)
Source: PLoS One. 2026 Oct 15;21(10):e0359871. doi: 10.1371/journal.pone.0359871 (PMC13671504; doi:10.1371/journal.pone.0359871)
Supplement: Peer review history

# Author comment: Author response to Decision Letter 0

**DOI:** 10.1371/journal.pone.0359871.r001

**Transfer Alert**

This paper was transferred from another journal. As a result, its full editorial history (including decision letters, peer reviews and author responses) may not be present.

28 Jan 2026

# Peer review: Decision Letter 0

**DOI:** 10.1371/journal.pone.0359871.r002

**Authors:** Bruno Pereira Nunes

4 May 2026

Dear Dr. Nguyen,

Thank you for submitting your manuscript to PLOS ONE. After careful consideration, we feel that it has merit but does not fully meet PLOS ONE’s publication criteria as it currently stands. Therefore, we invite you to submit a revised version of the manuscript that addresses the points raised during the review process.

The manuscript has been reassessed by two reviewers. The comments are appended below. There are major concerns about the manuscript related to the methods, results, and discussion of the paper that affect the technical soundness of your study and the conclusions of the paper. We request that authors conduct a detailed review of the comments and provide changes to the text.

Please submit your revised manuscript by Jun 18 2026 11:59PM. If you will need more time than this to complete your revisions, please reply to this message or contact the journal office at plosone@plos.org. When you're ready to submit your revision, log on to https://www.editorialmanager.com/pone/ and select the 'Submissions Needing Revision' folder to locate your manuscript file.

- A letter that responds to each point raised by the academic editor and reviewer(s). You should upload this letter as a separate file labeled 'Response to Reviewers'.
- A marked-up copy of your manuscript that highlights changes made to the original version. You should upload this as a separate file labeled 'Revised Manuscript with Track Changes'.
- An unmarked version of your revised paper without tracked changes. You should upload this as a separate file labeled 'Manuscript'.

If you would like to make changes to your financial disclosure, please include your updated statement in your cover letter. Guidelines for resubmitting your figure files are available below the reviewer comments at the end of this letter.

If applicable, we recommend that you deposit your laboratory protocols in protocols.io to enhance the reproducibility of your results. Protocols.io assigns your protocol its own identifier (DOI) so that it can be cited independently in the future. For instructions see: https://journals.plos.org/plosone/s/submission-guidelines#loc-laboratory-protocols. Additionally, PLOS ONE offers an option for publishing peer-reviewed Lab Protocol articles, which describe protocols hosted on protocols.io. Read more information on sharing protocols at https://plos.org/protocols?utm_medium=editorial-email&utm_source=authorletters&utm_campaign=protocols.

As the corresponding author, your ORCID iD is verified in the submission system and will appear in the published article. PLOS supports the use of ORCID, and we encourage all coauthors to register for an ORCID iD and use it as well. Please encourage your coauthors to verify their ORCID iD within the submission system before final acceptance, as unverified ORCID iDs will not appear in the published article. *Only*  the individual author can complete the verification step; PLOS staff *cannot*  verify ORCID iDs on behalf of authors.

We look forward to receiving your revised manuscript.

Kind regards,

Bruno Pereira Nunes, Ph.D.

Academic Editor

PLOS One

Journal Requirements:

When submitting your revision, we need you to address these additional requirements.

1. Please ensure that your manuscript meets PLOS ONE's style requirements, including those for file naming. The PLOS ONE style templates can be found at

https://journals.plos.org/plosone/s/file?id=wjVg/PLOSOne_formatting_sample_main_body.pdf and

https://journals.plos.org/plosone/s/file?id=ba62/PLOSOne_formatting_sample_title_authors_affiliations.pdf

2.Thank you for stating the following financial disclosure: "This work is independent research funded by the National Institute for Health and Care Research (NIHR, Artificial Intelligence for Multiple Long-Term Conditions (AIM), OPTIMising therapies, disease trajectories, and AI assisted clinical management for patients Living with complex multimorbidity (OPTIMAL study), NIHR202632). The views expressed in this publication are those of the author(s) and not necessarily those of the NHS, the National Institute for Health and Care Research or The Department of Health and Social Care."

Please state what role the funders took in the study.  If the funders had no role, please state: "The funders had no role in study design, data collection and analysis, decision to publish, or preparation of the manuscript."

If this statement is not correct you must amend it as needed.

Please include this amended Role of Funder statement in your cover letter; we will change the online submission form on your behalf.

3. We note that you have indicated that there are restrictions to data sharing for this study. PLOS only allows data to be available upon request if there are legal or ethical restrictions on sharing data publicly. For more information on unacceptable data access restrictions, please see http://journals.plos.org/plosone/s/data-availability#loc-unacceptable-data-access-restrictions.

Before we proceed with your manuscript, please address the following prompts:

a) If there are ethical or legal restrictions on sharing a de-identified data set, please explain them in detail (e.g., data contain potentially identifying or sensitive patient information, data are owned by a third-party organization, etc.) and who has imposed them (e.g., a Research Ethics Committee or Institutional Review Board, etc.). Please also provide contact information for a data access committee, ethics committee, or other institutional body to which data requests may be sent.

b) If there are no restrictions, please upload the minimal anonymized data set necessary to replicate your study findings to a stable, public repository and provide us with the relevant URLs, DOIs, or accession numbers. For a list of recommended repositories, please see

https://journals.plos.org/plosone/s/recommended-repositories. You also have the option of uploading the data as Supporting Information files, but we would recommend depositing data directly to a data repository if possible.

We will update your Data Availability statement on your behalf to reflect the information you provide.

4. If the reviewer comments include a recommendation to cite specific previously published works, please review and evaluate these publications to determine whether they are relevant and should be cited. There is no requirement to cite these works unless the editor has indicated otherwise.

[Note: HTML markup is below. Please do not edit.]

Reviewers' comments:

Reviewer's Responses to Questions

**Comments to the Author**

1. Is the manuscript technically sound, and do the data support the conclusions?

Reviewer #1: Partly

Reviewer #2: Partly

**********

2. Has the statistical analysis been performed appropriately and rigorously?

Reviewer #1: Yes

Reviewer #2: Yes

**********

3. Have the authors made all data underlying the findings in their manuscript fully available?

The PLOS Data policy

Reviewer #1: No

Reviewer #2: Yes

**********

4. Is the manuscript presented in an intelligible fashion and written in standard English?

Reviewer #1: Yes

Reviewer #2: Yes

**********

Reviewer #1: PLOS One: Longitudinal physical-mental multimorbidity trajectories by sociodemographic factors: a cohort study using electronic health records in Scotland (2000-2021)

Review:

In the current manuscript, the authors present a study that aims to quantify the process of progressing from no disease to a single disease to multimorbidity and eventually to death over a twenty year follow-up period. The states are further characterised by diseases being physical OR mental therefore creating two single disease states (physical and mental) and three multimorbidity states (two or more physical, two or more mental, one or more of both physical and mental). They quantify the transition probabilities using a multi-state model based on data from over 800k adults (>18) in Tayside and Fife (Scotland). The data are mainly from hospital records (ICD-10 diagnostic codes), although they also have information on age, sex assigned at birth, deprivation (SIMD), and mortality.

This research is highly relevant, as it begins to capture some of the life trajectories of how multimorbidity accumulates and in this particular case, the impact of having either a physical or a mental condition as the first type of diagnosis. This type of research is gradually gaining attention and I would suggest to the authors this paper (doi: 10.1016/j.lanhl.2025.100790 ), which makes a call for precisely this type of research and includes some relevant references that the authors might want to use to strengthen their background and discussion.

I believe that most of the general findings presented are generally correct. However, I have some concerns regarding the data, the impact of the inclusion/exclusion criteria has on the results, and some of the interpretation. I will expand on these points below.

1. Representativeness of data. The dataset available (80% random sample of all data) consists of nearly 1M individuals. This includes all adults (>18). The authors use the first 5 years of follow-up to identify all individuals with one or more of the 55 diagnoses that are included. Estimates in Scotland in 2005 suggest that the prevalence of people living with one or more condition was around 20% (1M people out of 5.2). The authors have excluded around 12.5% of people (identified as having one or more conditions). This would suggest that they are some of the diagnosis identified via ICD-10 are incomplete (or not available at all). It should be possible to use their information to determine how well ascertained their diagnoses are by looking at historical prevalence estimates of the 55 conditions (in 2005). The reason why this is important is because if the lack of diagnostic ascertainment varies by the type of condition (which they suggest in their discussion), this will have implications for their interpretation of the data as the conditions identified have a strong age association (which is part of what they are aiming to study). An extra point in this issue is that there might be diagnostic differences by age so age specific prevalence estimates might be ideal (but might be too difficult to obtain). As a related point, please clarify why they have a random 80% sample of the data.

2. Inclusion/Exclusion criteria. The choice of including only those that are disease free makes sense from the perspective of understanding the disease state transitions from everyone starting in the disease free state. However, as the authors highlight, the probability of having one condition (or more) increases with age. This obviously means that the data selected for their analysis is strongly biased towards the younger age groups. This means that the transition probabilities that have been estimated are more likely to reflect accurately that group. This means both, the overall estimates and the age (young) specific ones. This is because the transitions estimated for the older age groups (certainly for the >65’s) are conditional transitions. Conditional on getting to that age disease free. This is a major issue as the types of multimorbidity are very different in young adults compared to the elderly (see: doi: 10.1186/s12916-020-01543-8). This is partly substantiated by their findings but it is important to realise that this approach limits what they can say about the transitions in any group beyond the 18-44 age band.

3. Besides my statement above of the bias created by the inclusion/exclusion criteria used, there are some added issues regarding the interpretation of the results. The authors say that this highlights the increased importance of mental conditions on multimorbidity. This can be contested on multiple parts. First, the way the groups of conditions are created combined with the ascertainment of the different conditions suggests that there will likely be many cases of depression and anxiety that are likely not recorded. Cases with serious mental illness are much more likely to be identified and these are also more likely to be diagnosed in the younger adults. This would a) bias the identification of mental health states to the young adults, increase the chances of these being serious, and reduce the chances that the older adults with a physical condition are then identified with depression or anxiety arising from the first physical condition. Some of these issues can be explored by determining the level of ascertainment of the different conditions.

4. Based on their findings, the most common transitions (most people) are to physical conditions. There is a very small proportion that transition to a Mental M. However, in the young it appears that the transitions to Mental M and Physical M are roughly the same (please provide the actual transition probabilities with 95% CI in a table as part of supplementary material). Given these transitions, they can work out the probability of ending with Physical and Mental MM conditional on following each one of the different routes. This way they can more directly do a comparison of what is most likely. Please provide 95%CI across all reported estimates, particularly in the Abstract.

5. Related to the issue with their selection criteria. The analysis that looks at SIMD quantiles is likely biased in the elderly groups. This is because the chances of being disease free in the older age groups will be smaller in the more deprived quantiles creating a sample that will ‘look more similar’ as there is survival bias. This issue impacts on all their interaction estimates that incorporates SIMD.

6. The diagnostic ascertainment will likely affect women more, specifically in the mental health diagnoses that are NOT severe. I believe this is also picked up by the authors. Suggest this is highlighted earlier.

Minor point:

The current sentence: “Ho et al.[19] demonstrated that of all the studies the most common population examined were adults of any age (42.4%), older adults (38.2%), middle-aged and older adults (14.1%), as young and middle-aged adults aged 18-64 years among the lowest with 1.8%.”, does not make sense. I would say that ‘adults of any age’ include those 18-64. Do they mean studies that ONLY included 18-64 account for 1.8%? Unclear why this would be relevant to highlight as in this study they include ‘all adults’.

Reviewer #2: Longitudinal physical-mental multimorbidity trajectories by sociodemographic factors: a

cohort study using electronic health records in Scotland (2000-2021)

General comment

Using electronic health records data of more than 858, 789 Scottish adults (>=18 years) in the University of Dundee Health Informatics Centre from Jan 2005 to Dec 2021, the study investigates the physical and mental health trajectories of multimorbidity. This study addressed an important research question. However, the following concerns need to be addressed.

Comments

Definition of multimorbidity based on ICD-10 code: “A condition was considered present if a relevant ICD-10 code was found in the patient’s record, and the diagnosis date was assigned to the date of first occurrence.”.

1. Transition from old ICD-9 to ICD-10. What are the potential impacts on the diagnosis of multimorbidity?

2. Potential impact of misclassification? For example, a COPD case could be diagnosed as asthma initially; however, subsequent diagnostic testing confirmed a final diagnosis of copd. The potential impacts of misclassification need to be addressed in the discussion.

Five-year lookback period: “To ensure participants enter in a comparatively disease-free state, a 5-year lookback period [23] (1 January 2000 - 31 December 2004)”

1. What ICD coding system was used during this period, ICD-9 or ICD-10?

2. How complete was the information during this lookback period in your dataset?

Follow-up period: Most of the individuals (around 70%) were younger than 45 at the baseline. After a maximum of 21 years of follow-up, those individuals were still younger than 65 years (median age at the end of follow-up was 49 years. As we know, multi-morbidity is higher among older individuals, do you have enough follow-up time for the outcomes?

**********

what does this mean?). If published, this will include your full peer review and any attached files.

If you choose “no”, your identity will remain anonymous but your review may still be made public.

**Do you want your identity to be public for this peer review?**  For information about this choice, including consent withdrawal, please see our Privacy Policy

Reviewer #1: **Yes:**  Rafael Perera Salazar

Reviewer #2: No

**********

[NOTE: If reviewer comments were submitted as an attachment file, they will be attached to this email and accessible via the submission site. Please log into your account, locate the manuscript record, and check for the action link "View Attachments". If this link does not appear, there are no attachment files.]

To ensure your figures meet our technical requirements, please review our figure guidelines: https://journals.plos.org/plosone/s/figures

You may also use PLOS’s free figure tool, NAAS, to help you prepare publication quality figures: https://journals.plos.org/plosone/s/figures#loc-tools-for-figure-preparation.

NAAS will assess whether your figures meet our technical requirements by comparing each figure against our figure specifications.

# Author comment: Author response to Decision Letter 1

**DOI:** 10.1371/journal.pone.0359871.r003

22 Jul 2026

I have uploaded a letter that responds to each point raised by the reviewers (file labeled 'Response to Reviewers').

Attachment Submitted filename: Response to Reviewers.docx

# Peer review: Decision Letter 1

**DOI:** 10.1371/journal.pone.0359871.r004

**Authors:** Bruno Pereira Nunes

20 Sep 2026

Longitudinal physical-mental multimorbidity trajectories by sociodemographic factors: a cohort study using electronic health records in Scotland (2000-2021)

PONE-D-26-04365R1

Dear Dr. Nguyen,

We’re pleased to inform you that your manuscript has been judged scientifically suitable for publication and will be formally accepted for publication once it meets all outstanding technical requirements.

Within one week, you’ll receive an e-mail detailing the required amendments. When these have been addressed, you’ll receive a formal acceptance letter and your manuscript will be scheduled for publication.

An invoice will be generated when your article is formally accepted. Please note, if your institution has a publishing partnership with PLOS and your article meets the relevant criteria, all or part of your publication costs will be covered. Please make sure your user information is up-to-date by logging into Editorial Manager at Editorial Manager® and clicking the ‘Update My Information' link at the top of the page. For questions related to billing, please contact billing support.

If your institution or institutions have a press office, please notify them about your upcoming paper to help maximize its impact. If they’ll be preparing press materials, please inform our press team as soon as possible -- no later than 48 hours after receiving the formal acceptance. Your manuscript will remain under strict press embargo until 2 pm Eastern Time on the date of publication. For more information, please contact onepress@plos.org.

Kind regards,

Bruno Pereira Nunes, Ph.D.

Academic Editor

PLOS One

Additional Editor Comments (optional):

Reviewers' comments:

Reviewer's Responses to Questions

**Comments to the Author**

Reviewer #1: All comments have been addressed

**********

2. Is the manuscript technically sound, and do the data support the conclusions?

Reviewer #1: Yes

**********

3. Has the statistical analysis been performed appropriately and rigorously?

Reviewer #1: Yes

**********

4. Have the authors made all data underlying the findings in their manuscript fully available?

The PLOS Data policy

Reviewer #1: No

**********

5. Is the manuscript presented in an intelligible fashion and written in standard English?

Reviewer #1: Yes

**********

Reviewer #1: Many thanks to the authors as they have kindly replied to all my queries and questions. I find the manuscript insightful and useful.

**********

what does this mean?). If published, this will include your full peer review and any attached files.

If you choose “no”, your identity will remain anonymous but your review may still be made public.

**Do you want your identity to be public for this peer review?**  For information about this choice, including consent withdrawal, please see our Privacy Policy

Reviewer #1: **Yes:**  Rafael Perera

**********

# Editor report: Acceptance letter

**DOI:** 10.1371/journal.pone.0359871.r005

**Authors:** Bruno Pereira Nunes

PONE-D-26-04365R1

PLOS One

Dear Dr. Nguyen,

I'm pleased to inform you that your manuscript has been deemed suitable for publication in PLOS One. Congratulations! Your manuscript is now being handed over to our production team.

At this stage, our production department will prepare your paper for publication. This includes ensuring the following:

* All references, tables, and figures are properly cited

* All relevant supporting information is included in the manuscript submission,

* There are no issues that prevent the paper from being properly typeset

You will receive further instructions from the production team, including instructions on how to review your proof when it is ready. Please keep in mind that we are working through a large volume of accepted articles, so please give us a few days to review your paper and let you know the next and final steps.

Lastly, if your institution or institutions have a press office, please let them know about your upcoming paper now to help maximize its impact. If they'll be preparing press materials, please inform our press team within the next 48 hours. Your manuscript will remain under strict press embargo until 2 pm Eastern Time on the date of publication. For more information, please contact onepress@plos.org.

You will receive an invoice from PLOS for your publication fee after your manuscript has reached the completed accept phase. If you receive an email requesting payment before acceptance or for any other service, this may be a phishing scheme. Learn how to identify phishing emails and protect your accounts at https://explore.plos.org/phishing.

If we can help with anything else, please email us at customercare@plos.org.

Thank you for submitting your work to PLOS One and supporting open access.

Kind regards,

PLOS One Editorial Office Staff

on behalf of

Dr. Bruno Pereira Nunes

Academic Editor

PLOS One
